# Estimation of Undetected Asymptomatic COVID-19 Cases in South Korea Using a Probabilistic Model

**DOI:** 10.3390/ijerph18094946

**Published:** 2021-05-06

**Authors:** Chanhee Lee, Catherine Apio, Taesung Park

**Affiliations:** 1 Interdisciplinary Program in Bioinformatics, Seoul National University, Seoul 08826, Korea; lch951022@snu.ac.kr (C.L.); 2019-20240@snu.ac.kr (C.A.); 2 Department of Statistics, Seoul National University, Seoul 08826, Korea

**Keywords:** COVID-19, SARS-CoV-2, asymptomatic, undetected, screening, probabilistic model

## Abstract

Increasing evidence shows that many infections of COVID-19 are asymptomatic, becoming a global challenge, since asymptomatic infections have the same infectivity as symptomatic infections. We developed a probabilistic model for estimating the proportion of undetected asymptomatic COVID-19 patients in the country. We considered two scenarios: one is conservative and the other is nonconservative. By combining the above two scenarios, we gave an interval estimation of 0.0001–0.0027 and in terms of the population, 5200–139,900 is the number of undetected asymptomatic cases in South Korea as of 2 February 2021. In addition, we provide estimates for total cases of COVID-19 in South Korea. Combination of undetected asymptomatic cases and undetected symptomatic cases to the number of confirmed cases (78,844 cases on 2 February 2021) shows that 0.17–0.42% (89,244–218,744) of the population have COVID-19. In conclusion, to control and understand the true ongoing reality of the pandemic, it is of outermost importance to focus on the ratio of undetected asymptomatic cases in the total population.

## 1. Introduction

The coronavirus disease 2019 COVID-19 pandemic represents the biggest global shock in decades affecting all major life aspects [1,2]. COVID-19 is an infectious disease caused by novel severe acute respiratory syndrome coronavirus 2 (SARS-CoV-2), which is known to have originated from the city of Wuhan, China in December 2019 [3]. COVID-19 was initially divided into four types: mild, moderate, severe, and critical cases [4]. However, with the global outbreak of coronavirus, there is increasing evidence that many infections of COVID-19 are asymptomatic, but can transmit the virus to others. Asymptomatic infections refer to the positive detection of nucleic acid of SARS-CoV-2 in patient samples by reverse transcriptase-polymerase chain reaction (RT-PCR) but have no typical clinical symptoms or signs and no apparent abnormalities in images, including lung computed tomography (CT) [5,6]. 

Asymptomatic infections have the same infectivity as symptomatic infections [7]. Therefore, early detection of an infected person and cutting off the route of transmission are the key points to controlling the spread of COVID-19 (test-trace-isolate strategy). However, most asymptomatic patients do not seek medical assistance due to no obvious clinical signs and poor prevention awareness, which has contributed to the rapid spread of COVID-19. This has then become a great challenge in the prevention and control of this specific type of patients globally, which has gained more worldwide attention.

South Korea saw its first imported case of COVID-19 on 20 January 2020 [8] and a sharp increase in the number of COVID-19 cases was observed since then with most infections being reported from specific clusters [9]. Outbreaks of COVID-19 related to mass gathering, religious activities, workplaces, and hospitals have accounted for the largest portion of cases in the national outbreak [10]. As of 2 February 2021, there was a total of 78,844 confirmed cases and 1435 deaths in the nation, according to the Korea Centre for Disease Control and Prevention [11]. In addition, Korea’s three antibody titre results confirms increase in the proportion of undetected cases among the general populations (0.03%, 0.07%, and 0.07% in the 1st, 2nd and 3rd survey respectively especially among the younger generation with a 0.22% in the 3rd survey) [12,13]. 

For the test-trace-isolate strategy to accurately work, it is therefore important that true infected populations are estimated correctly. The rate of underestimation also differs from country to country according to its testing policies or prevalence of asymptomatic COVID-19 cases in their population. Making political decision such as lockdown or maintaining social distancing polices without consideration of undetected asymptomatic cases in the population may undermine the proper management of COVID-19 epidemic. The objective of this study is to develop a probabilistic framework for estimating undetected asymptomatic patients. 

Several attempts to solve this problem have been made. The conventional way of estimating asymptomatic ratio of a disease is done using seroprevalence data. However, the collection of these data requires significant logistical effort, time, and cost [14]. Method of estimating the asymptomatic ratio by using Bayes theorem was proposed by using information on Japanese who were evacuated from Wuhan, China on charter flights. This approach is intuitive and effective for robust estimation but had a small sample size (*n* = 565), and the estimates relied on samples from Japanese evacuees from Wuhan. By the simplicity of the model, it was hard to use the probabilistic model directly to other situations in different countries [14]. There have been other methods that model the dynamics of the COVID-19 disease and estimate undetected cases. One is ordinary differential equation ODE based modelling which was done by dividing the population into different categories. [15,16]. Methods that use machine learning based estimation were also proposed [17]. These two approaches rely on the assumptions made by the researcher and therefore the results are dependent on the model and often making it complicated to understand.

Instead of employing ODE based modelling approaches, we developed a probabilistic model that is easy to understand and more robust to the above assumptions made in estimating undetected asymptomatic COVID-19 patients. This probabilistic model is a general framework for estimating undetected asymptomatic patients and can be used in a wide range of settings regardless of the specific situation each country is facing right now. 

## 2. Methods

### 2.1. Data

To achieve the above objective, we used the publicly available data from Ministry of Health and Welfare (MOHW) of South Korea [18]. MOHW through daily official briefings, provides updates on the number of confirmed cases, tests performed, positivity rate (positive tests/total tests), and other important data of COVID-19 in South Korea. In South Korea, three types of screenings (recommended or mandatory, voluntary, and random sampling) have been performed.

Firstly, results from regular screening centers are available. This is mandatory or recommended screenings for individuals who show symptoms related to COVID-19 or have epidemiological associations with infected persons. Secondly, results from temporary screening centers are available. These are voluntary screenings for anyone who is willing to be tested for free. From 14 December 2020, a total of 201 temporary screening centers began testing for COVID-19 for managing the third wave (peak) in Korea. After one month of operation of temporary screening centers (from 14 December 2020 to 13 January 2021), a total of 111,5478 cases were examined, and 3301 (0.3%) patients were found early. Lastly, in Pohang city, South Korea, random sampling for each household over the whole population was performed. On 25 January 2021, the mayor of Pohang, a small urban city located at the southern part of South Korea with a population of 502,736, issued an executive order requiring more than one person per household to undergo diagnostic tests as the spread of coronavirus infection continued. From January 26 to February 4, a total of 196,410 people were examined and 43 confirmed cases of COVID-19 were discovered, giving a positivity rate of 0.02%. Out of 43 confirmed cases, 33 cases were asymptomatic. MOHW reports an aggregated result which consists of different screening results (mandatory + voluntary + Pohang); however, to carry out the estimation procedure effectively, we not only use the aggregated screening results from MOHW but also utilize the two specific screening results, from temporary screening centers and Pohang city, for estimating probabilities for the unscreened group.

### 2.2. Statistical Analysis

**Figure 1:**
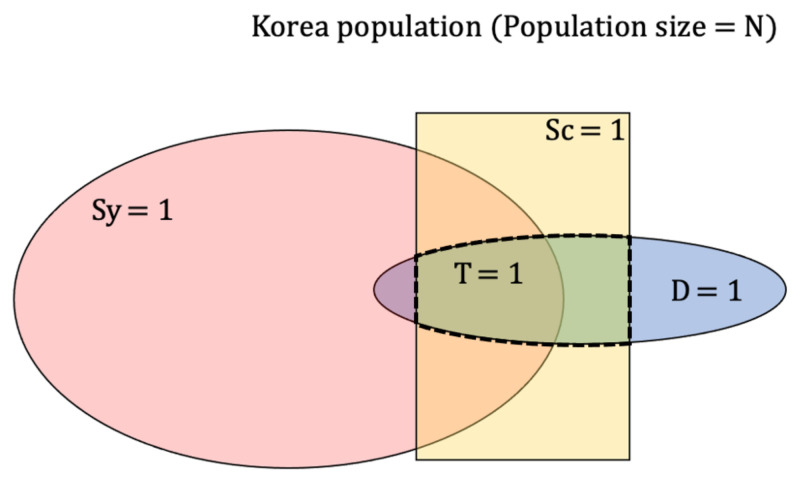
Representing four random variables Disease (D), Symptoms (Sy), Screening (Sc), Test result (T) as a diagram when PPV = 1 and NPV = 1. T is defined in a population where Sc = 1 and D = 1.

Four random variables are defined as follows; D = {0,1}, denoting the infection status of a person, where 1 represents infected and 0 otherwise; Sy = {0,1}, denoting symptoms status of COVID-19, where 1 represents symptoms present and 0 otherwise; Sc = {0,1}, denoting screening status for COVID-19, where 1 represents screening performed and 0 otherwise; and finally, T = {0,1}, denoting results status from screening, where 1 represents results positive and 0 negative. Relationship and diagram of the random variables defined are represented in Figure 1. One thing to note is

(1) $$\left\{T=0\right\}\cup \left\{T=1\right\}=\left\{\mathrm{Sc}=1\right\}$$

By defining four different random variables, we can formulate probabilities regarding the random variables above from the joint distribution P (D, Sy, Sc, T). Our interest is to estimate P (D = 1, Sy = 0, Sc = 0). This is the probability of an individual being infected with COVID-19 but not showing any symptoms and not screened. We restricted our analysis to South Korea’s population and collected data until 2 February 2021. The objective of the research is the estimation of undetected asymptomatic cases of COVID-19 in South Korea as of 2 February 2021. In addition, two assumptions were made in the calculations, which are Positive Predicted Value (PPV) being 1 and Negative Predicted value (NPV) being 1. These assumptions are made to simplify computations in the estimation procedures.

To estimate P (D = 1, Sy = 0, Sc = 0) effectively, we factorized it into known probabilities using chain rule of probability. The result is given as follows.

$$P\left(D=1,\mathrm{Sy}=0,\mathrm{Sc}=0\right)=\frac{\delta_{2}}{\delta_{1}}*\frac{P\left(\mathrm{Sc}=0\right)}{P\left(\mathrm{Sc}=1\right)}*P(\mathrm{Sy}=0|T=1)P\left(T=1\right),$$

where, $\delta_{1}=\frac{P(D=1\;|\;\mathrm{Sc}=1)\;}{P(D=1\;|\;\mathrm{Sc}=0)}$ and $\delta_{2}=\frac{P(\mathrm{Sy}=0|\;D=1,\;\mathrm{Sc}=0)}{P(\mathrm{Sy}=0\;|\;D=1,\;\mathrm{Sc}=1)}$.

$\delta_{1}$ is the ratio of the prevalence of COVID-19 patients from the screened population to that from the unscreened population while $\delta_{2}$ is the ratio of asymptomatic patients among the undetected to that among the detected (screened) COVID-19 patients. PPV = 1 and NPV = 1 are assumed in the derivation of $\delta_{1}=\frac{P(D=1\;|\;\mathrm{Sc}=1)\;}{P(D=1\;|\;\mathrm{Sc}=0)},\;\delta_{2}=\frac{P(\mathrm{Sy}=0|\;D=1,\;\mathrm{Sc}=0)}{P(\mathrm{Sy}=0\;|\;D=1,\;\mathrm{Sc}=1)}$. Detailed derivation of $\delta_{1}$ and $\delta_{2}$ in $P\left(D=1,\;\mathrm{Sy}=0,\;\mathrm{Sc}=0\right)=\frac{\delta_{2}}{\delta_{1}}*\frac{P\left(\mathrm{Sc}=0\right)}{P\left(\mathrm{Sc}=1\right)}*P(\mathrm{Sy}=0|\;T\;=1)P\left(T=1\right)$, and factorizations of other probabilities in the joint distribution are in the supplementary material. Table 1 shows all the factorization results in the joint distribution.

Using the MOHW data, we can estimate $\frac{P\left(\mathrm{Sc}=0\right)}{P\left(\mathrm{Sc}=1\right)}*P(\mathrm{Sy}=0|\;T=1)P\left(T=1\right)$ and have partial information of $\delta_{1}=\frac{P(D=1\;|\;\mathrm{Sc}=1)\;}{P(D=1\;|\;\mathrm{Sc}=0)}$ and $\delta_{2}=\frac{P(\mathrm{Sy}=0|\;D=1,\mathrm{Sc}=0)}{P(\mathrm{Sy}=0|D=1,\mathrm{Sc}=1)}$. Using the joint distribution in Table 1, it can be easily shown that $P\left(\mathrm{Sy}=0\;|\;D=1,\mathrm{Sc}=1\right)=P(\mathrm{Sy}=0|T=1)$; therefore, denominator of $\delta_{2}$ is equivalent to P(Sy=0|T=1) and we can use the estimates from MOHW which is 0.4.

By the fact that the denominator of $\delta_{2}$ is estimated as 0.4, we can constrain $\delta_{2}$ to be in the range of $1\leq \delta_{2}\leq 2.5$. This is because we assume P $(\mathrm{Sy}=0|\;D=1,\;\mathrm{Sc}=0)\;>\;P\left(\mathrm{Sy}=0|D=1,\mathrm{Sc}=1\right)$. It is logical to assume people that are infected but not tested will have a higher chance of being asymptomatic than people that are infected and tested; therefore, $1\leq \delta_{2}$. Since the numerator of $\delta_{2}$ is also a probability, the maximum value of $\delta_{2}$ is 2.5 when the numerator P(Sy=0| D=1, Sc=0)=1. This is an extreme case where all infected, yet tested population are asymptomatic.

$\delta_{1}$ is $\frac{P(D=1\;|\;\mathrm{Sc}=1)\;}{P(D=1\;|\;\mathrm{Sc}=0)}$, so we can interpret this value as ratio of infection rate in tested population to that of untested population. We assume that P(D=1|Sc=0)≤ P(D=1|Sc=1) is reasonable, since the population that is screened is more likely to have higher infection rate. $\delta_{1}$ can be influenced by testing policies of each countries. Countries that have higher requirements on COVID-19 testing, such as letting only those who show severe symptoms or who had close contact with an infected person be screened, will have higher $\delta_{1}$, while other countries that allow more people to take COVID-19 screening without many requirements will have lower value of $\delta_{1}$. Therefore,$\;1\leq \delta_{1}$ and $\delta_{1}$ = 1 is a special case where screened population and unscreened population have same infection rate.

To summarize our methodology, our aim is to estimate P (D = 1, Sy = 0, Sc = 0) using probabilistic framework. To use the data and assumptions we made, we performed chain rule of probability and factorized $P\left(D=1,\;\mathrm{Sy}=0,\;\mathrm{Sc}=0\right)$ to $\frac{\delta_{2}}{\delta_{1}}*\frac{P\left(\mathrm{Sc}=0\right)}{P\left(\mathrm{Sc}=1\right)}\;*\;P(\mathrm{Sy}=0|\;T=1)\;*\;P\left(T=1\right)$. All quantities are known except for $\delta_{1},\;\delta_{2}$, and $\delta_{2}$ is well restricted in the range of $1\leq \delta_{2}\leq 2.5$, while $1\leq \delta_{1}$. Using some additional data collected recently, we will estimate $\delta_{1}$ and present the estimated range of P (D = 1, Sy = 0, Sc = 0) in the result section using sensitivity analysis [19].

## 3. Results

In the result section, we first organize important probabilities from the MOHW data in Table 2. These estimates from MOHW data are used in the estimation of $P\left(D=1,\;\mathrm{Sy}=0,\;\mathrm{Sc}=0\right)=\frac{\delta_{2}}{\delta_{1}}\;*\;\frac{P\left(\mathrm{Sc}=0\right)}{P\left(\mathrm{Sc}=1\right)}*P(\mathrm{Sy}=0|\;T=1)P\left(T=1\right)$.

We present two estimation scenarios for estimating $\delta_{1}$, using the voluntary screening results from temporary screening centers and random sampling screening results from the city of Pohang. Finally, putting these results together, we will provide an interval estimate, for estimating proportion of undetected asymptomatic cases in South Korea on 2 February 2021.

### 3.1. First Scenario: Inference from Voluntary Screenings in Temporary Screening Centers

From 14 December 2020, 201 temporary screening centers were opened nationwide in South Korea, enabling anyone to be tested for COVID-19. After one month of operation, the Korean government reported that a total of 1,115,478 cases were examined and 3301 (0.3%) patients were found early. We use this result to estimate P(D=1 | Sc=0) as 0.0030. Using this estimate, $\delta_{1}$ is estimated to be 4.67, and accounting for $\delta_{2}$ we have an interval estimate of 0.0011–0.0027. In terms of population, the number of undetected asymptomatic patients are estimated to be 57,000–139,900.

We consider this scenario as the worst case for South Korea, because even though temporary screening centers allow anyone to be tested, it is more likely that people who suspect they may have been infected with COVID-19 went on to get tested at the temporary centers. Therefore, those populations that were tested in the temporary screening centers do not represent true {Sc = 0} and estimates of P (D = 1 | Sc = 0) in the first scenario are expected to be somewhere between true P (D = 1 | Sc = 0) and true P (D = 1 | Sc = 1). Next, we will look at second scenario that represent true {Sc = 0} better than first scenario.

### 3.2. Second Scenario: Inference from Random Sampling of Each Household from Total Population in Pohang, South Korea

On 25 January 2021, city of Pohang issued an executive order requiring more than one person to undergo diagnostic tests for every household. By this order, a total of 196,410 people were examined and 43 confirmed cases of COVID-19 (0.02%), while the total population of Pohang is reported to be 502,736 by January 2021. Using this estimate, $\delta_{1}$ is estimated to be 70, and accounting for $\delta_{2}$ we have an interval estimate of 0.0001–0.0002. In terms of population, the number of undetected asymptomatic patients is estimated to be 5200–10,400. Instead of giving range to $\delta_{2}$, we can estimate $\delta_{2}$ as a point estimate using the data from the 43 confirmed cases where 33 cases were asymptomatic patients. We will use this information to estimate P(Sy=0| D=1, Sc=0), resulting $\delta_{2}$ to be estimated as 1.88. Combining $\delta_{1}$, $\delta_{2}$ we have point estimate of rate of undetected asymptomatic cases in total population as 0.00013, in terms of population 6,900, which lies mid-way between the above interval estimate.

This estimate is lower than that that from the first scenario since $\delta_{1}$ changed from 4.67 to 70. As expected, the result in temporary screening centers had higher positivity rate than tests held in Pohang, showing those who went on to temporary screening centers were more likely to be infected than those who were tested randomly from each household from total population. Estimating P (D = 1 | Sc = 0) with only the result from Pohang can be biased because it is not accurate to assume P (D = 1 | Sc = 0) in total population of Korea to be the same as P (D = 1 | Sc = 0) from the city of Pohang.

### 3.3. Estimation of the Proportion of Undetected Asymptomatic Cases

**Figure 2:**
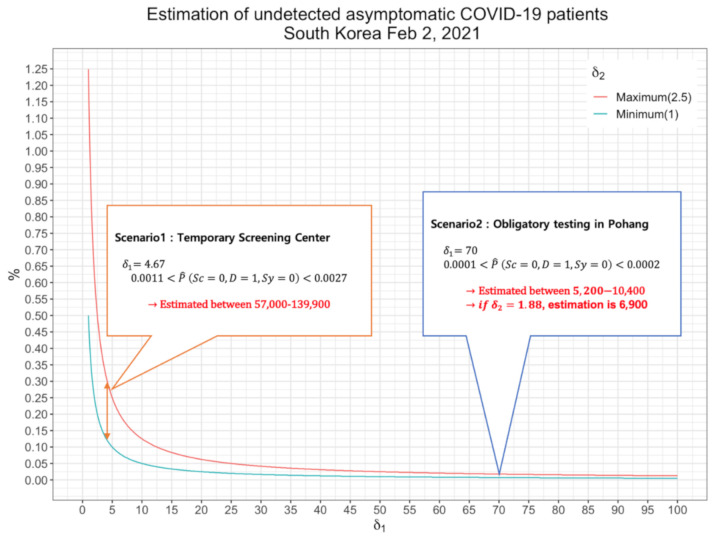
Estimation of undetected asymptomatic COVID-19 cases in South Korea, 2 February 2021 for two different scenarios.

In the process of deriving the interval estimates from the two scenarios, we performed sensitivity analysis by varying $\delta_{2}$ values from 1 to 2.5, which we showed in the methods section. In the above two scenarios, the former scenario provides nonconservative (0.0011–0.0027) and the latter conservative (0.0001–0.0002) estimates of the range of asymptomatic patients and their estimates can be combined to provide a more encompassing range of asymptomatic patients which takes into consideration random screening and voluntary screening. For our interval estimates to be robust, it was therefore reasonable to choose the lower bound value as the smallest value among the lower bounds of the two scenarios and the upper bound as the highest value from the upper bounds of the two scenarios. We set the lower bound of our estimate as 0.0001, minimum value from second scenario, and upper bound as 0.0027, maximum value from the first scenario. Our final estimate for the proportion of undetected asymptomatic cases of COVID-19 in South Korea as of 2 February 2021 is 0.0001–0.0027 and in terms of the population, 5200–139,900. We included the first scenario so that the true proportion of undetected asymptomatic cases is not underestimated. Figure 2 summarizes estimation result for the two scenarios.

### 3.4. Estimation of Total COVID-19 Patients in South Korea

Along with the inference of the proportion of undetected asymptomatic patients, it is also possible to estimate the total number of COVID-19 patients in the population (Total = undetected cases + detected cases (confirmed)). To estimate undetected cases, which encompasses both undetected symptomatic cases and undetected asymptomatic cases, we need an estimate for undetected symptomatic cases, also. This can be done in a similar way of estimating undetected asymptomatic cases by using the result of P (D = 1, Sy = 1, Sc = 0) from Table 1. Summing up the estimates of undetected symptomatic cases and undetected asymptomatic cases, we estimate undetected yet infected cases to be 10,400–139,900, which are the upper bound values from the conservative and nonconservative scenarios. This is because as $\delta_{2}$ changes from 1.0 to 2.5, the proportion of undetected asymptomatic cases changes from 40% to 100% leaving the remaining proportion for symptomatic cases. As of 2 February 2021, there were a total of 78,844 confirmed cases in the nation, according to the Korea Centre for Disease Control and Prevention [11]. Therefore, adding our estimates to the confirmed cases, the total number of COVID-19 patients in South Korea is estimated to be 89,244–218,744 people in the population that have COVID-19. This result encompasses both worst case and conservative scenarios of the pandemic situation of Korea.

## 4. Discussion

After the first confirmed case of coronavirus appeared in Korea on 20 January 2020, many strategies and efforts were undertaken to stop the spread of the disease. By combining testing, contact tracing, early isolation, and the free treatment of positive cases, two days per week transparent press briefings on COVID-19, voluntary engagement of citizens and businesses, combined with digital technologies without taking to “lockdown” measures, South Korea has been able to contain pandemic situation considerably when compared to other countries, especially in 2020.

However, with the ban on gatherings of more than five persons as part of the government’s special antivirus measures, still 300 to 600 new cases were being reported daily in February 2021. One can attribute this to the undetected asymptomatic patients that spread the disease silently since asymptomatic infections have the same infectivity as symptomatic infections. To control and understand the true ongoing reality of the pandemic, it is therefore of importance to focus on the ratio of undetected asymptomatic cases in total population.

Our research findings show that as $\delta_{1}$ increases, estimates for undetected asymptomatic cases change inverse proportionally. This shows that even though two countries might have a similar data of confirmed cases, which is what we usually observe from outside, the ratio of undetected asymptomatic cases could be very different according to $\delta_{1}\left(=\frac{P(D=1\;|\;\mathrm{Sc}=1)\;}{P(D=1\;|\;\mathrm{Sc}=0)}\right)$. $\delta_{1}$ can vary according to country’s criteria on screening and knowing this quantity can explain how the pandemic is really happening inside each country. One way of estimating $\delta_{1}$ is by conducting two-way diagnostic tests. Main diagnostic testing should focus on the population that is more prone to be infected, which reduces unnecessary testing costs and resources and used to make an estimate for P(D=1 | Sc=1). The other test should be designed to target the populations {Sc = 0}, which are not suitable for main diagnostic testing, and it should be used to make an estimate for P(D=1 | Sc=0). In South Korea, two tests (voluntary and Pohang) that are different from the regular screening centers were performed which made the estimation of P(D=1 | Sc=0) possible.

One can argue that our estimate of P(D=1 | Sc=0) using data from Pohang can be biased as it may not represent the whole COVID-19 situation of the country. That is, there is no evidence that P(D=1 | Sc=0) = P(D=1 | Sc=0, Pohang) holds for the whole country. It would be best to use the data from obligatory random sample test result nationwide, for estimation of P(D=1 | Sc=0); however, this is the best estimate that can be made with current available data. Secondly, for calculation simplicity we made two assumptions, PPV = 1 and NPV = 1. This is unlikely to be true in real world testing; therefore, relieving these assumptions could be the next step in making better estimates.

In the future, we hope to find a way of estimating P(D=1 | Sc=0) without resorting to two-way tests, which can be done by understanding the relationship between P(D=1 | Sc=0), P(D=1 | Sc=1) and screening policy of a country. Since not all countries are performing two-way diagnostic test as South Korea does, it is difficult for our probabilistic model to be expanded internationally directly. However, provided a range of values $\delta_{1}$ and $\delta_{2}$ that reflect if we find a way to estimate P (D = 1 | Sc = 0) using P (D = 1 | Sc = 1) and screening policy of a country, our model would be expanded to serve those other regions in the world. Finding an exact relationship between those quantities could be difficult; therefore, data-driven machine learning technique could be utilized in the estimation of P (D = 1 | Sc = 0) using other data that are relevant for estimating. There have been many studies related to asymptomatic cases worldwide and variations in screening policies and genetics among different countries could be useful data for estimating P (D = 1 | Sc = 0) [20,21]. Lastly, in the process of this analysis, we discovered a Bayesian approach for the estimation of asymptomatic patients and hope to include this approach in our future analysis to expand our probabilistic model into more sophisticated statistical frameworks [22].

## 5. Conclusions

From this study, we developed a general probabilistic model for estimating the proportion of undetected asymptomatic patients among the population in South Korea. We devised two scenarios for the estimation using real-world data from temporary screening centers nationwide and the random sampling tests held in Pohang. By combining these two real world scenarios, we gave an interval estimation of 5200–139,900 to be the number of undetected asymptomatic cases in South Korea as of 2 February 2021. We also gave an interval estimation of total COVID-19 patients (undetected + detected cases) in South Korea to be 89,244–218,744. We observe that the estimated total number of confirmed cases is higher than the current number of confirmed cases (78,844). These results show the role of asymptomatic cases in the spread of COVID-19 which emphasizes the importance of considering these asymptomatic cases in any prevention plans designed to curb the spread of COVID-19.

## Figures and Tables

**Table 1:** Derivation of the joint probability distribution P (D, Sy, Sc, T) using chain rule of probability.

Disease	Symptoms	Screening	Test Result	Factorized Probabilities
1	1	1	1	$P\left(\mathrm{Sy}=1|T=1\right)*\;P\left(T=1\right)$
1	1	1	0	0
1	1	0	-	$\frac{\delta_{2}}{\delta_{1}}*\frac{P\left(\mathrm{Sc}=0\right)}{P\left(\mathrm{Sc}=1\right)}*P\left(\mathrm{Sy}=1|T=1\right)*\;P\left(T=1\right)\;$
1	0	1	1	$P\left(\mathrm{Sy}=0|T=1\right)*\;P\left(T=1\right)$
1	0	1	0	0
1	0	0	-	$\frac{\delta_{2}}{\delta_{1}}*\frac{P\left(\mathrm{Sc}=0\right)}{P\left(\mathrm{Sc}=1\right)}*P\left(\mathrm{Sy}=1|T=0\right)*\;P\left(T=0\right)\;$
0	1	1	1	0
0	1	1	0	$P\left(\mathrm{Sy}=1|T=0\right)*\;P\left(T=0\right)$
0	1	0	-	$\frac{\delta_{2}}{\delta_{1}}*\frac{P\left(\mathrm{Sc}=0\right)}{P\left(\mathrm{Sc}=1\right)}*P\left(\mathrm{Sy}=0|T=0\right)*\;P\left(T=1\right)\;$
0	0	1	1	0
0	0	1	0	$P\left(\mathrm{Sy}=0|T=0\right)*\;P\left(T=0\right)$
0	0	0	-	$\frac{\delta_{2}}{\delta_{1}}*\frac{P\left(\mathrm{Sc}=0\right)}{P\left(\mathrm{Sc}=1\right)}*P\left(\mathrm{Sy}=0|T=0\right)*\;P\left(T=0\right)\;$

**Table 2:** Estimates of probabilities using data from Ministry of Health and Welfare of South Korea (MOHW) until 2 February 2021.

Probability	Explanation	Estimates from MOHW Data
P (Sc = 0)	proportion of unscreened persons in the total population	0.8923
P (Sc = 1)	proportion of screened persons in the total population	0.1077
P (T = 0| Sc = 1)	proportion of negative test result given that person is screened	0.9860
P (T = 1 | Sc = 1)	proportion of positive test result given that person is screened	0.0140
P (Sy = 0 | T = 1)	proportion of symptomatic given that person is positive	0.4000
P (Sy = 1 | T = 1)	proportion of asymptomatic given that person is positive	0.6000
P (T = 0)	$P\left(T=0\right)=\;P\left(T=0,\;\mathrm{Sc}=1\right)=P\left(\mathrm{Sc}=1\right)*P(T=0\;|\;\mathrm{Sc}=1)\;$	0.1062
P (T = 1)	$P\left(T=1\right)=\;P\left(T=1,\;\mathrm{Sc}=1\right)=P\left(\mathrm{Sc}\;=1\right)*P(T=1\;|\;\mathrm{Sc}=1)$	0.0015

## Data Availability

Data available in a publicly accessible links provided in the reference [11,18].

## Supplementary Materials

The following are available online at https://www.mdpi.com/article/10.3390/ijerph18094946/s1, Detailed derivation of factorized probabilities from the joint distribution P (D, Sy, Sc, T).

## References

[1] Monshi M.M.A., Poon J., Chung V. (2020). Deep learning in generating radiology reports: A survey. Artif. Intell. Med..

[2] Richardson S., Hirsch J.S., Narasimhan M., Crawford J.M., McGinn T., Davidson K.W., Barnaby D.P., Becker L.B., Chelico J.D., Cohen S.L. (2020). Presenting characteristics, comorbidities, and outcomes among 5700 patients hospitalized with COVID-19 in the New York City area. JAMA.

[3] Susilo A., Rumende C.M., Pitoyo C.W., Santoso W.D., Yulianti M., Herikurniawan H., Sinto R., Singh G., Nainggolan L., Nelwan E.J. (2020). Coronavirus disease 2019: Tinjauan literatur terkini. J. Penyakit Dalam Indones..

[4] (2020). Diagnosis and treatment plan of Corona Virus Disease 2019 (tentative sixth edition). Glob. Health J..

[5] WHO (2020). Laboratory Testing for 2019 Novel Coronavirus (2019-nCoV) in Suspected Human Cases: Interim Guidance, 14 January 2020.

[6] Gao Z., Xu Y., Sun C., Wang X., Guo Y., Qiu S., Ma K. (2021). A Systematic Review of Asymptomatic Infections with COVID-19. J. Microbiol. Immunol. Infect..

[7] Chen Y., Wang A., Yi B., Ding K.Q., Wang H.B., Wamg J.M., Shi H.B., Wang S.J., Xu G.Z. (2020). The epidemiological characteristics of infection in close contacts of COVID-19 in Ningbo city. Chin. J. Epidemiol..

[8] Shim E., Tariq A., Choi W., Lee Y., Chowell G. (2020). Transmission potential and severity of COVID-19 in South Korea. Int. J. Infect. Dis..

[9] Park Y.J., Choe Y.J., Park O., Park S.Y., Kim Y.-M., Kim J., Kweon S., Woo Y., Gwack J., Kim S.S. (2020). COVID-19 National Emergency Response Center, Epidemiology and Case Management Team. Contact tracing during coronavirus disease outbreak, South Korea, 2020. Emerg. Infect. Dis..

[10] Park S.Y., Kim Y.-M., Yi S., Lee S., Na B.-J., Kim C.B., Kim J.-I., Kim H.S., Park Y., Huh I.S. (2020). Coronavirus disease outbreak in call center, South Korea. Emerg. Infect. Dis..

[11] Korea Disease Control and Prevention Agency. http://www.kdca.go.kr/.

[12] Apio C., Kamruzzaman M., Park T. (2020). Confidence intervals for the COVID-19 neutralizing antibody retention rate in the Korean population. Genom. Informatics.

[13] Kamruzzaman M., Apio C., Park T. (2020). Updated confidence intervals for the COVID-19 antibody retention rate in the Korean population. Genom. Informatics.

[14] Nishiura H., Kobayashi T., Miyama T., Suzuki A., Jung S.-M., Hayashi K., Kinoshita R., Yang Y., Yuan B., Akhmetzhanov A.R. (2020). Estimation of the asymptomatic ratio of novel coronavirus infections (COVID-19). Int. J. Infect. Dis..

[15] Mahajan A., Solanki R., Sivadas N. (2021). Estimation of undetected symptomatic and asymptomatic cases of COVID-19 infection and prediction of its spread in the USA. J. Med Virol..

[16] Liu Z., Magal P., Webb G. (2021). Predicting the number of reported and unreported cases for the COVID-19 epidemics in China, South Korea, Italy, France, Germany and United Kingdom. J. Theor. Biol..

[17] Vaid S., Cakan C., Bhandari M. (2020). Using machine learning to estimate unobserved COVID-19 infections in North America. J. Bone Jt. Surg. Am. Vol..

[18] Ministry of Health and Welfare of South Korea. http://ncov.mohw.go.kr/.

[19] Saltelli A. (2002). Sensitivity analysis for importance assessment. Risk Anal..

[20] Napoli P.E., Nioi M. (2020). Global Spread of Coronavirus Disease 2019 and Malaria: An Epidemiological Paradox in the Early Stage of A Pandemic. J. Clin. Med..

[21] Nioi M., Napoli P.E., Fossarello M., D’Aloja E. (2020). Autopsies and Asymptomatic Patients During the COVID-19 Pandemic: Balancing Risk and Reward. Front. Public Heal..

[22] Wu S.L., Mertens A.N., Crider Y.S., Nguyen A., Pokpongkiat N.N., Djajadi S., Seth A., Hsiang M.S., Colford J.M., Reingold A. (2020). Substantial underestimation of SARS-CoV-2 infection in the United States. Nat. Commun..

