# Supplementary material for: Estimation of Undetected Asymptomatic COVID-19 Cases in South Korea Using a Probabilistic Model
Source: Int J Environ Res Public Health. 2021 May 6;18(9):4946. doi: 10.3390/ijerph18094946 (PMC8124955; doi:10.3390/ijerph18094946)
Supplement: Supplementary file 1 [file ijerph-18-04946-s001.zip › 5/ijerph-1173327.pdf]

International Journal of
Environmental Research and Public Health

MDPI

Article

The Importance of Peer Mentoring, Identity Work and Holding Environments: A Study of African American Leadership Development

Audrey J. Murrell [1,*], Stacy Blake-Beard [2] and David M. Porter, Jr. [3,†]

1 Katz School of Business, University of Pittsburgh, Pittsburgh, PA 15260, USA
2 Tuck School of Business, Dartmouth College, Hanover, NH 03755, USA; stacy.blakebeard@tuck.dartmouth.edu
3 Federal Reserve Bank of San Francisco, San Francisco, CA 94105, USA; david.porter@sf.frb.org
* Correspondence: amurrell@pitt.edu
† Opinions and conclusions herein are those of the authors and do not necessarily represent the views of the Federal Reserve System and the Federal Reserve Bank of San Francisco.

Abstract: Mentoring is well-known for its positive impact on diversity and inclusion across a wide variety of organizational contexts. Despite these demonstrated advantages, efforts to develop diverse leaders either through access to informal mentoring relationships or via formal mentoring programs are often complex, expensive, and frequently produce mixed results. We examine the unique impact of peer mentoring to support and develop African American leaders using a formalized program approach. Our findings show that peer mentoring is effective in providing a safe environment for the necessary work of identity to take place among African American leaders. This identity work takes the form of holding behaviors such as enabling perspectives, empathic acknowledgement and containment that are critical for the development, support and validation of diverse leaders. Our findings clearly show the benefit of external identity peer mentors for providing support and validation for African American leaders that can be absent within traditional hierarchical mentoring. By examining the outcomes of an actual leadership development program over time, we provide recommendations on how to enhance diverse leadership development by recognizing and cultivating the positive impact of identity-based peer mentoring.

Keywords: peer mentoring; diversity; leadership; social identity

Citation: Murrell, A.J.; Blake-Beard, S.; Porter, D.M., Jr. The Importance of Peer Mentoring, Identity Work and Holding Environments: A Study of African American Leadership Development. *Int. J. Environ. Res. Public Health* **2021**, *18*, 4920. https://doi.org/10.3390/ijerph18094920

Academic Editor: Paul B. Tchounwou

Received: 22 March 2021
Accepted: 27 April 2021
Published: 5 May 2021

Publisher's Note: MDPI stays neutral with regard to jurisdictional claims in published maps and institutional affiliations.

Copyright: © 2021 by the authors. Licensee MDPI, Basel, Switzerland. This article is an open access article distributed under the terms and conditions of the Creative Com-mons Attribution (CC BY) license (http://creativecommons.org/licenses/by/4.0/).

1. Introduction

Mentoring is well-known for its positive impact on diversity and inclusion across a wide variety of organizational contexts [1]. It is clear within this extensive body of work that mentoring offers both psychosocial and instrumental benefits for individuals and organizations [2]. The positive impact of mentoring for leadership development has also been well-documented [3]. Despite these demonstrated advantages, efforts to develop diverse leaders either through access to informal mentoring relationships or via formal mentoring programs are often complex, expensive, and frequently produce mixed outcomes or results [4]. Thus, better understanding how to effectively develop diverse leaders from an evidence-based approach is a clear need for both research and organizational practice. Developing diverse leaders also means paying close attention to providing a psychologically safe work environment, one in which diverse individuals feel safe to voice ideas, seek feedback, provide honest feedback and task risks as they develop their leadership competence and confidence [5].

It is the case that effective leadership development programs, especially for diverse leaders, can face unique challenges in cultivating positive relationships and often fail to value or include different forms of leadership identities [6,7]. Diverse leaders can

experience challenges to having their identities validated and respected by others who view them as different from or in conflict with traditional definitions and expectations of leadership based on the organization's history, its culture and societal norms [8]. These circumstances highlight the question of how organizations can leverage the well-documented power of mentoring relationships in order to effectively cultivate and support leadership development that advances diversity and inclusion efforts. Thus, we explore the intersection of peer mentoring and identity-work through an evidence-based leadership development program for African Americans mid-career managers. We examine the unique impact of peer mentoring to support and develop African American leaders using a formalized program approach. By analyzing qualitative outcome data collected across this year-long formal program, we explore the impact of peer mentoring as a buffer for the necessary work of racial identity and the cultivation of holding environments that we argue are essential for developing diverse leaders. Thus, we examine three key research questions. First, what evidence is there that peer mentoring provided within a formal program can provide support in the form of holding environments for African American leaders? Second, what is the unique impact of external peer mentors who share racial identity group membership in providing holding environments for developing African American leaders? Third, what recommendations can we provide on how organizations can enhance diverse leadership development efforts by recognizing and cultivating the positive impact of identity-based peer mentoring?

1.1. Mentoring and Diversity

Mentoring has traditionally been defined as a series of relationships that are dynamic, reciprocal and have a dual impact on both the mentor and the mentee [9]. Based on previous research, mentoring serves two essential functions—career and psychosocial [10]. Career functions are those aspects of mentoring that enhance learning the ropes and preparing the individual for advancement within an organization [11]. Career functions include activities such as sponsorship, exposure and visibility, coaching, protection, and challenging assignments [12]. Psychosocial functions are those aspects of the relationship that enhance a sense of competence, clarity of identity, and effectiveness in a professional role. Psychosocial functions include activities such as role modeling, acceptance and confirmation, counseling, and friendship [13]. The connection between mentoring and positive identities at work has been previously discussed across both career and psychosocial functions [14]. Mentoring has been positioned as a powerful tool to enable the careers of those advancing through the ranks in organizations [15-18]. Additionally, mentoring has been linked to several positive outcomes, such as improving both mentor and protégé job performance, helping to socialize protégés, supporting long-range human resource planning, and promoting the development and support of organizational leaders [1,12-14]. Thus, gaining a better understanding of the key role that identity and identity work play is an important contribution to existing research and practice on mentoring and leadership development.

Mentoring has gained increasing attention as a powerful tool to enable the careers of individuals as they advance through the ranks in organizations [1,4,15,16]. Historically, a mentor was generally defined as a more senior or knowledgeable individual who uses his or her influence and experience to help with the advancement of a protégé or mentee [10]. However, recent work has expanded the types of relationships beyond the traditional senior-junior mentoring to include other relationships such as peer mentoring [17-19], virtual mentoring [20], group mentoring [21], and reverse mentoring [22]. Across these examinations, the outcomes of various mentoring relationships are clear. Those with access to mentoring have been consistently shown to benefit from their involvement in these relationships; they report higher salaries, increased promotion rates, greater career satisfaction, higher organizational commitment and less intention to leave the organization as well as lower levels of turnover [16,23-26].

Within this work, the various challenges that diverse leaders face when mentors are different in terms of gender, race, and/or other demographic factors are also well documented [27,28]. For example, previous work examined the experience of executives of color who are attempting to navigate the complex environments within traditional and predominantly white corporations [4]. One of the key findings noted as a dimension of success is the leaders' ability to build a foundation of competence, credibility, and confidence throughout their careers. The notion of credibility is essential because it involves both a process of identity transformation and social construction. For both experienced and emergent leaders, the organization's culture and content can either reinforce or detract from how diverse individuals are perceived as a leader in terms of credibility. Those who are seen as a 'good fit' within the organization are also often seen as having strong credibility. Perceptions of credibility are also enhanced by receiving validation from influential mentors and sponsors [29].

Hostile or unsupportive environments not only yield barriers for feelings of psychological safety and inclusion, they also signal (intentionally or unintentionally) a lack of credibility for diverse mentees as future leaders and executives [3]. The key takeaway from this work is that lack of support from mentors and sponsors, driven in part by implicit notions of leadership that are incompatible or conflicting with individuals from backgrounds, can create real barriers for the development and advancement of diverse leaders. The issue of how diverse mentoring relationships can be supported through the various phases of initiation and cultivation to produce high-quality relationships is a necessary yet complex aspect of effective leadership development and deserves additional attention from both research and practice [29].

While diverse mentoring relationships (e.g., cross-race, cross-gender, etc.) within a single organization may have many advantages that are critical for individual and organizational outcomes, the reality is that these relationships are complex, more likely to produce conflict, and may not meet all of the needs for women and people of color within organizations [3,27]. This reality may be particularly challenging for those seeking to "breakthrough" to senior level leadership positions [4,27]. We argue that this lack of necessary support is especially true if mentoring relationships (both formal and informal) do not explicitly address identity issues and engage in identity work as part of the mentoring relationships [30-32]. This assertion is supported by a wide variety of empirical and theoretical work that shows that race (as one aspect of diversity) is embedded within the organizational context, a consistent driver of work attitudes and outcomes and a moderator of the return-on-investment employees received from training and other developmental activities [33-35]. Thus, attention should be devoted to how we cultivate future leaders who will positively impact organizations because they have access to diverse mentoring relationships that include support for the necessary work of identity.

1.2. The Power of Peer Mentoring

Though mentors have traditionally been viewed as senior individuals, Kram and Isabella [18] provided an early examination into peer relationships as another type of mentoring between individuals. They argued that peer relationships can serve the same functions as traditional senior-junior mentoring relationships and yet can be more readily available to individuals, because of both sheer numbers and overall accessibility. Moreover, Kram and Isabella [18] suggest that peer relationships may achieve a greater degree of communication, support, and collaboration than traditional hierarchical mentoring relationships. They examined peers across a variety of career stages and conducted in-depth interviews of a "focal person" along with significant others who were identified during the interviews. Their results supported the notion that peer mentoring provides much of the same range of career and psychosocial support functions as traditional mentoring relationships, including information sharing, career advice, exposure, coaching, and sponsorship, as well as emotional support, feedback, and friendship.

While peer mentors provide career and psychosocial support in a manner comparable to that of traditional senior mentors, important conceptual differences exist between these types of mentorships, often related to the horizontal or vertical nature of the relationship. An important distinction to note for our purposes relates to reciprocity. Traditional mentoring relationships are frequently conceptualized and measured as unidirectional (typically from the perspective of the mentee), whereas peer mentorships are described as either uniquely mutual or reciprocal [19,36]. Both of these notions underscore the unique value provided by reciprocity within peer mentoring. While reciprocity has been noted as important for effective mentoring relationships in general [37,38], it is clearly a unique benefit that takes place within peer mentoring [18,19].

Given the changing nature of organizations in terms of being more networked and flat, peer or lateral mentoring is more readily available within the environment and provides critical career and social support [39,40]. Peer mentoring relationships provide both important job-related and technical knowledge and have been shown to be a valuable resource for knowledge transfer and learning [37,38]. Thus, peer mentoring can be a powerful conduit for the transfer of tacit knowledge into explicit knowledge [41]. This transfer is particularly critical given that much of the knowledge shared between peers is learned from personal experience and thus not typically part of the formal knowledge management processes within the organization [42]. In fact, some argue that peers can actually compensate for an absence of traditional mentors because peer mentoring is less dependent on status, power and access to organizational resources [43].

Clearly there is an important contribution to be made by examining peer mentoring as a source of support and as a tool for developing diverse leaders. First, this examination responds to a number of scholars who call for greater attention to the diverse types of mentoring and their impact on key career and organizational outcomes [14,15]. Second, it draws attention to the importance of reciprocity as a defining feature of peer mentoring that distinguishes it from traditional hierarchical types of mentoring [17,19]. Third, peer or lateral mentoring relationships can meet the needs of the leaders who do not represent the traditional views of leadership or where needs for identity-related connections are important [7]. Examining the importance of peer mentoring for developing diverse leadership would benefit from an evidence-based research approach such that it provides a valid model for future research and organizational practice. Looking at peer mentoring from a relational view can also provide a unique perspective that these relationships are not simply a resource for support or information, they also have an impact on shaping attitudes and behaviors. For example, the idea that peer mentors can act as agents of social influence is supported by several well-known theoretical perspectives that include social learning theory [44], social information processing theory [45] and social comparison theory [46].

Social learning theory emphasizes the importance of observing and modeling the behaviors, attitudes, and emotional reactions of others in learning and personal development. For instance, one study by showed that employees' performance of organizational citizenship behaviors was related to the frequency and consistency of organizational citizenship behavior by other peer employees in their workgroup [47]. Ibarra demonstrated that investment bank and management consulting firm employees who made the transition from entry-level to management positions observed and interacted with peer employees whom they admired in order to learn what behaviors, attitudes and perceptions helped the admired peer employees to be successful [48]. These studies show that social learning can influence the behavior, attitudes and perceptions of peers who share relational ties. Thus, we would expect peer mentoring relationships, particularly those characterized by strong relational ties and some level of reciprocity, to involve social learning about issues concerning the organization and its values and culture. Seeing peer relationships as a valuable source of knowledge and learning challenges traditional one-to-one approaches that often assume the only meaningful learning comes from more senior experts within the organization. For example, Files and her colleagues conducted a pilot

program for the advancement of women in academic medicine and found that peer mentoring facilitated academic productivity, promotion in academic rank and enthusiasm for the profession as critical outcomes [49].

Social information processing also plays a key role in shaping perceptions, attitudes, and behaviors in organizations. The core argument of social information processing theory is that because organizations are complex and ambiguous environments, perceptions are influenced by the social context in which they are formed. This belief is especially relevant for leadership development because leadership competencies are complex, dynamic and contextual in nature [50,51]. This type of influence may occur as a result of direct statements from peers or through intentional or unintentional behavioral cues [45]. According to this theory, social information affects how individuals: (1) learn to react to social cues; (2) form perceptions by focusing attention on some aspects of the work environment but away from others; (3) construct their interpretations of organizational events; and,(4) understand the requirements of their jobs. For example, this understanding is clearly illustrated in what is known as the "preceptor model" that is currently used within training programs for medical and health care professionals [52].

Similarly, Coleman, Katz, and Menzel found that doctors' decisions to prescribe a new drug were similar to the decisions of professional associates with whom they had talked about the drug [53]. Mark and her colleagues reviewed several innovative mentoring programs that each showed evidence of peer influence on recruitment, retention and development of female junior faculty within academic medicine [54]. Other studies have shown that employees' attitudes towards new technology were similar to the attitudes of individuals with whom they frequently communicate [54]. For example, Meyer found that employees had similar perceptions of organizational coordination to employees with whom they interacted with frequently [55]. Similarly, Dabos and Rousseau showed that faculty members' beliefs regarding promises made to them by their university were similar to the beliefs of individuals with whom they maintained direct peer relationships [56]. Thus, utilizing peer mentoring within an organizational context should enhance necessary leadership skills such as critical thinking, ethical decision-making, and effective problem-solving [57].

We agree that social information processing can occur through peer mentoring relationships because peers share information and knowledge related to the completion of their work through such ties [58]. For example, peer advice networks are characterized by cognitive trust or the belief that another has the ability and competence to provide help [59]. Therefore, asking a peer for advice and looking to them as role model of excellent performance are both an indication of respect for the opinion of that individual and an expectation that help from that individual is available and useful [60]. These expectations suggest that peer mentoring, through the exchange of information, can help individuals understand and interpret the complexity of the profession and their current or future leadership role. The practice of sharing information highlights an important process that may explain how peer mentoring relationships can serve as a mechanism for social influence that is relevant for enhancing diversity within organizational leadership.

Social influence among peers can also occur when individuals draw comparisons between themselves and other individuals in order to better understand ambiguous situations [61]. Traditional social comparison theory [46] suggests that: (1) individuals learn about themselves through comparison with others; (2) individuals who have similar demographic characteristics are often chosen for comparison; and (3) social comparisons will have strong effects when objective non-social comparisons are unavailable and when others' evaluations are important to the individual. These tendencies are clearly relevant to the notion of lateral mentoring that has already been shown to help with early socialization, learning, moral support and the need to have safe conversations about the complex dynamics that impact their careers [62].

Social comparison is prevalent in organizations because, in many cases, evaluations regarding individual job performance within the organization are subjective. Social

comparison can result in similar perceptions between individuals when one identifies with his or her peers. However, such social comparison may also lead to negative identity when there are status differences as is the case for traditional hierarchical mentoring, particularly when power and aspects of diversity or identity intersect [27]. In these cases, identity discordance or conflict can provide negative signals that the individual does not fit within the organization or the profession. Social comparison processes could be one explanation for research showing a relationship between lack of diversity and disinterest in academic disciplines or specializations among medical students [63]. However, when peers provide feedback on another's performance, this input can seem less threatening and serve as signal to the individual regarding how she or he should interpret the other more formal evaluations as they develop and perform within various leadership roles [62].

Thus, peer relationships are often used for social comparison because they develop between individuals with similar attributes or aspirations [64,65]. For instance, Wheeler and Miyake showed that social comparison was most frequent among close friends, followed by peers with whom individuals were somewhat close, and least likely among individuals who were not friends [66]. Peer relationships defined by friendship ties involve expressions of personal affect, social support, a sense of identity and personal belongingness [67]. Individuals depend on peers for counseling and companionship, especially for sensitive issues [68]. For example, Morrison found that peer network size was positively related to organizational commitment, while advice network size was not [69]. Krackhardt and Stern demonstrated that individuals were more likely to share resources with peers from other departments than with non-peers during a simulated organizational crisis [70]. Finally, individuals tend to make career decisions that are similar to those of their peers [71]. This tendency to engage in social comparisons has led some to use peer mentoring groups as a tool for facilitating collaboration among junior faculty in order to provide peer support which is found to be critical during early career stages [72].

Overall, these theoretical perspectives (social learning, social information processing and social comparison) provide the basis for our argument that peer relationships serve as an important source of social influence, identity development and social support. Prior evidence suggests that individuals make social comparisons with their peers, identify with individuals they consider models or exemplars of what they would like to become themselves and exchange knowledge and information with peers they see as knowledgeable [73]. Individuals are therefore likely to compare their professional and job-related perceptions to those with whom they have advice, peer and/or friendship ties, particularly when these relationships are strong (versus weak) and reciprocal (versus unidirectional) in nature [74]. Given that access to diverse peers may be much more readily available within most organizations, opportunities for peer support, feedback and knowledge transformation among diverse individuals maybe greater than by traditional hierarchical mentoring alone [75]. These peer relationships can clearly provide a "safe space" for the necessary work of identity for diverse individuals within leadership roles [7].

1.3. Mentoring Diverse Leaders as Identity Work

Work by Ibarra and Petriglieri [76] specifically outlines identity work as "people's engagement in forming, repairing, maintaining and strengthening or revising their identities" (p. 10). Several theories and perspectives highlight the important identity work through direct experiences. For example, Mezirow provides an excellent theoretical lens to understand the importance of transforming oneself through the process of immersion experiences [77]. These experiences are especially important when individuals encounter novel, unfamiliar or conflicting situations. Over time, they prompt these individuals to challenge and sometimes question traditional patterns of thinking or reacting. This process can be true not only for individuals but also for mentors, sponsors, and especially for leaders within the organization. Whenever we encounter a disruptive event or challenging environment (or experience), it can trigger a transformative learning process, create new points of reference, and prompt self-

reflection of existing values, norms, ideas or self-discovery. Each of these triggers can become a pivotal point of relating differently from others within the organization and the broader society. This reaction is especially true when individuals are faced with multiple, conflicting or ambiguous identities [78]. These challenges and/or conflicting identities can be addressed by helping to support how people manage discrepancies among both personal as well as social identities.

For example, Illeris outlines identity work through the lens of transformative learning as the stripping away of multiple layers of self-identity [79]. This transfiguration (or change) can only happen when people experience motivation that is strong enough to trigger transformative learning. Based on previous leadership development work, the conclusion is that mentoring and identity transformation are important to effective leadership development, especially for diverse leaders in organizations [80–82]. In order to mobilize and influence others, leaders must see themselves and be viewed by others as legitimate or "credible" within their assigned role. The process of legitimacy involves putting forth a claim by asserting oneself as an influential or knowledgeable person, which is then either accepted (validated) or rejected by followers and other leaders [30]. This claiming/accepting process takes place more smoothly for people who fit into prototypical profiles of leaders; meaning, they embody the commonly held and accepted characteristics of those in organizational leadership roles [31]. Rejection by followers or others in authority can challenge leadership identity and impede overall effectiveness [50]. Mentoring functions, such as role modeling, acceptance/confirmation and sponsorship, can be a critical tool for organizations seeking to engage in effective development for diverse leaders [83]. Thus, when it comes to developing diverse leaders, organizations need to pay attention to the importance of mentorship as identity work in supporting leadership development especially when developing diverse leaders [84].

The importance of identity work is supported by Heaphy and Dutton who argue that individuals are active participants in their own development as they engage in the co-creation of who they are as individuals and especially as leaders [85]. This means that mentoring relationships not only provide the opportunity for personal growth and professional development but also define, shape, and transform their co-constructed identities in the workplace. This transformative process can be seen in research that examines individual work identity and job crafting of one's career [86], the influence of role models on career and personal identity [83] and the influence of identity on mentoring relationships to both influence and affirm personal identity among high-potential managers [83,87]. This process points to definitions of identity work as both important to shaping external or public displays of role-appropriate qualities of leadership and the dual need for internal coherence or consistency of personal values and valued leadership behaviors [76]. It also calls for the need to gain a better understanding of how to balance socially constructed notions of organizational leadership with personal definitions of leadership as personal identity that can be supported and facilitated by high quality mentoring relationships [7]. The need is especially important for developing diverse leaders as the notion of psychological safety has been shown in previous work to shape leadership style and have a positive impact on both team and organizational outcomes [84]. Thus, psychological safety is critical to identity work as diverse leaders attempt to manage identity conflicts, discrepancies and potential identity threats to derive a meaningful sense of self as a leader [88].

Despite the substantial research on the importance of mentoring for leadership development, there is still a need for more examples of how to facilitate, support and integrate identity work within formal efforts to effectively develop diverse leaders. Some research points to leadership development initiatives that shift away from traditional hierarchical, power-focused, and competitive views of leadership toward collaborative, interconnected, and values-based approaches as one effective evidence-based model [89]. Recent evidence exists that relational and identity-based approaches to developing diverse leaders are effective especially in building the pipeline of women into leadership roles

[90]. Thus, developing evidence-based leadership development approaches is essential to facilitate positive identity work that integrates personal, social and leadership identities that are necessary for effectiveness as an organizational leader and for well-being as a person [91].

This insight suggests that paying attention to and developing efforts to support the necessary identity work is critical. Clearly this perspective will require a shift from leadership development-based approaches that tend to focus on reinforcing traditional organizational norms and definitions of leadership and toward more relational, inclusive, and collaborative definitions of what it means to be a leader [92]. This type of shift is often discussed within feminist models arguing that effective and inclusive leadership development requires a safe environment. This means that a protected harbor is necessary for the critical "identity work" in which individuals can examine, challenge, redefine traditional definitions and develop more inclusive notions of leadership that integrate diverse social identities [93]. The notion of providing a safe space for identity work to take place is consistent with the idea of "holding environments" which form the basis of high-quality relationships and the work of effective mentoring.

1.4. Mentoring as Holding Environments

Holding environments [94] have traditionally been studies within developmental and clinical psychology to capture relationships that entail care-giving and care-receiving as necessary support for individual development and overall well-being [95]. When confronted with conflicting situations, new challenges and other anxiety-provoking circumstances, individuals both seek out and welcome these holding environments that can be provided by co-workers, friendships, and other interpersonal connections. These holding environments do not necessarily involve close personal friendships but are constructed for specific situations that can address both work-related and non-work-related issues or challenges. As Kahn [94] states, "holding environments at work emerge when opportunity, desire and competence coincide" (p. 266).

Mentoring as development networks have been described as holding environments especially for leaders [96]. These developmental networks provide a range of career and psychosocial functions of mentoring that include identity development especially for diverse leaders. We argue that the connection of identity work with holding environments is important for developing diverse leaders as it ensures that leaders can safely examine identity ambiguity, resolve identity conflict and engage in personal transformation. Much like Illeris' notion of identity transformation, the concept of holding environments includes the idea that leadership challenges are "developmental triggers" that facilitate identity transformation and the building of leadership competence and resilience [79, 97]. Within this approach, three types of holding behaviors are important for effective leadership identity work to take place: empathic acknowledgement, enabling perspective and containment [94,96,98].

Empathetic acknowledgement involves individuals such as mentors, colleagues and peers who recognize, confirm and validate the lived experiences, conflicts and challenges encountered by an individual or leader. Within the context of identity work, empathetic acknowledgement often involves the confirmation of issues such as unconscious bias, sexism, racism and various forms of discrimination that are validated as legitimate experiences from a point of mutual understanding and empathy. Enabling perspective is where others help the individual to make sense out of conflicting or contradictory information by engaging in self-reflection and insights shared by peers, mentors, and other developmental relationships. Containment involves the accessibility of others and often involves showing compassion, warmth and accepting others' feelings without judgement or condemnation. The holding behavior of containment is focused on creating a safe environment for others to express emotion and serves as a non-judgmental sounding board and involves high levels of interpersonal trust. In contrast, enabling perspective focuses on mutual sensemaking where the end results in what is often referred to as negotiated

interpretation of sharing identity-related experiences or challenges. Empathic acknowledgement can also involve creating safe spaces for shared experiences. However, this phenomenon places a greater emphasis on validation and identification that helps to restore regard and respect given challenging situations and experiences.

These holding behaviors are like psychosocial functions of mentoring relationships as documented previously by Kram's original work and later research by Higgins and Kram [2,10]. In comparison to the career functions which are instrumental in nature, mentoring functions that create holding environments are more relational in nature and require some levels of interpersonal trust, shared identity and psychological safety. It also means that individuals who provide this support have some level of availability and competence to create and sustain these holding environments that not only provide support but also help individuals with coping, adjustment and mobilizing productive responses to challenging situations [94]. Thus, it would make sense to expect peer mentoring to provide these types of holding environments especially when peers share the same social identity group. Peer mentors can be uniquely available and over time become trusted to provide containment, empathetic acknowledgement and enabling perspective within these lateral relationships. Facilitating formalized peer mentoring opportunities may also be useful in providing necessary identity-relevant support as holding environments for diverse leaders. Having formalized peer mentoring over time may provide the opportunity for high-quality relationships to emerge and provide levels of psychological safety that are necessary for openness, sharing of experiences and relational trust that are critical for identity-based leadership development.

Thus, we explore peer mentoring among African American managers within a formalized program to support identity-based leadership development and cultivate mutually beneficial holding environments. We examine a formal diverse leadership pipeline program that provides facilitated workshops along with peer mentoring circles over a 1-year period. Using qualitative interview and focus group data, we explore the role that containment, empathic acknowledgement and enabling perspective experiences serve as impactful tools for identity-based mentoring and leadership development among African Americans. Our objective is to provide an evidence-based approach for the use of holding environments via peer mentoring in order to provide support for the range of identity-specific challenges that diverse leaders uniquely face within traditional organizations. Our goal is to demonstrate that the use of facilitated peer mentoring with an explicit focus on identity-related work and the cultivation of holding environments can increase the overall effectiveness of formal mentoring efforts specifically for developing African American leaders.

2. Methods

2.1. Research Setting

Our research setting was an executive education leadership development program aimed at strengthening the pipeline of African American leaders. Individuals were recruited from a variety of different organizations, industries and geographic regions within the United States. University faculty designed the program and facilitated all of the workshops which covered key topics including leadership competency, mentoring, organizational politics, racism and bias (both unconscious and deliberate). A key feature of the executive education program was to enable each participant to establish personalized developmental goals necessary for their advancement and overall well-being in the next phases of their career.

Participants were engaged in several day-long interactive workshops that included fundamentals of effective mentoring, peer mentoring and building a diverse development network. Each program session was facilitated by University-based research faculty members and included both current research and best practices along with cases and interactive exercises to help engage participants. A total of 41 African American mid-level

managers participated in this year long program from companies within the private, public and non-profit sectors. Participants were recruited based on having achieved some level of current leadership responsibility within their respective organizations. All participants were based in the U.S. but worked in both local and global companies.

2.2. Peer Mentoring Circles

Participants were placed into peer mentoring "circles," which each consisted of 3–4 program participants. Each peer circle participated in workshop exercises and discussions during the program to help with the initial phases of relationship cultivation. Each peer circle was also assigned one of the faculty members as a "coach" to answer questions, provide check-ins to ensure the circles were staying connected and serve as a resource for questions, concerns and emerging needs throughout the program's duration. Attention was paid to ensure that each peer circle had a balance of diversity in terms of industry, gender, geographic region and professional expertise. All members were classified as "mid-career" in terms of overall tenure within their respective organizations. In addition, participants from the same organizations were deliberately not put into the same peer mentoring circles. Between program sessions, each faculty member did "check-ins" virtual meetings with the participants, for both ongoing evaluation and data collection.

2.3. Data Collection

Two key points of data collection took place during the formal program. The first assessment took place three months after the initial program session where overview materials were presented, peer circles were formed and a discussion of the benefits and responsibilities of mentoring relationships were outlined. Each faculty coach conducted an hour-long telephone interview with each participant within their assigned peer mentoring circles. This initial point of data collection was to gather early-stage mentoring information including contact frequency and to solicit feedback from each participant. Several interview questions about their mentoring experiences were included and adopted from existing mentoring questions developed and validated by Ragins & McFarlin [99] and Thomas [100]. Interview questions included both open-ended questions and items taken from these established mentoring scales which were asked by faculty coaches. Using these established scales, participants were asked to verbally respond using a standard 1-5 Likert scale. We included several open-ended interview questions during the first phase of data collection and open-ended focus group questions during the second phase of data collection.

At the completion of the formal program, faculty coaches conducted in-person focus group interviews with each mentoring circle. A set of questions were developed that addressed participants' overall evaluation of the mentoring experience. Participants were also asked about the content of their interactions, sharing information, and reflections on the value of their peer-to-peer interactions. These discussions used open-ended questions which allowed for a content examination of the development of "holding behaviors" within the peer mentoring circles without prompting or pre-disposing responses to various questions.

2.4. Measures

Our primary measures focus on the presence and frequency of holding behaviors. We examined participants' open-ended responses and coded mentions of behaviors or actions that illustrated the presence of holding behaviors as outlined by Kahn [94]:

1. Containment—creating a safe, reliable interactions that includes opportunities for others to express emotions, feelings and reactions. Key behaviors include: accessibility, attention, compassion and acceptance.

2. Empathic Acknowledgement—creating empathic context that affirms the other's sense of being knowledgeable, worthwhile and valued. Key behaviors include: empathy, validation and acknowledgement of others self-exploration and questioning.
3. Enabling Perspective—creating a context in which others can discover a sense of competence, positive sense of self and creating a space for rational thought and action. Key behaviors include: self-reflection, sensemaking, task focusing and negotiated interpretation.

3. Results

Our analysis focused primarily on qualitative data that was collected during both mid-program check-in interviews and focus groups at the end of the formal program. Thirty-seven of 41 participants were interviewed during the program mid-point which yielded a follow-up rate of 90%. This initial interview phase was to confirm that the mentoring relationships formed during were taking place and involved some level of meaningful interactions. A vast majority of participants reported having "occasional or frequent" (78%) interaction. Mentoring connections typically occurred via telephone (43%) or some combination of telephone and email (49%). Consistent with previous research, these early interactions tended to focus on "getting to know one another" with discussions centering on work-related activities and on the developmental action plans required as part of the formal program (56%). The overall level of satisfaction within this initial phase was quite high. A majority of participants (81%) reported that their relationship was either "effective" or "very effective" when asked to verbally respond based on a 5-point Likert-type scale. Using responses to questions that were adapted from previous mentoring scales, we examined the presence of either career or psychosocial mentoring functions during the early phase of these peer relationships. Among the most frequent types of mentoring functions reported were: role modeling (85%); acceptance and confirmation (75%); exposure and visibility (82%), counseling (52%) and friendship (27%).

Interestingly, some participants reported also talking about personal issues such as family–work balance and managing workplace conflicts, but these types of activities occurred less frequently among participants during this early stage of relationship initiation. During this early phase, a number of participants mentioned the importance of allocating time to cultivate their mentoring relationships and the value of having access to African American peers as part of the program, particularly when their organization lacked racial diversity among its leadership. While some discussion surrounding issues of race and how to be effective as a person of color in the organization was reported during our initial follow-up, the frequency of this type of sharing was limited during this initial phase of the peer mentoring program.

Peer Mentoring Interviews. Overall perceptions of the mentoring experience among our African American leaders were quite positive and remained positive across the year-long program. During the last session of the program, we conducted face-to-face group interviews to discuss the mentoring experience and gain a better understanding of the value that participants received from interacting within their peer circles. Interestingly, we witnessed a change in the type of information that was shared between members of the mentoring circles as they continued to cultivate their relationships. Circle members reported most frequently talking about their own personal experiences related to career or life success and satisfaction relative to the previous interviews. Later discussions seemed to shift toward more specific leadership and personal development concerns (e.g., culture, dealing with conflict, racial issues). It is reasonable to conclude that these "tough" issues emerged as interpersonal trust was developed overtime during the program. There was also more information about the shared experiences surrounding racial identity related to their roles as leaders within the organization which was less prominent during the initial phase of the mentoring relationship.

Peer Mentoring Holding Behaviors. One of key aspects of our analyses was to examine the presence and type of holding behaviors within these peer mentoring relationships.

We categorized participant responses based on the definitions of holding behaviors and found evidence of all three types as described by Kahn [94] but in varying degrees. Most frequently mentioned by peers were holding behaviors within the category of “enabling perspective.” This category involves peer mentoring relationships that create a context where one can renew a sense of self in reference to work and other roles. The enabling perspective involves “making sense” out of experiences, situations, conflicts and other circumstances that they face within their leadership role. These enabling experiences involved conversations that provided a safe space for self-reflection, interpretation and on gaining greater understanding. Participants frequently shared that these peer mentoring relationships “provide an unbiased perspective” that allow for “an independent perspective not clouded by my organization.” Often mentioned were issues of “fresh perspective,” having an “external perspective,” and advice from someone who is “not biased by corporate culture.” Peer mentoring was also described as providing a strong “sounding board” from someone who “helped with my awareness.”

Peer conversations that helped to make sense out of the experiences that were being faced which were similar because of issues of race and leadership. These conversations provided an “objective sounding board” because peers who were external to their organizations were often cited as most valuable. These conversations also included opportunities for peers to provide “greater focus” to one another in terms of what could be changed or controlled about their current circumstances. Participants shared that these conversations where sensemaking took place often lead to useful strategies for how to manage challenges, tasks and their overall careers. This finding is similar to what Kahn [94] calls, “negotiated interpretation” or helping others develop actionable interpretations of their situations where individuals feel less restricted due to anxiety, anger, or other emotions. The use of peers for these enabling perspectives was clearly the most dominant type of holding behaviors experienced by these peer mentoring circles (see Figure 1). A noteworthy observation is that enabling perspective as holding behaviors did not seem to be negatively impacted by the lack of face-to-face interaction among the peer mentoring circles. Engaging in sensemaking, self-reflection and negotiated interpretation appear to have taken place via distance communication tools (phone, video, email, etc.).

Next most frequently reported within these peer relationships at the end of the year-long program were holding behaviors within the category of “empathic acknowledgment.” These peer experiences focused on affirming ones’ sense of self, providing validation for others’ skills, knowledge or abilities and the expression of empathy where there is a shared identification of experiences around issues of race, gender, and what does it means to be a “leader.” Participants mentioned that these peer relationships were valued because of the “safe haven” that provided “confidentiality” and “someone you can safely share ideas with.” The presence of empathy, affect, understanding and psychological safety were key aspects of these leaders’ experiences of holding behaviors from peer mentors of color (see Figure 1). Issues of confidentiality were mentioned frequently as part of empathic acknowledgment received from peers. This finding reflected a reluctance to trust those inside of the organization because of concerns with negative perceptions, backlash, retaliation and unwelcome disclosures, which could damage future career opportunities. Holding behaviors among these African American peer leaders may provide a unique “safe space” to address sensitive matters of race and identity that cannot be addressed with traditional senior level sponsors inside of the organization or even other peers inside of their organization.

We received fewer responses that would be examples of containment according to Kahn [94]. This dimension of holding behaviors involves creating a safe and reliable environment for sharing of experiences and feelings. The dimension of containment can appear to overlap somewhat with empathic acknowledgement. However, one distinction is the level of affect and depth of relational engagement. Containment involves paying attention to and showing compassion for other’s situation, experiences and concerns. In contrast, empathic acknowledgement involves greater involvement through empathy,

validation and is much more likely to include reciprocal sharing. One way to define containment is by the individual feeling "cared for" or heard without negative judgements. Responses from African American peers that reflected containment centered around objective listening from a non-threatening, unbiased perspective. The notion of confidentiality and lack of "conflict of interest" were frequently shared as comments from peers reflecting this notion of containment. Thus, containment is clearly about "being heard" and receiving a compassionate, confidential response from an individual that shares similar identity-related issues or concerns (see Figure 1).

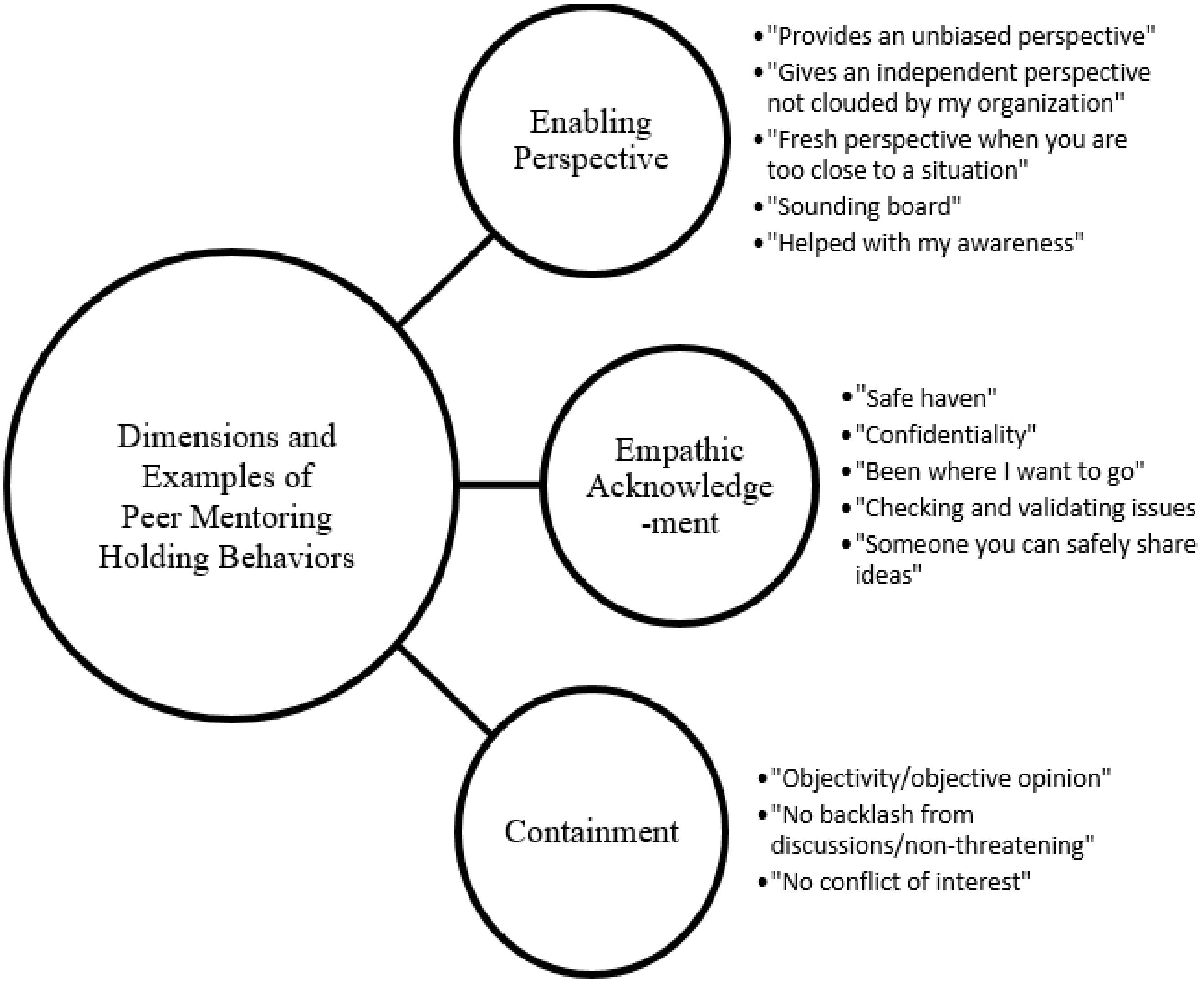

Figure 1. African American Leaders' Peer Mentoring Experiences.

During our group discussions, we also provided an opportunity for participants to share challenges and issues they faced within these peer mentoring circles. The comments from participants reflected an interesting paradox about the benefits versus limitations of external peer mentoring. While enabling perspective, empathic acknowledgement and containment were clearly benefits of these relationships, peer mentors external to the organizations have some limitations. For example, participants frequently shared that while external peers provide a "safe haven," the limitation is that they "may not appreciate some nuances of the organization" or "lack knowledge of key players" within their company. Issues such as "not knowing the structure" or "not knowing everything about their industry" were frequently mentioned. Lacking internal knowledge of the organizational

dynamics was cited as a particular limitation when dealing with political issues or dynamics that are specific to a particular industry (e.g., public sector versus private sector).

A second limitation that participants shared about having external peer mentors related to the notion of mentors as a source of protection within their specific organizations. Several participants shared that while valuable, these formal peer mentoring relationships provided "limited protection" because they did "not have knowledge of the political scheme and key players" or ties inside of the same organizations. One participant shared that although supportive, their peer mentor "does not have their ear to the ground to help you navigate" as another example of the lack of protection. This finding is interesting as previous work tends to restrict issues of protection to being provided exclusively by more senior sponsors rather than lateral peer mentors. However, these African American leaders frequently mentioned that peers provided protection and a buffer within the environment, but that support is specific to internal rather than external peer mentors. The notion of "lack of protection" was frequently mentioned as a challenge and issue of concern among these African American leaders.

4. Discussion and Future Directions

Based on our research findings, we contend that engaging in identity-based sensemaking for emerging African American leaders is essential for supporting their development especially as they build competence and confidence throughout their careers. This identity-based sensemaking is foundational for diverse leaders as they navigate unique challenges while often lacking access to informal networks and sources of information which allow them to effectively "make sense" of their experiences within the organization. Furthermore, the feedback and advice they may receive from non-Black mentors often comes from vastly different experiences within their organization [101]. Feedback from our participants clearly indicates that peer mentoring provides a unique safe space for this identity work to take place and can be facilitated by a well-designed formal program. Our program participants shared that peer mentoring provided a safe environment for the necessary work of identity to take place. This identity work takes the form of holding behaviors such as enabling perspectives, empathic acknowledgement and containment that are critical for the development, support and validation. Peer mentoring created a "safe space" for these diverse leaders to "make sense" out of issues of race and leadership. Our findings clearly show the benefit of external peer mentors who share key aspects of social identity as they provide both support and validation for diverse leaders that can be absent within traditional hierarchical mentoring especially when there are differences based on dimensions such as race and gender [3].

Peer mentoring was clearly seen as a positive resource that provides enabling perspectives which create a context for people to engage in sensemaking, self-reflection, and negotiated interpretation [94]. Our program participants frequently mentioned that peer mentors provide an "unbiased perspective" and "independent perspective" that are essential for making sense out of their roles as diverse leaders. Having access to peer mentors who share the same racial group identity appears to be essential for sensemaking that takes the unique issue of race (and other identity dimensions) into consideration. These "negotiated interpretations" that took place during peer mentoring interactions allowed African American leaders to have protected conversations about race and leadership. One of the benefits of these conversations was that they supported peer mentors in gaining a clear understanding of personal versus organizational factors that can impact their overall effectiveness. These discussions were important resources for diverse leaders to develop both competence and confidence within their leadership roles.

As we observed from participants' feedback, having protected conversations to better understand their role as a leader within the context of race, gender and other social identities provides a critical developmental experience to help them understand and navigate their organizational and career journeys. Thus, these enabling perspective holding behaviors provided by peers are a vital resource for developing clarity in both thought

and action as a leader. An enabling perspective may also be a resource that is uniquely provided by peers external to the individual's organization. Our participants frequently mentioned that peer circles provided an unbiased perspective or sounding board that was not biased or clouded by the dynamics within their own organizations. Clearly this is a critical aspect of the sensemaking process that external peers can provide and one that is important to the unique experience of being an African American leader.

One of the key features of our formalized program is the longitudinal nature of the peers mentoring effort. Clearly having peer mentoring over this year-long period allowed for the development of holding behaviors that Kahn [94] labeled as "empathic acknowledgment." This acknowledgement involves empathy, validation and acceptance of others' experiences and perspectives. Thus, participants in peer relationships must have developed some level of trust which cannot be accomplished by a short-term program or a single workshop experience. Participants shared feedback after at the end of our year-long program that peer mentors provided a safe, confidential and trusted source of support and validation. This finding is consistent with Kahn's notion that holding behaviors provide an affirming environment where the individual feels safe, valued and accepted [94]. This type of relational trust must be developed over time and facilitated by norms of acceptance, identity and validation which were essential elements of our program model. While we did not explicitly assess the difference between empathic acknowledgement of external versus internal peer mentors, feelings of psychological safety may likely be higher if provided by external peers because concerns over confidentiality, politics and trust are not as problematic as in the case of peers inside of the same organization.

We observed less frequent examples among our peer mentors of containment, which takes place when peers "remain in other's vicinity, allowing time and space for uninterrupted contact and connections" (p. 269) [94]. This absence may be a consequence of the remote nature of these peer mentoring relationships, since individuals were not only in different organizations but frequently in different geographic locations. Our participants shared that one challenge of these external peer mentors was lack of face-to-face access, which is essential for experiencing containment as a holding behavior. They often shared concerns over being unable to connect in "real time" such that gaining access was limited due to differences in location, time zone and overall availability. Containment frequently involves an individual feeling "cared for," connected and "un-alone," which can be difficult to maintain within peers who face obstacles to availability and accessibility. While there may be no way to provide all aspects of holding behaviors in a signal peer mentoring program, participant feedback speaks to the importance of creating a diverse developmental network such that multiple relationships are available in order to meet leaders' diverse needs [2].

This research confirms the importance of peer mentoring, identity work and holding environments for the development of diverse leaders. Over this year-long program, we witnessed important "identity work" being done within these peer mentoring circles. Perspectives based on social categorization argue for identity work as a process of integration of identities [78]. It is clear from the findings of our program that peer mentors can provide both a safe space and a buffer for the necessary work of identity. As peers continue to develop trust over time, they may experience identity transformation as the co-creation of new identities within organizational leadership that are an integration of both traditional and diverse leadership identities. This type of co-creation involves the transformation of diverse identities into a collective or superordinate identity similar to what has been discussed as strategies for the reduction of intergroup bias and conflict, known as the common identity model [102]. This acceptance and integration of previously conflicting or diverse identities are suggested as an important transformation process that can be facilitated by diversified mentoring relationships when identity work is an explicit and permitted aspect of developing diverse leaders [103,104].

This acknowledgement suggests that identity work must be part of the explicit content of mentoring diverse leaders that provides a "safe place," helps to support

sensemaking and provides validation in order to overcome biases and traditional obstacles. This approach may be useful for other types of mentoring where issues of trust and psychological safety are important such as leadership programs that are devoted to role modeling of ethical behavior [105]. If mentoring efforts are not sensitive to the necessary work of identity for diverse leaders, then acceptance and confirmation challenges will continue to serve as obstacles to forming high-quality mentoring relationships [106]. This component is an important aspect of mentoring diverse leaders that not only acknowledges that transformative processes are important but also accepts that shared identity work is necessary. Instead of focusing on a one-size-fits all approach to mentoring diverse leaders, programs that combine efforts across different types of mentoring (e.g., one-to-one, peer mentoring, role modeling, reverse mentoring, etc.) and purposefully provide a safe space for identity work to take place may be a more effective approach for developing the pipeline of diverse leaders [101]. While peer mentoring, as well as other types of programs, may be seen as more complex and expensive, the benefits of diversified mentoring relationships that is documented by extensive research support a strong return on this investment of time and resources [28].

Author Contributions: Conceptualization, A.J.M., S.B.-B., D.M.P.J.; methodology, A.J.M., S.B.-B., D.M.P.J.; formal analysis, A.J.M., S.B.-B., D.M.P.J.; writing-original draft preparation, A.J.M., S.B.-B., D.M.P.J.; writing-review and editing, A.J.M., S.B.-B., D.M.P.J.; project administration, A.J.M. All authors have read and agreed to the published version of the manuscript.

Funding: This research received no external funding.

Institutional Review Board Statement: Institutional Review Board review and approval were not required for this study due to classification as program evaluation.

Informed Consent Statement: Informed consent was obtained from all subjects involved in the study as part of program participation.

Data Availability Statement: Datasets from this research and not available.

Conflicts of Interest: The authors declare no conflict of interest.

References

1. Allen, T.D.; Eby, L.T.; Poteet, M.L.; Lentz, E.; Lima, L. Career Benefits Associated with Mentoring for Proteges: A Meta-Analysis. *J. Appl. Psychol.* **2004**, *89*, 127–136, doi:10.1037/0021-9010.89.1.127.
2. Higgins, M.C.; Kram, K.E. Reconceptualizing mentoring at work: A developmental network perspective. *Acad. Manag. Rev.* **2001**, *26*, 264, doi:10.2307/259122.
3. Murrell, A.J.; Blake-Beard, S. *Mentoring Diverse Leaders: Creating Change for People, Processes, and Paradigms*; Routledge Press: New York, NY, USA, 2017.
4. Thomas, D.A.; Gabarro, J.J. *Breaking through: The Making of Minority Executives in Corporate America*; Harvard University Press: Boston, MA, USA, 1999.
5. Newman, A.; Donohue, R.; Eva, N. Psychological safety: A systematic review of the literature. *Hum. Resour. Manag. Rev.* **2017**, *27*, 521–535.
6. Cheng, C.-Y.; Sanchez-Burks, J.; Lee, F. Connecting the dots within creative performance and identity integration. *Psychol. Sci.* **2008**, *19*, 1178–1184.
7. Murrell, A.J.; Blake-Beard, S.; Porter, D.M.; Perkins-Williamson, A. Inter-organizational formal mentoring: Breaking the concrete ceiling sometimes requires support from the outside. *Hum. Resour. Manag.* **2008**, *47*, 275–294.
8. Roberts, L.M.; Cha, S.E.; Hewlin, P.F.; Settles, I.H. *Bringing the Inside Out: Enhancing Authenticity and Positive Identity in Organizations, Exploring Positive Identities and Organizations: Building a Theoretical and Research Foundation*; Routledge: New York, NY, USA, **2009**; pp. 149–170.
9. Allen, T.D.; Eby, L.T.; Chao, G.T.; Bauer, T.N. Taking stock of two relationship aspects of organizational life: Tracing the history and shaping the future of socialization and mentoring research. *J. Appl. Psychol.* **2017**, *201*, 324–337.
10. Kram, K. E. *Mentoring at work: Developmental relationships in organizational life*. Scott, Foresman: Glenview, IL, USA, 1985.
11. Allen, T.D.; Eby, L.T.; Lentz, E. Mentorship behaviors and mentorship quality associated with formal mentoring programs: Closing the gap between research and practice. *J. Appl. Psychol.* **2006**, *91*, 567–578, doi:10.1037/0021-9010.91.3.567.
12. Brabazon, T.; Schulz, S. Braving the bull: Women, mentoring and leadership in higher education. *Gend. Educ.* **2018**, *32*, 1–18, doi:10.1080/09540253.2018.1544362.

13. Murrell, A.J.; Zagenczyk, T.J. The gendered nature of role model status: An empirical study. *Career Dev. Int.* **2006**, *11*, 560–578, doi:10.1108/13620430610692953.
14. Wanberg, C.R.; Welsh, E.T.; Hezlett, S.A. Mentoring research: A review and dynamic process model. In *Research in Personnel and Human Resources Management*; Elsevier Publishing: New York, NY, USA, 2004; Volume 22, pp. 39–124.
15. Murrell, A.J.; Crosby, F.J.; Ely, R.J. (Eds.) *Mentoring Dilemmas: Developmental Relationships within Multicultural Organizations*; Taylor Francis: Philadelphia, PA, USA, 1999.
16. Scandura, T.A. Mentorship and career mobility: An empirical investigation. *J. Organ. Behav.* **1992**, *13*, 169–174, doi:10.1002/job.4030130206.
17. Kroll, J.; Blake-Beard, S.; McMillian-Roberts, K. An exploration of the peer group mentoring experiences of university female basketball athletes. *Mentor. Tutor. Partnersh. Learn.* **2020**, *28*, 229–252, doi:10.1080/13611267.2020.1749355.
18. Kram, K.E.; Isabella, L.A. Mentoring alternatives: The role of peer relationships in career development. *Acad. Manag. J.* **1985**, *28*, 110–132.
19. McManus, S.E.; Russell, J. Peer mentoring relationships. In *The Handbook of Mentoring at Work: Theory, Research, and Practice*; SAGE Publications: Thousand Oaks, CA, USA, 2012; pp. 273–298.
20. Ensher, E.A.; Heun, C.; Blanchard, A. Online mentoring and computer-mediated communication: New directions in research. *J. Vocat. Behav.* **2003**, *63*, 264–288, doi:10.1016/s0001-8791(03)00044-7.
21. Friedman, R.; Kane, M.; Cornfield, D.B. Social support and career optimism: Examining the effectiveness of network groups among black managers. *Hum. Relat.* **1998**, *51*, 1155–1177.
22. Giscombe, K. Advancing women through the glass ceiling with formal mentoring. In *The Handbook of Mentoring at Work: Theory, Research, and Practice*; SAGE Publications: Thousand Oaks, CA, USA, 2012; pp. 549–572.
23. Nair N, Vohra N. Mentoring as a means to achieve inclusion. In., Murrell, A.J. & Blake-Beard, *Mentoring diverse leaders: Creating change for people, processes and paradigms*, Routledge, New York, NY, USA, **2017**, pp. 124-144.
24. Blake-Beard, S.D. The costs of living as an outsider within: An analysis of the relationships and career success of Black and White women in the corporate sector. *J. Career Dev.* **1999**, *26*, 21–36.
25. Dreher, G.F.; Ash, R.A. A comparative study of mentoring among men and women in managerial, professional, and technical positions. *J. Appl. Psychol.* **1990**, *75*, 539.
26. Viator, R.E. The association of formal and informal public accounting mentoring with role stress and related job out-comes. *Account. Organ. Soc.* **2001**, *26*, 73–93.
27. Ragins, B.R. Diversified mentoring relationships in organizations: A power perspective. *Acad. Manag. Rev.* **1997**, *22*, 482–521.
28. Blake-Beard, S.D.; Murrell, A.; Thomas, D. Unfinished business: The impact of race on understanding mentoring relationships. In *The Handbook of Mentoring at Work: Theory, Research, and Practice*; Ragins, B.R., Kram, K.E., Eds.; SAGE Publications: Thousand Oaks, CA, USA, 2012; pp. 223–248.
29. Blake-Beard, S., Murrell, A., Krothapalli, V., Halem, J., & Kweder, M. Mentoring as a connecting competency builder. In *Positive Organizing in a Global Society: Understanding and Engaging Differences for Capacity Building and Inclusion*; Routledge: London, UK, 2015; pp. 110.
30. Carroll, B.; Levy, L. Leadership development as identity construction. *Manag. Commun. Q.* **2010**, *24*, 211–231, doi:10.1177/0893318909358725.
31. Caza, B.B.; Wilson, M.G. Me, myself, and I: The benefits of work-identity complexity. In *Exploring Positive Identities and Organizations: Building a Theoretical and Research Foundation*; Roberts, L.M., Dutton, J.E., Eds.; Routledge Taylor and Francis Group: New York, NY, USA, 2009; pp. 99–123.
32. De Rue, D.S.; Ashford, S.J. Who will lead and who will follow? A social process of leadership identity construction in organizations. *Aca. Manag. Rev.* **2010**, *35*, 627–647.
33. Thomas, D.A. The impact of race on managers' experiences of developmental relationships (mentoring and sponsorship): An intra‐organizational study. *Jnl. of Org. Beh.* **1990**, *11*(6), 479–492.
34. Murrell, A. J., & James, E. H. Gender and diversity in organizations: Past, present, and future directions. *Sex Roles*, **2001**, *45*(5), 243–257.
35. James, E. H. Race-related differences in promotions and support: Underlying effects of human and social capital. *Org. Sci.*, **2000**, *11*(5), 493–508.
36. Beattie, R. S., & McDougall, M. F. Inside or outside HRM? Locating lateral learning: two case studies from the voluntary sector. HRM–the Inside Story, Sage: London, UK, 1997.
37. Young, A. M., & Perrewe, P. L. What did you expect? An examination of career-related support and social support among mentors and protégés. *J of Manag.* **2000**, *26*(4), 611–632.
38. Young, A. M., & Perrewé, P. L. (2004). The role of expectations in the mentoring exchange: An analysis of mentor and protégé expectations in relation to perceived support. *J of Manag. Iss.* **2004**, 103–126.
39. Eby, L.T.; Allen, T.D.; Hoffman, B.J.; Baranik, L.E.; Sauer, J.B.; Baldwin, S.; Evans, S.C. An interdisciplinary meta-analysis of the potential antecedents, correlates, and consequences of protégé perceptions of mentoring. *Psychol. Bull.* **2013**, *139*, 441–476.
40. Higgins, M.C.; Thomas, D.A. Constellations and careers: Toward understanding the effects of multiple develop-mental relationships. *J. Organ. Behav.* **2001**, *22*, 223–247.
41. Nonaka, I. A dynamic theory of organizational knowledge creation. *Org. Sci.* **1994**, *5*(1), 14–37.

42. Swap, W., Leonard, D., Shields, M., & Abrams, L. Using mentoring and storytelling to transfer knowledge in the workplace. *J. of Manag. Info. Sys*. **2001**, *18*(1), 95–114.
43. Murrell, A. J., & South-Paul, J. E. The emerging power of peer mentoring within academic medicine. In., Murrell, A.J. & Blake-Beard, *Mentoring diverse leaders: Creating change for people, processes and paradigms*, Routledge, New York, NY, USA, **2017**, pp. 85–103.
44. Bandura, A. Social-learning theory of identificatory processes. *Handbook of socialization theory and research*, **1969**, *213*, 262.
45. Salancik, G. R., & Pfeffer, J. A social information processing approach to job attitudes and task design. *Admin. Sci. Quart*. **1978**, 224–253.
46. Festinger, L. A theory of social comparison processes. *Hum. Rel.* **1954**, *7*(2), 117–140.
47. Bommer, W. H., Miles, E. W., & Grover, S. L. Does one good turn deserve another? Coworker influences on employee citizenship. *Jnl of Organ. Beh*. **2003**, *24*(2), 181-196.
48. Ibarra, H. Provisional selves: Experimenting with image and identity in professional adaptation. *Adm. Sci., Quart*. **1999**, 764–791.
49. Files, J. A., Blair, J. E., Mayer, A. P., & Ko, M. G. Facilitated peer mentorship: a pilot program for academic advancement of female medical faculty. *Jnl of Woman's Health* **2008**, *17*(6), 1009–1015.
50. Creary, S.J.; Caza, B.B.; Roberts, L.M. Out of the box? How managing a subordinate's multiple identities affects the quality of a manager-subordinate relationship. *Acad. Manag. Rev.* **2015**, *40*, 538–562, doi:10.5465/amr.2013.0101.
51. Ely, R. J., Ibarra, H., & Kolb, D. M. Taking gender into account: Theory and design for women's leadership development programs. *Acad. of Manag. Learn. & Educ*. **2011**, *10*(3), 474–493.
52. McClure, E., & Black, L. (2013). The role of the clinical preceptor: an integrative literature review. *Jnl. of Nurs. Educ.* **2013**, *52*(6), 335–341.
53. Coleman, J. S., Katz, E., & Menzel, H. (1966). Medical innovation: a diffusion study. Indianapolis, The Bobbs-Merril Company. *Science* **1966**, *12*, 481–483.
54. Burkhardt, M. E. Social interaction effects following a technological change: A longitudinal investigation. *Acad. of Manag. Jnl.* **1994**, *37*(4), 869–898.
55. Meyer, M. A. The dynamics of learning with team production: Implications for task assignment. *The Quart. Jnl. of Econ*. **1994**, *109*(4), 1157–1184.
56. Dabos, G. E., & Rousseau, D. M. Mutuality and reciprocity in the psychological contracts of employees and employers. *Jnl of App. Psych*. **2004**, *89*(1), 52.
57. Seto-Pamies, D.; Papaoikonomou, E. A multi-level perspective for the integration of Ethics, Corporate Social Responsibility and Sustainability (ECSRS) in management education. *J. Bus. Ethics* **2016**, *136*, 523–538.
58. Ibarra, H., & Andrews, S. B. Power, social influence, and sense making: Effects of network centrality and proximity on employee perceptions. *Admin. Sci. Quart*. **1993**, 277–303.
59. McAllister, D. J. Affect-and cognition-based trust as foundations for interpersonal cooperation in organizations. *Acad. of Manag. Jnl.* **1995**, *38*(1), 24–59.
60. Haas, M. R., & Hansen, M. T Different knowledge, different benefits: Toward a productivity perspective on knowledge sharing in organizations. *Strat. Manag. Jnl*. **2007**, *28*(11), 1133-1153.
61. Sparrowe, R. T., Liden, R. C., Wayne, S. J., & Kraimer, M. L. Social networks and the performance of individuals and groups. *Acad. of Manag. Jnl.* **2001**, *44*(2), 316–325.
62. DeCastro, R., Sambuco, D., Ubel, P. A., Stewart, A., & Jagsi, R. Mentor networks in academic medicine: moving beyond a dyadic conception of mentoring for junior faculty researchers. *Acad. Med*. **2013,** *88*(4), 488.
63. Sánchez, J.; Peters, L.; Lee-Rey, E.; Strelnick, H.; Garrison, G.; Zhang, K.; Spencer, D.; Ortega, G.; Yehia, B.; Berlin, A.; et al. Racial and Ethnic Minority Medical Students' Perceptions of and Interest in Careers in Academic Medicine. *Acad. Med.* **2013**, *88*, 1299–1307, doi:10.1097/acm.0b013e31829f87a7.
64. Marsden, P. V. Homogeneity in confiding relations. *Soc. Net.* **1988**, *10*(1), 57–76.
65. Gibson, D. E. Developing the professional self-concept: Role model construals in early, middle, and late career stages. *Org. Sci.* **2003**, *14*(5), 591–610.
66. Wheeler, L., & Miyake, K. Social comparison in everyday life. *Jnl. of Per. & Soc. Psych.* **1992**, *62*(5), 760.
67. Coleman, J. S. Social capital in the creation of human capital. *Amer. Jnl. Soc.* **1988**, *94*, 95–120.
68. Sias, P. M., & Cahill, D. J. From coworkers to friends: The development of peer friendships in the workplace. *West. Jnl. of Comm.* **1998**, *62*(3), 273–299.
69. Morrison, E. W Newcomers' relationships: The role of social network ties during socialization. *Acad. of Manag. Jnl*. **2002**, *45*(6), 1149–1160.
70. Krackhardt, D., & Stern, R. N. Informal networks and organizational crises: An experimental simulation. *Soc. Psych. Quart*. **1988**, 123–140.
71. Kilduff, M The interpersonal structure of decision making: A social comparison approach to organizational choice. *Org. Beh. & Hum. Dec. Proc.*, **1990**, *47*(2), 270–288.
72. Bussey-Jones, J., Bernstein, L., Higgins, S., Malebranche, D., Paranjape, A., Genao, I., ... & Branch, W. Repaving the road to academic success: the IMeRGE approach to peer mentoring. *Acad. Med*. **2006**, *81*(7), 674–679.

73. Zagenczyk, T. J., Gibney, R., Murrell, A. J., & Boss, S. R. Friends don't make friends good citizens, but advisors do. *Grp. & Org. Manag*. **2008**, *33*(6), 760–780.
74. Ostroff, C., & Kozlowski, S. W. Organizational socialization as a learning process: The role of information acquisition. *Per. Psych.* **1992**, *45*(4), 849–874.
75. Varkey, P.; Jatoi, A.; Williams, A.; Mayer, A.; Ko, M.; Files, J.; Blair, J.; Hayes, S. The positive impact of a facilitated peer mentoring program on academic skills of women faculty. *BMC Med Educ.* **2012**, *12*, 14, doi:10.1186/1472-6920-12-14.
76. Ibarra, H.; Petriglieri, J.L. Identity work and play. *J. Organ. Chang. Manag.* **2010**, *23*, 10–25, doi:10.1108/09534811011017180.
77. Mezirow, J. An overview on transformative learning. *Contemporary theories of learning*, **2009**, 90–105.
78. Ashforth, B. E., & Mael, F. Social identity theory and the organization. *Acad. of Manag. Rev*. **1989**, *14*(1), 20–39.
79. Illeris, K. Transformative Learning and Identity. *J. Transform. Educ.* **2014**, *12*, 148–163, doi:10.1177/1541344614548423.
80. Blancero, D.M. & Nessler, C.N.C. Mentoring latinos: An examination of cultural values through the lens of relational cultural theory. In., Murrell, A.J. & Blake-Beard, *Mentoring diverse leaders: Creating change for people, processes and paradigms*, Routledge, New York, NY, USA, **2017**, pp. 44-64.
81. Roberts, L.M.; Creary, S.J. Positive identity construction: Insights from classical and contemporary theoretical perspectives. In *The Oxford Handbook of Positive Organizational Scholarship*; Cameron, K.S., Spreitzer, G., Eds.; Oxford University Press: New York, NY, USA, **2011**; pp. 70–83.
82. Snook, S.; Ibarra, H.; Ramo, L. *Identity-Based Leader Development*; Harvard University Press: Boston, MA, USA, **2010**.
83. Creary, S.J.; Roberts, L.M. G.I.V.E.-based mentoring in diverse organizations: Cultivating positive identities in diverse leaders. In., Murrell, A.J. & Blake-Beard, *Mentoring diverse leaders: Creating change for people, processes and paradigms*, Routledge, New York, NY, USA, **2017**, pp. 3-24.
84. Randel, A.E.; Galvin, B.M.; Gibson, C.B.; Batts, S.I. Increasing career advancement opportunities through sponsorship: An identity-based model with illustrative application to cross-race mentorship of African Americans. *Group Organ. Manag.* **2021**, *46*, 105–142, doi:10.1177/1059601120978003.
85. Dutton, J. E., & Heaphy, E. D. The power of high-quality connections. *Positive organizational scholarship: Foundations of a new discipline*; Berrett-Koehler Publishers: Oakland, CA, USA, 2003, *3*, 263–278.
86. Ibarra, H. Homophily and differential returns: Sex differences in network structure and access in an advertising firm. *Admin. Sci. Quart*. **1992,** 422–447.
87. Hewlett, S. A., Peraino, K., Sherbin, L., & Sumberg, K. *The sponsor effect: Breaking through the last glass ceiling*. *Harvard Business Review*: Boston, MA, USA, **2010**.
88. Nembhard, I.M.; Edmondson, A.C. Making it safe: The effects of leader inclusiveness and professional status on psychological safety and improvement efforts in health care teams. *J. Organ. Behav.* **2006**, *27*, 941–966, doi:10.1002/job.413.
89. Bilimoria, D. Modernism, postmodernism, and contemporary grading practices. *J. Manag. Educ.* **1995**, *19*, 440–457, doi:10.1177/105256299501900403.
90. Sugiyama, K.; Cavanagh, K.V.; Van Esch, C.; Bilimoria, D.; Brown, C. Inclusive leadership development. *J. Manag. Educ.* **2016**, *40*, 253–292, doi:10.1177/1052562916632553.
91. Vinnicombe, S.; Singh, V. Women-only management training: An essential part of women's leadership development. *J. Chang. Manag.* **2002**, *3*, 294–306, doi:10.1080/714023846.
92. Ely, R. J., & Meyerson, D. E. Advancing gender equity in organizations: The challenge and importance of maintaining a gender narrative. *Organ.* **2000**, *7*(4), 589–608.
93. Stockdale, M.S., Chrobot-Mann, D.M., Chance, R.C. & Crosby, F.J. Peer mentoring retreats for addressing dilemmas of senior women in STEM careers: The Nag's Heart Model. In., Murrell, A.J. & Blake-Beard, *Mentoring diverse leaders: Creating change for people, processes and paradigms*, Routledge, New York, NY, USA, **2017**, pp. 218-234.
94. Kahn, W. A. Holding environments at work. *The Jnl of App. Beh. Sci.* **2001**, 37(3), 260–279.
95. Zagenczyk, T.J.; Gibney, R.; Kiewitz, C.; Restubog, S.L.D. Mentors, supervisors and role models: Do they reduce the effects of psychological contract breach? *Hum. Resour. Manag. J.* **2009**, *19*, 237–259, doi:10.1111/j.1748-8583.2009.00097.x.
96. Ghosh, R., Hayes, R. K., & Kram, K. E. Developmental networks as holding environments for growing leaders: An integrative perspective. *Acad. of Manag. Conf. Proceed*, **2010**.
97. Giscombe, K. Creating effective mentoring programs for women of color. In., Murrell, A.J. & Blake-Beard, *Mentoring diverse leaders: Creating change for people, processes and paradigms*, Routledge, New York, NY, USA, **2017**, pp. 145-158.
98. Chandler DE, Kram KE, Yip J. An ecological systems perspective on mentoring at work: A review and future prospects. *The Acad. of Manag. Ann*. **2011** *Jun 1;5(1)*:519-70.
99. Ragins, B. R., & McFarlin, D. B. Perceptions of mentor roles in cross-gender mentoring relationships. *Jnl of Voc. Beh.* **1990**, *37*(3), 321–339.
100. Thomas, D.A. Beyond the simple demography-power hypothesis: How blacks in power influence white-mentor black-protégé developmental relationships. In A. Murrell, F. Crosby & R. Ely (Eds.), *Mentoring dilemmas: Developmental relationships within multicultural organizations*, Psychology Press: London, UK, 1999, pp. 157–170.
101. Davidson, M.N. *The End of Diversity as We Know It: Why Diversity Efforts Fail and How Leveraging Difference Can Succeed*; Berrett-Koehler Publishers: Oakland, CA, USA, **2011**.
102. Dovidio, J.F.; Gaertner, S.L.; Saguy, T. Commonality and the complexity of "we": Social attitudes and social change. *Personal. Soc. Psychol. Rev.* **2009**, *13*, 3–20.

103. Ragins, B.R.; Cotton, J.L. Mentor functions and outcomes: A comparison of men and women in formal and informal mentoring relationships. *J. Appl. Psychol.* **1999**, *84*, 529–550, doi:10.1037/0021-9010.84.4.529.
104. Ragins, B.R.; Kram, K.E. *The Handbook of Mentoring at Work: Theory, Research, and Practice*; SAGE Publications: Thousand Oaks, CA, USA, **2008**.
105. Weaver, G.R.; Treviño, L.K.; Agle, B. "Somebody I look up to:": Ethical role models in organizations. *Organ. Dyn.* **2005**, *34*, 313–330.
106. Viator, R.E. An analysis of formal mentoring programs and perceived barriers to obtaining a mentor at large public accounting firms. *Account. Horiz.* **1999**, *13*, 37–53, doi:10.2308/acch.1999.13.1.37.
