# Supplementary material for: Estimation of Undetected Asymptomatic COVID-19 Cases in South Korea Using a Probabilistic Model
Source: Int J Environ Res Public Health. 2021 May 6;18(9):4946. doi: 10.3390/ijerph18094946 (PMC8124955; doi:10.3390/ijerph18094946)
Supplement: Supplementary file 1 [file ijerph-18-04946-s001.zip › 5/ijerph-1187074 - update.pdf]

International Journal of
Environmental Research and Public Health

MDPI

Article

Mentoring as a Buffer for the Syndemic Impact of Racism and COVID-19 among Diverse Faculty within Academic Medicine

Jeannette E. South-Paul [1,*], Kendall M. Campbell [2], Norma Poll-Hunter [3] and Audrey J. Murrell [4]

[1] School of Medicine, University of Pittsburgh, Pittsburgh, PA 15261, USA
[2] Research Group for Underrepresented Minorities in Academic Medicine, Brody School of Medicine, Greenville, NC 27834, USA; campbellke16@ecu.edu
[3] Association of American Medical Colleges, Washington, DC 20001, USA; npoll@aamc.org
[4] School of Business, University of Pittsburgh, Pittsburgh, PA 15260, USA; amurrell@pitt.edu
* Correspondence: jsouthpaul@outlook.com

Abstract: Within this article, we explore the dual impact of two pandemics, racism and COVID-19, on the career and psychological well-being of diverse faculty within academic medicine. First, we present a discussion of the history of racism in academic medicine and the intensification of racial disparities due to the COVID-19 pandemic. As a result of the syndemic of racism and COVID-19, the outlook for the recruitment, retention, and advancement of diverse faculty and leaders within academic medicine is at risk. While mentoring is known to have benefits for career and personal development, we focus on the unique and often unacknowledged role that mentoring can play as a buffer for women and people of color, especially when working in institutions that lack diversity and are now struggling with the syndemic of racism and COVID-19. We also discuss the implications of acknowledging mentoring as a buffer for future leadership development, research, and programs within academic medicine and health professions.

Keywords: mentoring; diversity; racism; academic medicine; COVID-19

Citation: South-Paul, J.E.; Campbell, K.M.; Poll-Hunter, N.; Murrell, A.J. Mentoring as a Buffer for the Syndemic Impact of Racism and COVID-19 among Diverse Faculty within Academic Medicine. *Int. J. Environ. Res. Public Health* **2021**, *18*, 4921. https://doi.org/10.3390/ijerph18094921

Academic Editor: Paul B. Tchounwou

Received: 31 March 2021
Accepted: 29 April 2021
Published: 5 May 2021

Publisher's Note: MDPI stays neutral with regard to jurisdictional claims in published maps and institutional affiliations.

Copyright: © 2021 by the authors. Licensee MDPI, Basel, Switzerland. This article is an open access article distributed under the terms and conditions of the Creative Commons Attribution (CC BY) license (http://creativecommons.org/licenses/by/4.0/).

1. Mentoring as a Buffer for Diverse Leaders within Academic Medicine

As continual efforts are made to expand the diversity of the academic medicine workforce, health centers, accrediting bodies, medical societies, and others have established equity, diversity, inclusion, and anti-racism as priorities. It is clear that, while the need is great, the complexity of the challenges is also significant. This complexity means that equity, diversity, and inclusion work must be defined as multidimensional. Needs span from dismantling racist and privilege systems, to providing mentorship and development opportunities for those in training, to developing pathway programs, to addressing historical injustice, to adjusting to ongoing societal changes and challenges. It is important to acknowledge that academic medicine did not arrive at the place of a lack of diversity in leadership by chance. Rather, the historical disparities in workforce development, leadership advancement, and healthcare delivery are deliberate and based upon our nation's history of racism, sexism, and discrimination. The need for actions that can address these historic barriers is of critical importance. Thus, we make the unique case that mentoring is a powerful solution that not only supports diversity in workforce and leadership development, but offers a buffer for the negative impact of these persistent disparities.

As such, we explore the dual impact of two pandemics, that is, the syndemic of racism and COVID-19, on the need and complexity of developing diverse leaders within academic medicine. Leaders not only provide guidance for those who are following them into the professions they model, but also are critical to shaping the institutional structures

themselves. We offer mentoring as a solution to the existing challenges of a lack of diversity within academic medicine and the unique challenges for underrepresented racial and ethnic minority (URM) faculty. Underrepresented racial and ethnic minority communities include individuals who are Black or African American, Latinx (including those who identify as Hispanic or Latino), American Indian or Alaska Native, and Native Hawaiian and Other Pacific Islander. While mentoring has been shown to have benefits for career and personal development, we focus on the unique and often unacknowledged role that mentoring plays as a buffer for women and people of color, particularly for institutions that lack diversity. The unique challenges caused by this syndemic make the need for organization-sponsored programming even more urgent within academic medicine and health professions.

2. Challenges of the Racism Pandemic

Racism is a foundation of discrimination, and manifests through the enablement and propagation of systems that advantage some and disadvantage others [1]. This includes aversive racism, defined as a subtle, indirect, and unconscious form of bias, but it is also conceptualized as the rationalization of discriminatory actions by majority individuals who see themselves as fair and egalitarian [2]. Recent attention has also focused on the negative impact of racialized trauma that is specific to and a consequence of anti-black racism [3]. The impact of racism in combination with persistent sexism has produced a double impact for black women in academic medicine [4]. Racism, which is now considered a public health crisis, has influenced the focus of education and training, how populations are viewed and treated, and policies and practices, and thus has facilitated health disparities [5–7]. Academic medicine is not immune to acts of racism, historically and currently, and these acts influence the development of a diverse workforce. Historians attribute some of the disproportionately low numbers of Black physicians in US medical schools to the early twentieth century Flexner Report that laid the framework for modern medical education. The education specialist selected by the Carnegie Foundation and the American Medical Association (AMA), Abraham Flexner, reviewed all 155 medical schools in the US and Canada and published the findings in 1910 that provided criteria to standardize and improve medical schools [8].

However, these earlier recommendations resulted in the closing of many under-resourced institutions. Only 66 medical schools remained by 1923, as well as only two of seven existing Black medical schools. African Americans were 2.5% of US physicians in 1910, but had decreased to 2.2% in 2008 before increasing to approximately 5% of the workforce today [9]. In the early to mid-1960′s, federal policies were initiated to address health profession shortages to include the passage of the Health Professions Educational Assistance Act of 1963 [10]. As part of the Comprehensive Health Manpower Training Act of 1971, the Special Health Careers Opportunity Grant (SHCOG) Program (later to become the Health Careers Opportunity Program) was implemented in 1972 [11]. While the Health Career Opportunity Programs (HCOPs) have been a major catalyst for increasing diversity in the health professions, the impact has been slow, especially in diversifying leadership roles within academic medicine and health professions.

Within medicine, Nickens (1994) delineated efforts to increase diversity in three phases: Phase 1 as reflective of the social activism in the late 1960′s to mid-1970′s; Phase 2 from 1974–1990, described as a period of stagnation; and Phase 3, starting with a call to action marked by the Association of American Medical Colleges (AAMC) national initiative in 1990 called Project 3000 by 2000. Project 3000 by 2000 created a target to matriculate 3000 underrepresented minority medical students by the year 2000 through efforts promoted by the AAMC and implemented by individual medical schools in their communities. However, many efforts in the 1990′s were thwarted by aggressive campaigns to discredit and dismantle affirmative action state by state. There was then a resultant plunge in the modest gains in diversity that had been achieved in prior years. More recent calls for diversity are focused on the multiple benefits derived from having diverse classrooms

and working environments [12–17]. This includes research focused on physician workforce diversity that is associated with increased access to care, in particular, for underserved communities, increased patient adherence to treatment, and patient satisfaction with care [18–22].

3. Challenges of the COVID-19 Pandemic

History provides perspective, but the current urgency for increasing the representation of Black/African American, American Indian and Alaska Native, and Latinx physicians and scientists is underscored by the recent COVID-19 pandemic. Although broad segments of the nation have experienced COVID-19 cases, racial/ethnic minority communities have been devastated by this pandemic: more than 50 percent of those hospitalized and a significant percent of those requiring intensive care have been people of color [23]. Defeating SARS-CoV-2 requires the most diverse group of intellectuals and clinicians to effect change [24]. Furthermore, the pandemic of anti-Black violence has gripped dozens of communities inciting collective action to seek racial justice, particularly the availability of medical treatments, vaccines, and culturally sensitive approaches to engaging communities of color.

The effects of COVID-19 on health profession education have not been as widely shared in the literature. It is likely that these effects will become even more evident as the vaccination process is implemented and there is better virus control. COVID-related United States Medical Licensure (USMLE) exam testing irregularities, including cancellations, closed facilities, and poor communication, have imposed additional unwanted stressors on many students before and during testing. Major problems have occurred related to the three-step process for medical students to apply and prepare for the first USMLE examination, including changing testing dates and adjustments of arrangements to travel to distant sites to sit for the exam. In addition, as medical schools have shifted curricula to balance curricular learning objectives, and the AAMC has made suggestions for keeping learners safe while meeting learning objectives, students have faced rapidly changing, unpredictable schedules filled with uncertainty. Lastly, the urgencies of managing the health, workforce, and economics of the COVID pandemic on health systems and communities have reduced the focus of senior leadership on the needs of URM faculty and learners, contributing to the persistence of healthcare inequities and distrust of the medical profession [25].

These complex and devasting factors call for greater attention to diversity and inclusion to address the impact of the COVID-19 pandemic not only within healthcare systems, but also within the development of diverse providers and leaders within academic medicine. This need is underscored by multiple studies documenting the extent of distrust of the healthcare establishment in general and persistent healthcare disparities. The value of having more diverse clinicians to help reveal the complexities of this pandemic, as well as to care for the population disproportionately affected, cannot be questioned [26].

4. The Need for URM Faculty and Leaders within Academic Medicine

Both system-wide and organizational level interventions are needed to increase the numbers of URM faculty in medicine and especially within leadership ranks. While our focus is primarily on cultivating diverse leaders within academic medicine, this cannot be examined independently from addressing the issues of diversity with academic medicine faculty as the pipeline to leadership ranks. The presence of a diverse faculty and leaders is critical for advancing medical education, research, and clinical care. However, continued efforts are necessary to increase faculty and leadership diversity in academic medicine [27]. For example, in 2003, faculty who were reported as Black/African American, American Indian or Alaska Native, Native Hawaiian or Pacific Islander, or Hispanic/Latino alone represented 7% of full-time faculty at MD-granting institutions [28]. Despite the growth of medical schools, they only represent 7.3% of full-time faculty in 2020. URM faculty are also less likely to be represented at the associate and professor levels compared

to their White counterparts [29]. Among the permanent, interim, or acting department chairs at MD-granting institutions in 2020, 4% were reported as Black/African American, 3.5% as Hispanic/Latino, 0.1% as American Indian and Alaska Native, and 0.06% as Native Hawaiian or Other Pacific Islander. When removing the four HBCU medical schools and the four medical schools in Puerto Rico, the data on URM representation is even more stark [30,31].

Overall, the lack of diversity across faculty ranks and leadership threatens our ability to create more inclusive education, enriched learning, and training environments [32]. For example, faculty diversity within higher education has been associated with exposure to a broader range of teaching strategies to include more interactive, cooperative pedagogy with less lecture-based learning, and the inclusion of curricular content focused on racial and ethnic or gender issues [33]. Faculty of color were reported to interact more frequently with students compared to White faculty, and increased faculty diversity contributed to greater use of effective educational practices [34]. More recent data also show that faculty diversity was related to increased graduation rates among underrepresented minority students at the baccalaureate level.

A residency program's ability to attract diverse resident physicians may also be influenced by the extent of its program diversity. One study found that resident and faculty URM representations were correlated [35]. In another study, women who attended medical school with higher numbers of orthopedic female faculty and residents were more likely to apply for a residency in orthopedics—a specialty that has had an extremely low representation of women [36]. While recruitment efforts have shown modest benefits, the use of formal mentoring programs has consistently shown a positive impact on a range of diversity and organizational outcomes [37].

Despite the benefits of faculty diversity in the classroom and in clinical training, the representation, progress, and advancement of URM faculty in medicine has been encumbered by several factors, including limited access to mentors, sponsors, and role models [38]; reported experiences of discrimination [39]; and the isolation and absence of inclusive academic cultures [40]. In addition, they are often burdened by requests to serve in other roles to support diversity-related initiatives. Often as one of few URM faculty, they may struggle with their orientation to serve and the lack of formal recognition within the promotion process for these efforts. These negative experiences also influence the recruitment and advancement of URM faculty in academic medicine [41]. Research shows that racial and ethnic minority faculty at the assistant and associate levels are more likely to leave academic medicine compared to their White counterparts, and to leave sooner, limiting their access to leadership ranks [42].

To address the unique experiences of racial and ethnic minority faculty, academic health centers have developed a range of programs to support professional and career development [43]. Interventions to help racial and ethnic minority faculty navigate and overcome gratitude and minority taxes to promote their recruitment and retention in healthcare are essential for diversity and inclusion efforts. While not promoted into leadership ranks, the gratitude tax is manifested as a loyalty of URM faculty to their institution, where they may be undervalued and underappreciated [44]. This loyalty may cause them to forego a promotion or advancement at another institution, because they are thankful to have a job at their current institution [45]. The minority tax includes racism, lack of faculty development, lack of mentorship, isolation, and an often solitary focus on disparities without peer support. This tax contributes to racial and ethnic minority faculty leaving academic medicine. To address these challenges, and to achieve needed interventions, mentorship and sponsorship are needed to assure that diverse academic leaders are realizing their full potential in a place where they can thrive [46].

5. The Importance of Mentoring within Academic Medicine

Mentoring within medical and healthcare professions has been shown to impact career choice, personal development, research productivity, and career advancement [47]. There is little debate over the importance of mentoring programs within academic medicine; however, questions remain over the most effective aspects of program design, program delivery, and evaluation metrics [48]. Most systematic reviews of the existing literature find that mentoring produces faculty who are more productive, promoted more quickly, and more likely to stay in their academic institutions than those who do not have access or choose not to participate in formal mentoring efforts [49]. A majority of formal programs within academic medicine tend to take the form of traditional one-to-one mentoring. However, some researchers argue that the decline in the number and diversity of clinical and research faculty causes a significant reduction of the available pool of senior mentors that are needed using this traditional approach [50]. Few early career faculties will have access to a senior mentor for one-to-one matching within a formal program, and a lack of willingness may further limit access to informal mentoring relationships. Diverse senior mentors are an extremely scarce resource, and expectations for these individuals to be involved in multiple formal and informal mentoring relationships can produce an unfair burden or "mentoring tax" for these faculty members and clinicians [51].

While diverse mentoring relationships (e.g., cross race, cross-gender, etc.) within a single organization may have many advantages that are critical for individual and organizational outcomes, the reality is that these relationships are complex, more likely to produce conflict, and may not meet all of the needs for women and people of color within organizations, particularly those seeking to "break through" to senior level leadership positions [52]. This is especially true if mentoring relationships (both formal and informal) do not explicitly address racial, gender, and other identity issues or engage in identity work as part of the mentoring or sponsorship relationship. Identity work has been described as the process by which leaders engage in forming, repairing, maintaining, and strengthening or revising their personal, social, and work identities. Mentoring relationships have been shown to provide legitimacy for reconciling various identities as a mentor or sponsor, and can provide validation and legitimation for diverse leaders [53]. This notion is supported by a wide variety of empirical and theoretical work that shows race, as one aspect of diversity, to be embedded within the organizational context, a consistent driver of work attitudes and outcomes, and a moderator of the return on investment employees receive from training and other developmental activities [54]. Thus, attention should be devoted to how the experience of mentoring relationships shapes our ability to develop a diverse cadre of leaders who can create meaningful change and cultivate diversity, especially when mentoring or sponsorship involves cross-race relationships [55]. Because of the complexities, some argue that to advance women and people of color within organizations, more attention needs to focus on developing a diverse portfolio of mentoring programs, especially that develop leaders within academic medicine [56].

6. The Mentoring as a Buffer Paradigm

While mentoring has been well-documented to support career advancement and social support, more recent work has examined the "mentor as a buffer" hypothesis. There is the notion that mentoring can serve as a buffer, especially for the negative effects of discriminatory workplace practices and cultures [57]. This paradigm argues that high-quality mentoring relationships not only provide support, but help mentees cope with the negative impact of discrimination within the workplace, a lack of an inclusive workplace culture, and negative effects of abusive supervisors [58]. This is based on the notion that mentoring relationships provide what is called "holding behavior", in that they function to buffer mentees from stressful, negative, and detrimental experiences. This buffering effect means that negative experiences do not derail the careers of diverse leadership or

reduce the negative impact on feelings of psychological safety, organizational commitment, and perceptions of organizational support. This buffering effect is especially important in situations of both blatant and subtle racism, sexism, and other forms of discrimination. In fact, research acknowledges that women and people of color can experience ambient discrimination, which is the knowledge or awareness of discrimination in the workplace that is aimed at others, but has a negative impact on those with similar social identity membership [59]. This is often likened to the negative effects of second-hand smoke on non-smokers who are in close proximity to these toxic fumes.

Mentoring as a buffer to experienced and/or ambient discrimination or harassment can offset the negative impact in a variety of different ways by various mentors, including senior leaders and peers. For example, mentors can provide containment by creating a safe space and protection, and serve as an advocate for mentees to offset the impact of non-inclusive cultures and discriminatory actions. Mentoring relationships such as those provided by peers can be a source of empathy that provides much needed confirmation and validation for subtle forms of discrimination, harassment, and microaggressions. Organization-sponsored mentoring programs that provide senior role models can serve as a buffer by sharing their own experiences and insights and enabling perspectives that can help mentees make sense out of negative experiences and discrimination and navigate the environment.

This idea of mentoring as a buffer has been shown by a number of research studies that look at the notion of a psychological contract breach. The concept of the psychological contract involves an individual's perception of the reciprocal relationship that exists between them and the organization [60]. This research shows that mentors are able to help individuals to recognize and interpret changes, or what the research calls a "breach" in the psychological contract. These breaches in the psychological contract are known to have a negative impact on overall job satisfaction, commitment to the organizations, and retention [61]. While having a mentor does not guarantee that all promises by an organization will be kept, mentors can help with the recognition, interpretation, and coping behaviors that result as a function of a breach. Diverse leaders can experience challenges in having their identities validated and respected by others who view them as different from or in conflict with traditional definitions and expectations of leadership based on the organization's history, its culture, and societal norms. One study on inter-organizational formal mentoring found that using a combination of identity-based leadership development that includes senior and peer mentoring is effective at buffering the impact of negative workplace environments, non-supportive supervisors, and perceived breaches in the psychological contract [62].

This is especially important for URM leaders, who are often the target of discrimination or can be negatively impacted by ambient discrimination. In fact, recent research has shown that mentors and role models can reduce the negative impact of a psychological contract breach more effectively than formal supervisorial relationships, which often contribute to perceived inequalities at work [63]. This suggests that mentoring relationships are not only essential for helping to buffer the negative impact of direct experiences within the workplace, but can help mentees to understand, interpret, and cope with ambient discriminatory experiences that take place within the organization, profession, and, as we have witnessed by the racism pandemic, throughout the world. This buffering effect is especially important as URM leaders cope with the disproportionately negative impact of these dual pandemics (racism and COVID-19) or what is now called a syndemic [64]. Thus, the need for organization-sponsored mentoring that provides both direct and indirect benefits of different mentoring relationships (e.g., hierarchical, peer, or group mentoring), especially for diverse leadership, is both impactful and necessary for developing diverse leaders within academic medicine [65].

7. Mentoring Programs for Diverse Leaders within Academic Medicine

The multiple challenges presented in 2020—primarily centered around the dual pandemics of COVID-19 and the visible incidences of racial injustice and subsequent protests and insurrection at the capital—have impacted institutions of higher education, as well as every other community. Face-to-face communications have been replaced by multiple virtual platforms. Telemedicine has provided an effective technique for managing many healthcare encounters. Undergraduate medical education has likewise embraced Zoom, MS Teams, Webex, and Google Hangouts to deliver not only what had been delivered traditionally as a lecture, but also small group discussions.

Structured mentoring and career development programs that are embedded in institutional structures result in measurable increases in retention and advancement of young academics [66–68]. The unevenness of mentoring and faculty development programs for URM faculty across academic health centers has resulted in the development of important national programs. We highlight three examples of federal, philanthropic, and organization-led efforts that have longstanding track records in addressing faculty diversity in academic health centers. The federal initiatives supported via Titles VII and VIII education and training programs of the Public Health Service Act (42 USC 292 et seq.) provide financial support to advance faculty diversity through two primary mechanisms: (1) the Centers of Excellence, with a focus on racial and ethnic minority faculty recruitment and leadership development, and (2) the Faculty Loan Repayment, which provides racial and ethnic minority medical school faculty with funds to repay qualifying educational loans. The Centers of Excellence programs imbed one-on-one mentoring for participating scholars, as well as group activities to provide career support.

In 1983, the Robert Wood Johnson Foundation launched the Harold Amos Medical Faculty Development Program (AMFD) to mentor "individuals from historically disadvantaged and underrepresented backgrounds" and to advance as faculty and in leadership in academic health centers. The program is comprised of an annual stipend and a grant to support protected time for research and mentorship by a senior faculty member. The results over 30 years show that, of the nearly 300 participants, many have remained in academic medicine, holding positions as full professors, chairs of departments, leaders within the National Institutes of Health, and national and international leaders in research (https://www.amfdp.org/about/history (accessed on 2 May 2021).

Recognizing the uneven availability of faculty development programs across all medical institutions, the Association of American Medical Colleges initiated what was named at the time the Minority Faculty Career Development Seminar to address issues related to URM faculty retention and advancement in 1990. The three-day seminar brings together diverse senior faculty across the US to deliver culturally responsive workshops to URM early career faculty focused on strategies for career and leadership development. The seminar culture is about fostering a sense of belongingness in academic medicine through networking with peers, guidance tailored to the unique experiences of URM faculty, and access to diverse senior leaders. To date, the program has served about 2500 diverse faculty. These programs that have continued after 30 years signify the longstanding need for programs and resources to advance faculty diversity in health profession education.

8. Conclusions

It is important to acknowledge that the nation did not arrive at the place of physician workforce shortages by chance, and the previous and current health and healthcare disparities are the result of persistent acts of racism, sexism, and other forms of discrimination that occur at the individual, interpersonal, institutional, and societal levels. From the culmination of the Civil War, the victorious General William T. Sherman proposed dividing up the huge plantations of southeastern Georgia to grant each family of color "40 acres and a mule." However, the proposal faced substantial political opposition, and was never

widely adopted. It was almost a century later that this concept was again examined through a series of court decisions that interpreted the civil rights guarantees within the Equal Protection Clause of the Fourteenth Amendment—initiatives that became known as affirmative action. Supreme Court Justice William Brennan noted that the mandatory and voluntary programs that were now being supported would help to eliminate the persisting effects of discrimination [69]. These historic and systemic racist actions continue to influence every aspect of American society, and require purposeful intervention.

Academic health centers and national organizations are responding to the syndemic by issuing statements and creating structures that advance diversity, equity, inclusion, and now anti-racism. For example, the AAMC released an anti-racism framework that aspires to foster change at multiple levels: the individual through self-reflection, internal efforts at the AAMC, efforts at academic health centers, and through the engagement of the broader community. While these national and institutional efforts take hold, mentoring as a well-accepted and validated practice to support faculty advancement can be reimagined within the framework of "mentor as a buffer" to mitigate the adverse impacts of past, current, and future crises. Expanding institution-supported mentoring programs as part of a standardized solution offers great promise in advancing URM faculty success and thriving for all faculty in academic medicine [70].

Author Contributions: Conceptualization, J.E.S.-P., K.M.C., N.P.-H. and A.J.M.; investigation, J.E.S.-P., K.M.C., N.P.-H. and A.J.M.; resources, J.E.S.-P., K.M.C., N.P.-H. and A.J.M.; writing—original draft preparation, J.E.S.-P., K.M.C., N.P.-H. and A.J.M.; writing—review and editing, J.E.S.-P., K.M.C., N.P.-H. and A.J.M.; supervision, J.E.S.-P.; project administration, A.J.M. All authors have read and agreed to the published version of the manuscript.

Funding: This research received no external funding.

Institutional Review Board Statement: Not applicable.

Informed Consent Statement: Not applicable.

Conflicts of Interest: The authors declare no conflict of interest.

References

1. Jones, C.P. Toward the Science and Practice of Anti-Racism: Launching a National Campaign Against Racism. *Ethn Dis*. **2018**, *28*, 231–234, doi:10.18865/ed.28.S1.231.
2. Murrell, A.J. Aversive Racism: Foundations, Impact, and Future Directions. *Oxf. Res. Encycl. Bus. Manag.* **2020**, doi:10.1093/acrefore/9780190224851.013.194.
3. Mosley, D.V.; Hargons, C.N.; Meiller, C.; Angyal, B.; Wheeler, P.; Davis, C.; Stevens-Watkins, D. Critical consciousness of anti-Black racism: A practical model to prevent and resist racial trauma. *J. Couns. Psychol.* **2021**, *68*, 1.
4. Balzora, S. When the minority tax is doubled: Being Black and female in academic medicine. *Nat. Rev. Gastroenterol. Hepatol.* **2020**, *18*, 1.
5. Devakumar, D.; Selvarajah, S.; Shannon, G.; Muraya, K.; Lasoye, S.; Corona, S.; Achiume, E.T. Racism, the public health crisis we can no longer ignore. *Lancet* **2020**, *395*, e112–e113, doi:10.1016/S0140-6736(20)31371-4.
6. Kuehn, B.M. AHA Takes Aim at Structural Racism as a Public Health Crisis. *Circulation* **2021**, *143*, 501–502, doi:10.1161/CIRCULATIONAHA.120.053306
7. Hlavinka, E. *Racial Bias in Flexner Report Permeates Medical Education Today*; MedPage Today. 18 June 2020. Available online: https://www.medpagetoday.com/publichealthpolicy/medicaleducation/87171 (accessed on 2 May 2021).
8. Campbell, K.M.; Corral, I.; Linares J.L.I.; Tumin, D. Projected Estimates of African American Medical Graduates of Closed Historically Black Medical Schools. *JAMA Netw. Open*. **2020**, *3*, e2015220, doi:10.1001/jamanetworkopen.2020.15220.
9. Testoff, A.; Aronoff, R. The Health Careers Opportunity Program: One influence on increasing the number of minority students in schools of health professions. *Public Health Rep.* **1983**, *98*, 284.
10. Sullivan, L.W.; Mittman, I.S. The state of diversity in the health professions a century after Flexner. *Acad. Med.* **2010**, *85*, 246–253.
11. Hurtado, S. Linking diversity with the educational and civic missions of higher education. *Rev. High. Educ.* **2007**, *30*, 185–196.
12. Gurin, P.; Nagda, B.R.A.; Lopez, G.E. The benefits of diversity in education for democratic citizenship. *J. Soc. Issues* **2004**, *60*, 17–34.
13. American Council on Education; American Association of University Professors. *Does Diversity Make a Difference? Three Research Studies on Diversity in College Classrooms*; American Council on Education: Washington, DC, USA; American Association of University Professors: Washington, DC, USA, 2000.

14. Milem, J. Increasing Diversity Benefits: How Campus Climate and Teaching Methods Affect Student Outcomes. In *Diversity Challenged: Evidence on the Impact of Affirmative Action*; Orfield, G., Ed.; Harvard Education Publishing Group: Cambridge, UK, 2001; pp. 233–249.
15. Whitla, D.K.; Orfield, G.; Silen, W.; Teperow, C.; Howard, C.; Reede, J. Educational benefits of diversity in medical school: A survey of students. *Acad Med*. **2003**, *78*, 460–466.
16. Stahl, G.K.; Maznevski, M.L.; Voigt, A.; Jonsen, K. Unraveling the effects of cultural diversity in teams: A meta-analysis of research on multicultural work groups. *J. Int. Bus Stud.* **2010**, *41*, 690–709.
17. Greenwood, B.N.; Hardeman, R.R.; Huang, L.; Sojourner, A. Physician–patient racial concordance and disparities in birthing mortality for newborns. *Proc. Natl. Acad. Sci. USA* **2020**, *117*, 21194–21200.
18. Alsan, M.; Garrick, O.; Graziani, G. Does diversity matter for health? Experimental evidence from Oakland. *Am. Econ. Rev.* **2019**, *109*, 4071–4111.
19. Marrast, L.M.; Zallman, L.; Woolhandler, S.; Bor, D.H.; McCormick, D. Minority physicians' role in the care of underserved patients: Diversifying the physician workforce may be key in addressing health disparities. *JAMA Intern. Med.* **2014**, *174*, 289–291.
20. LaVeist, T.A.; Nuru-Jeter, A.; Jones, K.E. The association of doctor-patient race concordance with health services utilization. *J. Public Health Policy* **2003**, *24*, 312–323.
21. Xierali, I.M.; Castillo-Page, L.; Conrad, S.; Nivet, M.A. Analyzing physician workforce racial and ethnic composition associations: Geographic distribution (Part II). *Anal. Brief*. **2014**, *e14*, 9.
22. National Center for Immunization and Respiratory Diseases (NCIRD), Division of Viral Diseases. COVID-19 Racial and Ethnic Health Disparities. Available online: https://www.cdc.gov/coronavirus/2019-ncov/community/health-equity/racial-ethnic-disparities/disparities-hospitalization.html (accessed on 30 April 2021).
23. Crumb, L.; Campbell, K.M.; Crowe, A.; Harris, J.A.; Acheampong, C.; Little, J. Responding to COVID-19: Perspectives on Curricular Changes in a Rural Medical School. *South Med. J.* **2020**, *113*, 368–371, doi:10.14423/SMJ.0000000000001124.
24. Hudson, B.; Campbell, K.M. Does Criticism of Minority Faculty Result from a Lack of Senior Leadership Training and Accountability? *Acad. Med*. **2020**, *95*, 1792, doi:10.1097/ACM.0000000000003735.
25. Lucey, C.R.; Johnston, S.C. The transformational effects of COVID-19 on medical education. *JAMA* **2020**, *324*, 1033–1034.
26. Mader, E.M.; Rodríguez, J.E.; Campbell, K.M.; Smilnak, T.; Bazemore, A.W.; Petterson, S.; Morley, C.P. Status of underrepresented minority and female faculty at medical schools located within Historically Black Colleges and in Puerto Rico. *Med Educ. Online* **2016**, *21*, 29535.
27. Association of American Medical Colleges. U.S. Medical School Faculty by Gender and Race and Ethnicity, 2003–2007. In Diversity in Medical Education: Facts and Figures 2008. Available online: https://www.aamc.org/media/7581/download (accessed on 30 April 2021).
28. Fisher, A.J.; Mendoza-Denton, R.; Patt, C.; Young, I.; Eppig, A.; Garrell, R.L.; Richards, M.A. Structure and belonging: Pathways to success for underrepresented minority and women PhD students in STEM fields. *PLoS ONE* **2019**, *14*, e0209279.
29. Stout, R.; Archie, C.; Cross, D.; Carman, C.A. The relationship between faculty diversity and graduation rates in higher education. *Intercult. Educ.* **2018**, *29*, 399–417.
30. Umbach, P.D. The Contribution of Faculty of Color to Undergraduate Education. *Res. High Educ.* **2006**, *47*, 317–345.
31. Hurtado, S. Linking Diversity and Educational Purpose: How Diversity Affects the Classroom Environment and Student Development. In *Diversity Challenged: Evidence on the Impact of Affirmative Action*; Orfield, G., Ed.; Harvard Education Publishing Group: Cambridge, MA, USA, 2001; pp. 187–203.
32. Rodríguez, J.E.; López, I.A.; Campbell, K.M.; Dutton, M. The Role of Historically Black College and University Medical Schools in Academic Medicine. *J. Health Care Poor Underserved*. **2017**, *28*, 266–278, doi:10.1353/hpu.2017.0022.
33. Ku, M.C.; Li, Y.E.; Prober, C.; Valantine, H.; Girod, S.C. Decisions, decisions: how program diversity influences residency program choice. *J. Am. Coll. Surg.* **2011**, *213*, 294–305.
34. Phitayakorn, R.; Macklin, E.A.; Goldsmith, J.; Weinstein, D.F. Applicants' self-reported priorities in selecting a residency program. *J. Grad. Med Educ.* **2015**, *7*, 21.
35. Agawu, A.; Fahl, C.; Alexis, D.; Diaz, T.; Harris, D.; Harris, M.C.; Higginbotham, E.J. The influence of gender and underrepresented minority status on medical student ranking of residency programs. *J. Natl. Med Assoc.* **2019**, *111*, 665–673.
36. Aagaard, E.M.; Julian, K.; Dedier, J.; Soloman, I.; Tillisch, J.; Perez-Stable, E.J. Factors affecting medical students' selection of an internal medicine residency program. *J. Natl. Med. Assoc.* **2005**, *97*, 1264.
37. Fairmont, I.; Farrell, N.; Johnson, A.P.; Cabrera-Muffly, C. Influence of gender and racial diversity on the otolaryngology residency match. *Otolaryngol. Head Neck Surg.* **2020**, *162*, 290–295.
38. Yousuf, S.J.; Kwagyan, J.; Jones, L.S. Applicants' choice of an ophthalmology residency program. *Ophthalmology* **2013**, *120*, 423–427.
39. Loes, C.N.; Culver, K.C.; Trolian, T.L. How collaborative learning enhances students' openness to diversity. *J. High. Educ.* **2018**, *89*, 935–960.
40. Jarman, B.T.; Borgert, A.J.; Kallies, K.J.; Joshi, A.R.; Smink, D.S.; Sarosi, G.A. Underrepresented minorities in general surgery residency: Analysis of interviewed applicants, residents, and core teaching faculty. *J. Am. Coll. Surg.* **2020**, *231*, 54-58.
41. Webber, S.; Schwartz, A.; Kemper, K.J.; Batra, M.; Mahan, J.D.; Babal, J.C.; Pediatric Resident Burnout-Resilience Study Consortium. Faculty and Peer Support during Pediatric Residency: Association with Performance Outcomes, Race, and Gender. *Acad. Pediatrics* **2020**, *21*, 366–374.

42. Okike, K.; Phillips, D.P.; Swart, E.; O'Connor, M.I. Orthopaedic Faculty and Resident Sex Diversity Are Associated with the Orthopaedic Residency Application Rate of Female Medical Students. *J. Bone Jt. Surg.* **2019**, *101*, e56.
43. Sobel, A.D.; Cox, R.M.; Ashinsky, B.; Eberson, C.P.; Mulcahey, M.K. Analysis of factors related to the sex diversity of orthopaedic residency programs in the United States. *J. Bone Jt. Surg. Am. Vol.* **2018**, *100*, e79.
44. Campbell, K.M.; Rodríguez, J.E. Addressing the Minority Tax: Perspectives from Two Diversity Leaders on Building Minority Faculty Success in Academic Medicine. *Acad. Med.* **2019**, *94*, 1854–1857, doi:10.1097/ACM.0000000000002839.
45. Bonifacino, E.; Ufomata, E.O.; Farkas, A.H.; Turner, R.; Corbelli, J.A. Mentorship of Underrepresented Physicians and Trainees in Academic Medicine: A Systematic Review. *J. Gen. Intern. Med.* **2021**, *36*, 1023–1034.
46. Kaplan, S.E.; Raj, A.; Carr, P.L.; Terrin, N.; Breeze, J.L.; Freund, K.M. Race/ethnicity and success in academic medicine: Findings from a longitudinal multi-institutional study. *Acad. Med. J. Assoc. Am. Med. Coll.* **2018**, *93*, 616.
47. Diaz, T.; Navarro, J.R.; Chen, E.H. An institutional approach to fostering inclusion and addressing racial bias: implications for diversity in academic medicine. *Teach. Learn. Med.* **2020**, *32*, 110–116.
48. Harris, T.B.; Thomson, W.A.; Moreno, N.P.; Conrad, S.; White, S.E.; Young, G.H.; Monroe, A.D. Advancing holistic review for faculty recruitment and advancement. *Acad. Med.* **2018**, *93*, 1658–1662.
49. Sood, A.; Brakey, H.R.; Myers, O.; Greenberg, N.; Tigges, B.; Sigl, D.; Wilson, B. Exiting Medicine Faculty Want the Organizational Culture and Climate to Change. *Chron. Mentor. Coach.* **2020**, *4*, 359.
50. Carson, T.L.; Aguilera, A.; Brown, S.D.; Peña, J.; Butler, A.; Dulin, A.; Jonassaint, C.R.; Riley, I.; Vanderbom, K.; Molina, K.M.; et al. A Seat at the Table: Strategic Engagement in Service Activities for Early-Career Faculty from Underrepresented Groups in the Academy. *Acad. Med.* **2019**, *94*, 1089–1093, doi:10.1097/ACM.0000000000002603.
51. Blake-Beard, S.; Murrell, A.J.; Thomas, D.A. Unfinished Business: The Impact of Race on Understanding Mentoring Relationships. In *Handbook on Mentoring*; Rose-Ragins, B., Kram, K., Eds.; Sage Publications: Thousand Oaks, CA, USA, 2007.
52. Rodríguez, J.E.; Campbell, K.M.; Pololi, L.H. Addressing disparities in academic medicine: what of the minority tax? *BMC Med. Educ.* **2015**, *15*, 6, doi:10.1186/s12909-015-0290-9.
53. Beech, B.M.; Calles-Escandon, J.; Hairston, K.G.; Langdon, S.E.; Latham-Sadler, B.A.; Bell, R.A. Mentoring programs for underrepresented minority faculty in academic medical centers: A systematic review of the literature. *Acad. Med.* **2013**, *88*, 541–549, doi:10.1097/ACM.0b013e31828589e3.
54. Straus, S.E.; Straus, C.; Tzanetos, K. Career choice in academic medicine. *J. Gen. Intern. Med.* **2006**, *21*, 1222–1229.
55. South-Paul, J.E.; Campbell, K.M.; Murrell, A.J. Enhancing Diversity within Academic Health Centers. In *Diversity Across the Disciplines Research on People, Policy, Process, and Paradigm*; Murrell, A.J., Petrie-Wyman, J.L.; Soudi, A., Eds.; Information Age Publishing: Charlotte, NY, USA, 2019.
56. Straus, S.E.; Johnson, M.O.; Marquez, C.; Feldman, M.D. Characteristics of successful and failed mentoring relationships: A qualitative study across two academic health centers. *Acad. Med.* **2013**, *88*, 82–89.
57. Ragins, B.R.; Cotton, J.L. Easier said than done: Gender differences in perceived barriers to gaining a mentor. *Acad. Manag. Jnl.* **1991**, *34*, 939–951.
58. Kahn, W.A. Holding environments at work. *Jnl App. Beh. Sci.* **2001**, *37*, 260–279.
59. Chrobot-Mason, D.; Ragins, B.R.; Linnehan, F. Second hand smoke: Ambient racial harassment at work. *J. Manag. Psychol.* **2013**, *28*, 470–491.
60. Robinson, S.L.; Morrison, E.W. The development of psychological contract breach and violation: A longitudinal study. *J. Organ. Behav.* **2000**, *21*, 525–546. .
61. Rousseau, D.M. Schema, promise and mutuality: The building blocks of the psychological contract. *J. Occup. Organ. Psychol.* **2001**, *74*, 511–541.
62. Murrell, A.J.; Blake-Beard, S.; Porter, D.M. *The Importance of Peer Mentoring, Identity Work and Holding Environments: A Study of African American Leadership Development*; Working paper; 2021. Available online:https://www.mdpi.com/journal/ijerph/special_issues/Diversity_Equity_Inclusion (accessed on 30 April 2021).
63. Zagenczyk, T.J.; Gibney, R.; Kiewitz, C.; Restubog, S.L.D. Mentors, supervisors and role models: Do they reduce the effects of psychological contract breach? *Hum. Resour. Manag. J.* **2009**, *19*, 237–259.
64. Gravlee, C.C. Systemic racism, chronic health inequities, and COVID-19: A syndemic in the making? *Amer. Jnl. of Hum. Bio.* **2020**, doi:10.1002/ajhb.23482.
65. Murrell, A.J.; South-Paul, J.E. The emerging power of peer mentoring within academic medicine. In *Mentoring Diverse Leaders: Creating Change for People, Processes, & Paradigms*; Murrell, A.J., Blake-Beard, S., Eds.; Routledge: New York, NY, USA, 2017; pp.85–103.
66. Daley, S.P.; Broyles, S.L.; Rivera, L.M.; Brennan, J.J.; Lu, E.R.; Reznik, V. A conceptual model for faculty development in academic medicine: The underrepresented minority faculty experience. *J. Natl. Med. Assoc.* **2011**, *103*, 816–821.
67. Raphael, J.L. The Role of Sponsorship in Achieving Workforce Diversity in Academic Pediatrics. *Pediatrics* **2019**, *144*, e20191532.
68. Butts, G.C.; Johnson, J.; Strelnick, A.H.; Soto-Greene, M.L.; Williams, B.; Lee-Rey, E. Diversity in academic medicine no. 4 Northeast Consortium: innovation in minority faculty development. *Mt. Sinai J. Med.* **2008**, *75*, 517–522.

69. Sheet Metal Workers v. EEOC, 478 U.S. 421 (1986). Available online: https://supreme.justia.com/cases/federal/us/478/421/ (accessed on 30 April 2021).
70. Coe, C.; Piggott, C.; Davis, A.; Hall, M.N.; Goodell, K.; Joo, P.; South-Paul, J.E. Leadership Pathways in Academic Family Medicine: Focus on Underrepresented Minorities and Women. *Fam. Med.* **2020**, *52*, 104–111.
