# Supplementary material for: Estimation of Undetected Asymptomatic COVID-19 Cases in South Korea Using a Probabilistic Model
Source: Int J Environ Res Public Health. 2021 May 6;18(9):4946. doi: 10.3390/ijerph18094946 (PMC8124955; doi:10.3390/ijerph18094946)
Supplement: Supplementary file 1 [file ijerph-18-04946-s001.zip › 5/ijerph-1190072.pdf]

International Journal of
Environmental Research and Public Health

MDPI

Article

Spatial and Temporal Characteristics of Hand-Foot-and-Mouth Disease and Their Influencing Factors in Urumqi, China

Yibo Gao [1], Hongwei Wang [1,*], Suyan Yi [1], Deping Wang [2], Chen Ma [1], Bo Tan [1] and Yiming Wei [1]

[1] College of Resources and Environmental Sciences, Xinjiang University, Urumqi 830046, China; keioiobo@163.com (Y.G.); yisuyan95@sina.com (S.Y.); machen_666@163.com (C.M.); tanyan0829@163.com (B.T.); wei836764860@163.com (Y.W.)
[2] College of Life Sciences and Technology, Xinjiang University, Urumqi 830046, China; Wang-6212@163.com
* Correspondence: wanghw@xju.edu.cn; Tel.: +86-135-7920-8666

Abstract: Hand, foot, and mouth disease (HFMD) remains a serious health threat to young children. Urumqi is one of the most severely affected cities in northwestern China. This study aims to identify the spatiotemporal distribution characteristics of HFMD, and explore the relationships between driving factors and HFMD in Urumqi, Xinjiang. Methods: HFMD surveillance data from 2014 to 2018 were obtained from the China Center for Disease Control and Prevention. The center of gravity and geographical detector model were used to analyze the spatiotemporal distribution characteristics of HFMD and identify the association between these characteristics and socioeconomic and meteorological factors. Results: A total of 10,725 HFMD cases were reported in Urumqi during the study period. Spatially, the morbidity number of HFMD differed regionally and the density was higher in urban districts than in rural districts. Overall, the development of HFMD in Urumqi expanded toward the southeast. Temporally, we observed that the risk of HFMD peaked from June to July. Furthermore, socioeconomic and meteorological factors, including population density, road density, GDP, temperature and precipitation were significantly associated with the occurrence of HFMD. Conclusions: HFMD cases occurred in spatiotemporal clusters. Our findings showed strong associations between HFMD and socioeconomic and meteorological factors. We comprehensively considered the spatiotemporal distribution characteristics and influencing factors of HFMD, and proposed some intervention strategies that may assist in predicting the morbidity number of HFMD.

Keywords: hand; foot; and mouth disease; spatiotemporal analysis; geographical detector model; driving factors

Citation: Gao, Y.; Wang, H.; Yi, S.; Wang, D.; Ma, C.; Tan, B.; Wei, Y. Spatial and Temporal Characteristics of Hand-Foot-and-Mouth Disease and Their Influencing Factors in Urumqi, China. *Int. J. Environ. Res. Public Health* **2021**, *18*, 4919. https://doi.org/10.3390/ijerph18094919

Academic Editor: Paul B. Tchounwou

Received: 2 April 2021
Accepted: 2 May 2021
Published: 5 May 2021

Publisher's Note: MDPI stays neutral with regard to jurisdictional claims in published maps and institutional affiliations.

Copyright: © 2021 by the authors. Licensee MDPI, Basel, Switzerland. This article is an open access article distributed under the terms and conditions of the Creative Commons Attribution (CC BY) license (http://creativecommons.org/licenses/by/4.0/).

1. Introduction

Hand, foot, and mouth disease (HFMD) has resulted in major outbreaks worldwide in the past three decades, and has become a serious public health issue in affected countries [1,2]. The main pathogens of HFMD are Coxsackie virus A16 (CA16) and Enterovirus 71 (EV71) [3]. HFMD is commonly seen in children between the ages of 0 and 15, especially children under five years of age [4]. The typical clinical symptoms include fever, skin bursts on hands and feet, and painful sores in the mouth [5]. In recent years, outbreaks of HFMD have remained common in most of the provinces of China and have threatened the health of young children [6]. Therefore, it is essential to identify the driving factors of HFMD and establish an early warning system to reduce the disease burden [7].

In China, HFMD epidemics have shown spatiotemporal agglomeration effects. For example in Sichuan Province [8] and Zhejiang Province [6], China, spatiotemporal clusters have become increasingly concentrated. Existing studies have widely accepted that socioeconomic factors and meteorological factors are related to the morbidity number of HFMD. The gross domestic product (GDP) [9,10] population density [11,12], road density [13] and land cover types [14] have also been demonstrated to affect the morbidity number of HFMD. Different HFMD incidence patterns have different climate conditions [15]. Certain temperature, precipitation, air pressure and wind speed values as key meteorological factors could be considered alarm values for the early warning of HFMD. For example, Fu et al. [16] found that the monthly mean temperature had a positive effect on HFMD when it was higher than 17 °C. Cheng et al. found that HFMD occurrence was significantly influenced by extreme precipitation [17] However, Yang et al. [18] observed no such association in Hong Kong. Li et al. revealed a 6.8% drop in cases for every 1 hPa increase in air pressure in Guangdong [19] Deng et al. showed a strong association between HFMD and wind speed, this result may assist in predicting HFMD incidence [20].

Using the results of previous studies, we concluded that these driving factors have complicated mechanisms regarding HFMD occurrence and development in different regions. Little attention has been given to arid regions. A typical arid city, Urumqi is the capital of Xinjiang Uygur Autonomous and is located in northwestern China; its urban areas and population are expanding rapidly. Urumqi has unique meteorological conditions and socioeconomic conditions. Therefore, we selected Urumqi as our research area and first analyzed the spatiotemporal distribution of HFMD in Urumqi from 2014-2018, including the annual scale and the monthly scale; then, we used the center of the gravity model to find the trajectory of the center of HFMD cases. Next, we used a geographical detector to identify driving factors for HFMD considering both meteorological and socioeconomic aspects. Finally, by comprehensively considering the spatial and temporal distribution characteristics and influencing factors of HFMD in Urumqi, we propose some intervention strategies. These intervention strategies could be helpful in preventing and controlling HFMD. We aim to enrich HFMD studies in different regions and provide a theoretical basis for HFMD.

2. Materials and Methods

2.1. Study Area

Urumqi (42°45′ to 44°08′ N, 86°37 ′to 88°58′ E)is located in northwestern of China, in the central part of the Xinjiang Uyghur Autonomous Region. It lies at the northern foot of the Tianshan Mountains, south of the Dzungarian Basin. The total land area of Urumqi is 14,216.3 km^2, with a population of 3.552 million at the end of 2019, with a GDP exceeding 341.3 billion Yuan (Xinjiang Statistical Yearbook, 2019). Urumqi consists of eight districts: Tianshan District, Saybagh District, New Urban District, Toutunhe District, Daban District, Midong District, Shuimogou District and Urumqi County (Figure 1). The local climate type is a temperate continental climate, that is warm and rainy in summer, and cold and dry in winter [21]. The average annual temperature is 7 °C, the average annual precipitation is 280 mm, and the evaporation is 2730 mm [22]. Urumqi is an oasis city located in an arid region, and one of the most important rising cities in northwestern China and Central Asia [23]. Benefiting from the One Belt and One Road policy, Urumqi as important node city, has witnessed rapid economic development [24].

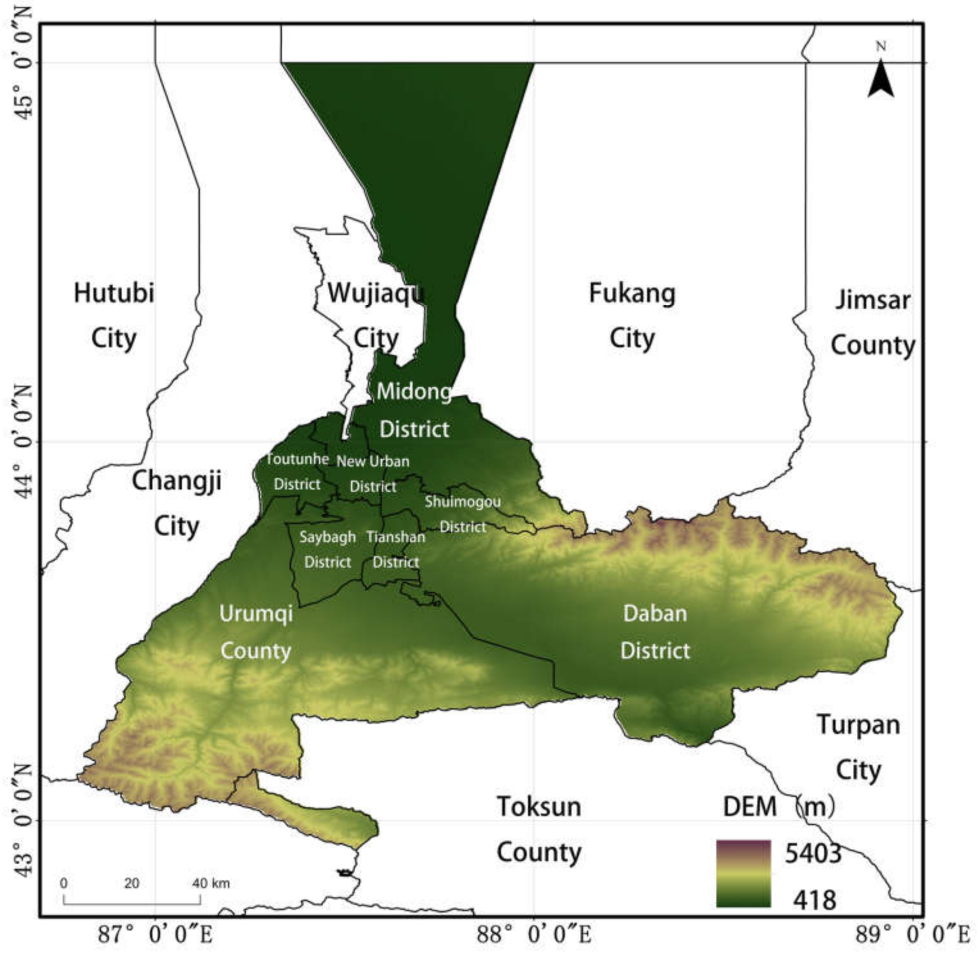

Figure 1. Location of Urumqi, China.

2.2. Data Acquisition

For the study area of Urumqi, we acquired district-level daily HFMD surveillance data from 2014 to 2018, including date of birth, age, sex, occupation and detailed address (Table 1), these data were obtained from the China Center for Disease Control and Prevention (CDC) Between 1 January 2014 and 31 December 2018, there were a total of 10,725 HFMD cases. We used ArcGIS software to display the detailed addresses of cases on a map.

Table 1. Sample data of HFMD.

Number	National Code of Current Address	Address Longitude and Latitude	Case Classification	Date of Accident	Reporting Unit
1	650100000	88.209° E<br>43.363° N	clinically diagnosed cases	2014/1/1	Tianshan District Hospital
2	650100001	88.119° E<br>43.463° N	clinically diagnosed cases	2016/1/1	Midong District Hospital

……	……	……	……	……	……
N	650100002	88.129° E<br>43.553° N	clinically diagnosed cases	2018/1/1	Tianshan District Hospital

We included three socioeconomic variables and three meteorological variables as the driving factors for HFMD in this study. Socioeconomic data including gross domestic product(GDP), population density and road network density, were obtained from the Urumqi Statistical Bulletin and Urumqi Statistical Yearbook [25]. Meteorological data, including temperature, precipitation and wind speed, were analyzed [26] from the China Climate Data Sharing Service System (http://data.cma.cn/ (accessed on 2 February 2021)) and National Meteorological Information Center [27].

2.3. Statistical Methods

2.3.1. Center of Gravity

The disease center of gravity (CoG) is an important index used to accurately depict the overall spatial distribution of a disease, this index is helpful for studying the direction of disease development and plays an important role in the prevention and control of future diseases. In this paper, we used the center of gravity for HFMD to analyze the spatial characteristics of HFMD cases. The formula for calculating the CoG of HFMD cases is as follows [28]:

$$\bar{X} = \sum_{i=1}^{n} w_i x_i / \sum_{i=1}^{n} w_i$$
$$\bar{Y} = \sum_{i=1}^{n} w_i y_i / \sum_{i=1}^{n} w_i \quad (1)$$

where X and Y represent the latitude and longitude coordinates of the gravity center of HFMD and X_i and Y_i indicate the latitude and longitude coordinates, respectively, of each HFMD case detailed address.

The shift distance of the gravity center can be used to express the spatial change characteristics of the feature type, and the expression is as follows [29]:

$$d = \sqrt{\left(\bar{X}_t - \bar{X}_{t-1}\right)^2 + \left(\bar{Y}_t - \bar{Y}_{t-1}\right)^2} \quad (2)$$

where (X_t, Y_t) and (X_{t-1}, Y_{t-1}) are the gravity centers of the elements at different times.

2.3.2. Geographical Detector

The geographical detector (GeoD) is a set of statistical methods used to detect spatial differentiation and reveal the driving forces behind it [30]. As the geographical detector is less restricted by the premise than the center of gravity, the GeoD has strong universality for exploring the formation mechanism and influencing factors of the spatial heterogeneity of geographic objects. It is freely available from http://www.geodetector.org/ (accessed on 11 February 2021) [31].

The GeoD is composed of interactive, factor, ecological, and risk detectors. In this study, we mainly applied the factor detector and the interaction detector [32]. Factor detector was used to calculate the explanatory power of each impact factor on the spatial differentiation of HFMD, this detector could quantitatively rank and screen the importance of HFMD driving factors. Based on the socioeconomic data and climate data, we used the GeoD to screen and extract the most important driving factors (X) of HFMD distribution (Y) in Urumqi. This model formula is as follows:

$$q = 1 - \frac{1}{N\sigma^2}\sum_{h=1}^{L} N_n \sigma^2 h \quad (3)$$

where q represents the explanatory power of a driving factor on HFMD spatial differentiation. The q-value range is between 0 and 1; it indicates the extent to which Y is interpreted by X. In detail, $q = 0$ indicates there is no association between Y and X, while $q = 1$ means that Y is completely determined by X [33], the higher the value of q is, the stronger the explanatory power of this factor for HFMD differentiation will be.

Interaction detectors are used to identify interactions among different driving factors(X), that is, whether factors X_1 and X_2 interact to increase or decrease the explanatory power of the dependent variable (Y) or whether the effects of these factors on Y are independent of each other. First, we compute the q-values of the two factors X_1 and X_2. Then, we superimpose these two factors and compute their q-values ($q(X_1 \cap X_2)$) [34]. The relationship between these two factors yields one of the following five results (Table 2).

Table 2. Types of interaction between two variables.

	Relationship	Interaction
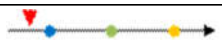	$q(X_1 \cap X_2) < Min(q(X_1),q(X_2))$	Nonlinear weakening
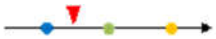	$Min(q(X_1),q(X_2)) < q(X_1 \cap X_2) < Max(q(X_1)),q(X_2))$	Single-factor nonlinear weakening
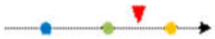	$q(X_1 \cap X_2) > Max(q(X_1),q(X_2))$	Double-factor enhancement
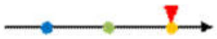	$q(X_1 \cap X_2) = q(X_1)+q(X_2)$	Independent
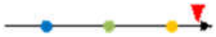	$q(X_1 \cap X_2) > q(X_1)+q(X_2)$	Nonlinear enhancement

3. Results

3.1. Spatial and Temporal Distributions of HFMD

The spatial distribution characteristics of HFMD in Urumqi were obtained and are shown in Figure 2. It is clear from the results that the overall HFMD spatial distribution in the research area changed significantly from 2014 to 2018, and the number of people suffering from HFMD is constantly increasing. As shown in Figure 2, each red area represents an area with a total number of HFMD cases between 67 and 128, indicating a high-level HFMD density. The red areas in the figure continuously extended from 2014 to 2018, and the increased area was concentrated in Tianshan District, Saybagh District, Toutunhe District and New Urban District. Simultaneously, the blue area in the figure continuously extended from 2014 to 2018, and blue areas indicate a low-level HFMD density in which number of HFMD cases is between 1 and 29; these regions are mostly concentrated in Daban District and Urumqi County. In summary, HFMD spread to a wide range of districts over the studied period, reaching a maximum in 2018.

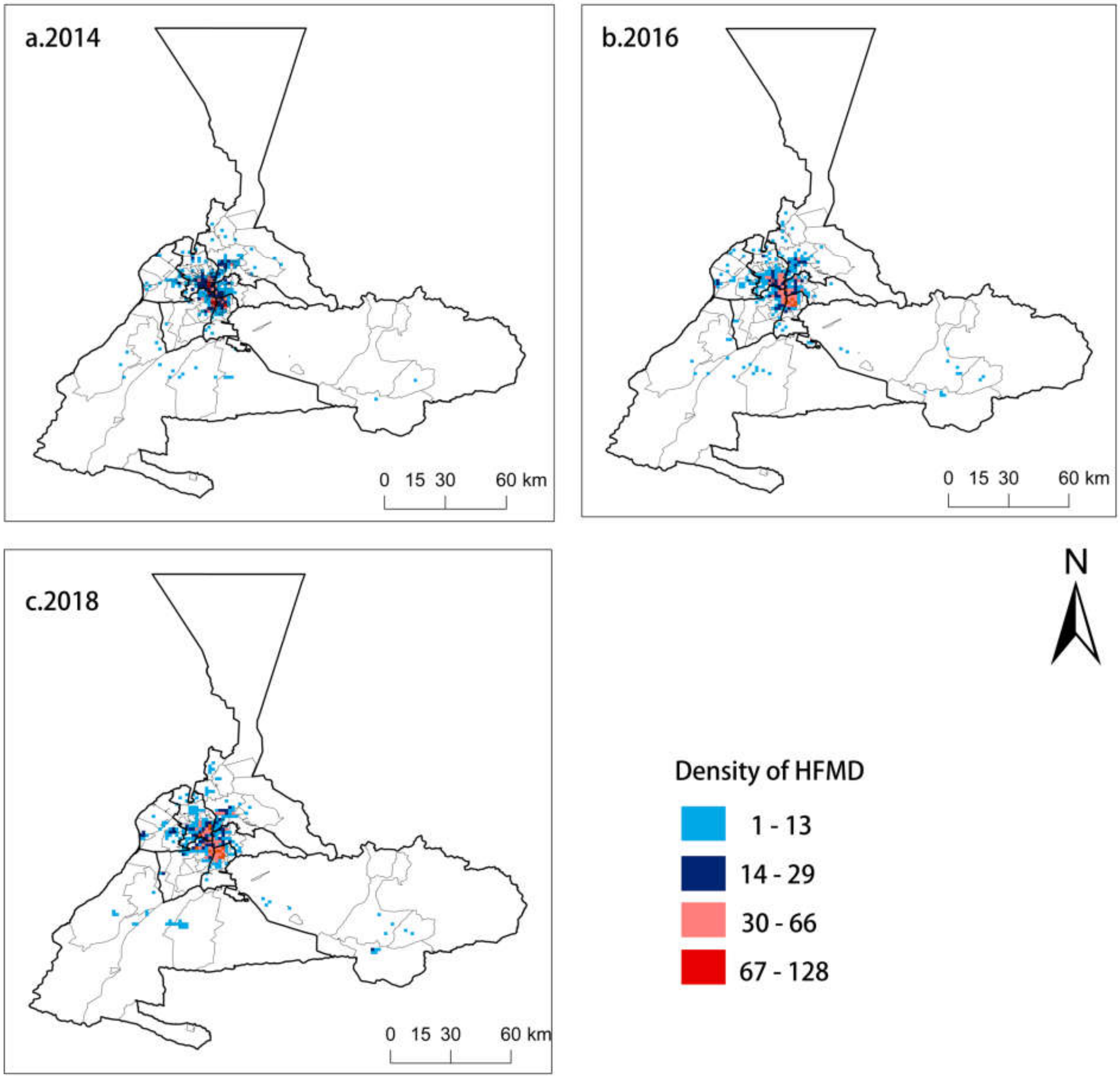

Figure 2. Spatial distribution of HFMD in Urumqi from 2014 to 2018 ((**a**). 2014, (**b**). 2016, (**c**). 2018).

There are significant differences observed in the HFMD case density distribution among different districts and counties in Urumqi. The case density distribution can be divided into two levels: ① high-density districts. As shown in Figure 3, the high-density districts include Tianshan District, Saybagh District, Toutunhe District and the New Urban District. The numbers of HFMD cases in these four districts showed trends of continuously increasing, and the maximum density was 128 cases. Among these four districts, Tianshan District and Shayibuck District are the traditional city centers of Urumqi and are gathering places for political, economic, cultural, and residential activities. In addition, the New Urban District is the subcenter of Urumqi., where high-end industries and strategic emerging industries are developing; ② low-density districts. As shown in Figure 4, low-density districts, including Shuimogou District, Midong District, Daban District and Urumqi County, had the lowest density of 0 patients. Low-density districts are mostly rural settlements and pastures that are mainly engaged in agricultural and animal husbandry production activities.

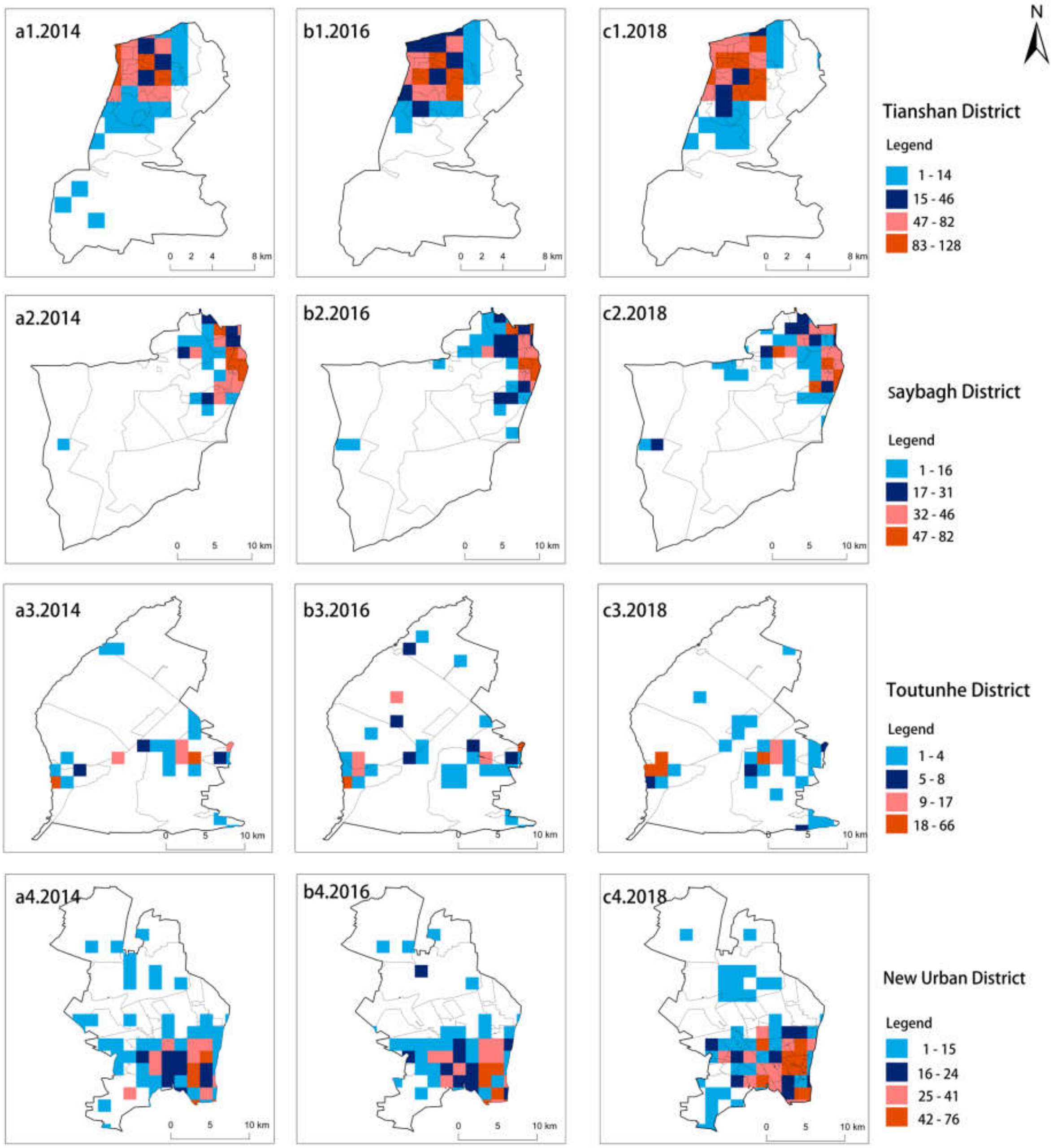

Figure 3. High-density HFMD districts in Urumqi from 2014 to 2018 ((**a1–a4**). 2014, (**b1–b4**). 2016, (**c1–c4**). 2018).

The temporal distribution characteristics of HFMD in Urumqi are shown in Table 3 and Figure 4. The annual-scale analysis showed that (Table 3) 2014 had the lowest number of cases, with a total of 2553. In 2016, the number of HFMD cases was 3689, showing an increase of 1136 and the growth rate was 44.49%.The highest number of HFMD cases occurred in 2018, with 4483 cases and a growth rate of 21.52%. The month-scale analysis showed that (Figure 5) from December to March, new cases were rare, and the increase rate of morbidity was extremely slow. From April to May, the number of new cases gradually increased. From June to July, the number of new cases increased sharply and reached a peak. From August to November, the number of new cases dropped rapidly, and the HFMD epidemic gradually subsided.

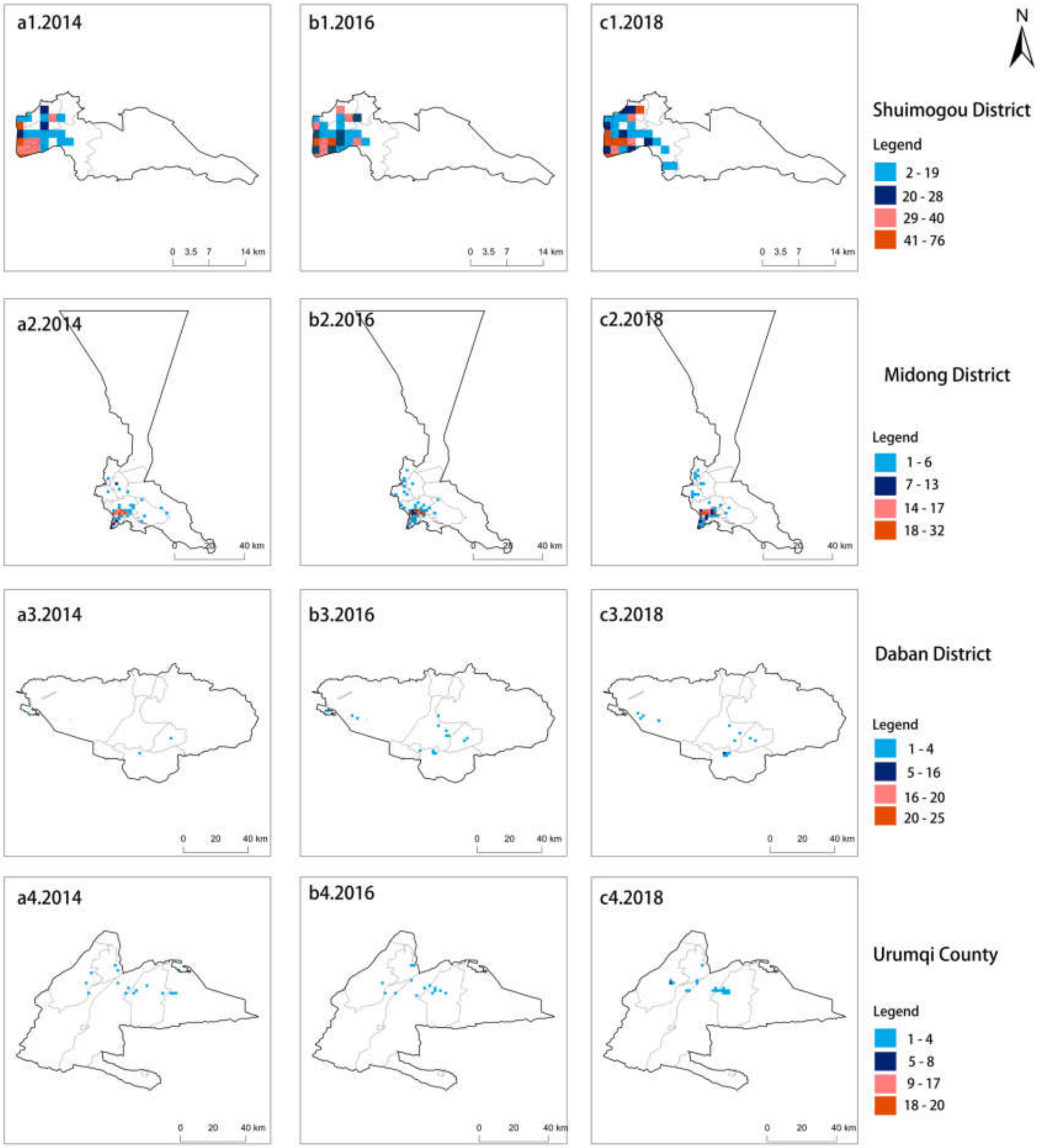

Figure 4. Low-density HFMD districts in Urumqi from 2014 to 2018 ((**a1–a4**). 2014, (**b1–b4**). 2016, (**c1–c4**). 2018).

Table 3. Number of HFMD cases in Urumqi from 2014 to 2018.

District / Year	2014	2015	2018	Average Number Of Cases	Average Growth Rate (%)
Tianshan	423	846	1123	797	66.37
Shayibuck	675	774	883	777	14.37
Toutunhe	223	327	289	280	17.5
New Urban	664	793	1059	839	26.4
Shuimogou	292	593	586	490	50.95

Midong	247	295	435	326	33.45
Daban	4	33	43	27	377
Urumqi	25	28	65	39	72.07
Sum	2553	3689	4483	-	-

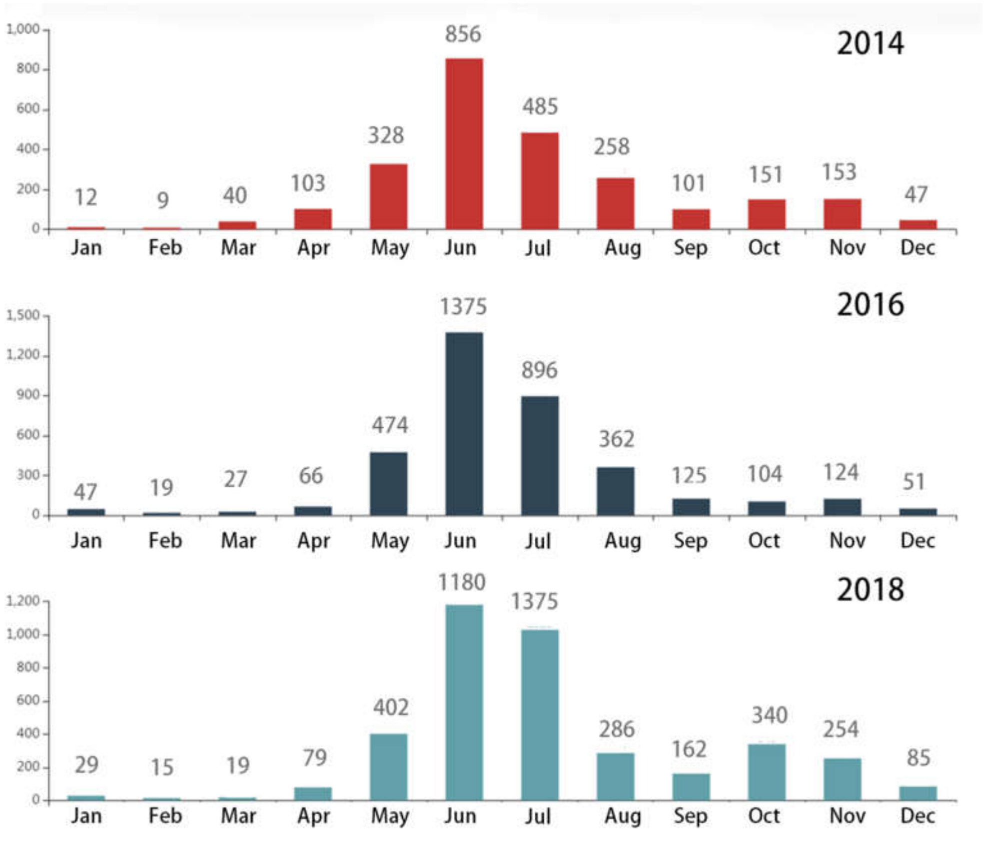

Figure 5. Month scale distribution of HFMD cases in Urumqi from 2014 to 2018.

3.2. Change of the Center of Gravity of HFMD

The HFMD cases and their geographical location information were used to build the center of the gravity model according to equations 2 and 3. The results are shown in Figure 6. In 2014, the HFMD COG was located in the New Urban District (87.5844° E, 43.8382° N),while in 2016 (87.5906° E, 43.8325° N)and 2018 (87.5913° E, 43.8309° N), the HFMD COG shifted to Shaybak District. From 2014 to 2016, the center shifted to the southeast, with a distance of 805.5 m, and from 2016 to 2018, the center again shifted to the southeast, with a distance of 182.9 m. This indicates that the overall development of HFMD in Urumqi from 2014 to 2018 expanded toward the southeast. New high-density clusters of HFMD have formed in the Shaybak District.

3.3. Analysis of the Driving Factors of HFMD

Relationships among socioeconomic factors, meteorological factors and the incidence of various infectious diseases have frequently been observed. On the basis of referring to existing studies [11,12,20], we screened factors at both socioeconomic and meteorological levels. Specifically, driving factors can be classified into six aspects: GDP (X_1), population density (X_2), road density (X_3), temperature (X_4), precipitation (X_5) and

wind speed (X_6). The q-value calculations shown in Table 4 display the extent of the influence of the six driving factors on the spatial heterogeneity of HFMD.

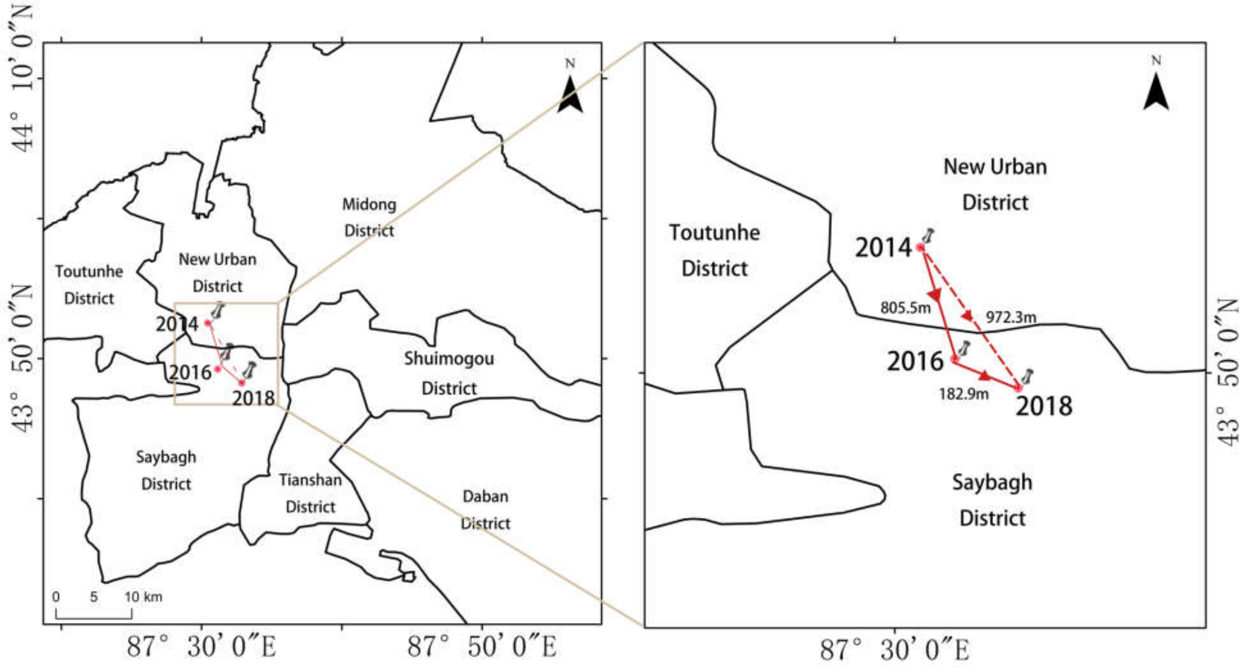

Figure 6. Changes in the center of gravity of HFMD from 2014 to 2018.

(1) Factor detector is mainly used to detect influencing factors' explanatory powers. Each driving factor has a different degree of influence. The top 5 driving factors of HFMD were ranked by the q-value (Table 4) as follows: population density (X_2) > road density (X_3) > GDP (X_1) > temperature (X_4) > precipitation (X_5) > wind speed (X_6).

Table 4. Detection results of single driving factors.

Level of Impact (q-Value)	GDP X_1	Population Density X_2	Road Density X_3	Temperature X_4	Precipitation X_5	Wind Speed X_6
2014	0.598	0.569	0.623	0.462	0.368	0.194
2016	0.451	0.552	0.449	0.495	0.414	0.151
2018	0.450	0.540	0.558	0.546	0.408	0.147
Mean Value	0.500	0.554	0.544	0.501	0.397	0.164

Note: The factor detection results all passed the 1% significance test.

(2)Interaction detector is mainly used to calculate the interactions among different factors. The analysis results showed a high degree of consistency with the previous section. Furthermore, indicated that the influence of any two factors on the HFMD cases was enhanced under their interaction. Tables 5–7 show the results of the interactions of population density (X_2) with other factors: $X_2 \cap X_3$ (0.878) > $X_2 \cap X_6$ (0.830) > $X_2 \cap X_1$ (0.829) > $X_2 \cap X_4$ (0.826) > $X_2 \cap X_5$ (0.825). The results showed that the interaction between population density∩ road density had the strongest effect on the spatial distribution of HFMD in 2014. Similarly, the interaction between population density∩ road density (q=0.881) had the strongest effect on the spatial distribution of HFMD in 2016, the interaction between

population density∩ road density (q = 0.859) also had the strongest effect on the spatial distribution of HFMD in 2018. This phenomenon shows that socioeconomic factors such as "fortifiers" can strengthen the explanatory powers of other factors regarding HFMD spatial heterogeneity.

Finally, according to the results of the factor detector and interaction detector, we chose the top five HFMD impact factors and created a map. Figure 7 illustrates the spatial distributions of HFMD under the influence of various driving factors. As shown in Figure 7, the spatial distribution of HFMD was affected by socioeconomic factors. HFMD cases gather around economically developed, densely-populated and high-road-density districts, and the mean p values were 0.500, 0.554, and 0.544, respectively. Tianshan District, Saybagh District, Toutunhe District and the New Urban District complied with the above characteristics. (2) As affected by meteorological factors, HFMD patients were concentrated in warm and moist districts, and the mean p values were 0.501 and 0.397 respectively. In dry and cold areas, virtually no one was infected with HFMD.

Table 5. Interaction detector results for 2014.

Variable	GDP X_1	Population Density X_2	Road Density X_3	Temperature X_4	Precipitation X_5	Wind Speed X_6
X_1	0.598					
X_2	0.829	0.569				
X_3	0.776	0.878	0.623			
X_4	0.637	0.826	0.667	0.462		
X_5	0.677	0.825	0.665	0.577	0.368	
X_6	0.594	0.830	0.625	0.834	0.362	0.194

Table 6. Interaction detector results for 2016.

Variable	GDP X_1	Population Density X_2	Road Density X_3	Temperature X_4	Precipitation X_5	Wind Speed X_6
X_1	0.451					
X_2	0.849	0.552				
X_3	0.580	0.881	0.449			
X_4	0.626	0.667	0.619	0.495		
X_5	0.601	0.665	0.472	0.572	0.414	
X_6	0.411	0.619	0.575	0.571	0.594	0.151

Table 7. Interaction detector results for 2018.

Variable	GDP X_1	Population Density X_2	Road Density X_3	Temperature X_4	Precipitation X_5	Wind Speed X_6
X_1	0.450					
X_2	0.805	0.540				
X_3	0.589	0.859	0.558			
X_4	0.634	0.818	0.669	0.546		
X_5	0.592	0.799	0.667	0.567	0.408	
X_6	0.612	0.609	0.573	0.624	0.577	0.147

Note: The factor detection results all passed the 1% significance test.

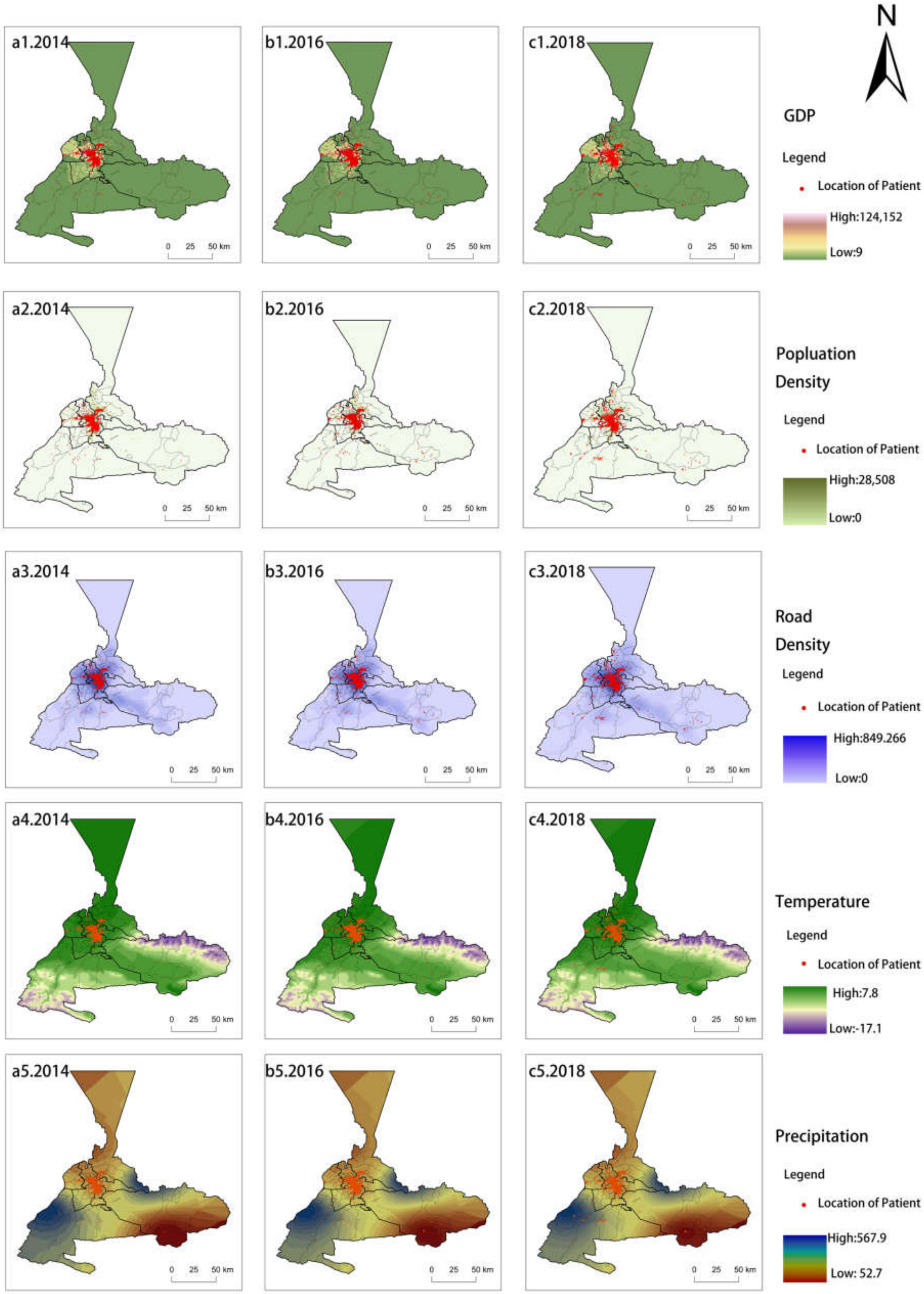

Figure 7. Spatial distributions of HFMD under the influence of different driving factors ((**a1–a5**). 2014, (**b1–b5**). 2016, (**c1–c5**). 2018).

4. Discussion

The present study describes the spatiotemporal distribution of HFMD in Urumqi and the relationships between driving factors and the morbidity number of HFMD. The results revealed that areas with high HFMD densities were located in four main urban districts, which are the economically developed areas of Urumqi. Through the geographical detector model, we found that socioeconomic and meteorological factors, including the population density, road density, GDP, temperature and precipitation were all significantly associated with the occurrence of HFMD.

Our study demonstrates that the morbidity number of HFMD has regional differences in that the density is higher in urban districts than in rural districts. The spatial distribution of HFMD was evident in Tianshan District, Saybagh District, Toutunhe District and the New Urban District, probably because the urban district areas have more residential areas and higher population densities, possibly leading to clustered HFMD infections. This is supported by a study in Shandong that found that the incidence rate was significantly higher in urban areas than in rural areas [35]. In addition, Qi et al. found that cluster centers were located in 9 major urban districts in Chongqing [36].

We observed that, from June to July, the number of HFMD cases increased sharply and reached a peak. From August to November, the number of HFMD cases dropped rapidly, and the HFMD epidemic gradually subsided. In June and July, the temperature is appropriate, virus-susceptible population spent more time in outdoors, which increases the possibility of exposure to the virus. While after August, the temperature drops rapidly, susceptible population stay indoors for a long time, thus reducing the possibility of infection. This finding was consistent with studies conducted in other provinces and regions in China. A study in mainland China found that July had a higher risk than other months, while November had a lower risk [37]. In addition, HFMD reached a single peak from May to July in Beijing [38]. These findings are also consistent with an existing study conducted in the Ili River Valley, China [39]. Different meteorological conditions may be the underlying reason for the different HFMD peak months.

Regarding the socioeconomic aspect, our current study found that the GDP was significantly associated with the morbidity number of HFMD. With the growth of GDP, the number of HFMD cases continuously increased. Furthermore, districts with higher GDP had more HFMD cases. Tianshan District, Saybagh District, Toutunhe District and the New Urban District are the economic centers of Urumqi, and their HFMD cluster effects were more obvious than those of other districts. This view was also supported by a study that found that GDP was significantly positively associated with the Shandong HFMD incidence [9]. A study conducted in Guangdong found that counties with higher relative risks were mainly gathered in the Pearl River Delta region, which is located in the economic center of Guangdong [10]. Another study concluded that urban areas had a higher risk of HFMD than poorer areas, which is similar to our finding that GDP is positively correlated with HFMD incidence [40].

We found that the road density was also significantly associated with the morbidity number of HFMD. According to geographical detectors and case maps, the vast majority of HMFD cases are distributed along roads. This reflected that a high road density led to an increased risk of HFMD. Urumqi is a transportation hub and a distribution center for passengers in Xinjiang. Summer is the peak tourist season of Urumqi, highways, railways, and air transport have to bear huge volumes of passenger transport. In the meantime, many tourists travel between different regions and tourist attractions, thus greatly accelerating the spread of the virus. This result is similar to the results obtained by Lin et al. Their study concluded that a high road density makes it easier for the virus to attach to certain substances, thus facilitating the spread of the virus [13]. This finding is consistent with other reports that clusters are more commonly observed in areas of high mobility [41,42], further suggesting that the disease may be spread by roads and railways.

Population density also played a large role in affecting the occurrence of HFMD. Districts with higher population densities have higher morbidity number of HFMD. Tianshan District, Saybagh District, Toutunhe District and the New Urban District follow this regularity. Compared with previous results, Huang et al. found that when a population density is relatively small, an outbreak of HFMD occurs relatively late, and when a population density gradually increases, HFMD outbreaks occur earlier [43]. Kim et al., according to this regularity, suggested a proposal that focuses on HFMD outbreaks in densely populated areas [44].

Regarding the meteorological aspect, our current study found that temperature and precipitation were significantly associated with the morbidity number of HFMD. High temperature circumstances strengthen the links between HFMD and driving factors. At higher temperatures, it is easier to contact a contaminated environment. Another study revealed the same results: in 143 cities in mainland China, close links were found between high ambient temperatures and HFMD occurrence. Due to an increase in temperature, the spread of HFMD accelerates. This may be because meteorological indices can increase the duration time of the impacts of temperature on HFMD [45]. Similarly, Cheng et al. observed that more than 61% of childhood HFMD cases occurred when the weather was rainy [18]. Extreme precipitation may enhance the contact rates in the population, lengthen the survival of viruses and expand the scope of viruses, thus driving the spread of HFMD [46]. Huang et al. provided abundant experimental data and found that HFMD can be aggravated at high temperatures and high relative precipitation [47].

Unlike previous studies that showed that wind speed has a positive effect on the occurrence of HFMD [48], according to the geographical detector results obtained in this study, the wind speed p-value was only 0.164, significantly lower than that of other factors; wind speed was not an obvious factor affecting HFMD morbidity number in Urumqi. Based on the results of previous studies, we conclude that meteorological factors play a complicated role in HFMD [49]. These inconsistent results may be due to different local meteorological and socioeconomic backgrounds. It seems that each country, region and terrain has a linear or nonlinear relationship with climatic parameters [50–52]. This suggests that different countries and regions must determine their unique associations with respective climatic factors.

The present study has some limitations. First, meteorological data we used in the study were obtained from meteorological surveillance stations surrounding Urumqi and may not reflect the actual climatic conditions of the infected people. Second, there is a lack of HFMD virus classification in this study. Further studies should consider stratification by virus type. Third, we did not take personal factors, such as occupation, family members, or income, into account. Taking other potential impacting factors into consideration in the geographical detector model would improve the prediction accuracy of HFMD occurrence.

5. Conclusions

In this study, HFMD in Urumqi showed significant spatiotemporal heterogeneity, mainly gathered in the urban district. The case density was higher in the urban districts than in the rural districts. The overall development of HFMD in Urumqi from 2014 to 2018 expanded toward the southeast. A geographical detector was applied to analyze the relationships between driving factors (socioeconomic, meteorological) and the morbidity number of HFMD. We found that the GDP, population density, road density, temperature and precipitation are driving factors of HFMD.

Comprehensively considering the spatial and temporal distribution characteristics and influencing factors of HFMD in Urumqi, we propose the following intervention strategies: (1) Given the density of patients and the development direction of HFMD over the years, the focus of prevention and control should be in the HFMD high-density areas including Tianshan District, Saybagh District, Toutunhe District and New Urban District. Through rational deployment of manpower and material resources to achieve efficient

prevention and control. Meanwhile, the government needs to focus on susceptible population within these areas and strengthen the implementation of vaccinations of children of the appropriate age. (2) From the perspective of GDP, the government should reduce the gathering activities which can cause serious transmission in developed areas, and improve the living and medical conditions in undeveloped areas. In developed urban areas such as Tianshan District, Saybagh District, Toutunhe District and New Urban District, there are many places open to virus-susceptible population, such as parks, training institutions, amusement parks, shopping malls, and supermarkets. As a result, susceptible groups and their families should reduce the frequency of entering these places so as to avoid infection. For rural areas with underdeveloped economies such as Shuimogou District, Midong District, Daban District, and Urumqi County, the government can improve the living condition and medical conditions in these areas, make it convenient for patients to receive timely medical treatment and prevent the disease from spreading. (3) From the perspective of population density, during the outbreak period of HFMD, susceptible groups and their families, from densely populated districts and counties such as Tianshan District, Saybagh District, and New Urban District, should reduce their participation in gathering activities to avoid infection. At the same time, in the process of urbanization, the government should plan for urban expansion in a reasonable manner thus optimizing the residential environment and reducing population density. (4) From the perspective of the road density, it is high in Tianshan District, Saybagh District, and New Urban District. And railway stations, bus stations, and airports are located in these areas. Hence, susceptible population and their families should avoid staying too long in these places during outbreak period of HFMD. At the same time, they should pay attention to personal hygiene to reduce the chance of getting infected. (5) From the perspective of climate, as the temperature in Urumqi city warms up and precipitation increases, it is essential for the meteorological department and the disease control department to remind the public to prepare for the high-incidence season of HFMD. The daily management of nursery institutions and schools and daily disinfection should be strengthened. Besides, the government should organize authoritative experts to publicize knowledge on how to prevent and control HFMD. These intervention strategies could be helpful in preventing and controlling HFMD.

Author Contributions: Conceptualization, Y.G. and H.W.; methodology, S.Y.; software, B.T. and Y.W.; investigation: C.M.; visualization, Y.G.; writing—original draft, Y.G.; supervision, D.W.; funding acquisition, H.W. All authors have read and agreed to the published version of the manuscript.

Funding: This research was funded by the National Natural Science Foundation of China (No. 41861037).and The Group supporting projects for study abroad sent by the People's Government of the Autonomous Region (Grant No. L06).

Institutional Review Board Statement: Not applicable.

Informed Consent Statement: Not applicable.

Data Availability Statement: 3rd Party data, not applicable.

Conflicts of Interest: The authors declare no conflicts of interest.

References

1. Qi, H.; Chen, Y.; Xu, D.; Su, H.; Zhan, L.; Xu, Z.; Huang, Y.; He, Q.; Hu, Y.; Lynn, H.; et al. Impact of meteorological factors on the incidence of childhood hand, foot, and mouth disease (HFMD) analyzed by DLNMs-based time series approach. *Infect. Dis. Poverty* **2018**, *7*, 7, doi:10.1186/s40249-018-0388-5.
2. Liu, Z.; Meng, Y.; Xiang, H.; Lu, Y.; Liu, S. Association of Short-Term Exposure to Meteorological Factors and Risk of Hand, Foot, and Mouth Disease: A Systematic Review and Meta-Analysis. *Int. J. Environ. Res. Public Heal.* **2020**, *17*, 8017, doi:10.3390/ijerph17218017.
3. Ooi, M.H.; Wong, S.C.; Lewthwaite, P.; Cardosa, M.J.; Solomon, T. Clinical features, diagnosis, and management of enterovirus 71. *Lancet Neurol.* **2010**, *9*, 1097–1105, doi:10.1016/s1474-4422(10)70209-x.

4. Wu, J.T.; Jit, M.; Zheng, Y.; Leung, K.; Xing, W.; Yang, J.; Liao, Q.; Cowling, B.J.; Yang, B.; Lau, E.H.Y.; et al. Routine Pediatric Enterovirus 71 Vaccination in China: a Cost-Effectiveness Analysis. *PLoS Med.* **2016**, *13*, e1001975, doi:10.1371/journal.pmed.1001975.
5. Park, S.K.; Park, B.; Ki, M.; Kim, H.; Lee, K.; Jung, C.; Sohn, Y.M.; Choi, S.-M.; Kim, D.-K.; Lee, D.S.; et al. Transmission of Seasonal Outbreak of Childhood Enteroviral Aseptic Meningitis and Hand-foot-mouth Disease. *J. Korean Med Sci.* **2010**, *25*, 677–683, doi:10.3346/jkms.2010.25.5.677.
6. Gui, J.; Liu, Z.; Zhang, T.; Hua, Q.; Jiang, Z.; Chen, B.; Gu, H.; Lv, H.; Dong, C. Epidemiological Characteristics and Spatial-Temporal Clusters of Hand, Foot, and Mouth Disease in Zhejiang Province, China, 2008-2012. *PLOS ONE* **2015**, *10*, e0139109, doi:10.1371/journal.pone.0139109.
7. Yan, S.; Wei, L.; Duan, Y.; Li, H.; Liao, Y.; Lv, Q.; Zhu, F.; Wang, Z.; Lu, W.; Yin, P.; et al. Short-Term Effects of Meteorological Factors and Air Pollutants on Hand, Foot and Mouth Disease among Children in Shenzhen, China, 2009–2017. *Int. J. Environ. Res. Public Heal.* **2019**, *16*, 3639, doi:10.3390/ijerph16193639.
8. Peng, D.; Ma, Y.; Liu, Y.; Lv, Q.; Yin, F. Epidemiological and aetiological characteristics of hand, foot, and mouth disease in Sichuan Province, China, 2011–2017. *Sci. Rep.* **2020**, *10*, 1–9, doi:10.1038/s41598-020-63274-3.
9. Li, L.; Qiu, W.; Xu, C.; Wang, J. A spatiotemporal mixed model to assess the influence of environmental and socioeconomic factors on the incidence of hand, foot and mouth disease. *BMC Public Heal.* **2018**, *18*, 1–12, doi:10.1186/s12889-018-5169-3.
10. Wang, Y.; Lai, Y.; Du, Z.; Zhang, W.; Feng, C.; Li, R.; Hao, Y. Spatiotemporal Distribution of Hand, Foot, and Mouth Disease in Guangdong Province, China and Potential Predictors, 2009–2012. *Int. J. Environ. Res. Public Heal.* **2019**, *16*, 1191, doi:10.3390/ijerph16071191.
11. Hu, M.; Li, Z.; Wang, J.; Jia, L.; Liao, Y.; Lai, S.; Guo, Y.; Zhao, D.; Yang, W. Determinants of the Incidence of Hand, Foot and Mouth Disease in China Using Geographically Weighted Regression Models. *PLOS ONE* **2012**, *7*, e38978, doi:10.1371/journal.pone.0038978.
12. Hong, Z.; Hao, H.; Li, C.; Du, W.; Wei, L.; Wang, H. Exploration of potential risks of Hand, Foot, and Mouth Disease in Inner Mongolia Autonomous Region, China Using Geographically Weighted Regression Model. *Sci. Rep.* **2018**, *8*, 17707, doi:10.1038/s41598-018-35721-9.
13. Lin, H.; Zou, H.; Wang, Q.; Liu, C.; Lang, L.; Hou, X.; Li, Z. Short-Term Effect of El Niño-Southern Oscillation on Pediatric Hand, Foot and Mouth Disease in Shenzhen, China. *PLOS ONE* **2013**, *8*, e65585, doi:10.1371/journal.pone.0065585.
14. Cao, C.; Li, G.; Zheng, S.; Cheng, J.; Lei, G.; Tian, K.; Wu, Y.; Xie, X.; Xu, M.; Ji, W. Research on the environmental impact factors of Hand-Foot-Mouth Disease in Shenzhen, China using RS and GIS technologies. In Proceedings of the 2012 IEEE International Geoscience and Remote Sensing Symposium; IEEE, 2012; pp. 7240–7243.
15. Huang, J.; Chen, S.; Wu, Y.; Tong, Y.; Wang, L.; Zhu, M.; Hu, S.; Guan, X.; Wei, S. Quantifying the influence of temperature on hand, foot and mouth disease incidence in Wuhan, Central China. *Sci. Rep.* **2018**, *8*, 1–9, doi:10.1038/s41598-018-20318-z.
16. Fu, T.; Chen, T.; Dong, Z.-B.; Luo, S.-Y.; Miao, Z.; Song, X.-P.; Huang, R.-T.; Sun, J.-M. Development and comparison of forecast models of hand-foot-mouth disease with meteorological factors. *Sci. Rep.* **2019**, *9*, 15691–15697, doi:10.1038/s41598-019-52044-5.
17. Cheng, J.; Wu, J.; Xu, Z.; Zhu, R.; Wang, X.; Li, K.; Wen, L.; Yang, H.; Su, H. Associations between extreme precipitation and childhood hand, foot and mouth disease in urban and rural areas in Hefei, China. *Sci. Total. Environ.* **2014**, *497-498*, 484–490, doi:10.1016/j.scitotenv.2014.08.006.
18. Yang, B.; Lau, E.H.Y.; Wu, P.; Cowling, B.J. Transmission of Hand, Foot and Mouth Disease and Its Potential Driving Factors in Hong Kong. *Sci. Rep.* **2016**, *6*, 27500, doi:10.1038/srep27500.
19. Li, T.; Yang, Z.; Liu, X.; Kang, Y.; Wang, M. Hand-foot-and-mouth disease epidemiological status and relationship with me-teorological variables in Guangzhou, southern China, 2008–2012. *Rev. Inst. Med. Trop. Sao Paulo* **2014**, *56*, 533–539. doi:10.1590/s0036 46652014000600014.
20. Deng, T.; Huang, Y.; Yu, S.; Gu, J.; Huang, C.; Xiao, G.; Hao, Y. Spatial-Temporal Clusters and Risk Factors of Hand, Foot, and Mouth Disease at the District Level in Guangdong Province, China. *PLOS ONE* **2013**, *8*, e56943, doi:10.1371/journal.pone.0056943.
21. Dai, L.; Zhang, Y.; Wang, L.; Zheng, S.; Xu, W. Assessment of Carbon Density in Natural Mountain Forest Ecosystems at Northwest China. *Int. J. Environ. Res. Public Heal.* **2021**, *18*, 2098, doi:10.3390/ijerph18042098.
22. Abduwaiti, A.; Liu, X.; Yan, C.; Xue, Y.; Jin, T.; Wu, H.; He, P.; Bao, Z.; Liu, Q. Testing Biodegradable Films as Alternatives to Plastic-Film Mulching for Enhancing the Yield and Economic Benefits of Processed Tomato in Xinjiang Region. *Sustain.* **2021**, *13*, 3093, doi:10.3390/su13063093.
23. Fu, R.; Zhang, X.; Yang, D.; Cai, T.; Zhang, Y. The Relationship between Urban Vibrancy and Built Environment: An Empirical Study from an Emerging City in an Arid Region. *Int. J. Environ. Res. Public Heal.* **2021**, *18*, 525, doi:10.3390/ijerph18020525.
24. Liu, Y. Sustainable Development in Urban Areas: Contributions from Generalized Trade. *Sustain. Cities Soc.* **2020**, *61*, 102312, doi:10.1016/j.scs.2020.102312.
25. Song, C.; Yang, X.; Shi, X.; Bo, Y.; Wang, J. Estimating missing values in China's official socioeconomic statistics using progressive spatiotemporal Bayesian hierarchical modeling. *Sci. Rep.* **2018**, *8*, 10055, doi:10.1038/s41598-018-28322-z.
26. Bo, Y.-C.; Song, C.; Wang, J.-F.; Li, X.-W. Using an autologistic regression model to identify spatial risk factors and spatial risk patterns of hand, foot and mouth disease (HFMD) in Mainland China. *BMC Public Heal.* **2014**, *14*, 358, doi:10.1186/1471-2458-14-358.

27. Du, Z.; Huang, Y.; Lawrence, W.R.; Xu, J.; Yang, Z.; Lu, J.; Zhang, Z.; Hao, Y. Leading Enterovirus Genotypes Causing Hand, Foot, and Mouth Disease in Guangzhou, China: Relationship with Climate and Vaccination against EV71. *Int. J. Environ. Res. Public Heal.* **2021**, *18*, 292, doi:10.3390/ijerph18010292.
28. Fan, F.; Fan, W. Understanding spatial-temporal urban expansion pattern (1990–2009) using impervious surface data and landscape indexes: a case study in Guangzhou (China). *J. Appl. Remote. Sens.* **2014**, *8*, 083609, doi:10.1117/1.jrs.8.083609.
29. Peng, T.; Sun, C.; Feng, S.; Zhang, Y.; Fan, F. Temporal and Spatial Variation of Anthropogenic Heat in the Central Urban Area: A Case Study of Guangzhou, China. *ISPRS Int. J. Geo-Information* **2021**, *10*, 160, doi:10.3390/ijgi10030160.
30. Wang, J.F.; Li, X.H.; Christakos, G.; Liao, Y.L.; Zhang, T.; Gu, X.; Zheng, X.Y. Geographical detectors-based health risk assessment and its application in the neural tube defects study of the Heshun region, China. *Int. J. Geogr. Inf. Sci.* **2010**, *24*, 107–127, doi:10.1080/13658810802443457.
31. Ju, H.; Zhang, Z.; Zuo, L.; Wang, J.; Zhang, S.; Wang, X.; Zhao, X. Driving forces and their interactions of built-up land expansion based on the geographical detector – a case study of Beijing, China. *Int. J. Geogr. Inf. Sci.* **2016**, *30*, 2188–2207, doi:10.1080/13658816.2016.1165228.
32. Qi, X.; Si, Z.; Zhong, T.; Huang, X.; Crush, J. Spatial determinants of urban wet market vendor profit in Nanjing, China. *Habitat Int.* **2019**, *94*, 102064, doi:10.1016/j.habitatint.2019.102064.
33. Xie, W.; Li, X.; Jian, W.; Yang, Y.; Liu, H.; Robledo, L.; Nie, W. A Novel Hybrid Method for Landslide Susceptibility Mapping-Based GeoDetector and Machine Learning Cluster: A Case of Xiaojin County, China. *ISPRS Int. J. Geo-Information* **2021**, *10*, 93, doi:10.3390/ijgi10020093.
34. Zhang, J.; Yu, L.; Li, X.; Zhang, C.; Shi, T.; Wu, X.; Yang, C.; Gao, W.; Li, Q.; Wu, G. Exploring Annual Urban Expansions in the Guangdong-Hong Kong-Macau Greater Bay Area: Spatiotemporal Features and Driving Factors in 1986–2017. *Remote. Sens.* **2020**, *12*, 2615, doi:10.3390/rs12162615.
35. Wang, J.; Hu, T.; Sun, D.; Ding, S.; Carr, M.J.; Xing, W.; Li, S.; Wang, X.; Shi, W. Epidemiological characteristics of hand, foot, and mouth disease in Shandong, China, 2009–2016. *Sci. Rep.* **2017**, *7*, 1–9, doi:10.1038/s41598-017-09196-z.
36. Qi, L.; Tang, W.; Zhao, H.; Ling, H.; Su, K.; Zhao, H.; Li, Q.; Shen, T. Epidemiological Characteristics and Spatial-Temporal Distribution of Hand, Foot, and Mouth Disease in Chongqing, China, 2009–2016. *Int. J. Environ. Res. Public Heal.* **2018**, *15*, 270, doi:10.3390/ijerph15020270.
37. Song, C.; He, Y.; Bo, Y.; Wang, J.; Ren, Z.; Yang, H. Risk Assessment and Mapping of Hand, Foot, and Mouth Disease at the County Level in Mainland China Using Spatiotemporal Zero-Inflated Bayesian Hierarchical Models. *Int. J. Environ. Res. Public Heal.* **2018**, *15*, 1476, doi:10.3390/ijerph15071476.
38. Tian, L.; Liang, F.; Xu, M.; Jia, L.; Pan, X.; Clements, A.C.A. Spatio-temporal analysis of the relationship between meteorological factors and hand-foot-mouth disease in Beijing, China. *BMC Infect. Dis.* **2018**, *18*, 158, doi:10.1186/s12879-018-3071-3.
39. Yi, S.; Wang, H.; Yang, S.; Xie, L.; Gao, Y.; Ma, C. Spatial and Temporal Characteristics of Hand-Foot-and-Mouth Disease and Its Response to Climate Factors in the Ili River Valley Region of China. *Int. J. Environ. Res. Public Heal.* **2021**, *18*, 1954, doi:10.3390/ijerph18041954.
40. Huang, J.; Wang, J.; Bo, Y.; Xu, C.; Hu, M.; Huang, D. Identification of Health Risks of Hand, Foot and Mouth Disease in China Using the Geographical Detector Technique. *Int. J. Environ. Res. Public Heal.* **2014**, *11*, 3407–3423, doi:10.3390/ijerph110303407.
41. Bie, Q.; Qiu, D.; Hu, H.; Ju, B. Spatial and Temporal Distribution Characteristics of Hand-Foot-Mouth Disease in China. *J. Geo-information Sci.* **2010**, *12*, 380–384, doi:10.3724/sp.j.1047.2010.00380.
42. Zhu, Q.; Hao, Y.T.; Yu, S.C. Epidemiology Characteristic and Spatial Time Analysis of Hand, Foot, and Mouth Disease in Guangdong Province from 2008 to 2010. *Mod. Prev. Med.* **2011**, *38*, 1824–1831.
43. Huang, J.-X.; Wang, J.-F.; Li, Z.-J.; Wang, Y.; Lai, S.-J.; Yang, W.-Z. Visualized Exploratory Spatiotemporal Analysis of Hand-Foot-Mouth Disease in Southern China. *PLOS ONE* **2015**, *10*, e0143411, doi:10.1371/journal.pone.0143411.
44. Kim, B.I.; Ki, H.; Park, S.; Cho, E.; Chun, B.C. Effect of Climatic Factors on Hand, Foot, and Mouth Disease in South Korea, 2010-2013. *PLOS ONE* **2016**, *11*, e0157500, doi:10.1371/journal.pone.0157500.
45. Yi, X.; Chang, Z.; Zhao, X.; Ma, Y.; Liu, F.; Xiao, X. The temporal characteristics of the lag-response relationship and related key time points between ambient temperature and hand, foot and mouth disease: A multicity study from mainland China. *Sci. Total. Environ.* **2020**, *749*, 141679, doi:10.1016/j.scitotenv.2020.141679.
46. Chen, C.; Lin, H.; Li, X.; Lang, L.; Xiao, X.; Ding, P.; He, P.; Zhang, Y.; Wang, M.; Liu, Q. Short-term effects of meteorological factors on children hand, foot and mouth disease in Guangzhou, China. *Int. J. Biometeorol.* **2013**, *58*, 1605–1614, doi:10.1007/s00484-013-0764-6.
47. Huang, Y.; Deng, T.; Yu, S.; Gu, J.; Huang, C.; Xiao, G.; Hao, Y. Effect of meteorological variables on the incidence of hand, foot, and mouth disease in children: a time-series analysis in Guangzhou, China. *BMC Infect. Dis.* **2013**, *13*, 134, doi:10.1186/1471-2334-13-134.
48. Liao, Y.; Ouyang, R.; Wang, J.; Xu, B. A study of spatiotemporal delay in hand, foot and mouth disease in response to weather variations based on SVD: a case study in Shandong Province, China. *BMC Public Heal.* **2015**, *15*, 71, doi:10.1186/s12889-015-1446-6.
49. Bo, Z.; Ma, Y.; Chang, Z.; Zhang, T.; Liu, F.; Zhao, X.; Long, L.; Yi, X.; Xiao, X.; Li, Z. The spatial heterogeneity of the associations between relative humidity and pediatric hand, foot and mouth disease: Evidence from a nation-wide multicity study from mainland China. *Sci. Total. Environ.* **2020**, *707*, 136103, doi:10.1016/j.scitotenv.2019.136103.

50. Luo, C.; Ma, Y.; Liu, Y.; Lv, Q.; Yin, F. The burden of childhood hand-foot-mouth disease morbidity attributable to relative humidity: a multicity study in the Sichuan Basin, China. *Sci. Rep.* **2020**, *10*, 1–10, doi:10.1038/s41598-020-76421-7.
51. Dong, W.; Li, X.; Yang, P.; Liao, H.; Wang, X.; Wang, Q. The Effects of Weather Factors on Hand, Foot and Mouth Disease in Beijing. *Sci. Rep.* **2016**, *6*, srep19247, doi:10.1038/srep19247.
52. Yin, F.; Zhang, T.; Liu, L.; Lv, Q.; Li, X. The Association between Ambient Temperature and Childhood Hand, Foot and Mouth Disease in Chengdu, China: A Distributed Lag Non-linear Analysis. *Sci. Rep.* **2016**, *6*, 27305, doi:10.1038/srep27305.
