# Supplementary material for: Estimation of Undetected Asymptomatic COVID-19 Cases in South Korea Using a Probabilistic Model
Source: Int J Environ Res Public Health. 2021 May 6;18(9):4946. doi: 10.3390/ijerph18094946 (PMC8124955; doi:10.3390/ijerph18094946)
Supplement: Supplementary file 1 [file ijerph-18-04946-s001.zip › 5/ijgi-1113680-english.pdf]

International Journal of
Geo-Information

MDPI

Article

On the Representativeness of OpenStreetMap for the Evaluation of Country Tourism Competitiveness

Alexander Bustamante [1,2,*], Laura Sebastia [1] and Eva Onaindia [1]

[1] Valencia Research Institute for Artificial Intelligence (VRAIN), Universitat Politècnica de València, 46022 Valencia, Spain; lsebastia@dsic.upv.es (L.S.); onaindia@dsic.upv.es (E.O.)
[2] Facultad de Ingeniería, Universidad del Magdalena, Santa Marta 470001, Colombia
* Correspondence: albus2@doctor.upv.es

Abstract: Since 2007, the World Economic Forum (WEF) has issued data on the factors and policies that contribute to the development of tourism and competitiveness across countries worldwide. While WEF compiles the yearly report out of data from governmental and private stakeholders, we seek to analyze the representativeness of the open and collaborative platform OpenStreetMap (OSM) to the international tourism scene. For this study, we selected eight parameters indicative of the tourism development of each country, such as the number of beds or cultural sites, and we extracted the OSM objects representative of these indicators. Then, we performed a statistical and regression analysis of the OSM data to compare and model the data emitted by WEF with data from OSM. Our aim is to analyze the tourist representativeness of the OSM data with respect to official reports to better understand when OSM data can be used to complement the official information and, in some cases, when official information is scarce or non-existent, to assess whether the OSM information can be a substitute. Results show that OSM data provide a fairly accurate picture of official tourism statistics for most variables. We also discuss the reasons why OSM data is not so representative for some variables in some specific countries. All in all, this work represents a step towards the exploitation of open and collaborative data for tourism.

Keywords: collaborative data; open data analysis; tourism competitiveness; tourism statistics

check for updates

Citation: Bustamante, A.; Sebastia, L.; Onaindia, E. On the Representativeness of OpenStreetMap for the Evaluation of Country Tourism Competitiveness. *ISPRS Int. J. Geo-Inf.* **2021**, *10*, 301. https://doi.org/10.3390/ijgi10050301

Academic Editors: A. Yair Grinberger and Wolfgang Kainz

Received: 31 January 2021
Accepted: 1 May 2021
Published: 5 May 2021

Publisher's Note: MDPI stays neutral with regard to jurisdictional claims in published maps and institutional affiliations.

Copyright: © 2021 by the authors. Licensee MDPI, Basel, Switzerland. This article is an open access article distributed under the terms and conditions of the Creative Commons Attribution (CC BY) license (https://creativecommons.org/licenses/by/4.0/).

1. Introduction

Understanding tourism competitiveness of countries has become a key aspect to destinations. Tourism has shown to highly impact the social-cultural environment and economic growth of a country [1]. Therefore, countries invest a huge amount of money to collect data related to tourism industries, attractions, infrastructure, and so on. In addition, several organizations, such as the World Economic Forum (WEF), collect and analyze data from several countries in order to determine how competitive countries are in the tourism sector. WEF is a well-known organization devoted to the dissemination of world-wide data that also emit data which show the state of tourism competitiveness of countries. In a broad way, WEF is an organization for public–private cooperation that engages the foremost political, business, and other leaders of society to shape global, regional, and industry agendas [2]. WEF has published the Travel & Tourism Competitiveness Report since 2007.

The analysis of tourism on the economies typically relies on official tourism statistics provided by governments and institutions. Parallel to the dissemination of official statistical data, Information and Communication Technologies (ICT) particularly in general and mobile and social network technologies have opened a new door, and data coming from these new sources are used to analyze tourism, as shown in several recent studies [3,4]. These online tools, social networks, and collaborative platforms have emerged as a relevant data source to understand tourism behavior and traveling trends [5–8] to create accurate tourist profiles [9,10] and to elicit a picture of the tourism industry [11].

A remarkable example of these new sources is the free mapping service offered by the collaborative mapping platform OpenStreetMap (OSM) [12], with around 37,000 active contributors during a typical month. OSM is claimed to be the largest freely and openly accessible database of geographic data in the world [13]. It emerges as an alternative to the restricted use of other mapping services, such as Google Maps. One argument in favor of Google Maps could be the wide range of advanced features that it offers (street-view images, multimodal navigation, social recommendations, etc.). However, some services based on the OSM database also provide them. For example, Mapillary (www.mapillary.com (accessed on 13 January 2021)) is a service for crowdsourcing street-level photographs using smartphones and computer vision (with more than 1400 million geotagged photographs) or OpenRouteService (www.openrouteservice.org (accessed on 1 November 2020)) which provides multimodal navigation services, among other geography-related features (such as geocoding, isochrones, time-distance matrix, etc.). Numerous applications based on OSM can be found in the list of OSM-based services (https://wiki.openstreetmap.org/wiki/List_of_OSM-based_services (accessed on 30 May 2020)), some of them related to tourism services. Additionally, OsmAnd (https://osmand.net/ (accessed on 25 July 2020)) and MapOut (https://mapout.app/ (accessed on 21 December 2020)) provide some tourism-related services, such as offline mobile map viewing, navigation, POI searching, and tour management. Other works describe applications for e-bike navigation [14], the construction of sidewalk geometries for wheelchair users [15], or the evaluation of the impact of post-disaster recovery in tourist destinations [16].

This paper presents an exploratory analysis of the OSM data set and compares the obtained insight with the publicly available data of the tourism competitiveness provided by WEF for a group of about 130 countries worldwide. Specifically, we are interested in studying the representativeness and reliability of tourism-related data found in an open and collaborative platform, such as OSM; that is, our aim is to analyze how well the OSM data reflect the actual tourism competitiveness data from the WEF across eight indicators. We will investigate the relationship between OSM and the WEF tourism competitiveness report through *regression models* to study the relationship between the data collected from OSM for an indicator and the official values of such indicators in WEF.

Sometimes, official information is difficult to find, it is not possible to access it at the desired level of granularity, or it is not easily upgradeable. As explained above, social networks and collaborative platforms have emerged as a relevant and alternative data source that can be used in these cases. Therefore, in this paper, we will examine the tourism-related information of OSM and determine in which cases OSM is a reliable alternative data source to WEF and can be used for forecasting. In a nutshell, given the common acknowledgement that OSM is a powerful and user-friendly geo-data platform extensively used for tourism purposes, our aim is to give response to the following question: does OSM provide an accurate picture of the studied components of tourism competitiveness?. That is, we are interested in analyzing whether the elements mapped in OSM can be used to infer some WEF data. If the answer is yes, OSM data can be used to, for example, analyze the same components of tourism competitiveness at a more specific area (not necessarily at a country level, as WEF provides). Otherwise, we will analyze which aspects make this task difficult.

Given the nature of the OSM data, which is mainly related to attractions, accommodation, and infrastructure, the components of tourism competitiveness that will be analysed in this paper are those concerned to the endowments of these elements in each country. Therefore, other tourism competitiveness aspects, such as the dimension of touristic flows, pricing policies, destination marketing, the reputation of the place, and so forth are out of the scope of the analysis presented in this paper. Specifically, we will focus on attractions and accomodation, which are related to eight WEF indicators.

We will carry out an statistical and regression analysis of eight different tourism indicators over 133 countries from two different points of view: (1) considering all the countries as a whole, and (2) splitting the countries into three groups according to their

ICT level given by the ICT readiness pillar of WEF. The reason for this double analysis is that, according to [17], the status of a country's ICT services will determine, for instance, the success of a Volunteered Geographic Information (VGI) initiative or the expected growth in the years to come. Moreover, previous investigations [18] found that although OSM has had great global success, there is still a clear difference in the volume of contributed data between affluent and poorer communities. Therefore, we will also examine whether the country ICT level is an influential factor in the relation between OSM and WEF. We hypothesize that a higher ICT level would imply a better representativeness of OSM with respect to official data sources, given that technology in these countries is more easily accessible and hence users will participate more intensely in collaborative platforms (OSM, in this case).

An additional aspect that must be mentioned is that the two data sources we handle in this work, WEF and OSM, are of a very different nature, and thereby it is not always possible to measure exactly the same concept in both sources. For example, it could be the case that a particular variable is measured in different units in OSM and WEF, or it is not possible to find an exact element in OSM to a given WEF indicator. In both cases, some approximations have been computed, and we will discuss the limitations we have found regarding this.

Our Research Questions can be summarized in the following:

- **Question 1:** Can OSM data be used as a reliable alternative source to extract the WEF tourism indicators?
- **Question 2:** Is it possible to model the trend reflected in WEF tourism indicators with OSM data?
- **Question 3:** Does the ICT level of a country influence the models built to answer Question 2?

The paper is structured in the following sections. Section 2 gives an overview of previous work that uses OSM data in several contexts. Section 3 describes the WEF and OSM data sources used in our analysis. Section 4 describes the analysis we performed with WEF and OSM data, Section 5 presents the outcomes of this analysis, and Section 6 discusses these results. Finally, in the last section, we outline the conclusions and future research directions.

2. Related Work

Volunteered Geographic Information (VGI) [19] systems have emerged as an answer to the need for open and easy-to-use geographic data and as an alternative to Commercial Geographic Information systems which impose restrictions on the use of the data. Technological advancement has fostered the emerging role of the citizen as a source of data. Citizen sensing has dramatically affected mapping and map use, impacting on routine daily life activities, such as gaming and tourism, as well as on science and technology more generally [20]. Due to the proliferation of location-aware devices and the opportunities of Web 2.0, it is now possible for citizens to easily acquire geographical information, which may dramatically reduce the cost of map acquisition [21] and also allows to usually have up-to-date maps [22]. Additionally, it can become a tool for the empowerment of marginalized individuals and social groups [23].

However, citizen-derived data are also often of varied quality and trust levels. For example, the data generated may be poorly described and associated with little metadata. Additionally, there are other considerations in the use of VGI, including ownership rights, as well as privacy, legal, and ethical issues [20].

OpenStreetMap (OSM) is one of the most well-known VGI projects. The crowdsourced approach of OSM derives its success from citizens mapping and collecting data and information about their locality [13]. Features being mapped include the location of garbage cans, pedestrian crossings, land cover types, shops, education facilities, to government buildings, roads, and river networks. All data in the OSM database can be downloaded for free in a variety of spatial data formats. Additionally, a number of open source tools are available to

process this data and produce other formats [21]. The OSM project counts on experienced volunteers that spend time checking, updating, and improving OSM data. The process of validation aims to ensure the completeness and quality of data. Nevertheless, the fact that the OSM is either non-commercial or governmental and that validation is carried out by volunteers sometimes puts the validation of data in question [20].

In order to alleviate the doubts concerning the quality and precision of OSM data, a large number of works have investigated the robustness and validity of OSM in several fields, like in environmental epidemiological and exposure assessment studies [24]. This study compared OSM and Governmental Major Road Data in three different regions: Massachusetts (USA), Bern (Switzerland), and Beer-Sheva (South Israel). This investigation found that OSM data was fairly complete and accurate in all regions, and that the results in all regions were robust, with Massachusetts showing the best fit (R^2 of 0.93).

In the same direction, the work [25] evaluates the quality of OSM data with respect to its suitability for a certain application, specifically for pedestrian navigation. The analysis compares routes calculated with OSM data and routes done with the German topographic data set, using accessibility and length of routes as quality criteria. The study concludes that OSM is fairly accurate on average within about six meters of the position recorded by the Ordnance Survey, and with approximately 80% overlap of motorway objects between the two datasets.

Another relevant work is about comparing the accuracy of the OSM data on land use in four German metropolitan areas versus the Global Monitoring for Environment and Security Urban Atlas as a reference [26]. The study reveals the suitability of using OSM as an alternative complementary source for extracting land use information as it also highlights the potential of collaboratively collected land use features by mappers.

There have also been attempts to evaluate the quality of OSM—in terms of completeness, and positional and semantic accuracy in the cultural sector. In [27], authors show that the number of museums of Italy mapped in OSM accounts for 86% of the official total. In addition, OSM has records of positional and semantic information of 39% of the museums overall. The study also states that for 77.7% of the museums, the location reported by OSM is less than 150 me away from the actual location of the museum. Likewise, 90% of the museums have a similar denomination in OSM and in the official sources.

OSM has also been used to predict socio-economic indicators (sustainability, human development, vulnerability, risk, resilience, and climate change adaptation) for municipalities. In [28], authors present an interesting study that highlights the prospects of OSM to analyze interdisciplinary topics and factors like social cohesion, and provide meaningful insight into the spatial differences in social, environmental, or economic inequalities. One of the conclusions of this study is that further research is needed to determine the impact of regional and international differences in user contributions on the outputs.

In the specific field of tourism, we found some works that use OSM in analysis tasks. For instance, in [29], a framework for the assessment of the quality of OpenStreetMap is depicted. The approach analyses several quality measures, such as completeness, compliance, consistence, granularity, richness, and trust of OSM tags in Spain. The authors conclude that the current status of the Spanish OSM data can be considered satisfactory in some indicators (compliance and consistency), while in some others (granularity and richness) it should be improved. For tourism POIs, some elements are still missing. For instance, shopping and amenity destinations should include opening hours, phone numbers, and so forth, and specific categories like restaurants or hotels should include more detailed information (prices, cuisine, stars, etc.).

In the same way, ref. [30] evaluated the consistency of the information contained in the Compendium of Tourism Statistics of the World Tourism Organization with respect to the information published in OSM, especially information on places of accommodation, food and beverages, and travel agencies. Among the results shown in this paper, the high correlation that exists between the data from both sources with respect to information

on accommodation (0.81), food and beverage sites (0.87), and travel agencies (0.82) is remarkable.

In [31], the authors exposed how they used OSM data along with data from official sources and other platforms with the objective of identifying spatial patterns in park popularity in the state of Victoria, Australia. Statistically significant correlations were found between official data and OSM data, indicating that OSM vertices' density in a given area can be used to infer the number of visitors.

Finally, in [32], a methodology for computing composite indicators derived from OSM data as an alternative to statistical offices was presented. To demonstrate its use, they applied this methodology to a number of indicators used for real estate valuation of properties in Italy. Among these indicators, they considered a number of sites of historical relevance and a number of nearby hotels and hotel-related features.

3. Data

This section describes firstly the tourism indicators from the WEF data sources which will be used in our analysis. Subsequently, we overview some basic aspects of OSM, and we define the concept of direct and indirect variables.

3.1. WEF

Tourism competitiveness is regarded as the set of regulations, infrastructure, and resources that enable the sustainable development of the Travel & Tourism (T&T) sector. For our analysis, data on tourism competitiveness were retrieved from sources of the WEF organization. Particularly, we focus on the Travel & Tourism Competitiveness Report, of which the first edition was published in 2007. This report is based on secondary data from various international organisms and provides engaged leaders in T&T an in-depth analysis of tourism competitiveness of a large number economies across the world. The 2017 edition covers 141 economies and features data about 14 key factors and policies, also called pillars, that enable the sustainable development of the T&T sector and contribute to the development and tourism competitiveness of a country [33].

A pillar measures the strengths and weaknesses of a country in a scale of 1 (bad) to 7 (excellent), and it is based on a set of 90 indicators that are collected either from surveys or official national statistics. These indicators are mainly extracted from two sources:

- Survey indicators: These are data derived from responses to the WEF's Executive Opinion Survey that capture the opinions of business leaders around the world on a broad range of topics. These indicators are aimed to measure critical concepts to complement the traditional sources of statistics and provide a more accurate assessment of drivers of economic development. Survey indicators range in value from 1 to 7 (1: the lowest negative perception; 7: the highest positive perception).
- Hard data indicators: These are data which objectively represent the state of some resource or abstract concept, and they are often measured by official international or national organizations (e.g., number of stadiums, airports, ATMs, etc.). These indicators are normalized to a scale of 1 to 7 in order to align them with the Executive Opinion Survey's results

WEF uses the survey and hard data indicators to shape the 14 pillars, which are then compiled into a global Travel and Tourism Competitiveness index that represents how viable a country is within the T&T sector.

For our analysis, we opted for selecting indicators that measure tangible aspects that are rather directly perceived by tourists and can be determinant in the selection of a particular destination. The nine indicators selected as our study variables are shown in Table 1. The second column of Table 1 shows the indicator name alongside a brief description. The first column is the pillar that the indicator belongs to. The third column indicates the name of the variable in our study. The fourth column shows whether the indicator is a hard data indicator (H) or a survey indicator (S). Finally, the fifth column is explained in Section 3.2 as it is directly involved with the retrieval of the OSM data.

As can be observed, each variable is drawn from only one WEF indicator except for the variable WHS which stems from two indicators, the Number of World Heritage cultural sites and Number of World Heritage natural sites. The reason is that outstanding universal sites qualify both as cultural and natural sites.

Table 1. Tourism competitiveness variables. Source for indicators can be (S)urvey or (H)ard data. Indicators can be computed (D)irectly or (I)ndirectly from OSM data.

Pillar	Indicator	Variable	Source	Comp.
Tourist service infrastructure	***Presence of major car rental companies***<br>This indicator measures the presence of seven major car rental companies: Avis, Budget, Europcar, Hertz, National Car Rental, Sixt and Thrifty. For each country WEF counts how many of these companies operate via online research.	CAR	H	D
Tourist service infrastructure	***ATMs per adult population***<br>Number of automated teller machines (ATMs) per adult population of 100,000.	ATM	H	D
Tourist service infrastructure	***Hotel rooms***<br>Number of hotel rooms per population of 100.	HOT	H	I
Health and hygiene	***Hospital beds***<br>Hospital beds include inpatient beds available in public, private, general and specialized hospitals and rehabilitation centers. In most cases, beds for both acute and chronic care are included, per population of 10,000.	HBD	H	I
Cultural resources and business travel	***Number of World Heritage cultural sites***<br>Number of properties that the World Heritage Committee considers as having outstanding universal cultural value.	WHS	H	D
Natural resources	***Number of World Heritage natural sites***<br>Number of properties that the World Heritage Committee considers as having outstanding universal natural value.		H	D
Air Transport Infrastructure	***Airport density***<br>Number of airports with at least one scheduled flight per million of urban population.	AIR	H	D
Cultural resources and business travel	***Cultural and entertainment tourism digital demand***<br>This indicator measures the total online search volume related to the following cultural brandtags: Historical Sites, Local People, Local Traditions, Museums, Performing Arts, UNESCO, City Tourism, Religious Tourism, Local Gastronomy, Entertainment Parks, Leisure Activities, Nightlife and Special Events.	CDD	H	I
Natural resources	***Attractiveness of natural assets***<br>To what extent do international tourists visit your country mainly for its natural assets (i.e., parks, beaches, mountains, wildlife, etc.)? (1 = not at all; 7 = to a great extent).	NAT	S	I

All in all, we have a total of eight variables covering the most relevant aspects of tourism competitiveness that influence the tourist perception of the country. The selected indicators embody aspects that have a major impact on a tourist trip, such as the presence of car rental companies, the availability of accommodation, or the number of cultural/natural sites. Some of the variables in Table 1 refer to elements related to the tourism infrastructure, while others are intended to survey the tourism attractiveness of the country. The values of the indicators for every country are extracted from the Travel & Tourism Competitiveness Report, which is directly available and downloadable in electronic format [33].

3.2. OSM

In this section, we will describe the elements of OSM that will be used in our analysis. Objects drawn on a OSM map are called map features, but these map features are not a

tourism-specific site. However, the aggregation of web maps and user-generated content is fed with a broad variety of metadata (OSM tags) that provide valuable tourism information, like the location of accommodation, food establishments, or tourist attractions. Hence, we are able to collect information about *tourism competitiveness* within a geographical or administrative area, such as a country [34].

In this sense, Table 2 shows a list of five keys alongside a brief textual description of each one. At the end of the description, we show some examples of tags that represent a particular map feature. For instance, a bar is an element tagged in OSM as `amenity = "bar"`, and a museum is tagged as `tourism = "museum"`. An exhaustive list of the map's features can be found in the project web page (https://wiki.openstreetmap.org/wiki/Map_Features (accessed on 28 November 2020)).

Table 2. Keys of OSM to represent tourism elements.

Key	Description
Amenity	This key is used to map facilities used by visitors and residents. For example: bar (`amenity = "bar"`), fast food (`amenity = "fast_food"`).
Aeroway	This is mainly related to aerodromes `aeroway = "aerodrome"`, airfields `aeroway = "airfield"`, and other ground facilities that support the operation of airplanes and helicopters.
Historic	This key is used to describe various historic places. For example: archeological sites `historic = "archeological_site"`, ruins `historic = "ruin"`, etc.
Leisure	This key is used to tag leisure and sports facilities, such as water parks `leisure = "water_park"` and fitness centers `leisure = "fitness_center"`.
Tourism	It represent places and things of specific interest to tourists including places to see, places to stay, and things and places providing information and support to tourists. A museum is one of the possible values of this tag (`tourism = "museum"`).

There are no specific guidelines for the type of tags to define a map feature, except that they must always be string values. Although OSM contributors are allowed to use free-style attributes to define features, there exists a wiki page (https://wiki.openstreetmap.org/wiki/How_to_map_a (accessed on 30 June 2020)) that shows recommended combinations of tags to qualify an object. Tags are used to query and retrieve any object defined in OSM.

The two data sources we handle in this work, WEF and OSM, are of a very different nature, and thereby it is not always possible to measure exactly the same concept in both sources. Hence, a relevant aspect that must be considered in the data extraction is whether or not the OSM value of a particular variable is given in the same measurement units as the value of the corresponding indicator in WEF, which gives rise to:

- *Direct variables:* This is the case when the variable is measured in the same terms as the WEF indicator. For instance, the value retrieved from OSM for the variable CAR is the number of establishments that provide such particular service, as are the values obtained from WEF for the indicator "Presence of major can rental companies".
- *Indirect variables:* this is the case when the variable in OSM is measured in units other than the ones used in the WEF indicator. For instance, the value of the WEF indicator "Attractiveness of natural assets" is a value within the range 1 to 7 that comes from a survey, while the value we obtain from OSM for variable NAT is the number of natural beauty spots.

We can observe in the fifth column of Table 1 that variables are classified as direct (D) or indirect (I).

4. Methods

Our aim is to analyze how well the OSM data approximate the values of the WEF indicators and thus determine whether OSM is a reliable data source to evaluate tourism competitiveness.

Figure 1 shows the workflow followed in our analysis. First, the *Travel & Tourism Competitiveness Report 2017* was reviewed and, as explained in Section 3, eight variables related with attractions and accommodation infrastructure were selected. The data for each country corresponding to these variables in 2017 was downloaded from WEF. Then, the OSM database was studied, and the most appropriate data for each variable was extracted in 2017 (this will be explained in Section 4.1). Both data from WEF and OSM were combined to build some statistical models, as shown in Section 4.2. For evaluating these models, the following steps were performed: (1) OSM data were downloaded in 2019, (2) these new OSM data were used to infer the WEF values, by using the regression models and (3) the inferred values were compared to the actual WEF values in the *Travel & Tourism Competitiveness Report 2019*.

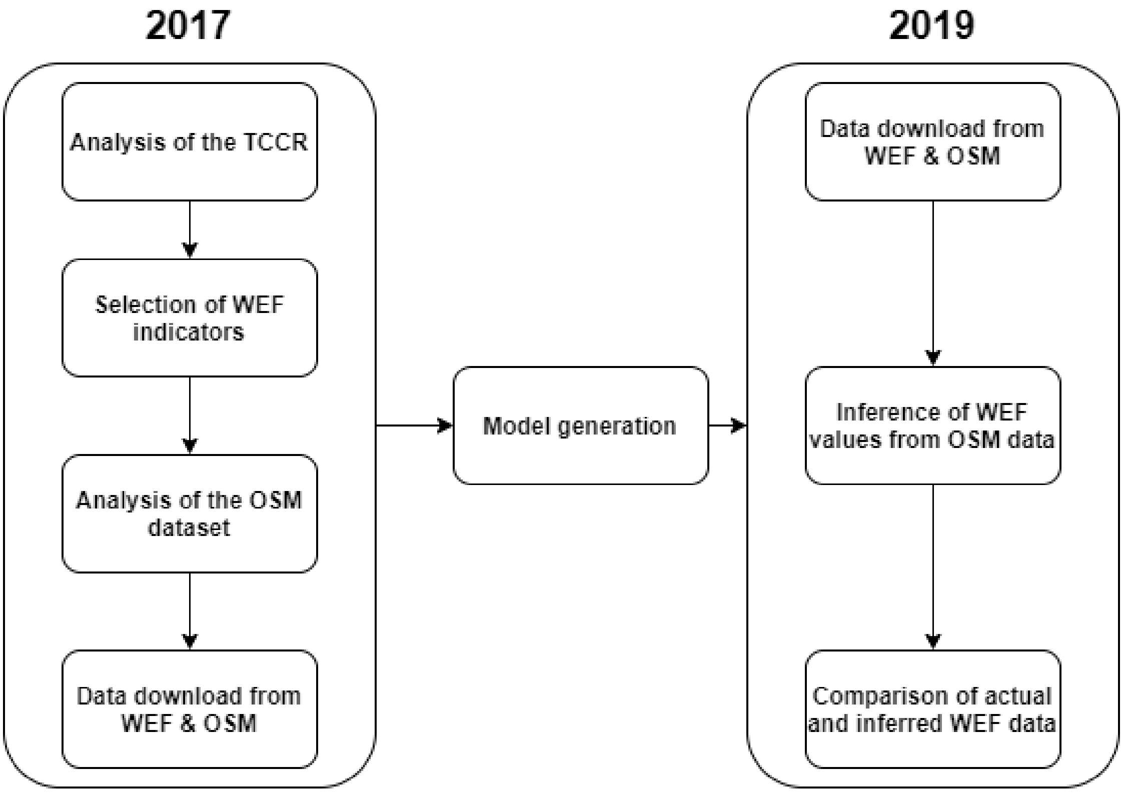

Figure 1. Methodological process.

4.1. OSM Data Processing

We follow a straightforward two-step process to retrieve the OSM data for each variable:

- Step 1. We identify the specific combination of OSM tags that better capture the meaning of the variable. As an example, for the WEF variable CAR (car rental companies), we selected the tags `amenity`, `name`, and `operator`, since this particular combination enables knowledge of whether a specific car rental company is present in a geographical area.
- Step 2. We query the OSM tags selected in Step 1 through the Overpass API (The Overpass API is an API that serves up custom selected parts of the OSM map data by search criteria, such as location, type of objects, tag properties, proximity, or combinations of them (https://wiki.openstreetmap.org/wiki/Overpass_API/Language_Guide (accessed on 3 July 2020))) within the delimited geographical area of a specific country. Algorithm 1 shows a query to retrieve the car rental companies in Colombia. Once the objects of type `amenity = "car_rental"` are retrieved, we can apply the query

Algorithm 1: Excerpt of Overpass code.

```
(
area['ISO3166-1:alpha2'~=~"CO"][admin_level=2];
->.a;
   (
     node[amenity~=~"car_rental"](area.a);
     way[amenity~=~"car_rental"](area.a);
     rel[amenity~=~"car_rental"](area.a);
    )
;out center;)
```

`name = "Europcar"` or the query `operator = "Europcar"` over the retrieved objects so as to find out if the car rental company `Europcar` is present in Colombia.

In some cases, it is necessary to apply two or more queries as described in Step 2 to retrieve the value of a particular variable. Aggregation, arithmetic operations, or more complex operations are needed to approximate the value of some variables with OSM data. Both Overpass queries and the subsequent approximation operations have been implemented in Python.

In the following, we explain the tags used to retrieve the variables, as well as the operations needed in some cases to approximate the value of the WEF indicator.

CAR. We first retrieve all features that match the tag `amenity = "car_rental"`, and then we check whether at least one of the features matches the name of the car rental company (e.g., `name = "Avis"` or `operator = "Avis"`).

ATM. The number of features in OSM that match the tag `amenity = "atm"` is relatively low and usually refers only to bank entities. There exist, however, ATMs in shopping malls or other types of establishments that are retrievable via the tag `atm = "yes"`. We estimated one ATM per feature tagged `amenity = "atm"` because it indicates that the object *is an actual ATM*, whereas we estimated two ATMs per feature tagged `atm = "yes"` because it indicates that the place *has some ATMs*. Finally, in order to calculate the number of ATMs per adult population of 100,000, we used the value of the population between 15 and 64 years that provided the World Bank (http://www.worldbank.org/ (accessed on 21 October 2020)).

HOT. The number of hotel rooms in OSM is extracted by finding the features tagged `tourism = "hotel"` and then using the value of the tag `rooms` of such features, which is an integer value that denotes the number of rooms of a hotel. Unfortunately, the tag `rooms` is not present in most of the hotel features, which is the reason why we opted for it, considering the number of hotels as the OSM value for variable HOT.

HBD. Similarly to variable HOT, we recover the value of HBD by using the tag `amenity = "hospital"` and then querying the tag `bed` over the `hospital` features to obtain the number of beds. As it happens with variable HOT, only the `hospital` features of a small group of 19 countries (e.g., United States, Saudi Arabia, France, United Kingdom, Indonesia, Germany, etc.) include the key `bed`. Therefore, we opted for it considering the number of hospitals as the OSM value for variable HBD.

WHS. This direct variable represents the number of natural and cultural sites of a country that are selected by UNESCO as World Heritage. The value of WHS is retrievable through the tags `heritage = "1"` or `heritage:operator= "World Heritage Centre (whc)"`, which return the number of OSM features tagged as World Heritage sites.

AIR. Given that the number of flights is not available in OSM, we focused exclusively on the number of airports using the tag `aeroway = "aerodrome"`. More particularly, we are interested in airports open to the general public that are recognized by the International Air Transport Association (IATA = "<air_code>") or International Civil Aviation Organization (ICTAO = "<air_code>"), where <air_code> is the airport code given by `IATA` or `ICTAO`, respectively.

CDD. We assume that the more historical, cultural, and leisure attractions of a country, the more online searches will yield. For variable CDD, we count the number of features that are categorized as museums (`tourism = "museum"`); historic places (e.g., `historic = "aircraft"|"aqueduct"`) and arts centers (`amenity = "arts_centre"`); theme parks, aquariums and water parks (`tourism = "theme_park"`, `tourism = "aquarium"`, `leisure = "water_park"`); and religious places (e.g., `building = "cathedral"|"chapel" |''church''`, `amenity = "place_of_worship"`), amongst others. For the case of features that represent a `building`, we also query the existence of the keys `historic` or `tourism` in the feature in order to ensure the building is categorized as a tourist attraction.

NAT. For this indirect variable, we recovered the number of places of tourist interest for their natural beauty, such as national parks (e.g., `boundary = "national_park"`), as well as map features that have both the keys `natural` and `tourism`. Examples of tags are `tourism = "attraction"` and `natural = "water"`, `natural = "bay"`, `natural = "cliff"`, `natural = "volcano"`, etc.

4.2. Statistical Analysis

In this section we will carry out a statistical analysis and investigate the relationship between the values of the official WEF indicators and the data collected from OSM. In particular, first, a linear correlation analysis between each WEF variable (denoted as variable-WEF) and its counterpart in OSM (denoted as variable-OSM) is performed, and then regression models are calculated to measure how well the OSM data fits the WEF indicators. In order to obtain the most accurate model that fits the data at hand, linear and non-linear regression models were tested, like multiplicative, double-squared, and squared-root-Y models, among others (see Table 3). These regression models are an alternative when linear models do not achieve the desired accuracy, or when the phenomenon under study has a behavior that can be considered non-linear. To assess the accuracy of each model, the determination coefficient (R^2), which measures the proportion of variation of the dependent variable (variable-WEF), is explained by the independent variable, and (variable-OSM) is calculated. Finally, the models are tested with new data from 2019 and the values predicted by these models are compared with the actual WEF values. These analyses will help us to answer our Research Questions 1 and 2.

Table 3. Models used in our analysis.

Model	Equation	Transformation on Y	Transformation on X
Linear	$y = \beta_0 + \beta_1 x$	None	None
Double Squared Root	$y = (\beta_0 + \beta_1 \sqrt{x})^2$	Square root	Square root
Multiplicative	$y = \beta_0 x^{\beta_1}$	Log	Log
Double Square	$y = \sqrt{\beta_0 + \beta_1 x^2}$	Square	Square
Log-Y square root-X	$y = e^{(\beta_0 + \beta_1 \sqrt{x})}$	Log	Square Root
Squared-Y square root X	$y = \sqrt{\beta_0 + \beta_1 \sqrt{x}}$	Square	Square Root
Square root-Y	$y = (\beta_0 + \beta_1 x)^2$	Square root	None
Square root-Y log-X	$y = (\beta_0 + \beta_1 ln(x)^2)$	Squared root	Log

As stated in [17], the status of a country's ICT services will determine how successful a VGI initiative could be and what growth may be expected in the years to come. Previous investigations [18] found that although OSM has had great global success, there is still a clear difference in the volume of contributed data between affluent and poorer communities. Since OSM relies upon volunteers and the amount of time and effort spent to the relevant area of the map, broader OSM coverage will happen in wealthier countries that have a high ICT level, given that this pillar measures the existence of modern infrastructure (mobile network coverage and quality of electricity supply), but also the capacity of businesses and individuals to use and provide online services. Therefore, in order to answer our Research Question 3, our analysis is carried out from two different points of view: (1) considering all

the countries as a whole, and (2) splitting the countries into three groups according to their ICT level given by the ICT readiness pillar of WEF.

Therefore, we used the value of the ICT readiness pillar (score from 1 to 7) to break up the analysis of countries into meaningful segments. Particularly, the values of this pillar that appear in the *Travel & Tourism Competitiveness Report 2017* range from 1.57 (Burundi) to 6.47 (Hong Kong SAR), so we created three ICT segments that stand for low, medium, and high ICT levels. Specifically, low ICT comprises countries that have values in $[1.5, 3.5]$, medium ICT includes countries with values in $[3.5, 5.0]$, and in the high ICT segment we found countries with values within $[5.0, 6.5]$. According to these intervals, 32 countries are classified as low ICT, 54 countries are classified as medium ICT, and 47 countries are classified as high ICT. In the Figure 2, we can observe how the countries are distributed according to the ICT level.

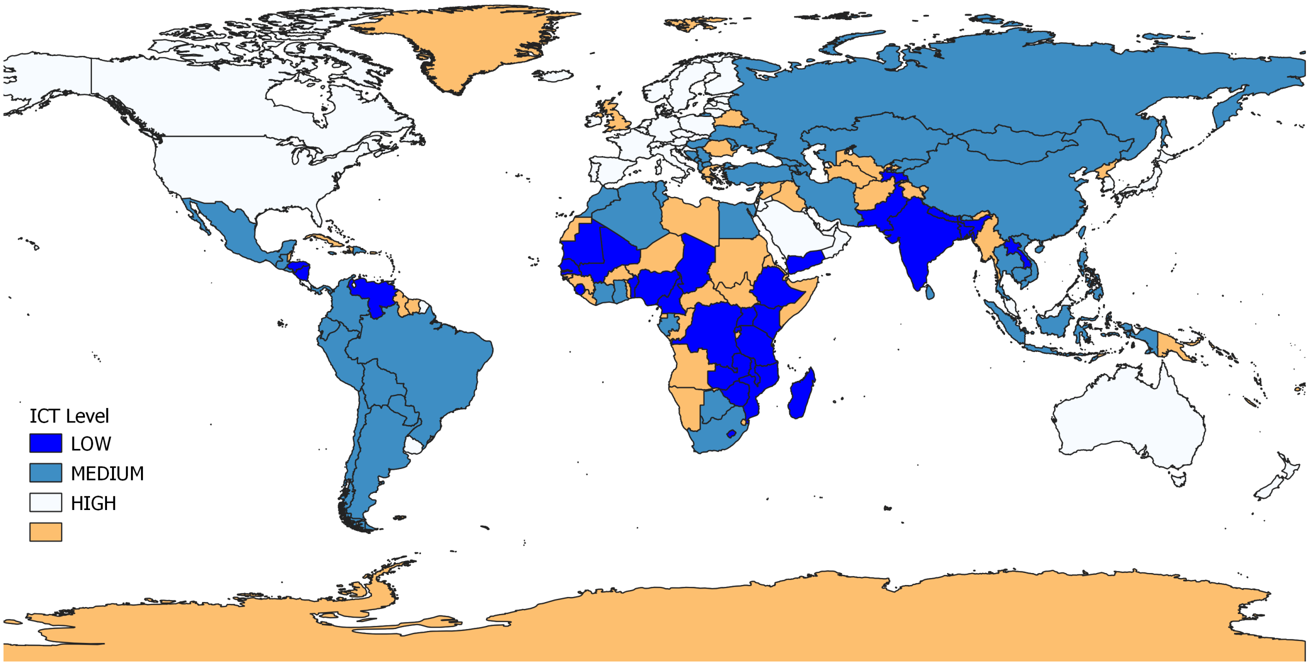

Figure 2. Map of countries by ICT level.

In summary, we performed the analysis of each variable by taking into account all the countries together, and also with respect to low, medium, and high ICT levels. First, data included in the OSM database at the beginning of 2018 is collected and processed as explained in Section 3.2. Then, the Statgraphics (www.statgraphics.com (accessed on 23 July 2020)) package is used to generate the regression models of each WEF variable from its OSM counterpart variable. In this case, the WEF values are extracted from the *2017 Travel & Tourism Competitiveness Report*. The models obtained using both approaches are compared and the models with the best determination coefficient are selected. In this selection, it is important to bear in mind that regression models are sensitive to outliers, that is, outliers may have a high effect on the regression model, an effect that increases as the amount of data decreases (as long as the data are not outliers). In other words, the models obtained for each ICT level will be more sensitive to outliers but, at the same time, they will allow to identify outliers.

Finally, we are interested in checking the applicability of the obtained models with new data. The main idea is to compare the last published WEF indicators (from *2019 Travel & Tourism Competitiveness Report*) with the predicted values given by our models, using as input data those that are included in the OSM database at the beginning of 2020. This way, data from the same period will be compared. In order to collect this new OSM data, we apply the same procedure explained in Section 3.2.

5. Results

From this point, we analyze how well the OSM data represent the eight WEF variables that measure the tourism competitiveness. Table 4 shows a summary of the results obtained in our analysis for each variable. Column *Best ICT segm.* indicates whether the best model has been found when considering the countries all together or when using the segmentation by ICT level. Columns *Best fit model* and *Overall adequacy to OSM* indicate the type of model that better fits the data and how well the data fits this model in each case. Each of the following sections is devoted to one variable; the details of the models for each ICT level, together with the correlation and R^2 values, are shown in Appendix A. The best model is selected for each variable, and then each of these models is applied to new OSM data (2019 data) in order to assess whether the model still gives a good fit. Column *Fit to 2019 data* in Table 4 compares the fitting to the model of data from 2017 with data from 2019 (Appendix B shows the R^2 value for each variable with both data sets).

Table 4. Summary of results for all the variables.

VAR.	Best ICT Segm.	Best Fit Model	Overall Adequacy to OSM	Fit to 2019 Data
CAR	All	Squared-Y Squared Root-X	Good	Slightly worse
ATM	All	Log-Y Squared Root-X	Fair	Slightly worse
HOT	ICT Segmentation	High: Squared Root-Y<br>Med: Multiplicative<br>Low: Multiplicative	Good<br>Good<br>Poor	Slightly worse<br>Similar<br>Slightly better
HBD	ICT Segmentation	High: Double Squared Root<br>Med: Multiplicative<br>Low: Double Square	Good<br>Good<br>Good	Slightly better<br>Slightly better<br>Better
WHS	ICT Segmentation	High: Log-Y Squared Root-X<br>Med: Log-Y Squared Root-X<br>Low: Double Square	Fair<br>Fair<br>Very good	Better<br>Better<br>Similar
AIR	All	Double Square	Very good	Slightly worse
CDD	ICT Segmentation	High: Log-Y Squared Root-X<br>Med: Squared Root-Y Log-X<br>Low: Double Square	Fair<br>Poor<br>Very good	Better<br>Slightly better<br>Similar
NAT	-	No model found	-	-

5.1. CAR

Firstly, we recall that this variable measures the presence of seven major car rental companies, so the variable CAR takes a value within $[0, 7]$. Appendix A summarizes the relationship between CAR-OSM and CAR-WEF, in addition to the model that best fits the data when the countries are all together and when they are grouped by ICT. It can be observed that the highest correlation (0.83) and the highest R^2 (0.704) are obtained when all the countries are considered. Specifically, the regression model that best fits the data is the following:

$$\text{CAR-WEF}(All) = \sqrt{7.61 + 15.83 * \sqrt{\text{CAR-OSM}}}. \quad (1)$$

The *p-value* lower than 0.05 indicates that there is a statistically significant relationship between CAR-WEF and CAR-OSM with a confidence level of 95%.

That said, the values obtained when the countries are classified by ICT are also acceptable, reflecting in all cases a strong and significant association. In general, the OSM coverage of this indicator across countries is relatively good as compared with the car rental companies registered in WEF.

Additionally, Figure 3a shows the mean values of CAR-OSM and CAR-WEF. The mean value of CAR-OSM for low ICT level countries is almost zero in contrast to the mean value

of CAR-WEF, which is about 3. This explains that the presence of car rental companies is not so extensive in this group of countries, and that the few existing companies are not well-mapped in the majority of countries. As an exception, the three most highly mapped countries are Nicaragua (6/7), Honduras (4/6), and Venezuela (3/4).

Countries that belong to the medium ICT level show a good correlation, partly supported by the positive correlation of some well-mapped countries like Morocco (5/6), Peru and Thailand (5/7), or Dominican Republic and Mexico (7/7), all important tourist destinations. In contrast, the relationship of countries that belong to the high ICT group is slightly worse because no car rental companies are mapped for quite a few countries that present high values of CAR-WEF like Lithuania, Slovenia, Jordan, Kuwait (CAR-WEF = 7) or Slovak Republic (CAR-WEF = 6). However, in this group, we can find the highest number of perfectly mapped countries with the best mapping possible 7/7 (e.g., France, Germany, Netherlands, United Arab Emirates, UK).

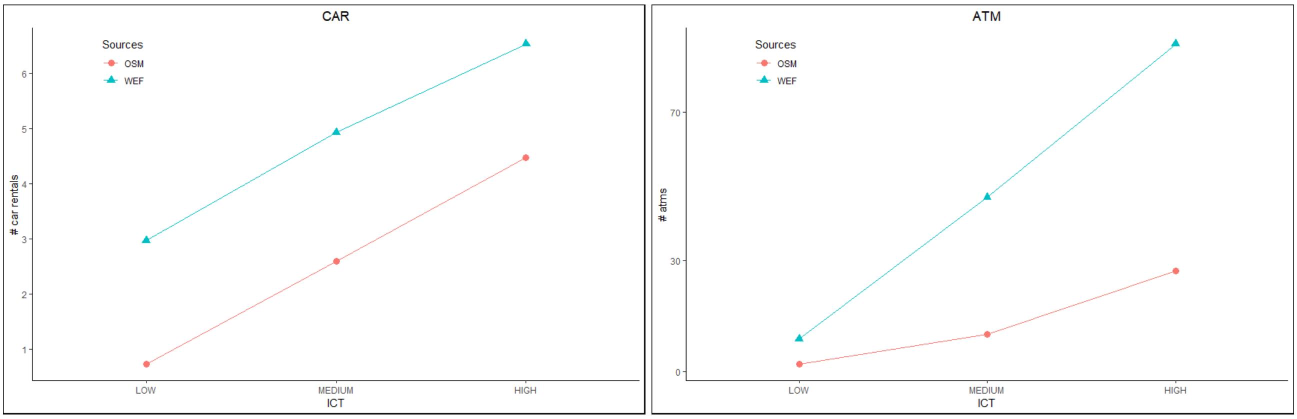

(**a**) CAR mean (**b**) ATM mean

Figure 3. CAR and ATM variables mean for different ICT levels.

Regarding the analysis with 2019 data, we can observe in Appendix B that the R^2 value is slightly worse than the R^2 obtained with data from 2017. This indicates that the model is not as well-adjusted to 2019 data as to 2017 data. However, the difference is not particularly remarkable.

As a conclusion, we can say that OSM reflects the official values of car rental companies across world economies quite well. More importantly, we can conclude that CAR-OSM is generally well-mapped in important tourist destinations, which leads us to confirm the representativeness of CAR-OSM for tourism purposes.

5.2. ATM

In this case, ATM-OSM is a value calculated upon an estimate of the number of machines per OSM node and the country population in order to approximate the value of ATM-WEF as much as possible.

The figures for the variable ATM are shown in Appendix A. Just like in the case of CAR, the model that best fits the data is the model obtained when taking into account all the countries, which explains a proportion of 0.42 of the variability of the ATM-WEF. The obtained model is the following:

$$\text{ATM-WEF}(All) = e^{(2.18+0.39*\sqrt{\text{OSM-ATM}})}. \quad (2)$$

Regarding the ICT segmentation models, a remarkable point is that the goodness of fit is inversely proportional to the ICT readiness, and the relationship for countries that belong to the high ICT level is neither strong nor significant, which is a clear indication that ATMs are not well-mapped in OSM. In developed countries that count on a huge number of ATMs, it seems reasonable that OSM contributors are not very interested in mapping

such facilities, as an ATM is easily found all around. The null correlation comes from the fact that although the ATM-OSM values of some countries are relatively large, they are still far from the values ATM-WEF (e.g., UK, Sweden, Singapore, Australia, Canada, Japan, Korea, USA, United Arab Emirates); and, on the contrary, others are found amongst the top-mapped countries (e.g., Croatia, Austria, Switzerland, Slovak Republic, Germany, Portugal, France). The mapping of ATM-OSM thus appears to be a result of randomness, as evidenced in the non-significant p-value. On the other hand, we can observe a relatively strong relationship between ATM-OSM and ATM-WEF in the group of low ICT countries. Clearly, the number of ATMs in these countries is far less than the number of ATMs in countries with high ICT level (see Figure 3b). Additionally, these ATMs are not evenly scattered all around the country and users have to travel a large distance to use ATM facilities [35]. Therefore, the scarce existing ATMs are highly mapped in OSM because it is important to locate them accurately.

It is important to note that the number of ATMs is an estimation, as explained in Section 3.2, and results reflect that this estimation should be improved. The countries with the largest actual number of ATMs, those at the high ICT level, also have the largest number of ATMs in OSM (as shown in Figure 3b), but the difference between the expected (WEF) and calculated (OSM) value is significant, which makes it difficult to find a good model. In contrast, ATM-WEF and ATM-OSM are much more similar in the low ICT level, but even in this case, it is not easy to find a better model. In fact, the best model is obtained when all the countries are considered, which implies that the effect of outliers is somewhat mitigated. When this model is applied to 2019 data, the R^2 value is slightly worse, similarly to the case of CAR-OSM, but again this difference is not very remarkable.

All in all, we can conclude that ATM-OSM data do not follow a clear pattern to adjust to ATM-WEF data.

5.3. HOT

In order to compare the values for this variable, we transformed the value provided by WEF (see Section 3.2) into the total number of hotel rooms available in a country using the World Bank population estimates. Hence, we will analyze the relationship between the number of hotels (HOT-OSM) with the total number of hotel rooms (HOT-WEF).

Unlike previous variables, in this case, the best-fitted models are those obtained for countries classified according to the different ICT levels, as shown in Appendix A. Both medium and low levels follow a quite similar model, unlike a high level. Specifically:

$$\text{HOT-WEF}(High) = (202.60 + 0.06 * \text{HOT-OSM})^2 \quad (3)$$

$$\text{HOT-WEF}(Medium) = e^{(3.67+1.06*ln(\text{HOT-OSM}))} \quad (4)$$

$$\text{HOT-WEF}(Low) = e^{(4.75+0.86*ln(\text{HOT-OSM}))} \quad (5)$$

On the other hand, it can be observed that both the linear correlation and R^2 are significant and quite similar for high and medium ICT levels, since the developed, richer countries with a higher level of ICT also have better hotel infrastructure and a more organized and competitive tourism industry as is the case of countries like Mexico, Greece at the medium level and Spain and France at the high level. However, it has not been possible to find a good model for countries in the low ICT level. This may reflect uneven data and the presence of outliers. In fact, when looking deep into the data, four outliers are identified (Burundi, Nigeria, Tajikistan, and Uganda). A new model is generated with the low ICT level countries by eliminating these outliers; this model obtains a R^2 of 0.4723 and an acceptable fit for outliers, quite similar in some cases, compared to the previous model for low ICT level countries.

On the other hand, the model with all the countries also obtains acceptable fitness to the data, comparable to those obtained for the CAR variable.

When the models by ICT levels are applied to the 2019 data (see Appendix B), the R^2 value is slightly worse in the case of high ICT countries and it remains the same for medium ICT countries, whereas it is better in the case of low ICT countries.

Finally, as a conclusion, we can say that the number of hotels mapped in OSM is a significant data source for countries that belong to medium and high ICT levels, even taking into account that both variables are measuring different concepts.

5.4. HBD

As with the variable HOT, in this variable we will analyse the relationship between the number of hospitals mapped in OSM with the total number of hospital beds (HBD-WEF), so we converted the original value of HBD-WEF, which is given as the number of hospital beds per population of 10,000, into the total number of hospital beds available in a country.

In this case, it is clear that the best models are those obtained for countries classified according the ICT level. Specifically:

$$\text{HBD-WEF}(High) = (8.49 + 2.93 * \sqrt{(\text{HBD-OSM})})^2 \quad (6)$$

$$\text{HBD-WEF}(Medium) = e^{(2.09+1.004*ln(\text{HBD-OSM}))} \quad (7)$$

$$\text{HBD-WEF}(Low) = \sqrt{(5.47E6 + 239.29 * \text{HBD-OSM}^2)}. \quad (8)$$

Appendix A shows that the strength and significance of the relationship between HBD-OSM and HBD-WEF is always increasing with a higher ICT level. The fact that in the high ICT level, the model explains a proportion of 0.829 when HBD-OSM and HBD-WEF refer to different concepts is especially remarkable.

This model behaves better when 2019 data are used. As shown in Appendix B, the value of R^2 is higher in all cases, even reaching 0.97 in the case of low ICT level countries.

All in all, we can say that institutions for health care are generally well-mapped in OSM, which are valuable data for tourism purposes.

5.5. WHS

As we can observe in Appendix A, in this case, the model obtained for all the countries is not the best option. The best figures are obtained for countries that belong to the low ICT level, and models for countries in the medium and high ICT levels are comparable with the model with all the countries. The models for the different ICT levels are:

$$\text{WHS-WEF}(High) = e^{(0.86+0.49*\sqrt{\text{WHS-OSM}})} \quad (9)$$

$$\text{WHS-WEF}(Medium) = e^{(0.62+0.58*\sqrt{\text{WHS-OSM}})} \quad (10)$$

$$\text{WHS-WEF}(Low) = \sqrt{(-18.69 + 5.09 * \text{WHS-OSM}^2)}. \quad (11)$$

Unlike other variables, in the case of WHS, a total of 15 countries present higher values in OSM than in WEF. Thus, Figure 4a shows a very similar gap for medium ICT and high ICT countries, and larger than the difference in the mean values of low ICT countries.

The good measures in the low ICT level are due to the fact that a group of 25 countries of this level present WHS-WEF values that range from 1 to 6 sites, and very few countries have null values of WHS-OSM. Additionally, countries with the highest WHS-WEF are also the best-mapped, like India (15/35), Ethiopia (5/9), or Senegal (4/7). It is also worth noting that the number of mapped sites of three African countries is higher than its official value in WHS-WEF, an indication that OSM contributors catalog some outstanding sites of their countries as World Heritage, even though they are not officially recognized as such. All in all, we can draw a good OSM representativeness of WHS in countries with a low ICT level.

For countries that belong to a medium or high ICT level, there is no such strong positive relation. The main reason lies in the existence of some countries that have large

values of WHS-WEF but are poorly mapped in OSM as, for instance, China (9/52) in medium ICT or Italy (2/51) in high ICT; while others are exceptionally well-mapped, such as Russia (20/26) and Spain (41/45) in medium and high ICT, respectively. As a result, the strength of the correlation decreases notably, as well as the goodness of the model. We believe that correcting the mapping of outliers in medium ICT (e.g., China, Mexico, Greece) and high ICT (e.g., Italy, Germany, USA) would enable to obtain a much more precise picture of the World Heritage Sites.

Appendix B shows that the adjustment of models for medium and high ICT levels improves with 2019 data, around 20% in both cases. This indicates that the models are still valid and that OSM data contain less outliers than 2017 data. The model for the low ICT level shows a very good fit with both datasets.

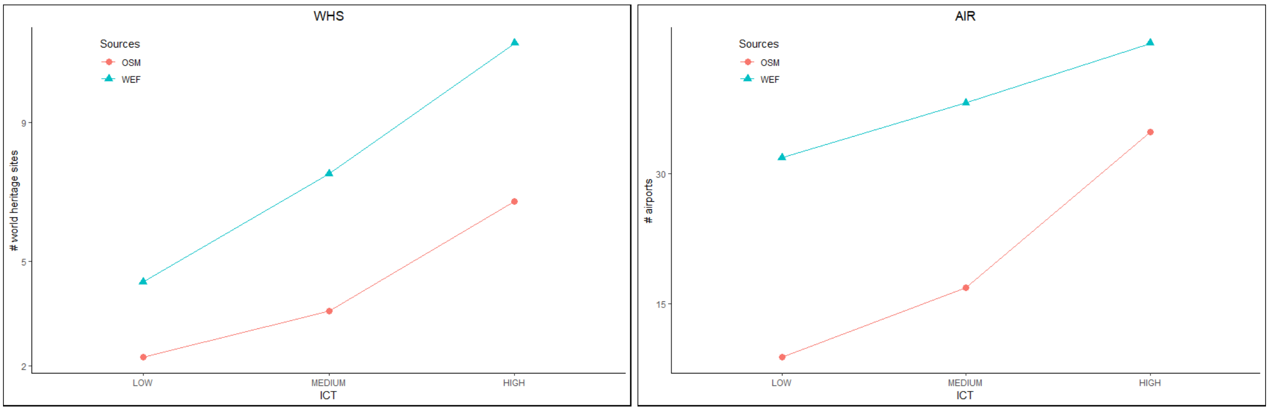

(**a**) WHS mean (**b**) AIR mean

Figure 4. WHS and AIR variables' means for different ICT levels.

5.6. AIR

For this variable, we converted the value of AIR-WEF, which measures airports per capita (million inhabitants), into the total number of airports using the World Bank population estimates. The result of comparing this value with the number of mapped airports (AIR-OSM) is shown in Appendix A. As we can see, there exists an almost perfect relationship for countries that belong to a high ICT level with only a few discrepancies due to OSM, which also records cargo or military airports. This results in an accurate model for countries in the high ICT level. In contrast, in low ICT, a very weak correlation is observed due to some outliers in the African continent, which means that the model hardly explains a proportion of 0.14 of the variability of AIR-WEF. When generating a new model by eliminating outliers (in this case, Burundi, Benin, Ethiopia, and Madagascar), no substantial improvement is obtained (R^2 = 0.1812). We can say, however, that there exists a strong association for important tourist destinations like India, Kenya, or Madagascar. The same trend is revealed by Figure 4b, where it can be observed that the gap in the difference of the mean values narrows down as the ICT level increases.

Therefore, the model with all the countries, that reaches a R^2 of 0.93, is considered the best model for this variable. The obtained regression model is:

$$\text{AIR-WEF}(All) = sqrt(2374.63 + 1.54 * \text{AIR-OSM}^2). \quad (12)$$

Appendix B shows that the R^2 for this model is slightly worse when applied to 2019 data, but it still has a good fit (0.916).

All in all, we can conclude that the higher the ICT level, the more representative the relationship between AIR-OSM and AIR-WEF, and the discrepancies in the low ICT level are mitigated by the good adjustment in the other levels. Despite the fact that the two sources are not measuring exactly the same airport concept (WEF counts only airports with one scheduled flight per million of urban population, whereas OSM is counting all airports

as long as they are tagged as public), the model with all the countries is able to explain a significant proportion of the AIR-WEF variability.

5.7. CDD

As explained above, in this case, the analysis is focused on the relationship between the online search index of cultural and entertainment activities (CDD-WEF) and the mapped locations in OSM that offer such activities. Appendix A shows that this relationship is strong in low ICT level countries, but it is weak and moderate in medium and high ICT level countries, respectively. The models obtained for this variable exhibit similar behaviour to the WHS variable. Therefore, the models for each ICT level are considered more accurate:

$$\text{CDD-WEF}(High) = \sqrt{-100.21 + 17.06\sqrt{\text{CDD-OSM}}} \tag{13}$$

$$\text{CDD-WEF}(Medium) = (-0.60 + 0.60 * ln(\text{CDD-OSM}))^2 \tag{14}$$

$$\text{CDD-WEF}(Low) = \sqrt{15.66 + 0.0002 * \text{CDD-OSM}^2}. \tag{15}$$

A close look at the collected data reveals that the highest coverage of mapped locations corresponds by far to European countries, which also have the highest search index globally. This is the main reason that justifies the stronger correlation of the high-ICT countries, since most European countries fall within this group. The second-ranked group of countries in relation to OSM coverage corresponds to both North and South American countries, and finally the Southeast Asian countries.

The disparity between the search index and mapped locations that makes the correlation weak and moderate in medium and high ICT countries, respectively, is mostly affected by the highly coverage of European countries in comparison to the rest of the countries. As an example, the search index of countries like Czech Republic (6.5) and Poland (14) is 5 and 2.5 times less than the search index of the USA (34), while the number of mapped locations is two and three times higher in these two countries than in USA. If we focus exclusively on medium ICT, Peru and Chile have almost the same search index as Greece, but 60% less mapped locations. This provides evidence that, globally, Europe is extensively much better-mapped than the rest of the world, especially concerning cultural interests.

As for low-ICT countries, the relationship is highly significant. Furthermore, the coefficient of determination in this case is $R^2 = 0.99$, thus indicating that 99% of variation of CDD-WEF is attributed to the predictor variable CDD-OSM. This value is still excellent when the model is applied to 2019 data. Moreover, the model adjustment for medium and high ICT levels improves with the new dataset.

5.8. NAT

In this case, NAT-WEF is a survey indicator that measures to what extent a country is visited by its natural assets, while NAT-OSM counts the number of natural assets. As we can see in Appendix A, no correlation is found between the two values, or a very weak relationship is found for the high ICT group. Additionally, the model's adjustment shows a similar trend. In the group with a high ICT level, we find that except Australia, Norway, and Spain, other countries that are well-renowned for their natural spots and also have a large value of NAT-WEF are very poorly mapped—namely, Iceland, Costa Rica, and Ireland.

Therefore, we conclude that OSM is not a very informative source when looking for the natural spots of a country.

6. Discussion

This section discusses the results presented in the previous section, describes the limitations encountered in this analysis, and provides suggestions to make OSM a user-generated VGI reference platform in tourism management.

From Table 4 and Appendices A and B, we can conclude that OSM is representative of WEF data for CAR, HBD, and AIR variables; in the case of HOT, WHS, and CDD, it depends

on the ICT level, and for ATM and especially NAT, the adequacy is not good. Moreover, we can observe that there is not a clear pattern regarding the OSM representativeness in comparison to WEF when the ICT level is taken into account. That is, in some cases, countries with a high ICT level show the best values (for example, for the AIR and HOT variables), whereas in other cases, such as WHS and CDD, countries with a low ICT level show better values. In the following, we will explain the difficulties we have faced that may explain these results.

The first limitation of OSM is the incompleteness of the data regarding the mapped elements—that is, many spots are not mapped (for example, ATMs), especially in countries with a low ICT level. In fact, in the several maps provided by Anderson [36], we can observe the huge differences in the editing density across countries, with Europe being the area with the highest density in contrast with low-ICT countries. This map also shows that the editing task also focuses on some specific areas of some countries. In general, well-governed countries with good Internet access tend to be more complete, and both sparsely populated areas and dense cities are the best-mapped [37]. However, in the last few years, there has been a significative effort in mapping many areas of Africa, as shown by Kateregga [38], which will have a positive impact on the representation of OSM with respect to WEF in these countries.

Another limitation is the incompleteness of the data with respect to the value of tags; that is, many spots are mapped but some lack information in key tags, and so we were not able to extract the same exact information as represented by WEF. This happens in variables such as HBD and HOT; there are tags defined in OSM to specify the value of the number of hospital beds or the hotel rooms but, in many cases, this information is not registered. As explained in Section 4, we have (quite successfully) overcome this difficulty in these cases by using an approximation. On the other hand, as explained above, in countries with a high ICT level, the information regarding World Heritage Sites is not registered in the appropriate tag, which has made it difficult to identify these spots. Given that these factors are important for the image of a country, authorized initiatives to record these types of data in OSM could be encouraged.

Additionally, we have missed some tags in the OSM catalog that would be very helpful in our analysis. For instance, in the case of NAT and CDD variables, a tag like `attraction:type = {Natural, Cultural}` would have been useful because it would have allowed us to retrieve data with greater precision and ease and it would increase the precision in our calculations.

On the other hand, apart from the incompleteness of OSM data, our interpretation of the WEF variables in terms of OSM tags may indeed affect the accuracy of the results. For example, the estimation we used in our analysis for the variable HOT works well for high and medium ICT countries, but it should be adjusted for low-ICT countries. This fact is especially remarkable in the variable AIR, where the R^2 is 0.96 for high-ICT countries and only 0.13 for low-ICT countries. In the latter case, it would be interesting to add some additional information for a better estimation. Sometimes, however, it is not easy to find; for example, [39] publishes the airport traffic data for the top 60 worldwide airports, with respect to passengers' traffic, but we have not found data about small airports. Another variable that would benefit from the combination of OSM data with external resources is WHS for high and medium ICT level countries: the Wikipedia gives an exhaustive list of World Heritage Sites by country [40]; however, in this case, a better approach would be to use the information in Wikipedia to complete the corresponding tag in OSM data.

We envision the following challenges to make OSM a user-generated VGI reference platform in tourism management: (1) To expand the OSM tagging system by including specific tourism-related tags; (2) encourage users, representatives, authorities, and tourism industry managers to participate in OSM; (3) foster a balance between the general freedom of OSM contributors to fill in data and producing data in a standardized way. Additionally, interesting initiatives like LinkedGeoData that collect spatial data from OSM and make it

available as an RDF knowledge base will help increase the visibility of OSM and incentivize its utilization by visitors.

7. Conclusions

Tourism research has fostered the exploitation of OSM in *smart tourism* projects, encouraged by promising outcomes of studies that regard OSM as a holistic tourism platform. This new vision of tourism that deals with hyper-connected tourists who consume content any time and through different channels revolves around two core elements, smart phones and geolocation, with OSM being mostly a globally used geodata platform.

In this paper, we have presented an exploratory analysis to study the representativeness of data gathered in OSM. We have undertaken a thorough analysis of eight variables of WEF that cover different tourism aspects, and examined how well OSM data reflect the official values of such variables. We carefully selected the most representative OSM tags to retrieve the information comprised in the eight variables, and then studied for each variable the relationship between the official value and the OSM value.

The presented analysis is a small sample that illustrates the adequacy of OSM user-generated content for obtaining a picture of the tourism industry in a country. We selected a few variables representing concepts that are measurable and comparable with official statistics, but the analysis is extensible to the large variety of maps, data, and volunteered geo-information offered by OSM.

Studies such as the one presented in this article are relevant because they serve to determine whether OSM data can be used as a reliable data source for tourism-related applications.

Further work can be done to study other indicators that highly influence tourism behaviour, such as road density, railroad infrastructure, or protected areas, as well as extending the analysis to other collaborative data sources, such as DBPedia and Foursquare, among others. In addition to the ICT level, some other aspects could also be considered, such as the country's population, geographical area, gross domestic product, or the International Monetary Fund classification in *Advanced countries* and *Emerging and developing countries*, among others, in the model generation.

Author Contributions: Alexander Bustamante: Conceptualization, Visualization, Software, Writing—original draft; Laura Sebastia: Methodology, Writing—review & editing ; Eva Onaindia: Supervision, Writing—review & editing. All authors have read and agreed to the published version of the manuscript.

Funding: This work has been supported by COLCIENCIAS through a PhD scholarship.

Institutional Review Board Statement: Not applicable.

Informed Consent Statement: Not applicable.

Acknowledgments: This work is supported by the Spanish MINECO project TIN2017-88476-C2-1-R. Map data copyrighted OpenStreetMap contributors and available from https://www.openstreetmap.org (accessed on 29 May 2020)).

Conflicts of Interest: The authors declare no conflict of interest.

Abbreviations

The following abbreviations are used in this manuscript:

MDPI	Multidisciplinary Digital Publishing Institute
DOAJ	Directory of open access journals
TLA	Three letter acronym
LD	linear dichroism

Appendix A. Models Obtained for All the Variables

This table shows the correlation value and the determination coefficient for each variable and ICT-based segment (*All* refers to all countries analysis and *High*, *Medium* and *Low* refer to the analysis based on country segmentation by ICT level). Column *Model* indicates the type of model that better fits the data at hand and column *p-value* shows the confidence level of this model.

Table A1. Models by ICT level.

VAR.	ICT	Model	Correlation	R^2	*p*-Value
CAR	All	Squared-Y Squared Root-X	0.83	0.7041	<0.05
	High	Double Squared Root	0.71	0.5174	<0.05
	Medium	Squared-Y Squared Root-X	0.78	0.619	<0.05
	Low	Double Square	0.80	0.6433	<0.05
ATM	All	Log-Y Squared Root-X	0.64	0.4209	<0.05
	High	Reciprocal-Y Squared-X	−0.27	0.0752	>0.05
	Medium	Log-Y Squared Root-X	0.46	0.2186	<0.05
	Low	Multiplicative	0.56	0.3151	<0.05
HOT	All	Squared Root-Y	0.83	0.6986	<0.05
	High	Squared Root-Y	0.88	0.7802	<0.05
	Medium	Multiplicative	0.88	0.7920	<0.05
	Low	Multiplicative	0.61	0.3799	<0.05
HBD	All	Log-Y Squared Root-X	0.77	0.6021	<0.05
	High	Double Squared Root	0.91	0.8290	<0.05
	Medium	Multiplicative	0.85	0.7325	<0.05
	Low	Double Square	0.83	0.7018	<0.05
WHS	All	Double Squared Root	0.67	0.4508	<0.05
	High	Log-Y Squared Root-X	0.69	0.4791	<0.05
	Medium	Log-Y Squared Root-X	0.63	0.4089	<0.05
	Low	Double Square	0.95	0.9121	<0.05
AIR	All	Double Square	0.96	0.9311	<0.05
	High	Double Square	0.98	0.9680	<0.05
	Medium	Double Squared Root-X	0.70	0.4933	<0.05
	Low	Reciprocal-Y Squared-X	0.37	0.1391	<0.05
CDD	All	Squared-Y Squared Root-X	0.63	0.4038	<0.05
	High	Log-Y Squared Root-X	0.66	0.4409	<0.05
	Medium	Squared Root-Y Log-X	0.52	0.2765	<0.05
	Low	Double Square	0.99	0.9947	<0.05
NAT	All	Log-Y Squared Root-X	0.17	0.0304	<0.05
	High	Log-Y Squared Root-X	0.23	0.0570	<0.05
	Medium	Double Square	−0.12	0.0150	<0.05
	Low	Double Square	−0.08	0.0072	<0.05

Appendix B. Comparison between 2017 and 2019 Data

This table shows a comparison between OSM from 2017 (original data) and 2019 (test data), where the determination coefficient for both sets of data and for the selected models can be observed.

Comparison between results with the original data from 2017 and test data from 2019.

Table A2. Model fit comparison

VAR.	ICT	R^2—2017 (Original Data)	R^2—2019 (Test Data)
CAR	All	0.7041	0.6697
ATM	All	0.4209	0.4149
HOT	High	0.7802	0.7587
	Medium	0.7920	0.795
	Low	0.3799	0.4343
HBD	High	0.829	0.86586
	Medium	0.7325	0.7949
	Low	0.7018	0.9733
WHS	High	0.4791	0.615
	Medium	0.4089	0.6575
	Low	0.91527	0.91966
AIR	All	0.9311	0.91628
CDD	High	0.4409	0.59992
	Medium	0.2765	0.3077
	Low	0.9947	0.9986

References

1. Perles-Ribes, J.F.; Ramón-Rodríguez, A.B.; Rubia, A.; Moreno-Izquierdo, L. Is the tourism-led growth hypothesis valid after the global economic and financial crisis? The case of Spain 1957–2014. *Tour. Manag.* **2017**, *61*, 96–109, doi:10.1016/j.tourman.2017.01.003.
2. World Economic Forum. 2017 Available online: https://www.weforum.org/about/world-economic-forum (accessed on 12 May 2020).
3. Li, J.; Xu, L.; Tang, L.; Wang, S.; Li, L. Big data in tourism research: A literature review. *Tour. Manag.* **2018**, *68*, 301–323.
4. e Silva, F.B.; Herrera, M.A.M.; Rosina, K.; Barranco, R.R.; Freire, S.; Schiavina, M. Analysing spatiotemporal patterns of tourism in Europe at high-resolution with conventional and big data sources. *Tour. Manag.* **2018**, *68*, 101–115. doi:10.1016/j.tourman.2018.02.020.
5. Nakahira, K.T.; Akahane, M.; Fukami, Y. Intelligent Interactive Multimedia: Systems and Services. *Smart Innov. Syst. Technol.* **2012**, *14*, 609–617.
6. Nin, J.; Villatoro, D. *Citizen in Sensor Networks: Second International Workshop, CitiSens 2013 Barcelona, Spain, September 19, 2013 Revised Selected Papers*; Lecture Notes in Computer Science (Including subseries Lecture Notes in Artificial Intelligence and Lecture Notes in Bioinformatics); Springer: Berlin/Heidelberg, Germany, 2014; Volume 8313, pp. 26–35.
7. Chua, A.; Servillo, L.; Marcheggiani, E.; Moere, A.V. Mapping Cilento: Using geotagged social media data to characterize tourist flows in southern Italy. *Tour. Manag.* **2016**, *57*, 295–310.
8. Bustamante, A.; Sebastia, L.; Onaindia, E. Can Tourist Attractions Boost Other Activities Around? A Data Analysis through Social Networks. *Sensors* **2019**, *19*, 2612.
9. Zeng, B.; Gerritsen, R. What do we know about social media in tourism? A review. *Tour. Manag. Perspect.* **2014**, *10*, 27–36.
10. Lalicic, L. Open innovation platforms in tourism: how do stakeholders engage and reach consensus? *Int. J. Contemp. Hosp. Manag.* **2018**, *30*, 2517–2536.
11. Chareyron, G.; Da-Rugna, J.; Raimbault, T. Big data: A new challenge for tourism. In Proceedings of the 2014 IEEE International Conference on Big Data, IEEE Big Data, Washington, DC, USA, 27–30 October 2014; pp. 5–7.
12. OpenStreetMap Contributors. Planet Dump Retrieved. 2017. Available online: https://www.openstreetmap.org (accessed on 20 May 2020).
13. Mooney, P.; Minghini, M. *A Review of OpenStreetMap Data*; Ubiquity Press: London, UK, 2017.
14. Wei, C.C.; Lin, J.S.; Chang, C.C.; Huang, Y.F.; Lin, C.B. The Development of E-Bike Navigation Technology Based on an OpenStreetMap. *Smart Sci.* **2018**, *6*, 29–35.
15. Mobasheri, A.; Huang, H.; Degrossi, L.C.; Zipf, A. Enrichment of OpenStreetMap Data Completeness with Sidewalk Geometries Using Data Mining Techniques. *Sensors* **2018**, *18*, 509.
16. Eckle, M.; Herfort, B.; Yan, Y.; Kuo, C.L.; Zipf, A. Towards using Volunteered Geographic Information to monitor post-disaster recovery in tourist destinations. In Proceedings of the 14th ISCRAM Conference, Albi, France, 21–24 May 2017; pp. 1008–1019.
17. Siebritz, L. Assessing the Accuracy of OpenStreetMap Data in South Africa for the Purpose of Integrating it with Authoritative Data. Master's Thesis, University of Cape Town, Ciudad del Cabo, South Africa, 2014.

18. Haklay, M. How good is volunteered geographical information? A comparative study of OpenStreetMap and Ordnance Survey datasets. *Environ. Plan. B Plan. Des.* **2010**, *37*, 682–703.
19. Goodchild, M.F. Citizens as sensors: The world of volunteered geography. *GeoJournal* **2007**, *69*, 211–221.
20. Costa Fonte, C.; Fritz, S.; Olteanu-Raimond, A.M.; Antoniou, V.; Foody, G.; Mooney, P.; See, L. *Mapping and the Citizen Sensor*; Ubiquity Press: London, UK, 2017.
21. Mooney, P.; Corcoran, P.; Ciepluch, B. The Potential for Using Volunteered Geographic Information in Pervasive Health Computing Applications. *J. Ambient. Intell. Humaniz. Comput.* **2013**, *4*, doi:10.1007/s12652-012-0149-4.
22. Hennig, S. OpenStreetMap used in protected area management. The example of the recreational infrastructure in Berchtesgaden National Park. *J. Prot. Mt. Areas Res.* **2017**, *1*, 30–41, doi:10.1553/eco.mont-9-2s30.
23. Coleman, D. The potential and early limitations of volunteered geographic information. *Geomatica* **2010**, *64*, 209–219.
24. Kloog, I.; Kaufman, L.I.; De Hoogh, K. Using Open Street Map Data in Environmental Exposure Assessment Studies: Eastern Massachusetts, Bern Region, and South Israel as a Case Study. *Int. J. Environ. Res. Public Health* **2018**, *15*, 2443.
25. Mondzech, J.; Sester, M. Quality analysis of OpenStreetMap data based on application needs. *Cartogr. Int. J. Geogr. Inf. Geovis.* **2011**, *46*, 115–125.
26. Arsanjani, J.J.; Mooney, P.; Zipf, A.; Schauss, A. Quality assessment of the contributed land use information from OpenStreetMap versus authoritative datasets. In *OpenStreetMap in GIScience*; Springer: Berlin/Heidelberg, Germany, 2015; pp. 37–58.
27. Balducci, F. Is OpenStreetMap a good source of information for cultural statistics? The case of Italian museums. *Environ. Plan. Urban Anal. City Sci.* **2019**, doi:10.1177/2399808319876949.
28. Feldmeyer, D.; Meisch, C.; Sauter, H.; Birkmann, J. Using OpenStreetMap Data and Machine Learning to Generate Socio-Economic Indicators. *ISPRS Int. J. Geoinf.* **2020**, *9*, 498.
29. Almendros-Jiménez, J.M.; Becerra-Terón, A. Analyzing the Tagging Quality of the Spanish OpenStreetMap. *ISPRS Int. J. Geoinf.* **2018**, *7*, 323, doi:10.3390/ijgi7080323.
30. Bustamante, A.; Laura, S.; Onaindia, E. Exploratory analysis of representativeness of tourism data in OpenStreetMap. In Proceedings of the 33 International Business Information Management (33 IBIMA 2019), Granada, Spain, 10–11 April 2019; pp. 4161–4169.
31. Levin, N.; Lechner, A.M.; Brown, G. An evaluation of crowdsourced information for assessing the visitation and perceived importance of protected areas. *Appl. Geogr.* **2017**, *79*, 115–126, doi:10.1016/j.apgeog.2016.12.009.
32. Roman, D.; Tarasova, T.; Paniagua Laconich, E.J. MethOSM: A methodology for computing composite indicators derived from OpenStreetMap data. *J. Spat. Inf. Sci.* **2019**, *2019*, 3–27, doi:10.5311/JOSIS.2019.19.491.
33. World Economic Forum. Travel and Tourism Competitiveness Report 2017. 2017. Available online: http://reports.weforum.org/travel-and-tourism-competitiveness-report-2017/ (accessed on 2 May 2020).
34. Ramm, F.; Topf, J.; Chilton, S. *OpenStreetMap:Using and Enhancing the Free Map of the World*; UIT Cambridge: London, UK, 2010.
35. Karunanayake, A.; De Zoysa, K.; Muftic, S. Mobile ATM for developing countries. In Proceedings of the 3rd International Workshop on Mobility in the Evolving Internet Architecture (MobiArch'08), Seattle, WA, USA, 22 August 2008, doi:10.1145/1403007.1403014.
36. Anderson, J. OpenStreetMap Contribution Analysis. A Research Collaboration with Mapbox. 2016. Available online: http://mapbox.github.io/osm-analysis-collab (accessed on 2 January 2021).
37. Barrington-Leigh, C.; Millard-Ball, A. The world's user-generated road map is more than 80% complete. *PLoS ONE* **2017**, *12*, e0180698.
38. Kateregga, G. The State of OpenStreetMap in Africa. 2020. Available online: https://medium.com/@kateregga1/the-state-of-openstreetmap-in-africa-223ecadd5556 (accessed on 20 January 2021).
39. *United States: Port Authority of New York and New Jersey*; 2019 Annual Airport Traffic Report; Airport Traffic: New York, NY, USA, 2020.
40. Wikipedia. World Heritage Sites by Country. 2020. Available online: https://en.wikipedia.org/wiki/World_Heritage_Sites_by_country (accessed on 22 November 2020).
