# Supplementary material for: Estimation of Undetected Asymptomatic COVID-19 Cases in South Korea Using a Probabilistic Model
Source: Int J Environ Res Public Health. 2021 May 6;18(9):4946. doi: 10.3390/ijerph18094946 (PMC8124955; doi:10.3390/ijerph18094946)
Supplement: Supplementary file 1 [file ijerph-18-04946-s001.zip › 5/ijgi-1151668.pdf]

International Journal of Geo-Information

MDPI

Article

Spatial Pattern of Highway Transport Dominance in Qinghai–Tibet Plateau at the County Scale

Zhiheng Wang [1,2], Hongkui Fan [2], Daikun Wang [3,*], Tao Xing [2], Dongchuan Wang [2], Qiaozhen Guo [2] and Lina Xiu [2]

1 Tianjin Key Laboratory of Soft Soil Characteristics and Engineering Environment, Tianjin Chengjian University, Tianjin 300384, China; wangzhiheng@tcu.edu.cn
2 School of Geology and Geomatics, Tianjin Chengjian University, Tianjin 300384, China; hongkuifan@126.com(H.K.F.); xingtao961126@126.com(T.X.); mrwangdc@126.com(D.C.W.); gqiaozhen@tcu.edu.cn(Q.Z.G.); xlntcu12@126.com(L.X.)
3 Department of Geography and Resource Management, The Chinese University of Hong Kong, Shatin, New Territories, Hong Kong 999077, China
* Correspondence: DerekWang@link.cuhk.edu.hk

Abstract: The highway is an important mode of transportation in the Qinghai–Tibet Plateau, and can be regarded as a major contributor to the high-quality and sustainable development of the Qinghai–Tibet Plateau. It is of great significance to explore its spatial distribution and characteristics for understanding the regional and geographical process. Although Qinghai–Tibet Plateau's highway transportation infrastructure has been experiencing rapid development in recent years, there lacks a systematic examination of the whole Qinghai–Tibet Plateau from the perspective of supportive capacity for its socio-economic activities. This paper applies geospatial analysis methods, such as network analysis, spatial statistics, and weighted overlay, to model the highway transport dominance in the Qinghai–Tibet Plateau in 2015 at the county scale and reveals the basic characteristics of the highway transport dominance's spatial pattern. The results are mainly of four aspects: 1) there is a significant difference between the east and west of the highway in the Qinghai–Tibet Plateau, showing an irregular circle structure of gradual attenuation from the east to west; 2) at the county scale, the highway transport dominance in the Qinghai–Tibet Plateau shows strong spatial autocorrelation and a certain extent of spatial heterogeneity, presenting a spatial distribution pattern of High–High and Low–Low clustering; 3) the urban locations of Lhasa, Xining and other center cities have obvious spatial constraints on the distribution of highway transport dominance and generally have a logarithmic decline trend; and 4) there are obvious differences in distribution among the three Urban Agglomerations in the Qinghai–Tibet Plateau. Due to the influence of traffic location, topography, construction of national trunk lines, and level of socio-economic development., the traffic conditions of Lan-Xi Urban Agglomeration and Lhasa Urban Agglomeration are better than Kashgar Urban Agglomeration. This study can be used to guide the optimization of the highway network structure and provide a macro decision-making reference for the planning and evaluation of major highway projects in the Qinghai–Tibet Plateau.

Keywords: highway transport dominance; spatial autocorrelation; Qinghai–Tibet Plateau; county scale

Citation: Wang, Z.; Fan, H.; Wang, D.; Xing, T.; Wang, D.; Guo, Q.; Xiu, L. Spatial Pattern of Highway Transport Dominance in Qinghai–Tibet Plateau at the County Scale. *ISPRS Int. J. Geo-Inf.* **2021**, *10*, 304. https://doi.org/10.3390/ijgi10050304

Academic Editors: Wolfgang Kainz, Alexandre B. Gonçalves and Filipe Moura

Received: 4 March 2021
Accepted: 1 May 2021
Published: 5 May 2021

Publisher's Note: MDPI stays neutral with regard to jurisdictional claims in published maps and institutional affiliations.

Copyright: © 2021 by the authors. Licensee MDPI, Basel, Switzerland. This article is an open access article distributed under the terms and conditions of the Creative Commons Attribution (CC BY) license (http://creativecommons.org/licenses/by/4.0/).

1. Introduction

Due to global warming and excessive disturbance of human activities, the ecological evolution and regional development trend of the Qinghai–Tibet Plateau have attracted extensive attention at home and abroad [1]. The highway is currently the most widely used and covered transportation mode in China. The construction of highways has great significance in promoting population mobility, business exchange, and technical development among cities [2,3]. It also has important influence on regional sustainable

development from the aspects of environment, society and economy [4,5]. The Qinghai–Tibet Plateau is the most unique unit of geography–ecology–population–transportation in the world. The development characteristics and laws of the regional transportation network, especially the highway network system, are an important entry point for the coordinated development of the human–land relationship [6], which can be regarded as an important influence to evaluate the level of sustainable development in the Qinghai–Tibet Plateau [7].

The transportation infrastructure level plays an important role in guiding, supporting, and guaranteeing the regional development [8]. How to quantitatively analyze and evaluate the level of regional transportation infrastructure has become a research hotspot for scholars in recent years. Scholars mainly use distance or time-cost indicators to evaluate traffic convenience from the perspective of accessibility. The research focus has gradually shifted from early theoretical research on the concept and definition of accessibility [9–11] to empirical research on methods [12,13] and technologies [14,15]. The types [16–18], levels [19] and scales [20] of the research objects have been improved, which has laid a solid foundation for subsequent research [21–24]. In China, Jin et al. put forward the concept of transport dominance, from the three perspectives of "quality", "quantity" and "advantage", using a multi-factor comprehensive integration method to construct an evaluation model. The technical level of transport is reflected by “quality”. The network scale of transport is represented by “quantity”. The relative priority of transport is regarded as “advantage” [25,26]. It is an important index to measure the accessibility, effectiveness and supporting capacity of a regional transportation network structure [20]. Subsequently, scholars conduct empirical analyses on the transport dominance in Shanxi Province, Shandong Province and Huang-Huai-Hai Plain in China [27–29]. At present, the study on regional transport dominance mainly focuses on evaluation methods of transport dominance evaluation, spatial–temporal pattern evolution, impact factors, spatial synergies analysis, etc.

The Qinghai–Tibet Plateau belongs to a special traffic region in China, called the Qinghai–Tibet Alpine Region [30]. Restricted by natural conditions, the economy and other impact factors, the transportation development in this region has been in a backward state for a long time, which has become one of the important factors restricting social and economic development. With the advancement of national policies (i.e., “Western Development Strategy” and “One Belt, One Road”) and the infrastructure construction, the transportation facilities in the Qinghai–Tibet Plateau have been greatly improved. With the construction and operation of various types of transportation facilities, more and more scholars have begun to pay attention to their spatial and temporal convergence effects, changes in the spatial structure of industrial layout, and social–economic development [31]. Sun et al. [32] studied the spatial distribution imbalance of transport dominance in Qinghai province at the county scale, and verified the positive interaction between transportation development and economic development. Li and Wu [33] identified that the accessibility pattern of Qinghai Province is high in the northeast and low in the southwest along the Liu–Ge Expressway. The accessibility of tourism resources forms a circle differentiation centered on the Huang He Valley and Qinghai Lake area and decays outward. In recent years, scholars have used methods, such as network analysis, cost distance, and hot spot analysis, to analyze the characteristics and spatial patterns of the transportation infrastructure in the Qinghai–Tibet Plateau [34]. However, the overall systematic evaluation of the highway is very rare. In particular, due to the leapfrog development of the highway transportation infrastructure in the Qinghai–Tibet Plateau in recent years, the accessibility of internal and external transportation in this region has been greatly improved. However, this situation does not seem to attract much attention, apparent especially in the lack of quantitative evaluation and analysis at the county scale. The existence of homogeneity makes the inner county’s micro-differentiation, such as the spatial distribution pattern and spatial equity, unable to be effectively reflected.

Compared with other transportation modes, highway transportation plays a more important role in promoting the construction of central cities, developing tourism resources, and promoting the development of the logistics industry in the Qinghai–Tibet Plateau. Based on the concept of geography and spatial–temporal analysis, this paper takes the highway traffic modes of the Qinghai–Tibet Plateau as the research object, constructs the evaluation index system of highway transport dominance, analyzes spatial differentiation characteristics of highway transport dominance, and explores the spatial inequality caused by the time–space convergence effect of the highway traffic system in order to provide a theoretical perspective and regional analysis case for the study of highway traffic patterns in the Qinghai–Tibet Plateau and its impact on human activities. The research framework of this paper is shown in Figure 1.

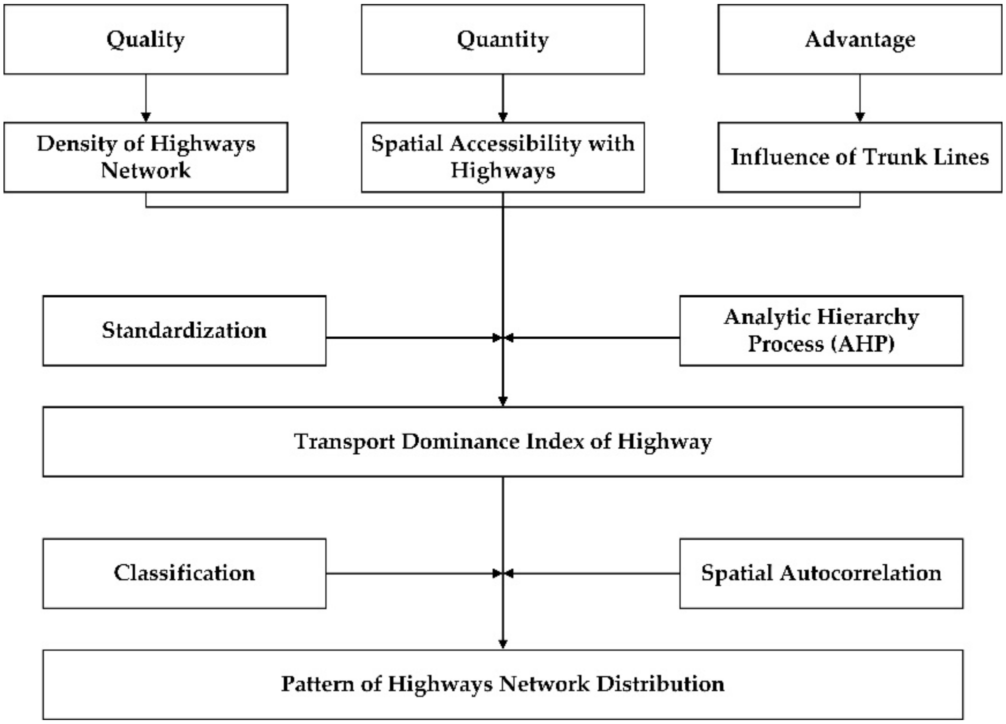

Figure 1. Research framework.

Combined with the actual situation and characteristics of the Qinghai–Tibet Plateau, this paper constructs an evaluation index system of highway transport dominance and uses the Analytic Hierarchy Process (AHP) method to determine the weight of indexes and measure the highway transport dominance of each county in the Qinghai–Tibet Plateau. By exploring the spatial pattern of highway transport dominance at the county scale, this paper analyzes the spatial differentiation characteristics of transport dominance in the Qinghai–Tibet Plateau.

The characteristics of vast territory, high altitude, cold climate and relatively little interference by human activities make the Qinghai–Tibet Plateau suitable for the environment background for global change research [35]. It is of great practical and strategic significance to study the spatial distribution pattern of highways in the Qinghai–Tibet Plateau to put forward effective ways to rationally develop transportation and land use, and then to realize the sustainable development of social economy and ecological environment in this region.

2. Study Area

The Qinghai–Tibet Plateau, known as the "Roof of the World", the "Third Pole", and the "Asian Water Tower", is the largest plateau in China and the highest plateau in the world [36,37]. To the south, it reaches the southern edge of the Himalayas, and to the north, it reaches the northern edges of the Kunlun Mountains, Altun Mountains and Qilian Mountains. To the west, it connects the Pamirs and the Karakoram Mountains, and to the east, it connects the western section of the Qinling Mountains and the Loess Plateau. The Qinghai–Tibet Plateau lies between 26°00′ and 39°47′N and 73°19′ and 104°47′E. The study area is about 2800 km long from east to west, and 300–1500 km from south to north with a total area of 2.5 million km^2 [38]. Topographically, it can be divided into six parts, namely the Northern Qinghai–Tibet Plateau, the Southern Tibetan Valley, Qaidam Basin, Qilian Mountains, Qinghai Plateau and Sichuan–Tibet Alpine Valley. The overview of the Qinghai–Tibet Plateau is shown in Figure 2.

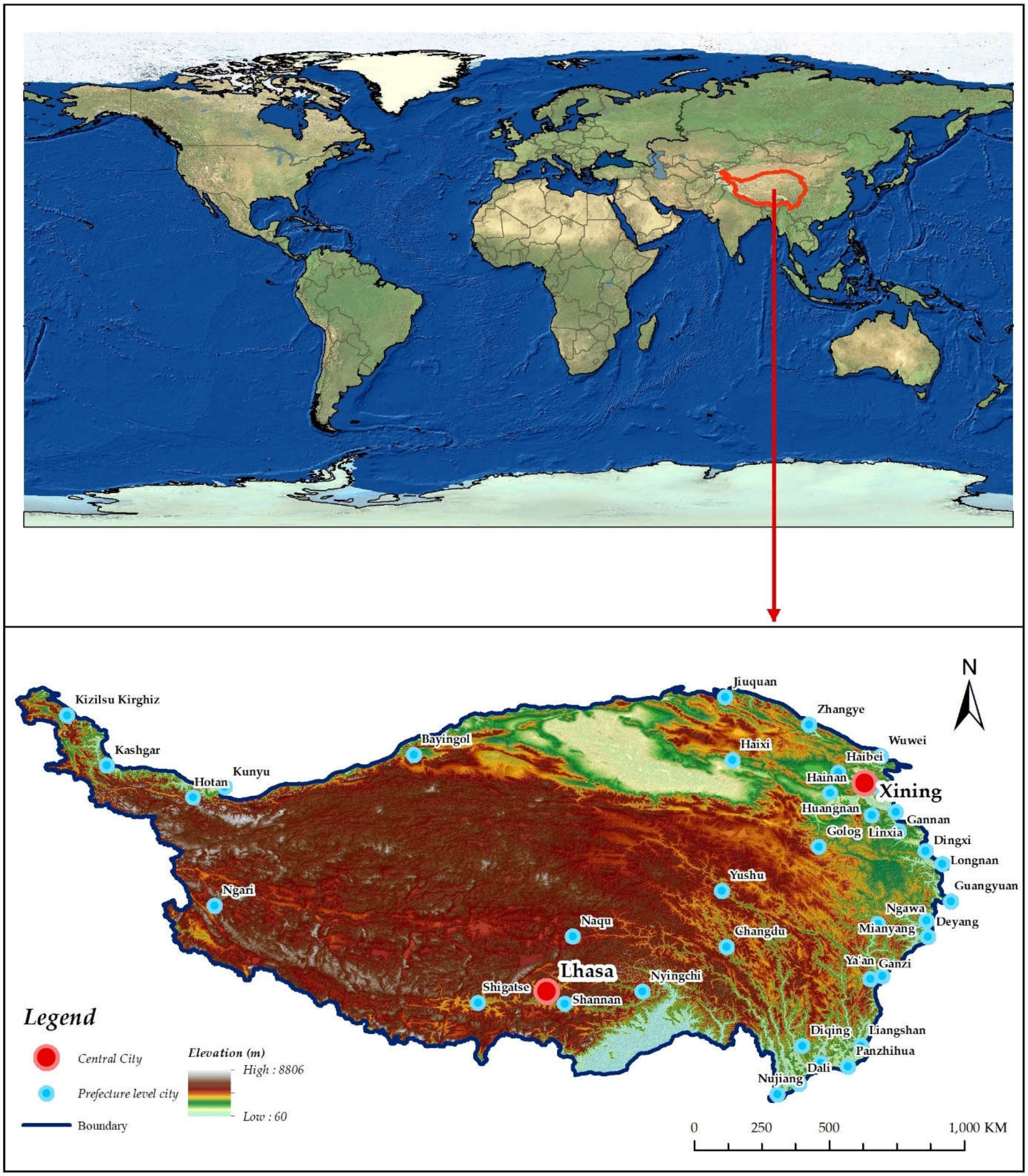

Figure 2. Overview of the Qinghai–Tibet Plateau.

From the perspective of China, the transportation area of the Qinghai–Tibet Plateau is remote, far from the three major economic and cultural centers in the east, and the development radiation capacity that can be received is relatively limited. Its transportation

distance is long and it is deeply affected by the complex terrain, which belongs to the type of lagging infrastructure development [39].

Figure 3 shows the main datasets in this research: 1) the administrative divisions datasets are supported by the China Basic Geographic Information Center; 2) the highway datasets of the Qinghai–Tibet Plateau in 2015 (includes freeways, 1st highway, 2nd highway, and 3rd highway, etc.), are derived from OpenStreetMap database; 3) passenger station datasets are obtained from Baidu Map POI (Points of Interest).

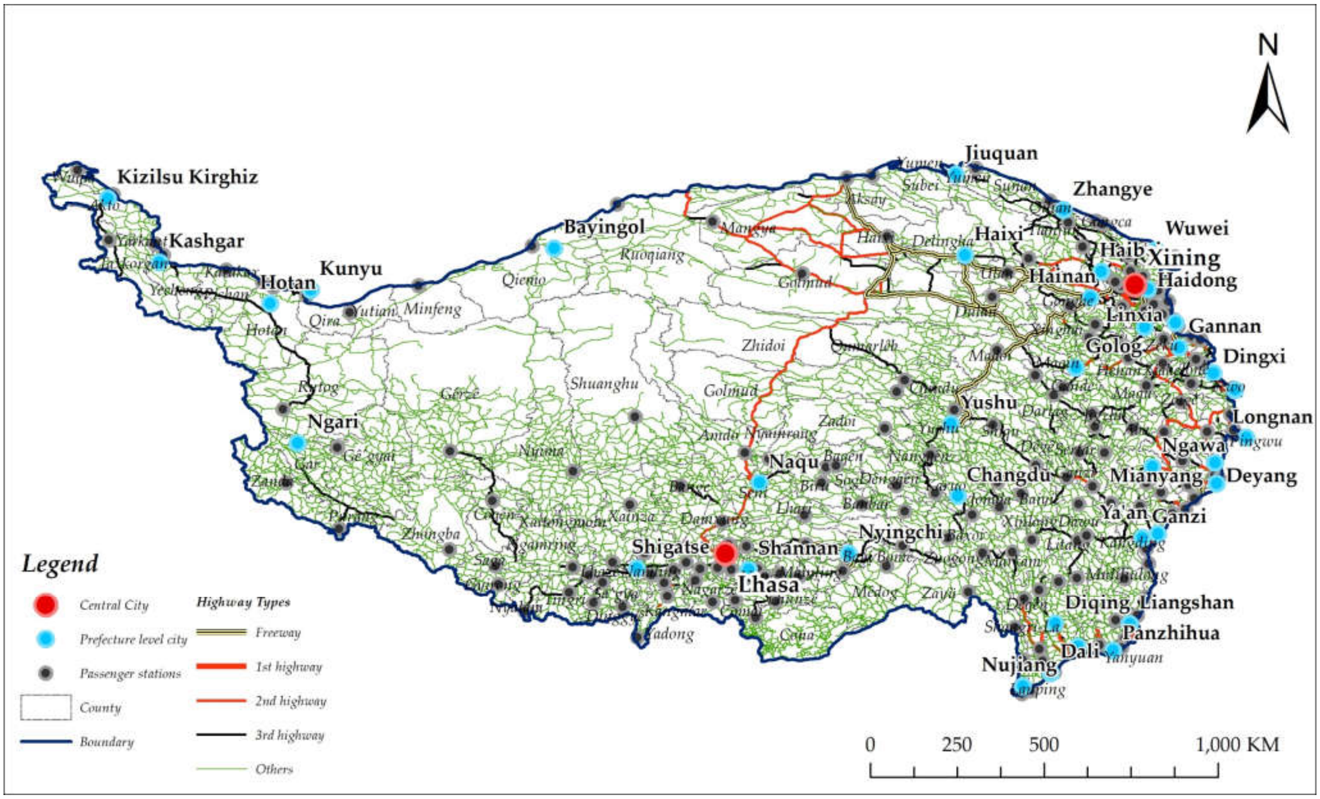

Figure 3. Spatial distribution of the main datasets.

3. Methods

Transport dominance is an integrated index to evaluate the merits and demerits of regional transportation. Its core is to judge regional traffic conditions from the relative perspective of quantitative methods on the larger regional system [40]. Regional transport dominance combines three aspects, including point, line, and area. Point reflects the spatial influence of the transport node. Line represents the spatial influence of the transport line, which could be linked by two nodes or two districts. Area shows the regional transport development [25]. The more significant the transport dominance is, the greater the conditions and potential for its economic development. Based on the analysis of the relevant research results, this paper constructs the evaluation index system of transport dominance from three perspectives, which includes quality, quantity, and advantage to measure the development level of transport infrastructure network and reflect its supportive capacity for its socio-economic activities in the Qinghai–Tibet Plateau. The weight of each indicator is determined by AHP to evaluate the highway transport dominance in the Qinghai–Tibet Plateau comprehensively.

3.1. Transport Dominance

Based on the relevant study results and characteristics of the Qinghai–Tibet Plateau, this paper selects the density of the highway network, the influence of trunk lines and the

spatial accessibility of highways as the evaluation index system for the comprehensive evaluation of the highway transportation dominance in the Qinghai–Tibet Plateau from three aspects: "quality", "quantity" and "advantage". The integration of three indicators can fully interpret regional highway transportation conditions. The density of the highway network and influence of trunk lines denote the supportive capacity and technological capability of highway transport facilities, while the spatial accessibility of highways reflects a node's location status in the whole study area.

1) **Density of highway network**. The density of the highway network refers to the ratio of the length of highways to the area of county. The higher the density of the highway network in the county, the better the traffic conditions in the county. Assume that the density of the highway network is D_i, L_{ij} is the length of the highway network, and $Area_i$ is the area of the county i. The density of the highway network in county i is calculated by Equation (1) below.

$$D_i = \sum_{j=1}^{m} P_{ij} \frac{L_{ij}}{Area_i} \quad i \in (1,2,3,\cdots,n) \tag{1}$$

In Equation (1), n is the number of counties; P_{ij} is the proportion of the j-th mode of transportation in county i, L_{ij} is length of the j-th mode of transportation. According to the situation of the Qinghai–Tibet Plateau, the P_{ij} values of freeway, 1st highway, 2nd highway, 3rd highway and others are given as 2.5, 2.0, 1.5, 1.0 and 0.5, respectively [41].

2) **Influence of trunk lines**. The influence of trunk lines is to evaluate the level of the traffic facilities and their influence on the regional development by using the method of scoring. Equation (2) is shown below:

$$C_i = \sum_{j=1}^{m} T_{ij} \quad i \in (1,2,3,\cdots,n) \tag{2}$$

In Equation (2), n is the number of counties, C_i refers to the influence of trunk lines in county i, j refers to the different types of highway, and T_{ij} refers to the influence of the trunk lines of type j in county i. With reference to the study results [42], and combined with the characteristics of the Qinghai–Tibet Plateau, the weight is assigned according to the situation of freeway, 1st, 2nd, 3rd, and others owned by the county. The weight assignment is shown in Table 1 (L_h refers to the buffer distance from the freeway).

Table 1. The weight of highway with different types.

Type	Criterion	Weight
Freeway	contains freeway exits	2.5
	$L_h \le 30$ km	2.0
	30 km < $L_h \le 60$ km	1.0
	$L_h > 60$ km	0
Ordinary highway	1st	2.5
	2nd	2.0
	3rd	1.5
	others	1

3) **Spatial accessibility of highways**. The accessibility is considered an important measurement index based on the transportation distance between nodes and the influence of their potential factors [43]. This study utilizes the shortest distance model to determine the accessibility of each county, as shown in Equation (3):

$$A_i = \sum_{j=1}^{n} I_{ij} \quad i,j \in (1,2,3,\cdots,n) \tag{3}$$

where A_i refers to the accessibility value of node i, I_{ij} is the shortest distance between two nodes with highway, and n is the total number of nodes in the county. The smaller the value of the accessibility coefficient, the better the spatial accessibility. It indicates that the traffic location conditions in the county are also better. On this basis, the accessibility of the entire transportation network A_{sum} is defined as shown in Equation (4):

$$A_{sum} = \sum_{i=1}^{n} A_i \quad i \in (1,2,3,\cdots,n) \tag{4}$$

The spatial accessibility coefficient in the region R_i is the ratio of the measurement value of the accessibility of each node to the average of the measurement value of accessibility of all nodes in the region, as shown in Equation (5):

$$R_i = \frac{A_i}{(A_{sum}/n)} \quad i \in (1,2,3,\cdots,n) \tag{5}$$

R_i can be used to indicate the relative accessibility situation between nodes in the entire highways network. The smaller the R_i, the better the accessibility condition. R_i is greater than 1, indicating that the accessibility of the node is lower than the average level in the area. On the contrary, its accessibility level is higher than the average level in the region.

4) The evaluation of transport dominance. The comprehensive evaluation of regional transport dominance is integrated by three indicators: density of the highway network, influence of trunk lines and spatial accessibility with highways. The values of these indicators have different effects on regional transport dominance. Therefore, standardization should be carried out in accordance with certain methods, and dimensionless processing should be carried out for the related indicator system, as shown in Equation (6):

$$Z_{ij} = \begin{cases} (y_{ij} - y_{min})/(y_{max} - y_{min}) \ (positive\ index) \\ (y_{max} - y_{ij})/(y_{max} - y_{min}) \ (reverse\ index) \end{cases} \tag{6}$$

where i=1, 2, 3, ……, n; j=1, 2, 3, ……, n, which are the total number of evaluation indexes and evaluation objects, respectively. y_{max} and y_{min} are the maximum and minimum values of different index i among all evaluation objects. Z_{ij} and Y_{ij} are the index values of different index i after and before dimensionless, as is shown in Equation (7):

$$F_i = \sum_{i=1}^{n} W_i * D_i \tag{7}$$

where F_i is the transport dominance in the study area, n is the total number of evaluation indicators, W_i is the weight of evaluation index, and D_i is the non-dimensionless evaluation index.

The weight of evaluation index W_i is determined by Analytic Hierarchy Process (AHP). AHP is a combination of qualitative and quantitative decision-making analysis methods [44–47]. It is a process of modeling and quantifying the decision process in a complex system. Using this method, decision makers can decompose complex issues into several levels and several factors and carry out simple comparisons and calculations among the various factors to get the weights of different schemes. AHP is often used for multi-objective, multi-criteria, multi-element, multi-level, unstructured and complex geographic decision-making problems, especially strategic decision-making problems.

3.2. Spatial Autocorrelation

The theory of spatial autocorrelation is used to reflect the spatial aggregation degree of a certain element attribute in the study area, which can be divided into global and local spatial autocorrelation [48]. Global Moran's *I* index and local Moran's *I* index are used in this study. Global Moran's *I* measures the interrelationship among spatial features as the whole, and the value of this index is between [−1, 1]. When the index is greater than 0, it

indicates a positive spatial correlation. The closer it is to 1, the higher the degree of aggregation. When the index is less than 0, it is a negative correlation. It is equal to 0, indicating that there is no spatial autocorrelation. The equation of global Moran's I calculation formula is shown in Equation (8) as follows:

$$I = \frac{n\sum_{i=1}^{n}\sum_{j=1}^{n} W_{ij}\,(x_i - \overline{x})(x_j - \overline{x})}{\sum_{i=1}^{n}\sum_{j=1}^{n} W_{ij}\sum_{i=1}^{n}(x_i - \overline{x})^2} = \frac{\sum_{i=1}^{n}\sum_{j=1}^{n} W_{ij}\,(x_i - \overline{x})(x_j - \overline{x})}{S^2\sum_{i=1}^{n}\sum_{j=1}^{n} W_{ij}} \quad (8)$$

where n is the sample size, that is, the number of spatial units. $S^2 = \frac{1}{n}\sum_{i=1}^{n}(x_i - \overline{x})^2$; $\overline{x} = \frac{1}{n}\sum_{i=1}^{n} x_i$; x_i, x_j are the attribute values of spatial unit i and j. W_{ij} represents the proximity relationship between spatial unit i and j. When i and j are adjacent spatial locations, W_{ij}=1, otherwise, W_{ij}=0.

The local Moran's I index measures the degree of spatial difference between the internal spatial units and the surrounding adjacent units in order to further investigate the heterogeneity of the local space within the area. The value of this index is between [−1, 1]. When the index is greater than 0, it indicates that the spatial difference between the attribute value of a certain unit and its neighboring units is small. The spatial pattern is expressed as High–High clustering or Low–Low clustering. When the index is less than 0, it indicates that the spatial difference between the attribute value of a certain unit and its neighboring units is large. The spatial pattern is expressed as High–Low differentiation or Low–High differentiation. When the value is equal to 0, it means that the spatial difference is not significant. The equation of local Moran's I_i is shown in Equation (9) below:

$$I_i = \frac{(x_i - \overline{x})}{S^2}\sum_{j} W_{ij}(x_j - \overline{x}) \quad (9)$$

where, S^2, x_i, x_j, $\overline{x}$ and W_{ij} have the same meanings as Equation (8).

4. Results

4.1. Density of Highways Network

In this paper, Equation (1) is used to calculate the density of highways at the county scale. According to the measured results and its spatial distribution, the study area is divided into five levels based on the principle of "the minimum difference within categories and maximum difference between categories"; it is shown in Figure 4, including the high-density area, relative high-density area, general area, relative low-density area, and low-density area.

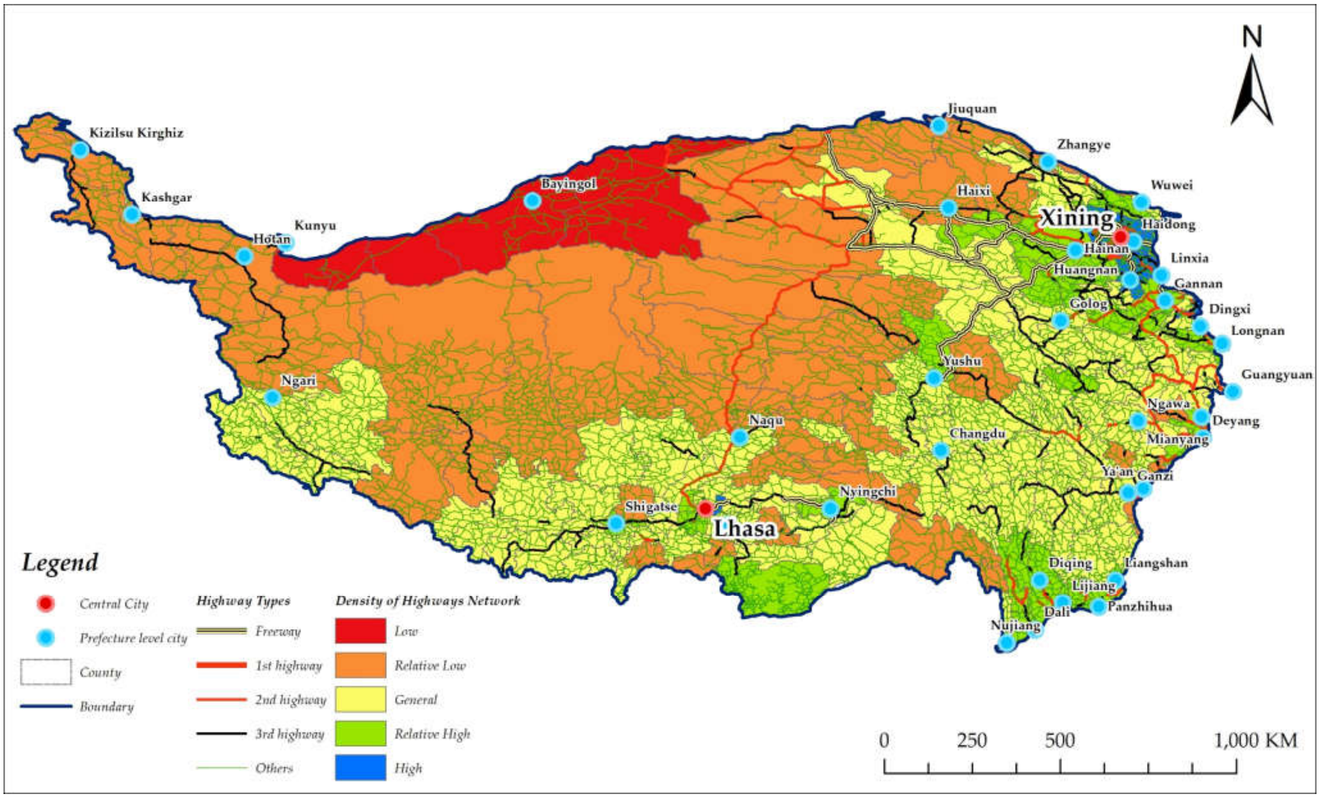

Figure 4. Density of highways network in the Qinghai–Tibet Plateau.

Overall, the density of the highway network in the Qinghai–Tibet Plateau is in a general condition. However, the development of the highways network between counties is uneven, showing a trend of becoming gradually sparse from east to west, from central cities to remote areas. There are two high-density transportation network areas on the Qinghai–Tibet Plateau. One is the urban agglomeration in the east with Xining as the center, and the other is the urban agglomeration in the south with Lhasa as the center. The traffic network density is lowest in the western and northern regions.

4.2. Influence of Trunk Lines

Based on the same processing principles as described in Section 4.1, the influence of highways in the Qinghai–Tibet Plateau is divided into five levels, including strong impact area, relative strong impact area, general impact area, relative weak impact area and weak impact area.

As is shown in Figure 5, the overall level of influence of trunk lines on the Qinghai–Tibet Plateau is relatively low. The degree of highway influence between counties is different, showing a trend of sparseness from east to west, from central cities to remote areas. Among them, strong impact areas are Golmud City in the middle and the urban agglomeration areas in the east with Xining as the center. Most of the surrounding areas of strong impact are areas with relatively strong impact. Areas with general impact are mainly connected to relative impact areas from the east, northeast, and southwest. Most of the areas with relative weak impact and weak impact areas are distributed in the western and southern areas.

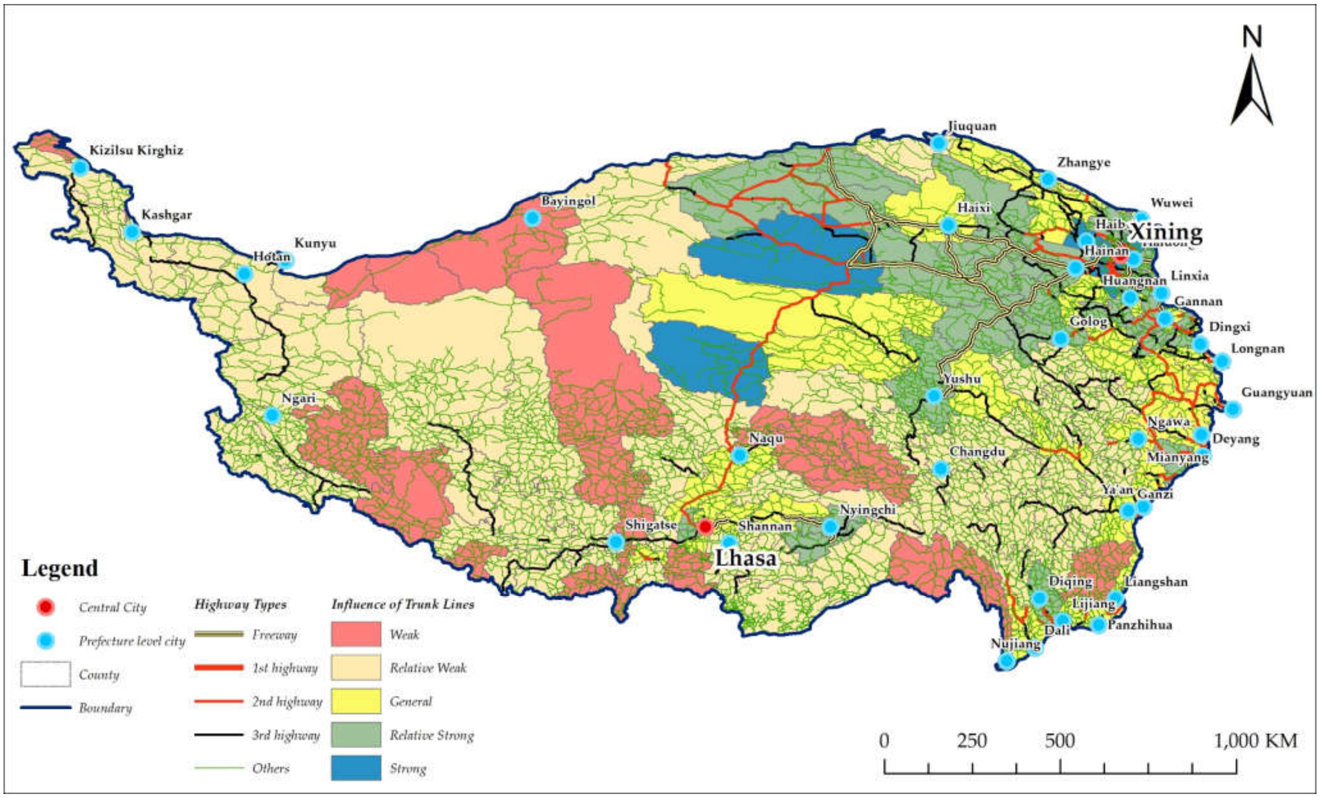

Figure 5. Influence of trunk lines in the Qinghai–Tibet Plateau.

4.3. Spatial Accessibility of Highways

Based on the highway dataset (freeway, 1st highways, 2nd highway, 3rd highway, and passenger stations, etc.) in the Qinghai–Tibet Plateau, this paper calculates the shortest travel distance of 209 counties with the Origin–Destination (OD) matrix and calculates the spatial accessibility of highway traffic using Equations (3), (4) and (5). According to the same processing principles as described in Section 4.1, the spatial accessibility of highways in the Qinghai Tibet Plateau are divided into five levels, including prominent area, good area, general area, poor area, and remote area.

As is shown in Figure 6, the spatial differentiation of highway accessibility in the Qinghai–Tibet Plateau shows a trend of decreasing from the center to the outer circle. The accessibility of highways in the eastern region is higher than that in the western region. The prominent areas are concentrated in the circle with the Xining–Lhasa axis as the core, with the shortest travel distance and the best highway accessibility. Its periphery is a good area with Lhasa, Xining and Chengdu as the core. The general area is connected with good areas mainly from the east, northeast, and southwest. The poor area is the sub-outer area of the Qinghai–Tibet Plateau, which is close to the general area from the east. The remote areas are mainly distributed in the northwest corner of the Qinghai–Tibet Plateau, including Hotan, Kashgar, and Kizilsu Kirgiz, with the highest traffic cost and the lowest accessibility.

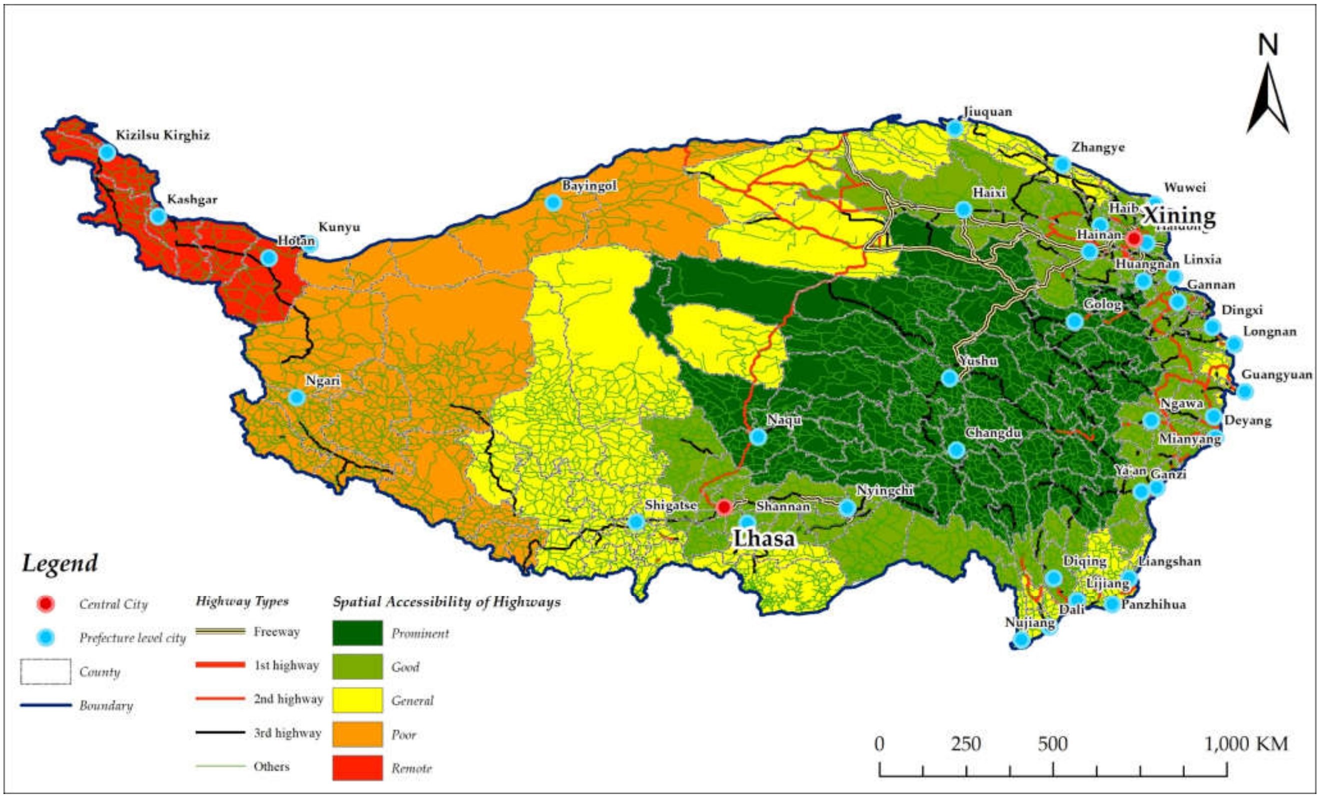

Figure 6. Spatial accessibility of highways in the Qinghai–Tibet Plateau.

4.4. Highway Transport Dominance

After the calculation of the three main indexes (density of highways network, influence of trunk lines, and spatial accessibility of highways), the weight of each index is determined by the AHP method, which are 0.088, 0.243, and 0.669, respectively, as shown in Table 2.

Table 2. Judgment matrix of Analytic Hierarchy Process for the calculation of transport dominance index of the Qinghai–Tibet Plateau.

Decision Objective	Density of highways network	Influence of trunk lines	Spatial accessibility of highways
Density of highways network	1	1/3	1/7
Influence of trunk lines	3	1	1/3
Spatial accessibility of highways	7	3	1

Then, the transport dominance index of the Qinghai–Tibet Plateau is calculated by using Map Algebra, as is shown in the Figure 7.

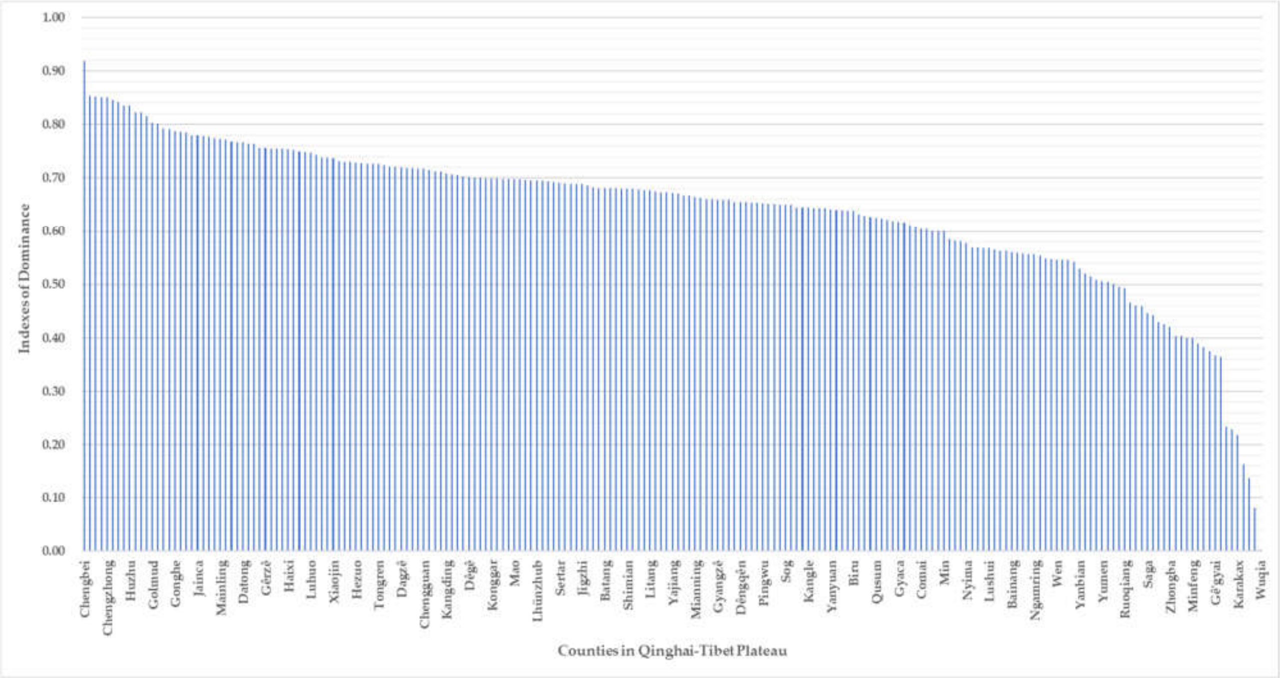

Figure 7. Transport Dominance Index of the Qinghai–Tibet Plateau at the county scale.

The transport dominance is divided in five levels by Natural Break, namely, high area, relative high area, general area, relative low area, and low area. The classification of Natural Break is based on the natural grouping in the data, and identifies the classification interval, which can be the most appropriate similarity, and maximizes the differences between classes [49]. As is shown in Table 3 below, there are 45 counties with a high grade of transport dominance in the Qinghai–Tibet Plateau, accounting for 21.53% of the total counties in the study area, and the land area is 583,639.50 km^2, accounting for 22.48% of the total area of the study area. A total of 97 counties belong to the relative high area, accounting for 46.41% of the total counties, and the land area is 831,603.45 km^2, accounting for 32.04% of the total area. There are 43 counties with general dominance, which is 20.57% of all counties, and it covers a land area of 720,643.25 km^2, which is 27.76% of the total area. A total of 17 counties are the relative low area, accounting for 8.13% of the total counties, and the land area is 341,422.76 km^2, accounting for 13.15% of the total area. Finally, there are 7 counties in the low area, accounting for 3.34% of the total counties, and the land area is 118,566.06 km^2, accounting for 4.57% of the total area.

Table 3. Statistics of highway transport dominance in the Qinghai–Tibet Plateau at county level.

Classification	Threshold	Number of counties	Proportion* of total number (%)	Area (km^2)	Proportion* of total area (%)
low	0–0.23	7	3.35	118566.06	4.57
relative low	0.23–0.47	17	8.13	341422.76	13.15
general	0.47–0.62	43	20.57	720643.25	27.76
relative high	0.62–0.73	97	46.41	831603.45	32.04
high	0.73–0.92	45	21.53	583639.50	22.48

*Note: The calculation of the proportion is based on rounding.

As shown in Figure 8, it is found that at the county scale, the difference of the highway transport dominance between Tibet and Qinghai is obvious. In Tibet, the range of highway transport dominance is from 0.364 to 0.785. There are six counties with a high

grade of transport dominance, accounting for 8.11% of the total counties. There are 34 counties with a relative-high grade of transport dominance, accounting for 45.94% of the total counties. There are 26 counties with a general grade of transport dominance, accounting for 35.14% of the total counties. There are eight counties with a general grade of transport dominance, accounting for 10.81% of the total counties. In Qinghai, the range of highway transport dominance is from 0.654 to 0.918. There are 31 counties with a high grade of transport dominance, accounting for 68.89% of the total counties. There are 14 counties with a relative-high grade of transport dominance, accounting for 31.11% of the total counties.

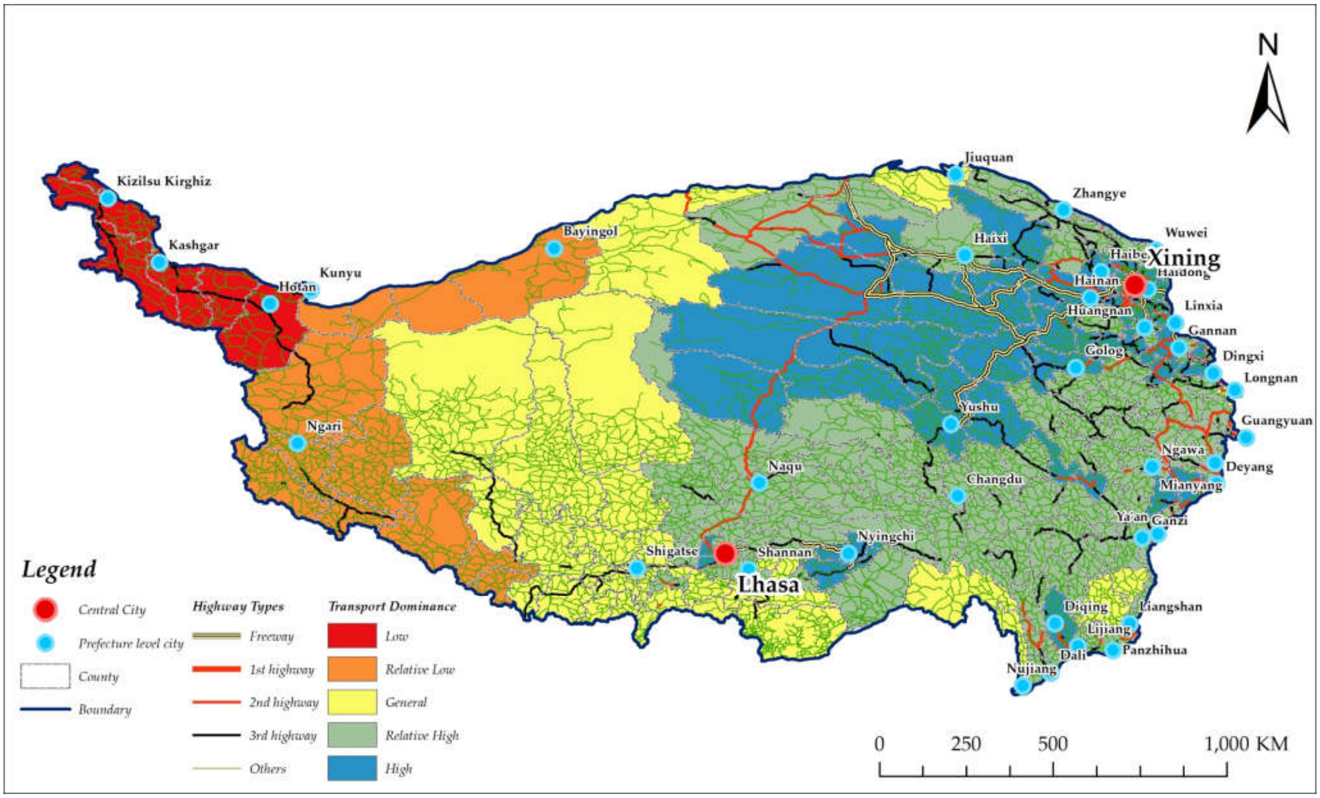

Figure 8. Distribution of highway transport dominance in the Qinghai–Tibet Plateau.

In general, the spatial pattern of highway transport dominance in the Qinghai–Tibet Plateau shows a phenomenon of the irregular circle gradually decreasing from the central area to the periphery. On the whole, the spatial difference varies greatly. With Lhasa, Xining, and Golmud as the center, the eastern region presents a "strong center-weak periphery" mode. The transport dominance gradually decreases from Lhasa, Xining, and the eastern dense urban areas to the surrounding regions. The overall development level of Golmud City is relatively high, and the transportation infrastructure and location conditions are more prominent in the Qinghai–Tibet Plateau. The western region presents a gradual-decay ring structure.

From the perspective of spatial pattern, there is a strong spatial autocorrelation of highway transport dominance in the Qinghai–Tibet Plateau. In terms of global spatial autocorrelation, as shown in Figure 9, the Moran's I is 0.452, which is much greater than 0. It also passes the test of significance level of 0.001, which indicates that the highway transport dominance in the Qinghai–Tibet Plateau presents a strong spatial autocorrelation, showing a significant agglomeration distribution trend.

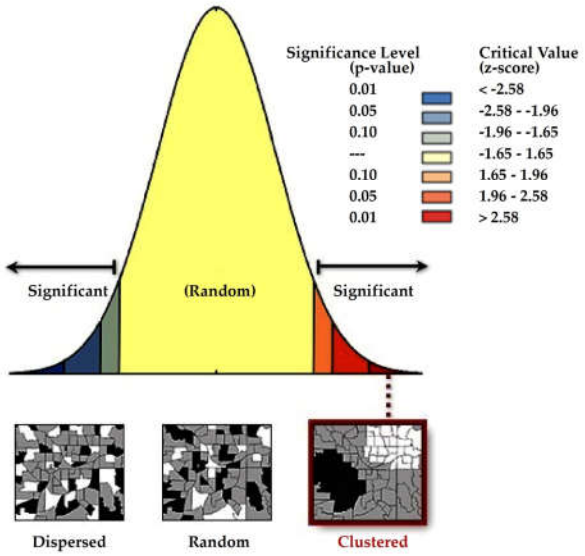

Figure 9. Global Moran's *I* index of highway transport dominance in Qinghai–Tibet Plateau.

As shown in Figure 10, from the perspective of local spatial autocorrelation, the highway transport dominance in the Qinghai–Tibet Plateau presents a spatial agglomeration pattern and shows spatial heterogeneity. In the local Moran's I spatial pattern, the High–High agglomeration area are mainly concentrated in 34 counties of Xining City and its surrounding counties, including Guinan, Gonghe, Xinghai, etc. The Low–Low agglomeration area are mainly concentrated in 22 counties, including Qiemo, Minfeng, Hotan, Karakax, etc.

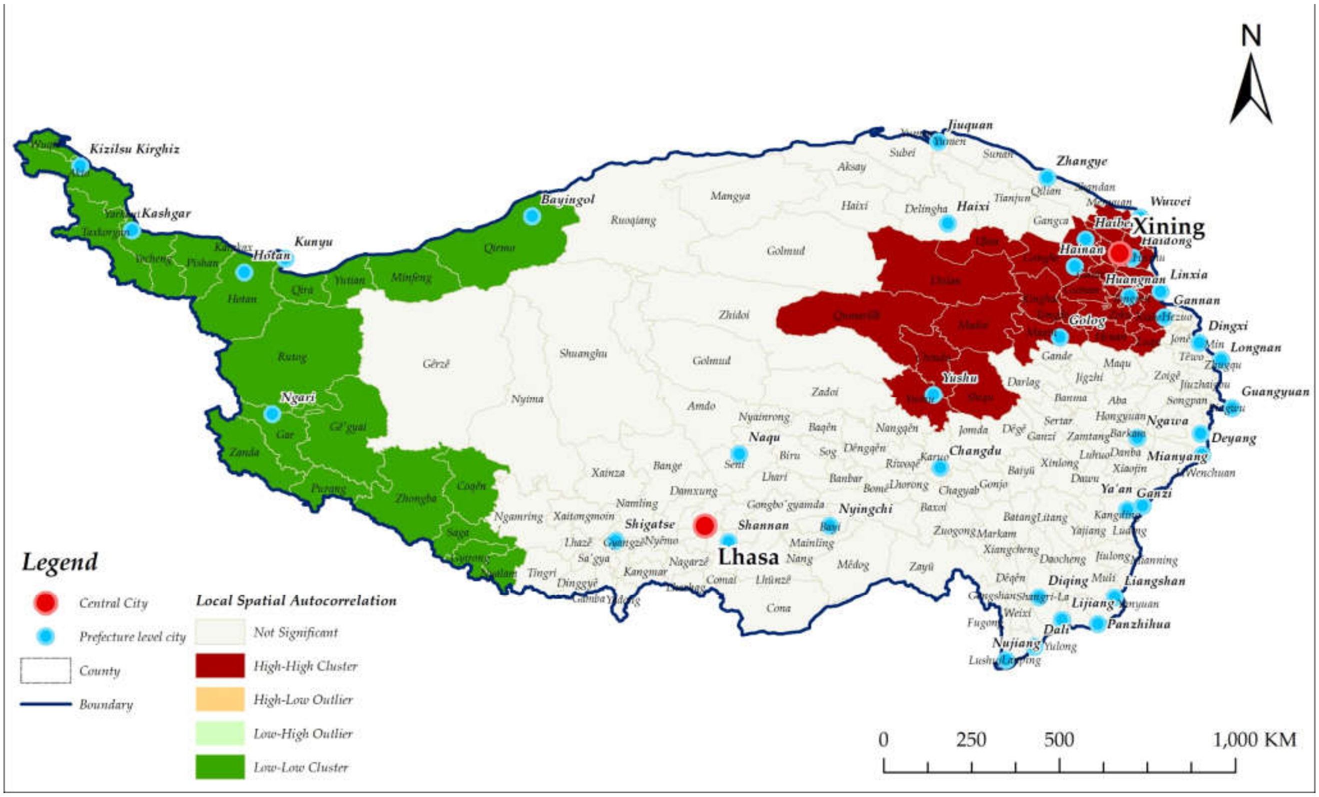

Figure 10. Local spatial autocorrelation of highway transport dominance in the Qinghai–Tibet Plateau.

5. Discussion

5.1. The Influence of Urban Location on Transport Dominance

From the perspective of the spatial pattern regularity, urban location has an obvious constraint relationship on the degree of transport dominance, showing a certain degree of the spatial convergence effect. In Figure 11, this paper selects Lhasa and Xining, two central cities in the Qinghai–Tibet Plateau, as the study objects, and takes 40 km, 80 km, 120 km, 160 km, 200 km, 240 km, 280 km, and 320 km, respectively, as buffer distances. Then, the highway transport dominance at each distance is identified and analyzed.

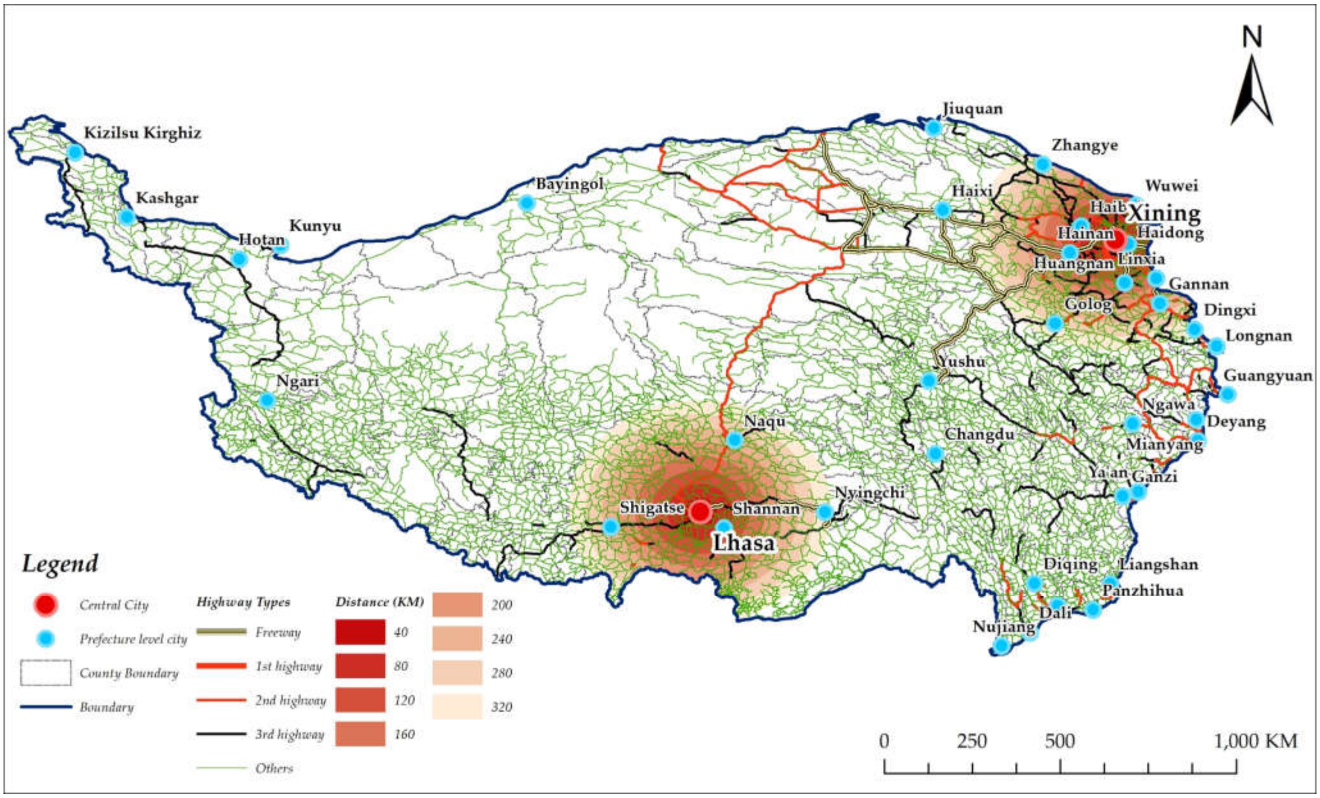

Figure 11. Influence area of cities of Xining and Lhasa in the Qinghai–Tibet Plateau.

According to the influence area (buffer zone) generated in Figure 11, the average value of highway transport dominance of each county in different influence areas is respectively counted. As shown in Figure 12, the increase in the county's highway transport dominance basically obeys a decreasing pattern as its distance from the central city decreases. For the highway transport dominance centered on Xining, the fitting accuracy of the correlation with the distance between the county and Xining is 0.735 (R^2=0.735). This value is lower than the fitting accuracy result of the highway transport dominance centered on Lhasa (R^2=0.9412). This is mainly due to the fact that Qinghai Province has been accelerating the construction of an integrated transportation system in recent years and has established two national comprehensive transportation hubs in Xining and Golmud. In terms of highways, it has initially established the radial highway network, which is expressed as "The south to Tibet, the north to Hexi Corridor, the east to Xining, and the west to Xinjiang". Xining has become an important node of the "cross-shaped" transportation hub connecting Gansu, Xinjiang, Qinghai, and Tibet. The rapid development of Golmud's comprehensive transportation system has also effectively improved the transport dominance of the surrounding areas, which in turn affected the spatial convergence of Xining, which is only about 750 km away, on the dominance of the surrounding areas.

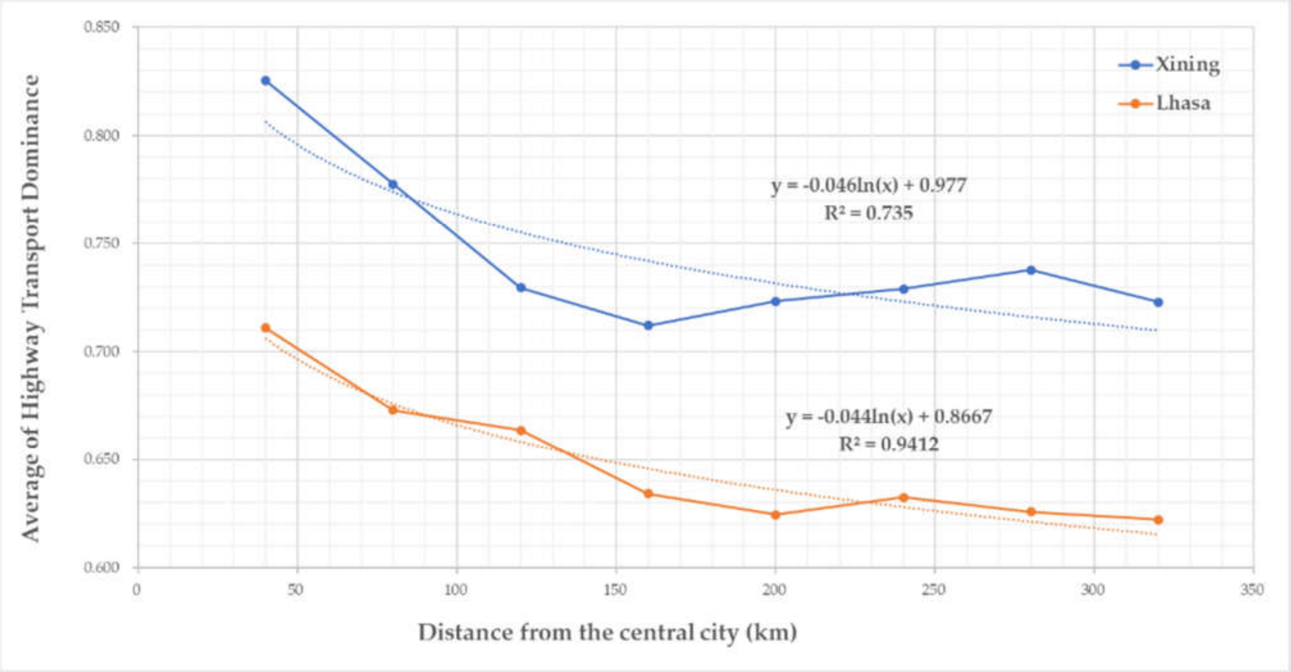

Figure 12. Relationship between the distance from the central city and highway transport dominance.

Meanwhile, there are two major characteristics that need to be further pointed out: 1) the absolute value of the highway transport dominance shows an upward trend; and 2) the surrounding area of the central city maintains a relatively high-intensity traffic infrastructure construction situation, and the highway traffic advantage has a trend of spatial distance convergence. The closer the area to the central city, the higher the transport dominance. The growth rate of this indicator is inversely proportional to distance.

5.2. Highway Network Distribution in Urban Agglomerations

During the "13th Five-Year Plan" period, China's urban agglomerations have accelerated development, forming the layout of (19+2) urban agglomerations (the "2" represents the urbanized areas centered on Lhasa and Kashgar). The urban agglomerations basically cover the key and optimized development zones in China's main functional zones. The study area of the Qinghai–Tibet Plateau mainly covers the Lan-Xi Urban Agglomeration, Lhasa Urban Agglomeration and Kashgar Urban Agglomeration, and their spatial distribution is shown in Figure 13.

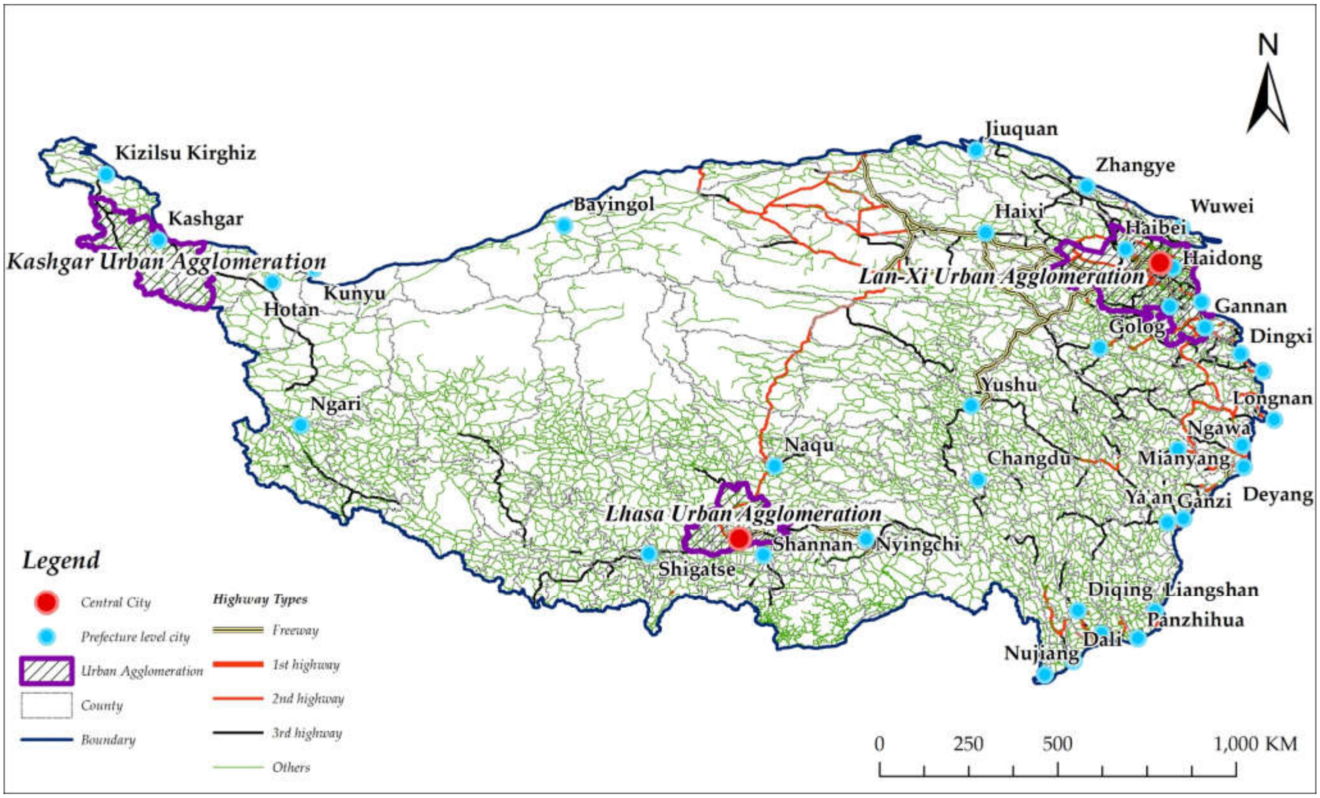

Figure 13. Distribution of urban agglomerations in the Qinghai–Tibet Plateau.

Based on the spatial distribution of urban agglomerations in the Qinghai–Tibet Plateau, the highway transport dominance of three urban agglomerations is calculated, as shown in Figure 14. In the Lan-Xi urban agglomeration, the average value of highway transport dominance is 0.784. Specifically, the average value of the density of the highway network is 0.224. The average value of influence of trunk lines is 0.754. The average value of spatial accessibility is 0.869. In the Lhasa urban agglomeration, the average value of highway transport dominance index is 0.711. Specifically, the average value of density of highway network is 0.082, and the average value of influence of trunk lines is 0.863. The average value of spatial accessibility index is 0.884. In the Kashgar urban agglomeration, the average value of highway transport dominance index is 0.15. Specifically, the average value of density of highway network is 0.031. The average value of the influence of trunk lines is 0.176. The average value of spatial accessibility is 0.156. It can be seen that with the implementation of "Western Development", "One Belt, One Road" and other national macro strategies, the Lan-Xi urban agglomeration and Lhasa urban agglomeration have significantly higher highway transport dominance than other regions [50–52]. This shows that the transportation infrastructures of these two urban agglomerations are relatively complete. The traffic conditions are also significantly improved, and the regional accessibility is getting better. However, due to the comprehensive influence of the social economy, topography and landforms, the highway transport dominance of the Kashgar urban agglomeration is significantly lower than that of the above two city circles. Considering the fragile and sensitive ecological environment of the Qinghai–Tibet Plateau, how to implement the concept of sustainable development and formulate scientific and reasonable transportation infrastructure planning, development goals and corresponding policies in urban agglomeration areas with different levels of highway infrastructure development is an issue worthy of further in-depth discussion.

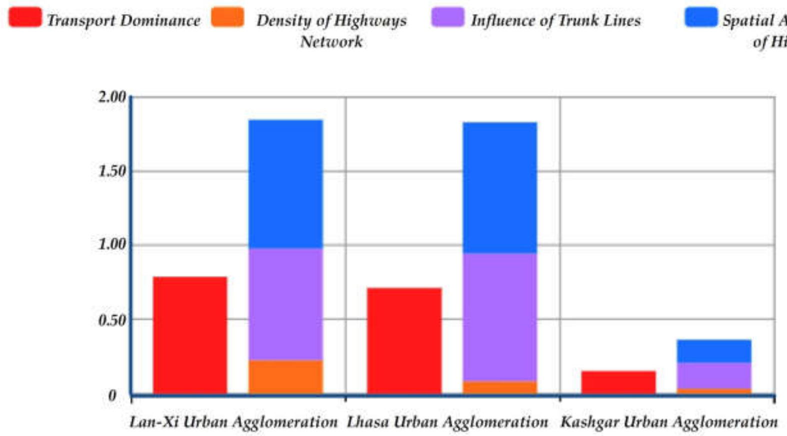

Figure 14. Statistics of highway transport dominance in three urban agglomerations in the Qinghai–Tibet Plateau.

6. Conclusions

Qinghai–Tibet is the international transportation hub between east and west Asia, joining south and north Asia and the South and North Silk Road, appearing much like ribbons surrounding these areas [53–55]. This paper is an application of various geospatial analysis methods (network analysis, O–D network, spatial statistics, weighted overlay, etc.) to provide an overall outline of the spatial distribution pattern and spatial highways convergence of the highest and largest plateau in the world. The study has important practical significance to the Qinghai–Tibet Plateau for high-quality and sustainable development. It also provides a clear structural picture to the government for the development of sustainable transport in the region with special, fragile and sensitive ecosystems in the world.

From the perspective of transport dominance spatial distribution at the county scale, the "hub-and-spoke system effect" of the central city on highway transport dominance is significant, as the central cities of growth poles, such as Lhasa, Xining, and Golmud, have outstanding advantages both in the driving role of the region and in their own development. It can be found that counties with higher highway transport dominance are mostly distributed around the central cities in their respective regions, and counties with higher dominance are also distributed in the transportation corridors between the related central cities, which shows that the central cities have obvious radiating and driving effects on them. Counties with low and relative-low highway transport dominance are distributed in the western region in patches. These areas have imperfect traffic lines, a bad natural environment, sparse population density and a correspondingly low development level.

From the perspective of spatial pattern, the highway transport dominance in the Qinghai–Tibet Plateau presents strong spatial autocorrelation and spatial agglomeration patterns (High–High pattern and Low–Low pattern) around Lhasa, Xining, and Golmud as the centers. Meanwhile, highway transport dominance in the Qinghai–Tibet Plateau shows a certain spatial heterogeneity, among which high-value areas are mainly distributed in Dulan, Yushu, Xinghai, etc. These regions have great vitality, social and economic development, and have unique advantages in terms of accessibility within and outside the region. Low-value areas are mainly distributed in counties of Qiemo, Minfeng, Rutog, Yutian, etc. These regions are in the Kunlun Mountains, Altun Mountains and other regions with complex geomorphic conditions, a fragile ecological environment, and high engineering constructions costs, leading to generally low transport dominance in these areas.

There is a certain spatial convergence effect of the central cities on highway transport dominance. It is not difficult to see that, although there are obvious spatial differences in the highway transport dominance in the Qinghai–Tibet Plateau, the changes in

development patterns are continuous. The core-periphery layer structures with Lhasa and Xining are obvious. This indicates that the inner spatial convergence effect of the circle structure with Lhasa and Xining as the core is relative strong, and the local driving effect on the regional transportation development is prominent. The spatial structure gradient of highway transport dominance is distinct, and the expansion of the circle layer area is obvious. For the spatial trend of highway transport dominance in the Qinghai–Tibet Plateau, on the one hand, it reflects the reality of the unbalanced and inadequate development of infrastructure in the plateau region [51,52]. On the other hand, it also reflects the necessity and urgency of implementing a regional, coordinated development strategy.

Author Contributions: Conceptualization, Zhiheng Wang and Dongchuan Wang; methodology, Zhiheng Wang, Hongkui Fan; software, Tao Xing; validation, Daikun Wang and Qiaozhen Guo; formal analysis, Zhiheng Wang and Dongchuan Wang; investigation, Lina Xiu; resources, Dongchuan Wang; data curation, Hongkui Fan and Tao Xing; writing—original draft preparation, Zhiheng Wang; writing—review and editing, Zhiheng Wang and Daikun Wang; visualization, Zhiheng Wang, Hongkui Fan and Tao Xing; supervision, Daikun Wang; project administration, Dongchuan Wang and Zhiheng Wang; funding acquisition, Dongchuan Wang. All authors have read and agreed to the published version of the manuscript.

Funding: This research was funded by the Second Tibetan Plateau of Scientific Expedition and Research Program (STEP), grant number 2019QZKK0608.

Data Availability Statement: The data presented in this study are available from the corresponding author (W.D) on reasonable request.

Acknowledgments: The authors acknowledge Institute of Tibetan Plateau Research, Chinese Academy of Sciences for providing basic geographic and thematic data of Tibetan Plateau. The authors would also like to thank the editors and anonymous reviewers for their thorough and valuable comments and suggestions.

Conflicts of Interest: The authors declare no conflict of interest.

References

1. Gao, Q.; Miao, Y.; Song, J. Research progress on the sustainable development of Qinghai–Tibet Plateau. *Geogr. Res.* **2021**, *40*, 1–17.
2. Wang, W.; Yang, W.; Cao, X. Road transport superiority degree and impact on economic growth in the concentrated contiguous severe poverty areas in China. *Prog. Geogr.* **2015**, *34*, 665–675.
3. Liu, D.; Jin, F. Development level and coordination of highway networks of prefecture-level administrative regions in China. *Prog. Geogr.* **2014**, *33*, 241–248.
4. Mahdinia, I.; Habibian, M.; Habibian, M.; Gudmundsson, H. An indicator-based algorithm to measure transportation sustainability: A case study of the U.S. states. *Ecol. Indic.* **2018**, *89*, 738–754.
5. Allen, J.; Farber, S. Planning transport for social inclusion: An accessibility-activity participation approach. *Transp. Res. Part D* **2020**, *78*, 102212.
6. Qi, W.; Liu, S.; Zhou, L. Regional differentiation of population in Tibetan Plateau: Insight from the "Hu Line". *Acta Geogr. Sin.* **2020**, *75*, 255–267.
7. Jiang, W.; Lü, Y.; Liu, Y.; Gao, W. Ecosystem service value of the Qinghai–Tibet Plateau significantly increased during 25 years. *Ecosyst. Serv.* **2020**, *44*, 101146.
8. Pritchard, J.P.; Moura, F.; e Silva, J.D.A.; Martinez, L.M. Spatial analysis of transportation-related social exclusion in the Lisbon metropolitan area. *Procedia Soc. Behav. Sci.* **2014**, 440-449.
9. Hansen, W.G. How Accessibility Shapes Land Use. *J. Am. Inst. Plan.* **1959**, *25*, 73–76.
10. Wachs, M.; Kumagai, T.G. Physical accessibility as a social indicator. *Socio Econ. Plan. Sci.* **1973**, *7*, 437–456.
11. Maćkiewicz, A.; Ratajczak, W. Towards a new definition of topological accessibility. *Transp. Res. Part B Methodol.* **1996**, *30*, 47–79.
12. Guzman, L.A.; Oviedo, D. Accessibility, affordability and equity: Assessing 'pro-poor' public transport subsidies in Bogotá. *Transp. Policy* **2018**, *68*, 37–51.
13. Cohen, T. Tools for addressing transport inequality: A novel variant of accessibility measurement. *J. Transp. Geogr.* **2020**, *88*, 102863.
14. Holl, A. Twenty years of accessibility improvements. The case of the Spanish motorway building programme. *J. Transp. Geogr.* **2007**, *15*, 286–297.

15. Li, M.; Guo, R.; Li, Y.; He, B.; Fan, Y. The Distribution Pattern of the Railway Network in China at the County Level. *Intern. J. Geo Inf.* **2019**, *8*, 336.
16. Murayama, Y. The impact of railways on accessibility in the Japanese urban system. *J. Transp. Geogr.* **1994**, *2*, 87–100.
17. Zhu, X.; Liu, S. Analysis of the impact of the MRT system on accessibility in Singapore using an integrated GIS tool. *J. Transp. Geogr.* **2004**, *12*, 89–101.
18. Dixit, M.; Sivakumar, A. Capturing the impact of individual characteristics on transport accessibility and equity analysis. *Transp. Res. Part D* **2020**, *87*, 102473.
19. Cao, X.; Xue, D.; Yan, X. A study on the urban accessibility of national trunk highway system in China. *Acta Geogr. Sin.* **2005**, *60*, 903–910.
20. Adhvaryu, B.; Chopde, A.; Dashora, L. Mapping public transport accessibility levels (PTAL) in India and its applications: A case study of Surat. *Case Stud. Transp. Policy* **2019**, *7*, 293–300.
21. Kwan, M.-P.; Murray, A.T.; O'Kelly, M.E.; Tiefelsdorf, M. Recent advances in accessibility research: Representation, methodology and applications. *J. Geogr. Syst. Vol.* **2003**, 129-138.
22. Páeza, A.; Scott, D.M.; Morency, C. Measuring accessibility: Positive and normative implementations of various accessibility indicators. *J. Transp. Geogr.* **2012**, *25*, 141–153.
23. Zheng, X.; Guan, X.; Wu, H.; Xiao, H. A Data-Driven Quasi-Dynamic Traffic Assignment Model Integrating Multi-Source Traffic Sensor Data on the Expressway Network. *Intern. J. Geo Inf.* **2021**, *10*, 113.
24. Croce, A.I.; Musolino, G.; Rindone, C.; Vitetta, A. Transport System Models and Big Data: Zoning and Graph Building with Traditional Surveys, FCD and GIS. *Intern. J. Geo Inf.* **2019**, *8*, 187.
25. Jin, F.; Wang, C.; Liu, X.; Wang, J.E. China's regional transport dominance:Density, proximity, and accessibility. *J. Geogr. Sci.* **2010**, *20*, 295–309.
26. Jin, F.; Chen, Z. Evolution of transportation in China since reform and opening-up: Patterns and principles. *Acta Geogr. Sin.* **2019**, *74*, 1941–1961.
27. Sun, W.; Zhang, Y. Assessment of Transportation Superiority Degree in Shanxi Province. *Prog. Geogr.* **2010**, *29*, 1562–1569.
28. Wang, C.; Wang, L.; Liu, R.; Yao, M. Empirical Research on Evaluation Model of Transport Superiority Degree—A Case study of Shandong Province. *Hum. Geogr.* **2010**, *25*, 73–76.
29. Zhou, N.; Hao, J.; Xing, T.; Wang, J.; Hong, S. The Spatial Configuration of Transport Superiority in Huang-Huai-Hai Plain. *Econ. Geogr.* **2012**, *32*, 91–96.
30. Miao, Y.; Wang, B. Zoning scheme of climatic influence for asphalt pavement in China. *J. Traffic Transp. Eng.* **2007**, *7*, 64–69.
31. Li, S.; Gong, J.; Deng, Q.; Zhou, T. Impacts of the Qinghai–Tibet Railway on Accessibility and Economic Linkage of the Third Pole. *Sustainability* **2018**, *10*, 3982.
32. Sun, C. Evaluation of county-level transport superiority degree in Qinghai province. Master's Thesis, Qinghai Normal University, Xining, China, 2017.
33. Li, L.; Wu, Q. Analysis on the traffic coverage of Qinghai Province based on the road network accessibility. *J. Xi'an Univ. Archit. Technol.* **2018**, *50*, 233–241.
34. Miao, Y.; Liu, H.; Song, J.; Dai, T. Research Progress of Transportation Facilities Construction and Their Impact Assessment in the Qinghai Tibet Plateau. *Adv. Earth Sci.* **2020**, *35*, 308–318.
35. Zhang, X.; Ge, Q. The structure, characteristic of land use in Tibetan Plateau and its rationed development strategy. *Chin. J. Agric. Resour. Reg. Plan.* **2002**, *23*, 14–19.
36. Qiu, J. China: The third pole. *Nature* **2008**, *454*, 393–396.
37. Immerzeel, W.W.; Lutz, A.F.; Andrade, M.; Bahl, A.; Biemans, H.; Bolch, T.; Hyde, S.; Brumby, S.; Davies, B.J.; Elmore, A.C.; et al. Importance and vulnerability of the world's water towers. *Nature* **2020**, *577*, 364–369.
38. Zhang, Y.; Li, B.; Zheng, D. A discussion on the boundary and area of the Tibetan Plateau in China. *Geogr. Res.* **2002**, *21*, 1–8.
39. Jin, F.; Liu, Y. Threshold study on transportation and industrial development in Qinghai–Tibet Plateau. *J. Nat. Resour.* **2000**, *15*, 363–368.
40. Jin, F.; Wang, C.; Li, X. Discrimination Method and Its Application Analysis of Regional Transport Superiority. *Acta Geogr. Sin.* **2008**, *63*, 787–798.
41. Meng, D.; Shen, J.; Lu, Y. Spatial Coupling between Transportation Superiority and Economy in Central Plain Economic Zone. *Econ. Geogr.* **2012**, *32*, 7–14.
42. Cheng, J.; Wang, C.; Liu, W. Distribution Configuration and Spatial Differentiation of Transport Superiority Degree in Northwest China. *Adv. Earth Sci.* **2016**, *31*, 192–205.
43. Li, P.; Lu, Y. Review and Prospectation of Accessibility Research. *Prog. Geogr.* **2005**, *24*, 69–78.
44. Colak, H.E.; Memisoglu, T.; Gercek, Y. Optimal site selection for solar photovoltaic (PV) power plants using GIS and AHP: A case study of Malatya Province, Turkey. *Renew. Energy* **2020**, *149*, 565–576.
45. Kittipongvises, S.; Phetrak, A.; Rattanapun, P.; Brundiers, K.; Buizer, J.L.; Melnick, R. AHP-GIS analysis for flood hazard assessment of the communities nearby the world heritage site on Ayutthaya Island, Thailand. *Int. J. Disaster Risk Reduct.* **2020**, *48*, 101612.
46. Amini, S.; Rohani, A.; Aghkhani, M.H.; Abbaspour-Fard, M.H.; Asgharipour, M.R. Assessment of land suitability and agricultural production sustainability using a combined approach (Fuzzy-AHP-GIS): A case study of Mazandaran province, Iran. *Inf. Process. Agric.* **2020**, *7*, 384–402.

47. Guler, D.; Yomralioglu, T. Suitable location selection for the electric vehicle fast charging station with AHP and fuzzy AHP methods using GIS. *Ann. GIS* **2020**, *26*, 169–189.
48. Stankov U.; Tanja, A.; Michal, K.; Vanja, P.; Marija, C.; Nataša, D.-K. Spatial autocorrelation analysis of tourist arrivals using municipal data: A Serbian example. *Geogr. Pannonica* **2017**, *21*, 106–114.
49. Fariza, A.; Basofi, A.; Aryani, M.D. Spatial Mapping of Diphtheria Vulnerability Level in East Java, Indonesia, using Analytical Hierarchy Process—Natural Break Classification. *J. Phys. Conf. Ser.* **2021**, *1803*, 012009.
50. Gao, X. Impact of Transport Accessibility on Land Use Change in the Qinghai–Tibet Plateau. Ph.D. Thesis, Shanxi Normal University, Xi'an, China, 2019. doi: 10.27292/d.cnki.gsxfu.2019.001788
51. Gao, X.; Cao, X.; Li, T.; Lv, M. Evolution of accessibility spatial pattern of the Qinghai–Tibet Plateau in 1976-2016. *Acta Geogr. Sin.* **2019**, *74*, 1190–1204.
52. Liu, Z.; Feng, X.; Wu, S.; Kong, L.; Yao, X. Spatio-temporal Dynamics of the Urban-Rural Construction Land and Ecological Land on Qinghai–Tibet Plateau. *J. Geo Inf. Sci.* **2019**, *21*, 1207–1217.
53. Yang, X.; Qiu, X.; Fang, Y.; Xu, Y.; Zhu, F. Spatial variation of the relationship between transport accessibility and the level of economic development in Qinghai–Tibet Plateau, China. *J. Mt. Sci.* **2019**, *16*, 1883–1900.
54. Zhu, Y.; Guang, G.; Lan, C.; Gao, J.; Pang, L. GIS-based analysis of traffic routes and regional division of the Qinghai–Tibetan Plateau in prehistoric period. *Prog. Geogr.* **2018**, *37*, 438–449.
55. Foggin, J.M. Environmental Conservation in the Tibetan Plateau Region: Lessons for China's Belt and Road Initiative in the Mountains of Central Asia. *Land* **2018**, *7*, 52–86.
