# Supplementary material for: Estimation of Undetected Asymptomatic COVID-19 Cases in South Korea Using a Probabilistic Model
Source: Int J Environ Res Public Health. 2021 May 6;18(9):4946. doi: 10.3390/ijerph18094946 (PMC8124955; doi:10.3390/ijerph18094946)
Supplement: Supplementary file 1 [file ijerph-18-04946-s001.zip › 5/ijgi-1151779.pdf]

ISPRS International Journal of Geo-Information

MDPI

Article

A Progressive and Combined Building Simplification Approach with Local Structure Classification and Backtracking Strategy

Zhiwei Wei [1,2,*], Yang Liu [3], Lu Cheng [3] and Su Ding [4]

1 The Aerospace Information Research Institute, Chinese Academy of Sciences, Beijing 100190, China
2 Key Laboratory of Network Information System Technology (NIST), Aerospace Information Research Institute, Chinese Academy of Sciences, Beijing 100190, China
3 School of Resource and Environment Science, Wuhan University, Wuhan 430079, China; 2015202050050@whu.edu.cn (Y.L.); 2018102050005@whu.edu.cn (L.C.)
4 College of Environmental and Resource Science, Zhejiang University, Zhejiang 311300, China; suding@zafu.edu.cn
* Correspondence: 2011301130108@whu.edu.cn

Abstract: Several algorithms have been developed to simplify buildings based on their local structure in past decades. However, different local structures are defined for certain purposes, and no algorithm can appropriately simplify all buildings. We propose a combined building simplification approach based on local structure classification and backtracking strategy. In this approach, local structures are classified and their based operations are defined by considering the buildings' orthogonal and non-orthogonal features. Each building is simplified to target scale with a selected local-structure-based operation progressively scale-by-scale. Rules are built to support the selection of local-structure-based operations with a binary decision tree, and a backtracking strategy is used when an invalid operation is applied. When the building is too small or the evaluation shows that it cannot be simplified based on local structures, template matching or enlargement algorithms are applied to simplify the building. A dataset (1:10k) collected from the Ordnance Survey was used for the experiment and simplified scale of 1:25k. Results satisfied legibility constraints and the change in area, orientation and position of simplified buildings are controlled within certain range by comparing with the results generated based on other four simplification algorithms. Possible use of our approach for continuous scale transformation of buildings is also discussed.

Keywords: map generalization; continuous scale representation; building simplification; buildings; progressive operation

Citation: Wei, Z.; Liu, Y.; Cheng, L.; Ding, S. A Progressive and Combined Building Simplification Approach with Local Structure Classification and Backtracking Strategy. *ISPRS Int. J. Geo-Inf.* **2021**, *10*, 302. https://doi.org/10.3390/ijgi10050302

Academic Editor: Wolfgang Kainz

Received: 4 March 2021
Accepted: 2 May 2021
Published: 5 May 2021

Publisher's Note: MDPI stays neutral with regard to jurisdictional claims in published maps and institutional affiliations.

Copyright: © 2021 by the authors. Licensee MDPI, Basel, Switzerland. This article is an open access article distributed under the terms and conditions of the Creative Commons Attribution (CC BY) license (http://creativecommons.org/licenses/by/4.0/).

1. Introduction

Simplification is an important operation in map generalization to decrease the complexity of geometric features after scale reduction [1,2]. As important man-made features on topographic maps, buildings have become the subject of intense research [3]. Many building simplification algorithms have been proposed in the past several decades. Building simplification is usually conducted on a single building, largely independent of contextual information. Furthermore, it sometimes can be conducted separately in a process of building map generalization.

Building simplification aims to represent buildings more concisely depending on the map scale or theme, with requirements of legibility and good representation of reality [4]. It can be implemented by replacing original buildings with a simpler form while preserving or even enhancing their main characteristics. For example, newly generated templates

(e.g., alphabetic shapes such as E, H, etc.) are used as simpler forms to represent simplified buildings at smaller scale [5,6]. If a building has a small area, it can also be replaced by a predefined symbol (e.g., a rectangle) or so-called building enlargement (*BE*) [7]. However, characteristics of buildings can differ across scales and regions, which may be inflexible to defining suitable templates for all buildings in template-based simplification (*TS*) algorithms. Another commonly used idea for building simplification is to remove the building's unimportant details while preserving important ones step-by-step or scale-by-scale. The details mainly refer to a building's local structures and many local-structure-based simplification (*LS*) algorithms have been developed, such as algorithms in which short edges, small corners, small bends, or small concaves are removed step-by-step or scale-by-scale to achieve building simplification [8–12]. With the development of Web-mapping, map services are required to support interactive zooming in and out to arbitrary scales. New methods such as continuous generalization and on-the-fly generalization have emerged. They can be achieved by applying generalization operations to generate coarser level of details from a given detailed dataset, on which *LS* algorithms can also be applied [13–15].

However, two main problems can arise in *LS* algorithms. First, different local structures are designed for certain purposes. For example, some algorithms assume buildings are designed with orthogonal features, wherein the buildings can be simplified based on square angles, concave structures, etc. [8–10]. However, buildings in the real world not only have orthogonal features, but some also have non-orthogonal features [16]. Thus, it may not be suitable for them to be simplified based on only one kind of defined local structure. Available local structures need to defined based on building characteristic in a *LS* algorithm, and the structure to be applied depends on generalization requirements and user demands. Second, *LS* algorithms are designed to remove a building's unimportant details, which can sometimes fail to appropriately simplify all buildings in a dataset. For example, a building may be too small to be simplified based on the local structures, and a *BE* algorithm may need to be applied in this situation [7]. Thus, other simplification algorithms need to be combined in case a building cannot be simplified based on the local structures. This idea, which combines different algorithms to achieve the building simplification task, has also been adopted in the Simplify Building Tool of ArcGIS. It combines rectangularization, squaring, regular pattern processing, and elimination of short edges in irregular shapes [17]. Steiniger et al. [18] utilized machine learning to learn control rules in an agent model for an agent with operations such as rectangularization, squaring, and scaling to achieve building simplification. Many scholars have also proven that it is effective to achieve map generalization combining several generalization algorithms [19–23].

Hence, we propose a progressive and combined building simplification approach considering cartographic requirements based on local structure classification and backtracking strategy. Local structures in building simplification are classified and their based operations are defined with consideration of each building's characteristics. Then the building is progressively simplified based on a selected local-structure-based operation, in which the application of this operation is supported by defined rules based on cartographic requirements. When a satisfactory result is not obtained with the selected local structure operation, a backtracking strategy is used. If the evaluation of the building shows that it cannot be simplified based on local structures, a *TS* algorithm is performed. If the building is too small to be simplified, a *BE* algorithm is applied.

2. Related Works

2.1. Building Simplification Constraints

Maps at a target scale need to meet the generalization requirements derived from map specifications or user demands. These requirements can be defined as constraints, and many constraints have been developed for different purposes. Two main kinds of

constraint can be summarized for building simplification: legibility and preservation (Figure 1) [4]. Legibility constraints ensure that the map is readable for human observers, while preservation constraints ensure a suitable representation of reality from the source map.

Legibility constraints

Minimum size (C1): The building size needs to be large enough to be interpretable, e.g., 0.5 mm × 0.5 mm at medium scale [24]. It can sometimes be converted to the minimum length and width of a building's bounding rectangle, e.g., 0.7 mm and 0.5 mm at scales from 1:25k to 1:50k [7].

Granularity (C2): Building edges needs to be large enough to avoid visual confusion, e.g., 0.3 mm [25].

Preservation constraints

Data correctness (C3): No data error is allowed, e.g., self-intersection [26].

Shape (C4): The building shape needs to be maintained, e.g., orthogonal features [27].

Size (C5): The building area needs to be maintained [25].

Orientation (C6): The main orientation of the building needs to be maintained [28].

Position (C7): The simplified building needs to be close to its original position [29].

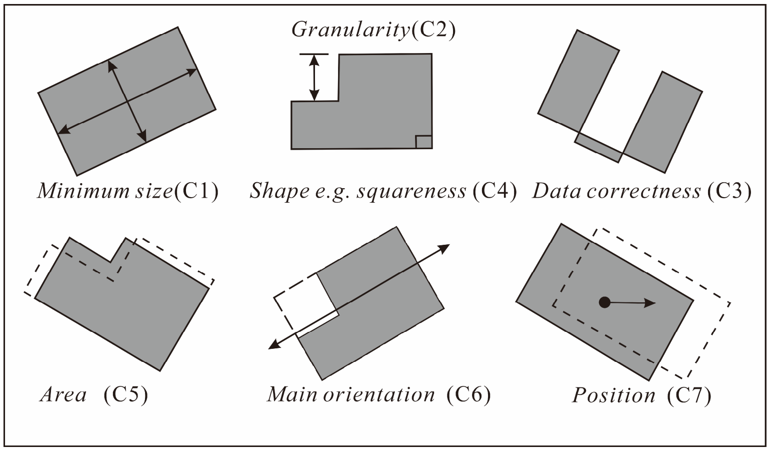

Figure 1. Constraints for building simplification.

Cartographic constraints in practice may have conflicting requirements and need to be optimized [30]. Buildings will inevitably violate legibility constraints with a reduction in scale. Attempts to resolve legibility constraints are bound to violate preservation constraints. Thus, legibility constraints must be satisfied for simplified buildings, while preservation constraints need to be satisfied as much as possible. This means legibility constraints have higher priority than preservation constraints. Furthermore, building data need to be correct before and after simplification. This means data correctness also has higher priority and must be satisfied.

2.2. Building Simplification Approaches

Spatial data can be represented as vector or raster models, and building simplification is also performed based on these two kinds of spatial data models.

Vector-based building simplification approaches can be classified into template-based and local-structure-based, which operate separately on the building itself or its local structures. In *TS* approaches, buildings are simplified by replacing them with a simpler form, e.g., predefined templates. These approaches have two concerns: defining suitable templates and matching buildings with the right one. To define suitable templates, Rainsford et al. [5] used alphabetic shapes such as E and H to represent rural buildings. Yan et al. [6] generated new templates by analyzing the characteristics of the regional environment. Wang et al. [7] used rectangles to enlarge buildings with small area. However, as

the characteristics of buildings vary across areas and scales, it can sometimes be unfeasible to use the limited types of templates to represent all buildings. Matching buildings with the right templates is performed mainly by matching candidates with a similar shape. For example, Ai et al. [3] matched templates by measuring shape similarity based on Fourier transform, while Yan et al. [6] measured shape similarity based on turning angle. However, the shape is just one characteristic of a building, and other characteristics may also be of interest. Furthermore, representing buildings by limited types of simpler templates may lead to oversimplification for some complex buildings.

In *LS* approaches, buildings are simplified by removing unimportant details while preserving important ones. The details mainly refer to the buildings' local structures. For example, Guo [8] took the square angle as a basic unit to progressively simplify buildings, while Chen et al. [10] filled concave structures detected based on constrained Delaunay triangulation step-by-step. Xu et al. [26] took the four adjacent points to define the bend structures as a basic unit and progressively removed the short edges. Sester [9] defined offset, extrusion or intrusion, and corner as the basic units in building simplification, then applied least-squares adjustment to obtain the simplified result. The least squares method was also used by Bayer [31], who designed recursive approaches based on the Douglas–Peucker algorithm. In these *LS* approaches, different local structures are defined for certain purposes, which may not be suitable for simplifying varied buildings. For example, concave structures defined by Chen et al. [10] assume buildings are designed with orthogonal features. Hence, it may not be suitable to simplify buildings with non-orthogonal features.

In raster-based approaches, morphological strategies are mainly used. For example, Damen et al. [32] aggregated and simplified buildings based on morphological operators such as erosion and dilation. Meijers [33] used the same morphological operators based on offset curves generated from a straight skeleton, in which the square angle corners can be better preserved. Kada [34] used dilation and erosion to simplify buildings based on cell decomposition and half-space modeling. With the development of artificial intelligence, Cheng et al. [11] presented a novel method for building simplification using a back-propagation neural network (BPNN) model. Shen et al. [16] simplified buildings based on super-pixel segmentation, which can be applied to buildings with orthogonal or non-orthogonal features. However, because vector data are the focus of this paper, it is not intuitive to use these raster-based algorithms. Some additional transformations are necessary if we apply them to vector data.

3. Methodology

3.1. Framework

Representation (RB^t_i) of building B_i at target scale (1: M_t) is obtained by simplifying its representation (RB^0_i) at original scale (1:M_0) scale-by-scale with our approach: RB^0_i will violate legibility constraints (minimum size and granularity) at the next possible scale (1:M_1) as the scale reduces gradually. A simplification operation is triggered to obtain a new representation (RB^1_i) based on RB^0_i; if $M_0 > M_1$, RB^1_i can be considered as a representation of B_i from scale M_0 to M_1. The simplification operation is implemented on RB^j_i repeatedly until RB^{j+1}_i at the target scale is obtained. The framework to simplify B_i to target scale is designed as follows (Figure 2).

Step 1. Perform preprocessing to remove potential abnormal nodes of buildings before applying any simplification operation (Section 3.2.)

Step 2. Violation detection at the next possible scale. Violations are defined based on whether the legibility constraints are violated at the next possible scale (Section 3.3).

Step 3. If RB^j_i violates granularity constraints at the next possible scale, perform a local-structure-based simplification (*LS*) algorithm (Section 3.4). This algorithm classifies local structures and defines their based operations (Section 3.4.1). Rules considering preservation constraints supporting the application of local-structure-based operation at

the current step are provided (Section 3.4.2). A backtracking searching strategy is also provided in case an invalid local-structure-based operation is applied. If a satisfactory result can be obtained with *LS* algorithm, return to Step 1; otherwise, proceed to Step 4. Whether the result is a satisfactory one is defined based on preservation constraints (Section 3.4.3).

Step 4. If the *LS* algorithm cannot obtain a satisfactory result, use a template-based simplification (*TS*) algorithm; then return to Step 1 (Section 3.5).

Step 5. If the minimum size constraint will be violated at the next possible scale, use a building enlargement (*BE*) algorithm (Section 3.5).

In all of the above steps, the simplification process is stopped while obtaining a simplified building at target scale.

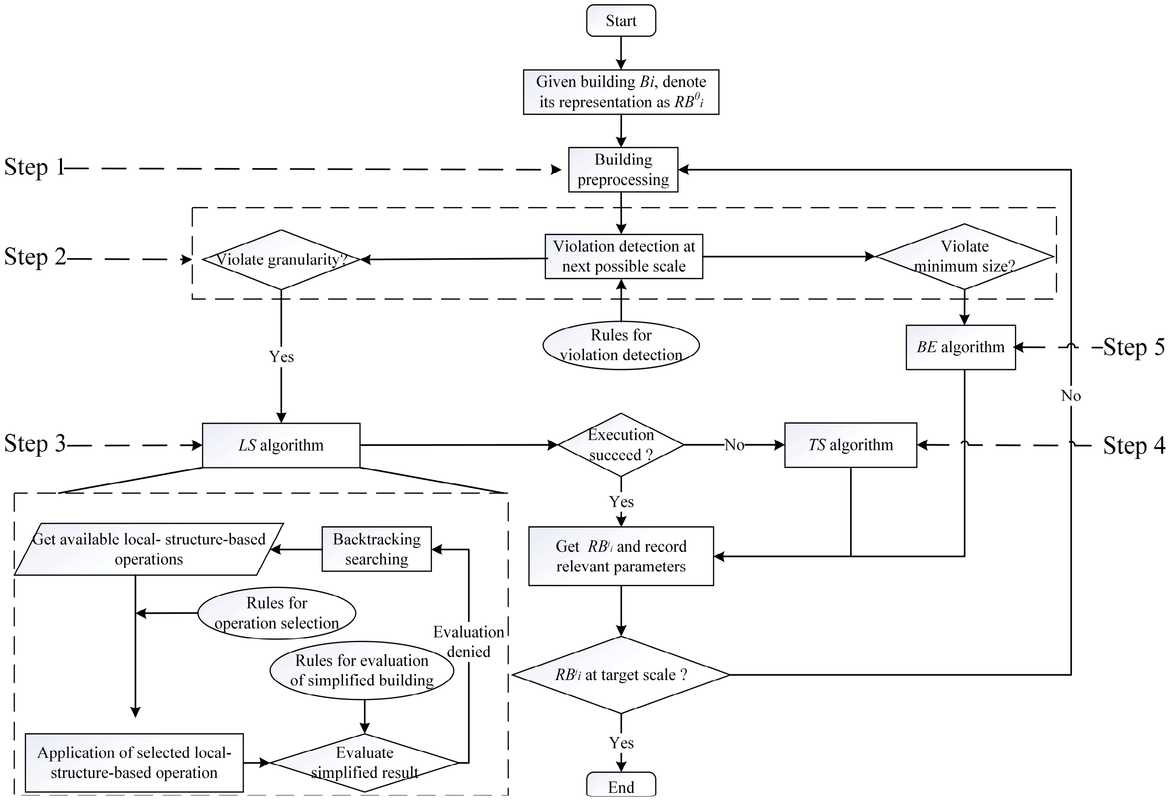

Figure 2. Framework of our approach.

3.2. Step 1：Building Preprocessing

Preprocessing is performed before application of any simplification operation for possible data error, e.g., abnormal nodes such as repeating nodes, collinear nodes, and sharp nodes [8]. Suppose node set of a building polygon as $AP = \{p_1, p_2, ..., p_n\}$, in which $p_1 \neq p_n$. The angle at node p_m is supposed as C^m_{angle}, denoted as the angle of edge $p_{m-1}p_m$ rotates counterclockwise around node p_m to edge $p_m p_{m+1}$, and $C_{angle} \in [0°, 360°]$. Thus, repeating nodes, collinear nodes, and sharp nodes are defined as followed.

Repeating nodes: Suppose distance between nodes p_m and p_n in *AP* as Dis_{mn}, if $Dis_{mn} < Td$, then p_m and p_n are repeating nodes, e.g., nodes *G* and *H* in Figure 3(a). *Td* is a threshold and $Td = 0.01\text{mm}$.

Collinear nodes: Suppose angle at node p_m in *AP* as C^m_{angle}, if $|C^m{}_{angle} - 180°| < \delta$, then p_m is a collinear node, e.g., node *B* in Figure 3a. δ is a threshold and $\delta = 5°$.

Sharp nodes: Suppose angle at node p_m in *AP* as C^m_{angle}, if $C^m{}_{angle} < \delta \vee (360 - C^m{}_{angle}) < \delta$, then p_m is a sharp node, e.g., node *E* in Figure 3a. δ is a

threshold and $\delta=5°$.

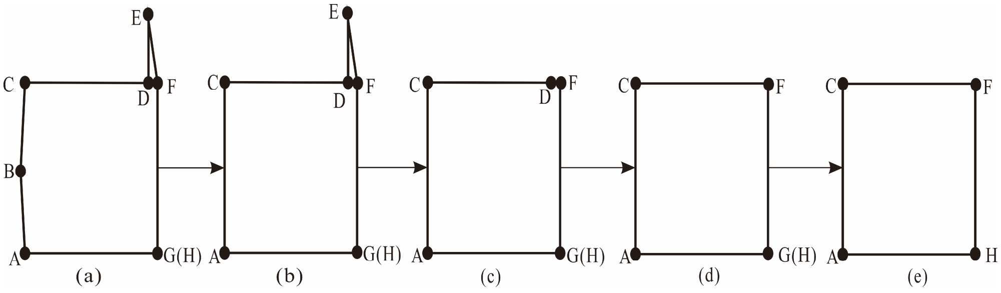

Figure 3. Preprocessing for buildings. (**a**) 0riginal polygon; (**b**–**e**) Preprocessing for buildings to remove abnormal nodes.

Preprocessing means delete repeating, collinear, and sharp nodes in the node set $AP=\{p_1, p_2,..., p_n\}$ progressively. For example, buildings in Figure 3b to Figure 3e are obtained by deleting collinear node *B*, sharp node *E*, collinear node *D*, repeating node *G* from the building in Figure 3a progressively.

3.3. Step 2：Definitions of Legibility Constraint Violations

Simplification operation is triggered for buildings violate different legibility constraints with a reduction in scale. Thus, the parameters describing legibility constraints can be used to determine at which scale these constraints are violated.

Two kinds of legibility constraints are defined in Section 2.1: minimum size and granularity. The minimum size constraint can be ruled by a building's minimum area (ST_a) and sometimes can be converted into the minimum length (ST_L) and width (ST_W) of its bounding rectangle [7]. The granularity constraint rules the minimum length (GT_L) of building edges. Take a representation of building B_i at scale 1: M_j as $B^j{}_i$, its area as $AreaB^j{}_i$, the length and width of its minimum bounding rectangle (MBR) as $LB^j{}_i$ and $WB^j{}_i$, and the length of its shortest edge as $MLe^j{}_i$. The next possible scales (1: $M^1{}_{j+1}$ to 1: $M^4{}_{j+1}$) considering $AreaB^j{}_i$, $LB^j{}_i$, $WB^j{}_{i,}$, and $MLe^j{}_i$ of $B^j{}_i$ separately can be computed as Equations (1)–(4):

$$M^1{}_{j+1} = \sqrt{AreaB^j{}_i / STa} * 1000 \quad (1)$$

$$M^2{}_{j+1} = LB^j{}_i / ST_L * 1000 \quad (2)$$

$$M^3{}_{j+1} = WB^j{}_i / ST_W * 1000 \quad (3)$$

$$M^4{}_{j+1} = MLe^j{}_i / GT_L * 1000 \quad (4)$$

A building needs to be simplified if a violation of any kind of legibility constraint is detected with a reduction in scale. Thus, the next possible scale (1: M_{j+1}) is denoted as $M_{j+1}=min\{M^1{}_{j+1}, M^2{}_{j+1}, M^3{}_{j+1}, M^4{}_{j+1},\}$. The violations of legibility constraints for a representation of building (B_{ji}) can be defined as shown in Table 1. Furthermore, they can be developed into rules in an if–then form in violation detection for buildings at the next possible scale [35]. For example, if $M^1{}_{j+1}=min\{M^1{}_{j+1}, M^2{}_{j+1}, M^3{}_{j+1}, M^4{}_{j+1},\}$, then the building is detected as violation on *STa* when scale is reduced to exceed $M^1{}_{j+1}$.

Table 1. Definitions of legibility constraint violations.

Description	Constraint violation
$M^1{}_{j+1}=min\{M^1{}_{j+1}, M^2{}_{j+1}, M^3{}_{j+1}, M^4{}_{j+1},\}$	Minimum size (violates ST_a)
$M^2{}_{j+1}=min\{M^1{}_{j+1}, M^2{}_{j+1}, M^3{}_{j+1}, M^4{}_{j+1}\}$	Minimum size (violates ST_L)

$M^3_{j+1} = min\{M^1_{j+1}, M^2_{j+1}, M^3_{j+1}, M^4_{j+1},\}$	Minimum size (violates ST_W)
$M^4_{j+1} = min\{M^1_{j+1}, M^2_{j+1}, M^3_{j+1}, M^4_{j+1},\}$	Granularity (violates GT_L)

3.4. Step 3：Local-Structure-Based Simplification Algorithm

In our approach, buildings are simplified based on their local structures if they violate the granularity constraint at the next possible scale. The granularity constraint rules the minimum length of building edges. Thus, the *LS* algorithm aims to eliminate the shortest edge of the building at the current step. The local structures in which the shortest edge may located are classified first, and their based operations to eliminate the shortest edge are then defined (Section 3.4.1). As the shortest edge may be located in various local structures, different local-structure-based operations can be used at the current step. A rule-driven selection of applied local-structure-based operation is provided (Section 3.4.2). Whether the operation can obtain a satisfactory result needs to be evaluated. A backtracking strategy is applied in case an unsatisfactory result is obtained (Section 3.4.3).

3.4.1. Local Structure Classification and Their Based Operations

(1) Local structure classification

A building polygon can be represented by a node set as $AP = \{p_1, p_2, ..., p_n\}$, in which $p_i \neq p_n$; or an edge set as $AE = \{e(p_i, p_j) \mid p_i, p_j \in AP\}$. As each node connects two edges in the building polygon, it can also be represented by a set of defined bends denoted as a node set $\{p_{m-1}, p_m, p_{m+1}\}$ or an edge set $\{e(p_{m-1}, p_m), e(p_{m-1}, p_{m+1})\}$, $p_{m-1}, p_m, p_{m+1} \in AP$ [36]. Other kinds of bends can also be defined in some complex polygons, e.g., water area boundaries [37]. However, as buildings are man-made, their polygonal features tend to have simple shapes and orthogonal features [26]. In our approach, we adopted the bend definition according to Fan et al. [36], which is also a basic type of bend. The angle of a bend (C_m) is considered as C^m_{angle} at node p_m, denoted as the angle of edge $p_{m-1}p_m$ rotated counterclockwise around node p_m to edge p_mp_{m+1}. According to bend angle, a bend can be defined as convex with $C^m_{angle} \in [0°, 180°]$ or concave with $C^m_{angle} \in [180°, 360°]$. An orthogonal feature in a building refers to two edges (e_m, e_n) in the building polygon being orthogonal, which means angle (E_{angle}) of edges e_m and e_n meets the condition $|E_{angle} - 90°| < \alpha$, where $E_{angle} \in [0°, 180°)$ and α is a threshold. According to whether an orthogonal feature is formed for two edges in a defined bend, the bend can also be defined as orthogonal or non-orthogonal.

If orthogonal features in buildings, e.g., natural polygons such as water area boundaries, are not considered. They are usually understood as being composed of different types of bends and simplified based on those bends [37]. Thus, buildings can be simplified based on defined bends if orthogonal features are not considered. If orthogonal features are considered, some *LS* algorithms have been proposed. For example, Sester [9] simplified buildings based on offset, intrusion (or extrusion), and corners. Chen et al. [10] simplified buildings based on concave parts. Xu et al. [26] took four adjacent nodes as basic units and progressively removed the short edges. In these algorithms, the local structures are all considered as a composition of two consecutively defined bends that may contain an orthogonal feature. For example, a concave part defined by Chen et al. [10] can be considered as a composition of two concave orthogonal bends, while the offset defined by Sester [9] can be considered as a composition of convex and concave orthogonal bends. Thus, if orthogonal features in buildings are considered, local structures can be defined based on two consecutive defined bends in which at least one orthogonal feature is contained. Thus, in our approach local structures are defined as follows:

Bend (Type 1): Represented as a node set $\{p_{m-1}, p_m, p_{m+1}\}$ or an edge set $\{e(p_{m-1}, p_m), e(p_{m-1}, p_{m+1})\}$, in which $p_{m-1}, p_m, p_{m-1} \in AP$, e.g., bends *ABC* and *CDE* in Figure 4(b).

Concave or convex (Type 2): Consists of two bends defined as convex or concave, and at least one is an orthogonal bend, e.g., concave *KABC* and convex *BCDE* in Figure 4c.

Offset (Type 3): Consists of two defined bends, one convex and one concave, and at least one is an orthogonal bend, e.g., offsets *AKJI* and *CDEF* in Figure 4d.

Corner (Type 4): Consists of two defined non-orthogonal bends, represented as an edge set $\{e_{m-1}, e_m, e_{m+1}\}$, in which e_{m-1} and e_{m+1} form an orthogonal feature, e.g., corner *FGHI* in Figure 4e.

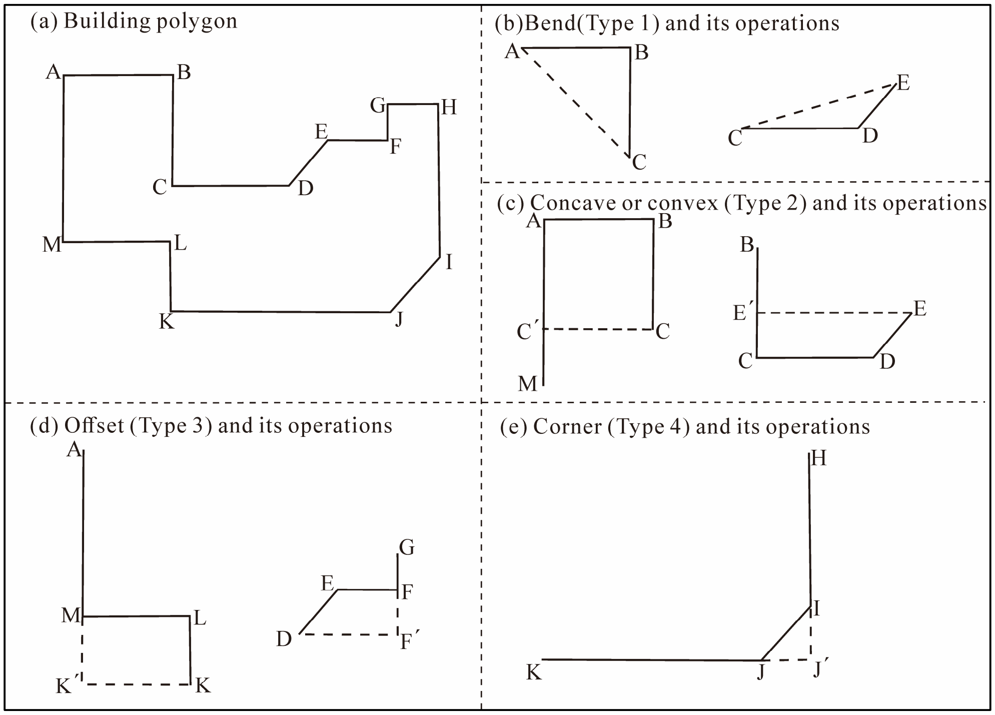

Figure 4. Local structure classifications and their basic operations (denoted by the dashed lines). (**a**) Building polygon; (**b**) bends; (**c**) concave and convex bends; (**d**) offsets; (**e**) corner.

(2) Local-structure-based operations to eliminate the shortest edge

Operations differ for the shortest edge may locate in different defined local structures. Given the shortest edge $e(p_i, p_j)$ in a local structure (*S*), operations to eliminate it based on the type of *S* are defined as follows:

Operation 1: If *S* is Type 1, which means $e(p_i, p_j)$ is in a bend, denoted as node set $\{p_{m-1}, p_m, p_{m+1}\}$. The operation to eliminate $e(p_i, p_j)$ is to connect nodes p_{m-1} and p_{m+1}. For example, as shown in Figure 4(b), to eliminate the shortest edge *AB* in bend *ABC*, the operation is to connect nodes *A* and *C*.

Operation 2: If *S* is Type 2, which means $e(p_i, p_j)$ is in a concave or convex bend and is denoted as node set $SP = \{p_1, p_2, p_3, p_4\}$. The operation to eliminate $e(p_i, p_j)$ is to fill the concave or convex. Draw a line parallel to edge $e(p_2, p_3)$ from node p_1 to edge $e(p_3, p_4)$. If an intersected node p_m is obtained, the node set of the processed *S* is represented as $SP = \{p_1, p_m, p_4\}$; otherwise, draw a line parallel to edge $e(p_2, p_3)$ from node p_4 to edge $e(p_1, p_2)$ to obtain an intersected node (p_n), then the node set of the processed *S* is represented as $SP = \{p_4, p_n, p_1\}$. For example, as shown in Figure 4(c), to eliminate the shortest edge *DE* in the defined concave *BCDE*, the operation is to draw a line parallel to edge *CD* from node *E* and intersect edge *BC* as a node E', and the node set of the processed *S* is represented as $SP = \{B, E', E\}$.

Operation 3: If the local structure (S) is Type 3, which means $e(p_i, p_j)$ is in an offset, denoted as node set $SP = \{p_1, p_2, p_3, p_4\}$ with two bends $B_1 = \{p_1, p_2, p_3\}$ and $B_2 = \{p_2, p_3, p_4\}$. The operation to eliminate $e(p_i, p_j)$ is to fill the orthogonal part of the offset. If B_1 is an orthogonal bend, then draw a line parallel to edge $e(p_2, p_3)$ from node p_4 to intersect extending edge $e(p_1, p_2)$ as node p_m, and the node set of the processed local structure (S) is represented as $SP = \{p_4, p_m, p_1\}$. Otherwise, if B_2 is an orthogonal bend, then draw a line parallel to edge $e(p_2, p_3)$ from node p_1 to intersect extending edge $e(p_3, p_4)$ as node p_n, and the node set of the processed local structure (S) is represented as $SP = \{p_1, p_n, p_4\}$. B_1 and B_2 may both be orthogonal bends, and two operations are available based on the above definitions. In this case, the concave orthogonal bend is filled. For example, as shown in Figure 4(d), supposing the shortest edge in the defined offset *DEFG* is edge *FG*, the operation is to draw a line parallel to edge *EF* from node *D* and intersect extending edge *FG* as node F', and the node set of the processed local structure (S) is represented as $SP = \{D, F', G\}$.

Operation 4: If S is Type 4, which means $e(p_i, p_j)$ is in a corner, denoted as node set $SP = \{p_1, p_2, p_3, p_4\}$. The operation to eliminate $e(p_i, p_j)$ is to fill the corner. Extend $e(p_1, p_2)$ and $e(p_3, p_4)$ to get an intersected node p_m, and the node set of the processed S is represented as $SP = \{p_1, p_m, p_4\}$. For example, as shown in Figure 4(e), to eliminate the shortest edge *IJ* in the defined corner *HIJK*, the operation is to extend edges *KJ* and *HI*, then intersect as a node J', and the node set of the processed S is represented as $SP = \{K, J', H\}$.

3.4.2. Selection of Applied Local-Structure-Based Operation

To eliminate the shortest edge $e(p_i, p_j)$ at the current step, available operations can be obtained based on local structures in which it is located, denoted as $Q = \{q_1, ..., q_n\}$, where q_n represents an operation that can eliminate $e(p_i, p_j)$. Applying a different q_n in Q can generate different results. The best operation to apply can be selected based on evaluating the results after applying each q_n in Q.

(1) Data structure for each local-structure-based operation

The application of operation q_n in Q will lead to different results, specifically changes in the characteristics of a building, e.g., damage to orthogonal features, or changes in the area or the main orientation. These changes can be recorded as the properties of each operation q_n in Q. For example, in Figure 5a, two operations, denoted as (1) and (2), are applied to eliminate the shortest edge, *CD*. Applying Operation (1) will lead to a self-intersection in the simplified building, while applying Operation (2) will not. Similarly, in Figure 5b, two operations are applied to eliminate the shortest edge, *BC*. Applying Operation (1) will damage an orthogonal feature, while applying Operation 2 will not. Furthermore, a new simplified building can be obtained after applying any operation. The simplified building may have a different area, main orientation, and position compared to the previous one. For example, two operations are applied to eliminate the shortest edge, *BC*, in Figure 5c. Applying Operation (1) will lead to an increase in area, while applying Operation (2) will lead to a decrease in area.

Changes in the characteristics of buildings after applying local-structure-based operation q_n can be recorded as a data structure, as shown in Table 2.

Table 2. Data structure to record changes in building characteristics after applying q_n.

Field	Type	Description
ID	Int	Unique building identification code.
SType	Int	Denotes type of local structure: 1 = bend, 2 = convex or concave, 3 = offset, 4 = corner.
IsInt	Bool	Denotes whether self-intersection is generated after applying q_n: true or false.
SDame	Bool	Denotes whether orthogonal features are damaged after applying q_n: true or false.
AreaC	Double	Denotes area change rate by comparing to original after applying q_n, as $AreaC = \lvert AreaA - AreaS \rvert / AreaA$, in which *AreaA* is the area of original one, *AreaS* is the area of the obtained building after applying q_n.
OriC	Double	Denotes main orientation change compared to original after applying q_n, as $OriC = \begin{cases} \lvert OriO - OriS \rvert & (\lvert OriO - OriS \rvert \le 90) \\ 180 - \lvert Ori - OriS \rvert & (\lvert OriO - OriS \rvert > 90) \end{cases}$, in which *OriC* is the MBR orientation of the original one, *OriS* is the MBR orientation of the obtained building after applying q_n.
PosC	Double	Denotes displaced distance by comparing to original after applying q_n.

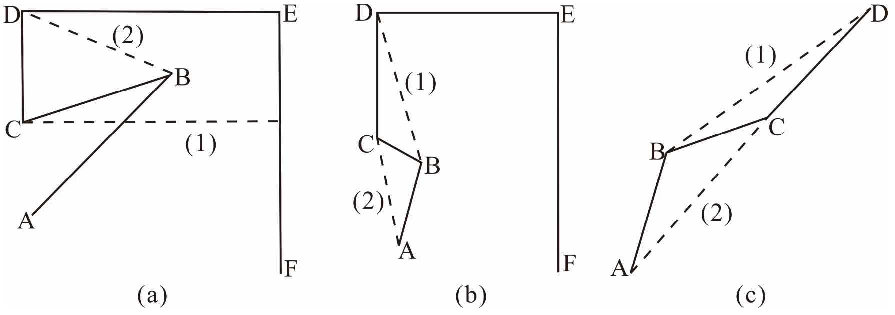

Figure 5. Changes in building characteristics after applying local-structure-based operation: (**a**) generate self-intersection; (**b**) damage orthogonal features. (**a–c**) Operations may lead to changes in area, main orientation, and position of a building.

(2) Selection of local-structure-based operation based on binary decision tree

The result obtained after applying a local-structure-based operation to eliminate the shortest edge can be evaluated according to cartographic constraints. Because the operation is triggered for legibility constraint violations, the preservation constraints are the main concern of our evaluation. Data correctness is a constraint that must be fulfilled. If the applying operation q_n in *Q* leads to self-intersection of the simplified building, i.e., property *IsInt* of q_n is true, then q_n needs to be deleted from *Q*. Thus, the constraints of concern are preservation of shape, size, main orientation, and position. The best local-structure-based operation is selected according to the evaluation.

The constraints may conflict with each other, and two parameters for constraints can be defined when evaluating simplified results: importance and priority (Steiniger et al., 2010). Our approach adopts constraint priority. It rules which constraint should be considered first and can be represented as a binary decision tree, Figure 6 [38]. In this tree, each node represents a kind of constraint, and the logical expression that expresses the constraint is defined in the node. For example, given two operations q_1 and q_2, the logical expression that expresses the shape preservation constraint can be defined as $q_1.SDame = q_2.SDame$? If true for the expression, the two operations both satisfy or violate the shape preservation constraint; if not, the operation with false *SDame* is considered the better one. The depth of the node represents the priority of its corresponding constraint,

and greater depth means lower priority. The priority of preservation constraints is optional parameters that can be set by users in our approach. Default priorities are also set in our approach. For the building in our approach, the shape-preserving constraint (maintaining orthogonal features) has higher priority. Further, the area-preserving constraint is considered next, then the main orientation. The position constraint is the last to be considered.

The selection process along the binary decision tree can be implemented with defined priorities and their logical expressions (Figure 6).

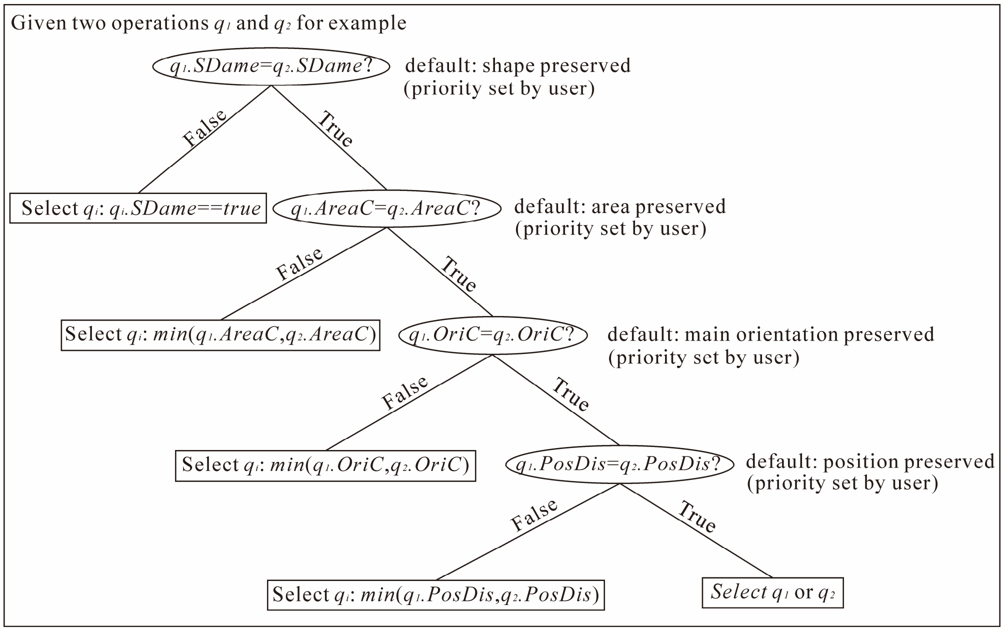

Figure 6. Local-structure-based operation selection process along binary decision tree.

3.4.3. Evaluation and Backtracking Strategy

Whether the simplified building is a reasonable result after applying the selected local-structure-based operation needs to be evaluated [39]. If an unsatisfactory result is obtained, a backtracking strategy is applied to obtain another possible satisfactory result.

(1) Evaluation

Although building characteristics are inevitably changed after simplification, these changes need to be limited to a certain range [8]. Thus, an evaluation is performed to determine whether changes in the area, main orientation, position, and shape with regard to the corresponding preservation constraints exceed thresholds. Suppose a selected operation q_n with properties *AreaC*, *OriC* and *PosC* (defined in Table 2) is applied. The rules for evaluation are defined as follows.

Rule 1: If $AreaC > T_a$, then the result obtained by applying q_n is evaluated as unsatisfactory.

Rule 2: If $OriC > T_o$, then the result obtained by applying q_n is evaluated as unsatisfactory.

Rule 3: If $PosC > T_p$, then the result obtained by applying q_n is evaluated as unsatisfactory.

Rule 4: If the building obtained by applying the selected operation p_n has fewer than four nodes, then it is evaluated as unsatisfactory.

T_a, T_o, T_p in above rules are thresholds and can be set according to the constraints illustrated in Section 2.1. For example, the position preservation constraint rules that the simplified building needs to be close to its original position, and the distance needs to be

limited, e.g., 0.5 mm [29]. Furthermore, *Tp* can be set as T_p=0.5mm. Thus, the result obtained by applying q_n violates any above rules, it is evaluated as an unsatisfactory one; otherwise, it is a satisfactory one.

(2) Backtracking strategy

Problem definition: Given a building B_i, the obtained representations of B_i with previous simplification operations are $RBS=\{RB^1{}_i, RB^2{}_i, \dots, RB^{k-1}{}_i\}$, and available operations for $RB^j{}_i$ in *RBS* are denoted as $Q_j=\{q^1{}_j, \dots, q^n{}_j\}$. Once $RB^k{}_i$ obtained from $RB^{k-1}{}_i$ based on operation $q^m{}_{k-1}$ selected in available operation set Q_{k-1} is evaluated as an unsatisfactory result, the backtracking strategy is used as follows: First, return to Q_{k-1} to select another $q^m{}_{k-1}$. Second, if all available operations in Q_{k-1} are evaluated as unable to obtain a satisfactory result, return to Q_{k-2} to select another operation, and so on.

However, increased backtracking depth may increase the possibility of making poor choices of operations to obtain satisfactory results, which may also lead to a huge searching space. Thus, a maximum searching step (*MaxS*) is set. Furthermore, the backtracking search needs to terminate when all available choices have been chosen. The backtracking strategy is performed as shown below.

Backtracking strategy.

Input: Obtained representations of B_i as $RBS=\{RB^1{}_i, RB^2{}_i \dots, RB^{k-1}{}_i\}, k \geq 1$, available operations for $RB^j{}_i$ in *RBS* as $Q_j=\{q^1{}_j, \dots, q^n{}_j\}$; maximum searching step as *MaxS*
Output: RB_k
Set searching step as *p*, and p=0
Get Q_j: Obtain available operation set Q_j based on $RB^j{}_i$ and start with j=k-1
When $Q_j \neq null$ AND p<$MaxS$ AND $j \geq 2$, Then
Selection: Select an operation $q^m{}_j$ in Q_j based on the binary decision tree defined in Section 3.4.2 to obtain $RB^k{}_i$, and whether $RB^k{}_i \in RBS$ needs to be determined.
If $RB^k{}_i \in RBS$, remove $q^m{}_j$ in Q_j, Continue
Else if $RB^k{}_i \notin RBS$, Then
p=p+1
Evaluation: Determine whether $RB^k{}_i$ satisfies rules defined for simplified building:
If evaluation accepted, Then return to $RB^k{}_i$, End.
Else if evaluation denied, remove $q^m{}_j$ in Q_j.
When Q_j=$null$ AND p<$MaxS$ AND $j \geq 2$, Then
j=j-1, return to Get Q_j
Else Return null, End.

3.5. Steps 4 and 5：Template-Based and Building Enlargement Simplification

Applying an *LS* algorithm may fail to yield a satisfactory result, which means that even if a backtracking strategy is applied, a satisfactory result will still not be obtained. A *TS* algorithm is then applied. And if a building violates the minimum size constraint, a *BE* algorithm is applied.

(1) Step 4: template-based simplification

A *TS* algorithm according to Yan et al. [6] is adopted in our approach. However, templates in his approach are formulated by analyzing the typical characteristics of the regional environment, which may be inflexible when defining suitable templates for all buildings. We modified that template definition for our approach.

Buildings are man-made features created according to a certain environment, and nearby buildings tend to have a similar shape [25]. Thus, other simplified buildings at target scale in the same region can also be used as available templates, which are defined as follows: the building to be simplified is denoted as $BS=\{B_1, \dots, B_m\}$, in which buildings

have successfully been simplified, and their representation at target scale is denoted as $BS'=\{B_n,...,B_{n+p}\}$, then the buildings in BS' are all considered as available templates. Shape similarity measurement based on turning function is adopted to select the best template [6]. The selected template needs to be scaled up for the area preservation constraint. If the scaled building violates legibility constraints at target scale, another template is selected on this occasion.

(2) Step 5: building enlargement simplification

A *BE* algorithm referring to Wang et al. [7] is adopted in our approach. The minimum size is ruled by the building area (ST_a) and sometimes can be converted into the minimum length (ST_L) and width (ST_W) of the building's bounding rectangle. Violations of the minimum size constraint for buildings at the next possible scale are defined in Section 3.3. Given a building B_i, the length and width of its bounding rectangle are denoted as LB_i and WB_i. It can be enlarged based on the type of violation according to Wang et al. [7].

Rule 1: If B_i violates ST_a at the next possible scale, it is enlarged by replacing it with a rectangle as ST_L*ST_W.

Rule 2: If B_i violates ST_W at the next possible scale, it is enlarged by replacing it with a rectangle as LB_i*ST_W.

Rule 3: If B_i violates ST_L at the next possible scale, it is enlarged by replacing it with a rectangle as WB_i*ST_L.

Rule 4: If no violation of minimum size constraint is found at the next possible scale, no building enlargement will be performed.

For example, the parameters for building enlargement at 1:25k can be set as $ST_a = 0.35mm^2$, $ST_L = 0.7mm$ and $ST_W = 0.5mm$ according to the Chinese National Administration of Surveying [40]. Buildings are enlarged based on defined rules (Figure 7).

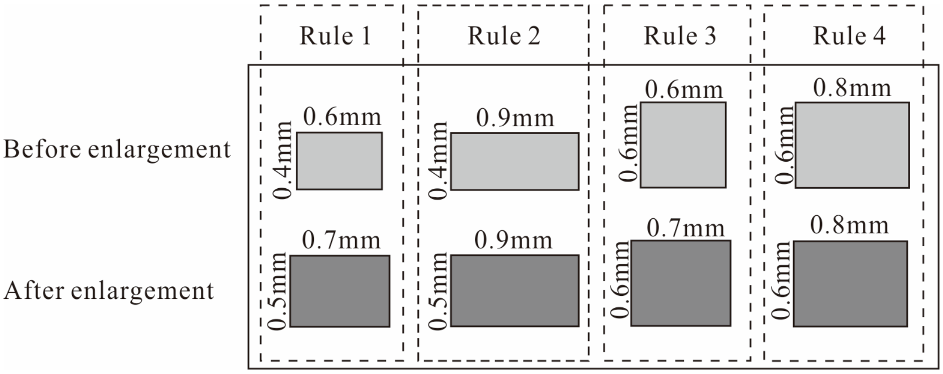

Figure 7. Building enlargement.

4. Experiment

Topographic building data with 122 buildings in different shapes collected from an open data product named OS Open Map Local and provided by the Ordnance Survey were used for our experiments (Figure 8). These are "street-level" map data with a nominal viewing scale of 1:10k [41]. Some basic geographic features, such as roads and vegetation, are also included in this study area. As we aim to simplify the buildings, and they are not shown in the figure, our approach was implemented on ArcEngine 10.2 (ESRI, USA) using C#. Data and code in our experiments are all available at: https://data.mendeley.com/datasets/tgngv7dkgn/3.

Parameters for legibility constraints based on Section 2.1 and defined in Section 3.3 were set as follows: $ST_a = 0.35mm^2, ST_L = 0.7mm, ST_W = 0.5mm, GT_L = 0.3mm$. Parameters for evaluation in the *LS* algorithm were set as follows: $AreaC \le 0.3, OriC \le 30^\circ, PosC \le 0.5mm$. Buildings in the dataset were simplified into scale of 1:25k (Figure 8). There is no selection, aggregation or typification in the experiments. The number of buildings simplified based

on different algorithms, as shown in Table 3. The data quality of simplified buildings at the target scales was evaluated according to the generalization constraints described in Section 2.1. All buildings at the target scales satisfied the set control parameters of legibility constraints. The preservation constraints were evaluated by comparing the changes in the area (*AreaC*), main orientation (*OriC*), and position (*PosC*) of the buildings between original and target scales, which are defined in Table 2 (Figure 9). Further, the change in shape is also evaluated by surface distance (*sDis*) according to Yan et al. [6] as in Equation (5) (Figure 9).

$$sDis = Area(B_o \cap B_s) / Area(B_o \cup B_s) \tag{5}$$

where $Area(B_o \cap B_s)$ is the area of intersection of buildings *Bo* and *Bs*, and $Area(B_o \cup B_s)$ is the area of union of buildings *Bo* and *Bs*. As buildings that violate the minimum size constraint are enlarged in our approach, this may lead to a large change in area and shape. Thus, changes in area and shape were not compared for buildings enlarged at the target scale. As shown in Figure 9, *AreaC* is almost controlled within 0.10, *OriC* within 5°, *PosC* within 0.2 mm, and s*Dis* within 0.8. The largest *AreaC* is 0.295, the largest *OriC* is 24.2°, and the largest *PosC* is 0.340 mm. All of these changes are controlled within set parameters ($AreaC \le 0.3, OriC \le 30^\circ, PosC \le 0.5mm$) for the *LS* algorithm. Further, *sDis* for several buildings after simplification is less than 0.6 in Figure 9 because they are simplified by *TS* algorithm in our approach.

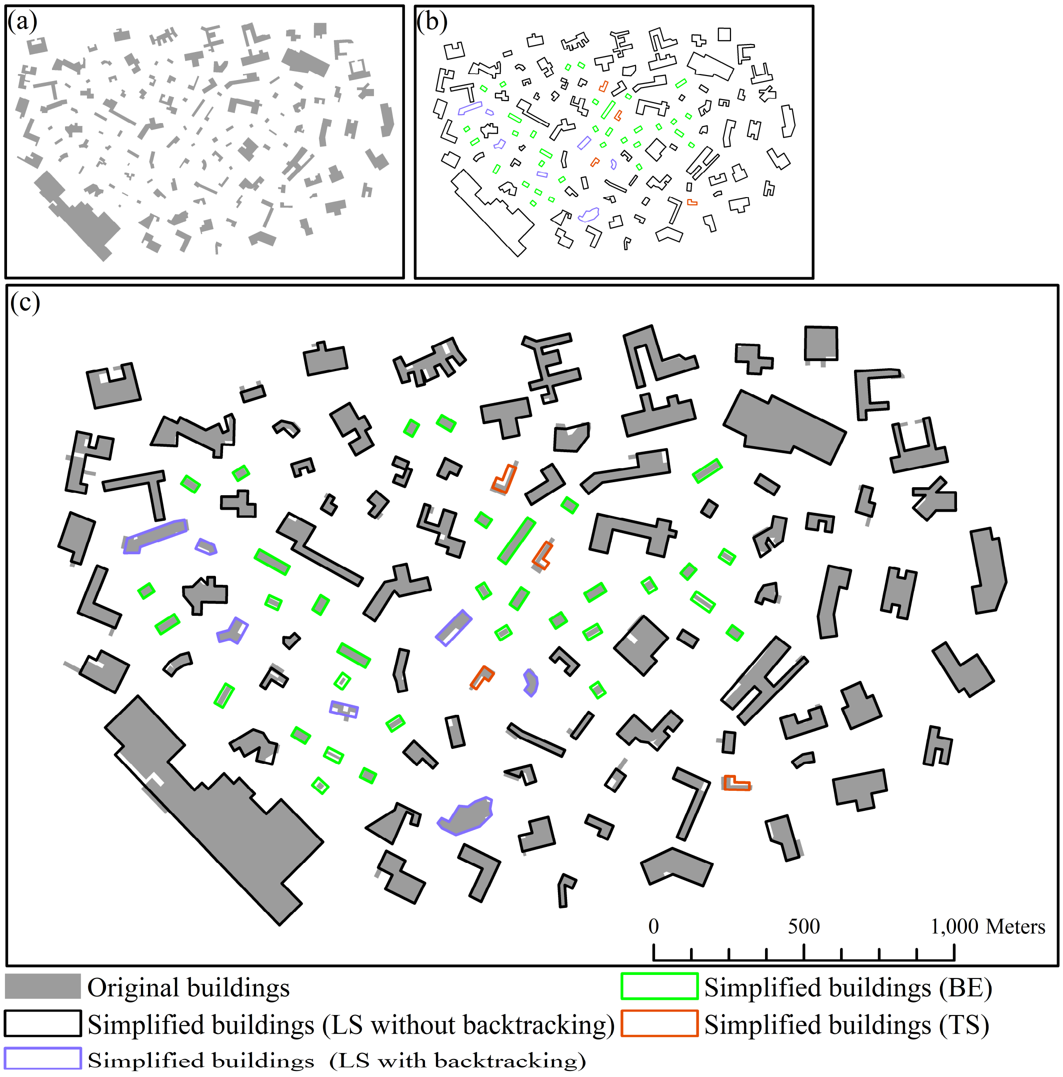

Figure 8. Simplified buildings at scale 1:25k: (**a**) original dataset; (**b**) simplified result; (**c**) comparison between the two. Result is scaled up for a clear view.

Table 3. Number of buildings simplified based on different algorithms.

Scale	Number of simplified buildings			
	LS without backtracking	*LS* without backtracking	*TS*	*BE*
1: 25k	78	7	4	33

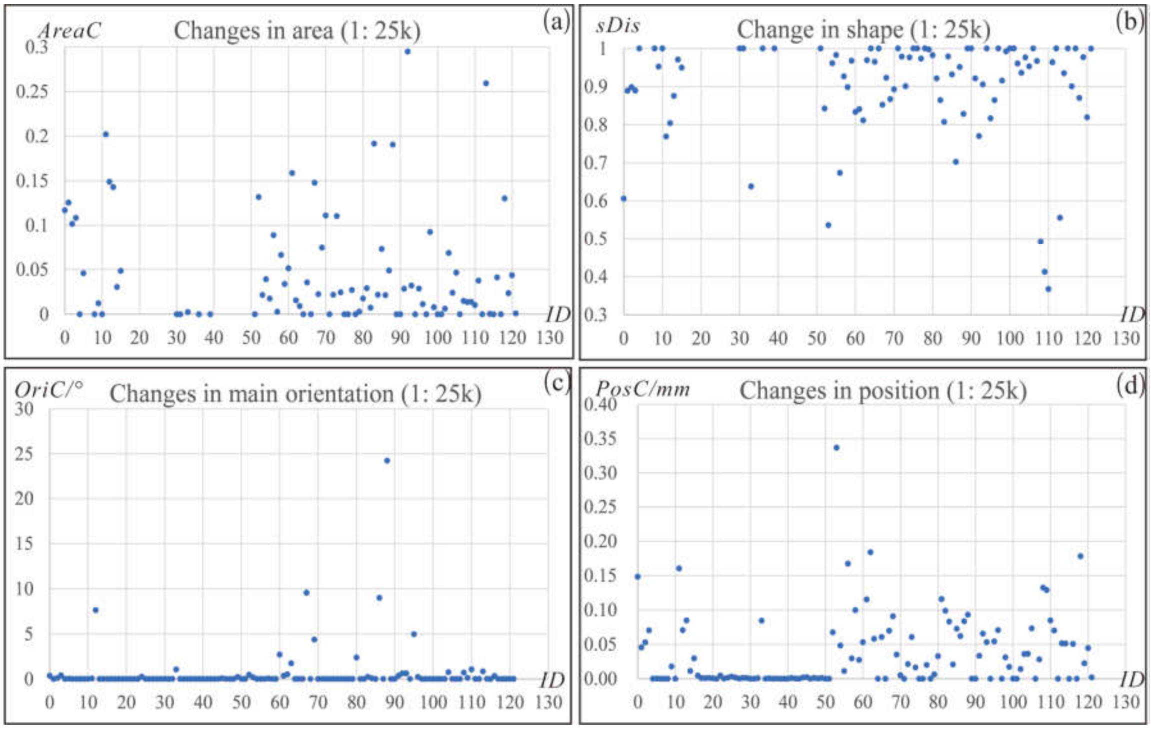

Figure 9. Comparison of changes in area (**a**), shape (**b**), main orientation (**c**) and position (**d**), shape between buildings at target and original scales.

5. Discussion

5.1. Comparisons

To validate the feasibility and adaptability of our approach, we compared simplified buildings obtained at 1:25k with our approach to the adjacent four-points (*AF*) algorithm [26], recursive regression (*RR*) algorithm [31], building enlargement (*BE*) algorithm [7] and the Simplify Building (*SB*) Tool in ArcGIS [17]. The same parameters were set as in our approach (if the algorithm requires specific parameters). The results were compared according to the generalization constraints described in Section 2.1, measured as follows: number of buildings violating minimum size (*BNS*) or granularity (*BNG*) constraint, and average changes in area (*aAreaC*), main orientation (*aOriC*), position (*aPosC*) and shape (*asDis*) between buildings at original and target scales, defined in Table 2 and Equation (5). Compared results are shown as Table 4.

As for the legibility constraints, Table 4 shows that all buildings obtained with our approach at 1:25k satisfied the legibility constraint, while 36 buildings obtained with *AF*, 42 buildings obtained with *RR*, and 33 buildings obtained with *SB* at 1:25k violated the minimum size constraint. In addition, one building obtained with *AF*, seven buildings obtained with *RR*, 65 buildings obtained with *BE*, and 47 buildings obtained with *SB* at 1:25k violated the granularity constraint. As for the preservation constraint, buildings obtained with our approach had a smaller *aOriC* compared to *AF*, *RR*, and *SB*. Buildings obtained with the listed simplification algorithms all had a small *aPosC* and *aPosC* < 0.042 mm. As for area and shape preservation constraints, buildings in our approach may be enlarged for they are too small, which may lead to a large change in area and shape. While buildings with small1 area are not simplified in *AF*, *RR* and *BE* algorithms, and these buildings will violate legibility constraints. With considering enlarged buildings: *aAreaC* was 0.809 and *asDis* was 0.819 with our approach; and the same for buildings obtained with *BE* and *aAreaC* was 0.775. Without considering enlarged buildings in our approach, *aAreaC* was 0.046, which means the change in area is also controlled at a low level with our approach; And *asDis* was 0.899 and similar to the *asDis* obtained by *AF* and *SB*, which

means shapes can also be preserved in simplified buildings by our approach as other approaches do.

Table 4. Comparison of results obtained based on our approach and other simplification algorithms.

Scale	Measures	Our approach	*AF*	*RR*	*BE*	*SB*
1:25k	*BNS*	0	36	42	0	33
	BNG	0	1	7	65	47
	aAreaC	0.809	0.009	0.091	0.775	0.013
	aOriC/°	0.627	1.000	0.742	0.002	1.189
	aPosC/mm	0.040	0.020	0.042	0.000	0.012
	asDis	0.819	0.926	0.855	0.893	0.972

Six buildings simplified by different simplification algorithms are listed in Figure 10 for detailed comparison. First, local structures are classified by considering orthogonal and non-orthogonal features of buildings in our approach, thus the approach can maintain the orthogonal and non-orthogonal features of simplified buildings. As shown in Figure 10, when Buildings A and F are simplified using our approach, their orthogonal features are maintained; and when Building E is simplified, its non-orthogonal features are maintained. However, using *AF* to simplify Buildings A and F may destroy their orthogonal features; and using *RR* is more likely to square the corners, which may lose non-orthogonal features, such as with Building E.

Second, a backtracking strategy is applied in case an unsatisfactory result is obtained when applying local-structure-based operation. This means it is more likely to obtain a satisfactory result with the backtracking strategy in our approach. For example, local-structure-based operations are triggered for the parts framed by a red rectangle in Buildings A and D, and the operations with higher priority will lead to a large decrease in area for Building A, a self-intersection, or a large increase in area for Building D. Thus, the suitable operations are selected with our backtracking strategy to obtain a satisfactory result, as shown in Figure 10. However, obtaining Building A with *AF* still violates the granularity constraint, and obtaining Building D with *AF* may lead a large change in area and position. Simplifying Building D with *RR* seems to result in oversimplification, and the same for Building E.

Third, we combine the *BE* and *TS* algorithms, as the *LS* algorithm is sometimes not applicable to simplify buildings. For example, Building B is too small to be simplified based on its local structures, and our approach applies the *BE* algorithm; and Building C is evaluated as unable to be simplified by the *LS* algorithm, and our approach applies the *TS* algorithm. However, *BE* is applied when the building violates the minimum size constraint, Buildings A, C, D, E, and F are not simplified with this algorithm. Further, when using ArcGIS, buildings with small area, such as Buildings A, B, and C, will not be simplified. Thus, a combination of the *BE* and *TS* algorithms may help produce a more reasonable simplified result.

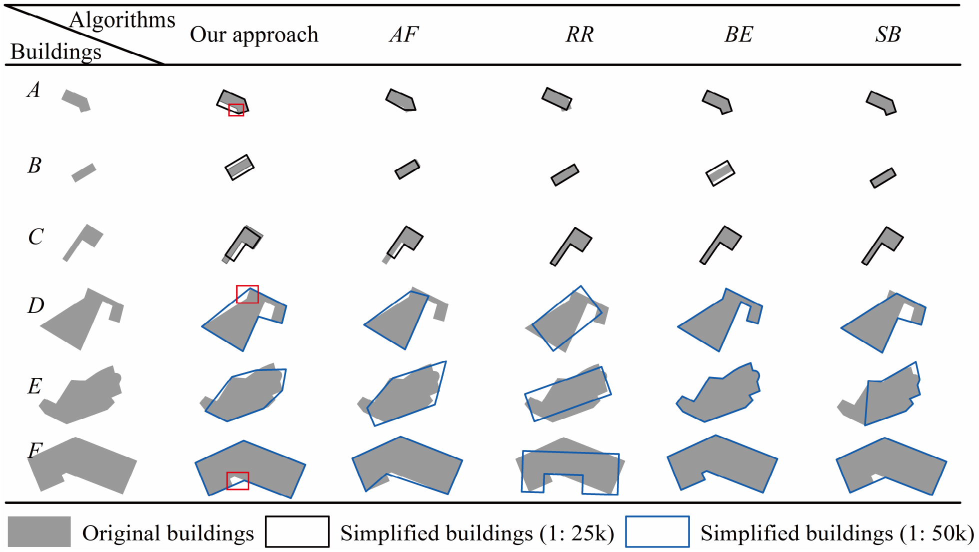

Figure 10. Comparison of typical buildings simplified using different simplification algorithms.

5.2. Possible Use for Continuous Scale Transformation of Buildings

As buildings are simplified scale-by-scale in our approach, it can be useful to build a continuous data structure for buildings in future applications. For a given building (B_i) that is simplified to a target scale progressively scale-by-scale, we can record each representation of B_i obtained after simplification with the corresponding scales in two matched rows as $BR = \{BR^1{}_i, BR^2{}_i, \ldots, BR^n{}_i\}$ and $SS = \{Scale^1{}_i, Scale^2{}_i, \ldots, Scale^n{}_i\}$. If $Scale^m{}_i$ in SS is recorded in ascending order and each $Scale^m{}_i$ in SS meets the condition $Scale^m{}_i < Scale^{m+1}{}_i, Scale^m{}_i \in SS$, then $BR^{m+1}{}_i$ can be considered to be obtained based on $BR^m{}_i$ from $Scale^m{}_i$ to $Scale^{m+1}{}_i$. Thus, the representation of B_i can be obtained by searching along SS with any given target scale. The condition $Scale^m{}_i < Scale^{m+1}{}_i, Scale^m{}_i \in SS$ can be met by deleting $Scale^m{}_i$ in SS, which may not meet the condition, and its corresponding representation $BR^{m-1}{}_i$ also needs to be deleted in BR. Then matched rows (BR and SS) after deletion are considered as the data structure of B_i to achieve its continuous scale representation.

For a given building (B_i) at 1:10k scale in Figure 11 as an example, its first representation is denoted as $BR^1{}_i$. A new representation ($B^{m+1}{}_i$) of B_i can be obtained by simplifying $BR^m{}_i$ for violating the legibility constraint. The parameters describing the legibility constraint are recorded in Table 5. The relations between $B^{m+1}{}_i$ and its corresponding scales are defined in Section 3.3. Thus, every representation and their corresponding scales are obtained and recorded with two matched rows, as shown in Figure 11. Intermediate scales that don't meet the condition $Scale^m{}_i < Scale^{m+1}{}_i, Scale^m{}_i \in SS$ (scales marked in red in Figure 11) are deleted. Thus, two matched rows after the deletion, $BR = \{BR^1{}_i, BR^2{}_i, BR^3{}_i, BR^5{}_i, BR^7{}_i, BR^8{}_i\}$ and $SS = \{10000, 11000, 15333, 19000, 42000, 56400\}$ can be considered as the data structure to support the continuous scale transformation of B_i. Given a target scale of 1:25k, the target representation can be obtained as $BR^5{}_i$ for $25000 \in [19000, 42000]$, and given a target scale of 1:50k, the target representation can be obtained as $BR^7{}_i$ for $50000 \in [42000, 56400]$. If a given target scale is smaller than 1:56,400, the target representation can be obtained by scaling BR_7 according to the given scale.

Table 5. Parameters describing legibility constraint for representations of B_i in Figure 11, which can help obtain corresponding scale of each representation. Invalid scales are highlighted in the table.

Representation	$BR^1{}_i$	$BR^2{}_i$	$BR^3{}_i$	$BR^4{}_i$	$BR^5{}_i$	$BR^6{}_i$	$BR^7{}_i$	$BR^8{}_i$
AreaB (m²)	1370	1357	1386	1421	1390	1626	1601	2300

LB (m)	56.8	56.8	56.8	56.8	56.8	56.8	56.8	56.8
WB (m)	35.3	35.3	35.3	35.3	31.0	3.3	28.2	40.5
MLe (m)	3.3	4.6	5.7	4.4	12.6	3.3	28.2	40.5
Violation	GT_L	GT_L	GT_L	GT_L	GT_L	GT_L	GT_L	ST_W
Start scale (1:*M*)	10000	11000	15333	19000	***14666***	42000	***11000***	56400
Validation	Valid	Valid	Valid	Valid	***Invalid***	Valid	***Invalid***	Valid

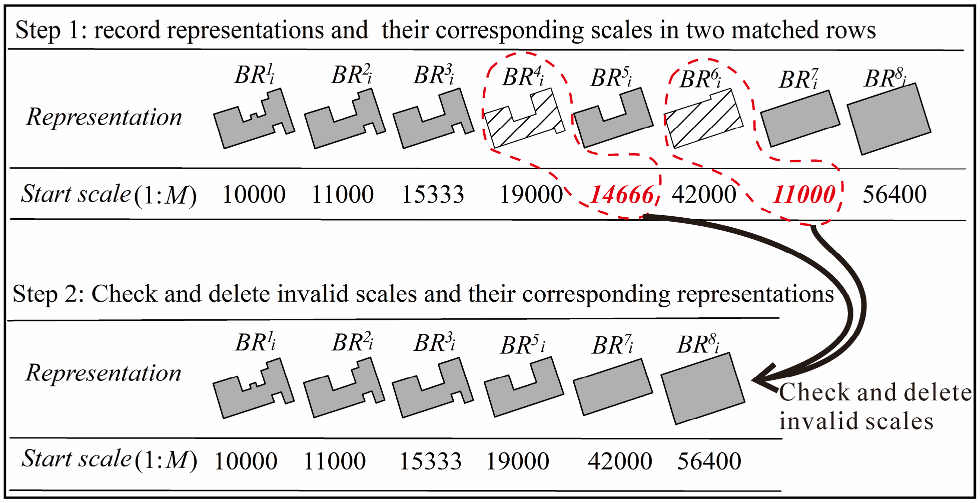

Figure 11. Construction of data structure: two matched rows recording representation of B_i and its corresponding scales: $BR=\{BR^1{}_i, BR^2{}_i, \ldots, BR^n{}_i\}$ and $SS=\{Scale^1{}_i, Scale^2{}_i, \ldots, Scale^n{}_i\}$. Representation of B_i at target scale can be obtained by searching along with *SS* and *BR*.

5.3. Limitations

Though our approach has been shown to be effective at simplifying buildings in a dataset, some limitations also need to be discussed.

First, generalization requirements may differ across scales, regions, and even user demands. Although requirements can be controlled by parameters defined in our approach or set by users, our approach still has some difficulty in automatically assigning suitable operations for buildings with different generalization requirements. For example, minimum size constraints may differ across scales. For example, according to the Chinese National Administration of Surveying [39], the minimum length and width of a building's minimum bounding rectangle at 1:5k and 1:10k scale are denoted as 1.0 and 0.7 mm, and those at 1:25k and 1:50k are denoted as 0.7 and 0.5 mm. In our approach, however, once the minimum length and width of the buildings' minimum bounding rectangle are set by the user, they will not change across scales.

As another example, the characteristics of buildings may differ across regions, e.g., orthogonal Building A and non-orthogonal Building B shown in Figure 12a. In simplification, orthogonal features may be the first to be maintained in orthogonal buildings, and area may be the first to be maintained in non-orthogonal buildings [16]. Figure 12a shows that orthogonal Building A may have a more reasonable result with higher priority in the shape-preservation constraint, while non-orthogonal Building B may have a more reasonable result with higher priority in the area-preservation constraint. Though different requirements for orthogonal and non-orthogonal buildings can be controlled by setting the priority of the preservation constraints in our *LS* algorithm, a mechanism to set different priorities in the preservation constraints for buildings with different characteristics is still required (described in Section 3.4). Many tools and standards have been introduced for expression and reasoning of knowledge or rules with the development of the knowledge graph. If different generalization requirements in building simplification are modeled as knowledge or rules with an effective knowledge representation model, it may be possible

to apply our approach to buildings with different generalization requirements across scales, regions, and even user demands.

Second, simplification is just one kind of generalization operation. It is sometimes not suitable to only use simplification in building generalization. For example, as shown in Figure 12b, original adjacent buildings after simplification may become too close to be distinguished by users; here, Buildings C, D, and E or Buildings F and G. Thus, some other generalization operations, such as selection, aggregation and typification may also be needed to achieve a reasonable result. Hence, to improve our proposed approach in the future, other generalization operations can be combined by applying them before or after the simplification of buildings.

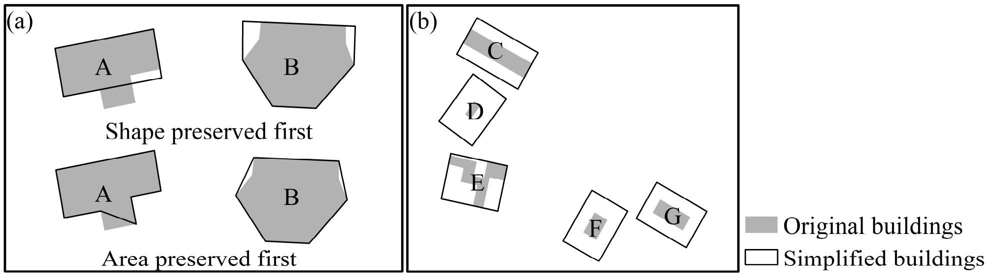

Figure 12. Limitations. (**a**) Buildings A and B are simplified with higher priority separately in shape-preservation and area-preservation constraints; (**b**) Buildings C, D, E or buildings F, G may become too close to be distinguished after simplification with a large reduction in scale.

6. Conclusion

We propose a progressive and combined building simplification approach that considers cartographic requirements based on local structure classification and backtracking strategy. In our approach, different simplification operations are triggered for buildings that violate minimum size or granularity constraints to generate the next scale representation of simplified buildings. The relations are computed between scales and parameters that describe minimum size or granularity, and simplified buildings at the target scale are then obtained. Rules supporting their application are built based on preservation constraints of buildings while applying specific simplification operations. Experiments show that simplified buildings obtained with our approach satisfy legibility constraints and the change in area, orientation and position of simplified buildings are controlled within certain range by comparing with the results generated based on other four simplification algorithms. Our results also show the possible use of our approach in continuous scale representation of buildings.

Future works will focus on the following: (1) a knowledge model to express cartographic knowledge or rules for building simplification across scales and regions, which can support our approach becoming suitable for various generalization requirements or user demands, and (2) approaches that combine semantic generalization operations, such as selection and aggregation, to achieve continuous transformation of building groups, not just single buildings.

Author Contributions: Methodology, Zhiwei Wei; Resources, Zhiwei Wei, Yang Liu and Lu Cheng; Software, Zhiwei Wei and Yang Liu; Validation, Zhiwei Wei and Lu Cheng; Writing – original draft, Zhiwei Wei; Writing – review & editing, Zhiwei Wei and Su Ding. All authors have read and agreed to the published version of the manuscript.

Funding: This work was research was funded by National Natural Science Foundation of China, grant number [41871378].

Data Availability Statement: The data that support the findings of this study are openly available in Mendeley Data at https://data.mendeley.com/datasets/tgngv7dkgn/3.

Acknowledgments: The authors would like to thank the editors and the anonymous reviewers for their helpful and constructive comments that greatly contributed to improve the manuscript.

Conflicts of Interest: No potential conflict of interest was reported by the author(s).

References

1. Lee, D. New cartographic generalization tools. In Proceedings of the 19th International Cartographic Conference of the ICA, Ottawa, ON, Canada, 14–21 August 1999; pp. 1235–1242.
2. Sester, M. Generalization based on least squares adjustment. *Int. Arch. Photogramm. Remote Sens.* **2000**, *XXXIII*, 931–938.
3. Ai, T.; Cheng, X.; Liu, P.; Yang, M. A shape analysis and template matching of building features by the Fourier transform method. *Comput. Environ. Urban Syst.* **2013**, *41*, 219–233.
4. Burghardt, D.; Schmid, S. Constraint-based evaluation of automated and manual generalized topographic maps. In *Cartography in Central and Eastern Europe*; Springer, Berlin, Heidelberg, 2009; pp. 147–162.
5. Rainsford, D.; Mackaness, W. Template matching in support of generalization of rural buildings. In *Advances in Spatial Data Handling, Proceedings of the 10th International Symposium on Spatial Data Handling, Ottawa, ON, Canada, 9–12 July*; Richardson, D.E., Van O, P., Eds.; Springer: Berlin/Heidelberg, Germany, 2002; pp. 137–151.
6. Yan, X.; Ai, T.; Zhang, X. Template matching and simplification method for building features based on shape cognition. *Int. J. Geo Inf.* **2017**, *6*, 250.
7. Wang, L.; Guo, Q.; Liu, Y.; Sun, Y.; Wei, Z. Contextual Building Selection Based on a Genetic Algorithm in Map Generalization. *Int. J. Geo-Inf.* **2017**, *6*, 271.
8. Guo, Q. The method of graphic simplification of area feature boundary as right angle. *Geomat. Inf. Sci. Wuhan Univ.* **1999**, *24*, 255–258.
9. Sester, M. Optimization approaches for generalization and data abstraction. *Int. J. Geogr. Inf. Sci.* **2005**, *19*, 871–897.
10. Chen, W., Long, Y., Shen, J.; Li, W. Structure recognition and progressive of then concaves of building polygon based on constrained D-Tin. *Geomat. Inf. Sci. Wuhan Univ.* **2011**, *36*, 584–587.
11. Cheng, B.; Liu, Q.; Li, X.; Wang, Y. Building simplification using backpropagation neural networks: A combination of cartographers' expertise and raster-based local perception. *GISci. Remote Sens.* **2013**, *50*, 527–542.
12. Buchin, K.; Meulemans, W.; Renssen, A.V.; Speckmann, B. Area-preserving simplification and schematization of polygonal subdivisions. *ACM Trans. Spat. Algorithms Syst.* **2016**, *2*, 1–36.
13. Oosterom P V.; Meijers, M. Vario-scale data structures supporting smooth zoom and progressive transfer of 2D and 3D data. *Int. J. Geogr. Inf. Sci.* **2014**, *28*, 455–478.
14. Huang, L.; Ai, T.; Oosterom, P.V.; Yan, X.; Yang, M. A matrix-based structure for vario-scale vector representation over a wide range of map scales: The case of river network data. *ISPRS Int. J. Geo-Inf.* **2017**, *6*, 218.
15. Peng, D.; Touya, G. Continuously generalizing buildings to built-up areas by aggregating and growing. In *UrbanGIS'17: 3rd ACM SIGSPATIAL Workshop on Smart Cities and Urban Analytics, 7–10 November 2017, Redondo Beach, CA, USA*; ACM: New York, NY, USA, 2017; 8p.
16. Shen, Y.; Ai, T.; Li, C. A simplification of urban buildings to preserve geometric properties using superpixel segmentation. *Int. J. Appl. Earth Observ. Geoinf.* **2019**, *79*, 162–174.
17. Wang, Z.; Lee, D. Building simplification based on pattern recognition and shape analysis. In Proceedings of the 9th International Symposium on Spatial Data Handling, Beijing, China, 10-12 August 2000; pp. 58–72.
18. Steiniger, S.; Taillandier, P.; Weibel, R. Utilising urban context recognition and machine learning to improve the generalization of buildings. *Int. J. Geogr. Inf. Sci.* **2010**, *24*, 253–282.
19. Ruas, A. Modèle de Généralisation de Données Géographiques à Base de Contraintes et d'autonomie. Ph.D. Thesis, Sciences de l'Information G´eographique, Marne La Vallee, France, 1999.
20. Duchêne, C.; Christophe, S.; Ruas, A. Generalisation, symbol specification and map evaluation: Feedback from research done at COGIT laboratory, IGN France. *Int. J. Digit. Earth* **2011**, *4* (Suppl. S1), 25-41.
21. Duchêne, C.; Ruas, A.; Christophe, C. The CartACom model: Transforming cartographic features into communicating agents for cartographic generalization. *Cartogr. Geogr. Inf. Syst.* **2012**, *26*, 1533–1562.
22. Maudet, A.; Touya, G.; Duchêne, C.; Picault, S. DIOGEN, a multi-level oriented model for cartographic generalization. *Int. J. Cartogr.* **2017**, *3*, 121–133.
23. Wang, L.; Guo, Q.; Wei, Z.; Liu, Y. Spatial conflict resolution in a multi-agent process by the use of a snake model. *IEEE Access* **2017**, *5*, 24249–24261.
24. Stoter, J.; Smaalen, J.;Bakker, N.; Hardy, P. Specifying Map Requirements for Automated Generalization of Topographic Data. *Cartogr. J.* **2009**, *46*, 214–227.
25. Regnauld, N. Contextual building typification in automated map generalization. *Algorithmica* **2001**, *30*, 312–333.
26. Xu, W., Long, Y., Zhou, T.; Chen, L. simplification of building polygon based on adjacent four-point method. *Acta Geod. Cartogr. Sin.* **2013**, *42*, 929–936.
27. Lokhat, I.; Touya, G. Enhancing building footprints with squaring operations. *J. Spat. Inf. Sci.* **2016**, *13*, 33–60.
28. Duchêne, C.; Bard, S.; Barillot, X. Quantitative and qualitative descr-iption of building orientation. In Proceedings of the 5th Workshop on Progress in Automated Map Generalization, Paris, France, 28-30 April 2003.

29. Liu, Y., Guo, Q., Sun, Y.; Ma, X. A combined approach to cartographic displacement for buildings based on skeleton and improved elastic beam algorithm. *PLoS ONE* **2014**, *9*, e113953.
30. Zhang, X.; Stoter, J.; Ai, T.; Kraak, M.-J.; Molenaar, M. Automated evaluation of building alignments in generalized maps. *Int. J. Geogr. Inf. Sci.* **2013**, *27*, 1550–1571.
31. Bayer, T. Automated building simplification using a recursive approach. In *Cartography in Central and Eastern Europe*; Gartner, G., Ortag, F., Eds.; Berlin, Heidelberg: Springer, 2009; pp. 121–146.
32. Damen, J.; van Kreveld, M.; Spaan, B. High quality building generalization by extending the morphological operators. In Proceedings of the 12th ICA Workshop on Generalization and Multiple Representation, Montpellier, France, 20–21 June 2008.
33. Meijers, M. Building simplification using offset curves obtained from the straight skeleton. In Proceedings of the 19th ICA Workshop on Generalisation and Multiple Representation, Helsinki, Finland, 14 June 2016.
34. Kada, M. Aggregation of 3D buildings using a hybrid data approach. *Cartogr. Geogr. Inf. Syst.* **2011**, *38*, 153–160.
35. Wei, Z.; He, J.; Wang, L.; Wang, Y.; Guo, Q. A collaborative displacement approach for spatial conflicts in urban building map generalization. *IEEE Access* **2018**. doi:10.1109/ACCESS.2018.2836188.
36. Fan, H.; Alexander, Z.; Wu, H. Detecting repetitive structures on building footprints for the purposes of 3D modeling and reconstruction. *Int. J. Digit. Earth* **2017**, *10*, 785–797.
37. Shen, Y.; Ai, T.; He, Y. A new approach to line simplification based on image processing: A case study of water area boundaries. *ISPRS Int. J. Geo-Inf.* **2018**, *7*, 41.
38. Taillandier, P.; Duchêne, C.; Drogoul, A. Automatic revision of rules used to guide the generalization process in systems based on a trial and error strategy. *Int. J. Geogr. Inf. Sci.* **2011**, *25*, 1971–1999.
39. Yang, M.; Yuan, T.; Yan, X.; Ai, T.; Jiang, C. A hybrid approach to building simplification with an evaluator from a backpropagation neural network. *Int. J. Geogr. Inf. Sci.* **2021**. doi:10.1080/13658816.2021.1873998.
40. National Administration of Surveying, Mapping and Geoinformation of China. *Compilation Specification for National Fundamental Scale Maps-Part1: Complilation Specifications for 1:25,000, 1:50,000 & 1:100,000 Topographic Maps*; China Zhijian Publishing House: Beijing, China, 2008.
41. Ordnance Survey. OS OPEN MAP-LOCAL: Product Guide and Technical Specification. 2016. Available online: https://www.ordnancesurvey.co.uk/documents/os-open-map-local-product-guide.pdf (accessed on 2020-10-21).
