# Supplementary material for: Estimation of Undetected Asymptomatic COVID-19 Cases in South Korea Using a Probabilistic Model
Source: Int J Environ Res Public Health. 2021 May 6;18(9):4946. doi: 10.3390/ijerph18094946 (PMC8124955; doi:10.3390/ijerph18094946)
Supplement: Supplementary file 1 [file ijerph-18-04946-s001.zip › 5/ijgi-1157178 - for pub.pdf]

International Journal of *Geo-Information*

MDPI

Article

Crowdsourcing of Popular Toponyms: How to Collect and Preserve Toponyms in Spoken Use

Daniel Vrbík [1,*] and Václav Lábus [2]

[1] Department of Applied Mathematics, Faculty of Science, Humanities and Education, Technical University of Liberec, 461 17 Liberec, Czech Republic
[2] Department of Czech Language and Literature, Faculty of Science, Humanities and Education, Technical University of Liberec, 461 17 Liberec, Czech Republic; vaclav.labus@tul.cz
* Correspondence: daniel.vrbik@tul.cz

Abstract: The article presents a process of collecting unstandardized toponyms, in particular urbanonyms (place names denoting objects located in the cadastre of the city), within the territory of two municipalities in the Czech Republic. The collecting process was performed in two phases by crowdsourcing, using a web map application created especially for this purpose. In the first phase (October 2019–September 2020) it was collecting as many unstandardized toponyms as possible. In the second phase (October 2020–January 2021) we focused on the degree of the knowledge of these toponyms among the population living within the studied territory. The interest on the side of the general public was surprising in both phases. In the first phase, over five hundred respondents submitted more than two and a half thousand place names, most of them during the first two weeks. More than nine hundred respondents actively participated in the second phase, thanks to which we received an average of 200 responses for each place name. As regards the motivation of the public, it was most often altruism, patriotism, and curiosity that stimulated them; in the second phase, the element of gamification, embedded into the map application, also had a positive effect. The collected data can be used, for instance, in the activities of local authorities in the process of standardization of place names or as reference data for maps used within the integrated rescue system.

Keywords: crowdsourcing; official toponyms; web map application; gamification; motivation; popular toponyms; urban toponyms; place names

Citation: Crowdsourcing of Popular Toponyms: How to Collect and Preserve Toponyms in Spoken Use. *ISPRS Int. J. Geo-Inf.* **2021**, *10*, 303. https://doi.org/10.3390/ijgi10050303

Academic Editors: Eufemia Tarantino and Wolfgang Kainz

Received: 9 March 2021
Accepted: 2 May 2021
Published: 5 May 2021

Publisher's Note: MDPI stays neutral with regard to jurisdictional claims in published maps and institutional affiliations.

Copyright: © 2021 by the authors. Licensee MDPI, Basel, Switzerland. This article is an open access article distributed under the terms and conditions of the Creative Commons Attribution (CC BY) license (http://creativecommons.org/licenses/by/4.0/).

1. Introduction

The article presents the potential of crowdsourcing data collecting regarding the data of linguistic and geographic character. Here we describe the methodology and current results of the project called Living Names (in Czech: Živá jména). The project, which was implemented in 2019, focuses on the research into popular toponyms in the city of Liberec in the Czech Republic.

Toponyms in the city, so-called urban toponyms or urbanonyms, can be divided into two categories: the official (standardized) and the unofficial (unstandardized, popular) ones. The standardized urbanonyms are characterized by a binding form, which is approved by local authorities and are used in official communication, on signs, in the state map series and derived plans, etc. Contrastingly, the popular urbanonyms are primarily used in common, unofficial, or semi-official communication and are perceived as a specific manifestation of the social and geographical stratification of their users [1,2], and that is why they are more variable than relatively stable standardized names. Popular urbanonyms may refer either to the same places or buildings as the official names, or to places or buildings beyond the scope of the official orientation system.

The Living Names project (in Czech: Živá jména) was created to collect popular urbanonymy through crowdsourcing. The project, implemented within the territory of the

city of Liberec and the municipality of Stráž nad Nisou (Czech Republic), started in October 2019 (https://mapy.fp.tul.cz/zivajmena/, accessed on 5 May 2021).

The reason why the city and the municipality were chosen for this project is the fact that the municipality of Stráž nad Nisou is completely surrounded by Liberec (in 1976–1990 it was even a part of the city of Liberec) and can thus be considered its suburb. Liberec is the fifth largest city in the Czech Republic in terms of population and together with Stráž nad Nisou its population equals 108,000 [3]. The relief of the territory, which also influences the local toponymy, is rather complicated. The territory lies in a basin between two mountain ridges, the axis of which is the Lužická Nisa river. From a historical point of view, this is an area where, until 1945, a strong German majority was present. After the end of World War II and the expulsion of the German-speaking population, the local orientation system underwent a radical reconstruction, both its official segment (the street and local place names went through a process of Czechization), and the popular one too (in fact, a new system was created, reflecting the communication and orientation needs of new settlers). The official urbanonymy went through further significant changes after the Velvet Revolution (1989), when more than 30 street names were changed (1991).

The long-term goal of the presented project is to create a database of popular names used during a specific period of time in the territory of two municipalities, supplemented by other characteristics of these names, using crowdsourcing methods and tools of geographic information systems, so that the local knowledge can be easily disseminated. The aim of the article is to present the possibilities and the limitations of crowdsourcing-based collecting of data of linguistic and geographical nature so that it is possible to apply this procedure, with possible modifications, to other localities. The article is structured as follows: (Section 2) background and related work, (Section 3) common requirements for collecting popular names and general introduction to technical security; (Section 4) description of the phase of collecting the toponyms and its general results; (Section 5) description of the phase of determination of the knowledge of the toponyms collected and its general results; (Section 6) discussion of the knowledge acquired, comparison with the results of similar projects, research limitations and a proposal of their solutions; and (Section 7) conclusions.

2. Background and Related Work

The homogeneous urban environment places specific demands on orientation, which means standardized toponymical techniques would meet with difficulty. Thus in popular urbanonymy, the dominating elements—as regards orientation and communication—are rather distinctive buildings or structures, not the names of the streets [4], [5]. The need to name distinctive buildings or structures in urban space is a consequence of creating a general mental idea of a physical world, which is developed by every individual [6]. Naming places, especially "from below", i.e., by ordinary users, is important for crystallizing a place's identity. The popular names then impersonate the projection of such an individual or shared mental map into oral communication.

Although the research into popular urbanonymy may result in interesting findings in the field of perception of the surrounding space and its linguistic representation, its implementation is rather demanding. Both the primarily spoken use of popular toponyms and the different orientation and communication needs of variously defined social groups of users imply that popular toponyms are very difficult to collect. An obstacle to developing quality databases containing unstandardized toponymy is, above all, the difficult excerption of sufficiently representative and authentic materials. Popular toponymy is excerpted mostly by traditional onomastic ways, especially by means of surveys or interviews, see [7]. Due to democratization of geographical information systems, e.g., [8], which make it possible to capture the spatial component of toponyms easily, and due to the shift of web technologies to Web 2.0 [9] (or in case of its geographic version termed as geoweb [10,11]), it is possible to involve the general public in the collecting process through the participation of the public, and on condition that appropriate procedures are

used, it is possible to gain necessary information quickly and efficiently [12–17]. This approach, known as "crowdsourcing", is successfully applied in different fields of science and research [18–21] and it also is used in the process of collecting toponyms [12–14,16,22].

The term crowdsourcing was coined by [23] to describe the phenomenon of outsourcing the tasks of the company through the use of the collective intelligence (undefined, of a large group of people or "crowd"). Although originally the term crowdsourcing was focused on the business context the term is also used in other situations where, in the form of an open call, a request is made for the voluntary involvement of a potentially large group of participants in a specific activity (most often data collection) [24]. According to [25] in the case of crowdsourcing the idea is that information obtained with the help of a large group of people is more accurate than information from a single person. However, it is not just about collecting data, but also about working together to find a solution to a problem [26], which can be called the "wisdom of the crowd" [27], thus crowdsourcing can be present in citizen science projects in Level 1 of engagement, described by [28].

We can find several efforts in the process of collecting popular names via crowdsourcing methods. For example, the Swedish National Mapping Agency (Lantmäteriet) in 2011 developed a mobile application for data collection, which led to 70,000 reported names in urban areas [29]. With the cooperation of the Maritime and Coastguard Agency in the United Kingdom, Ordnance Survey developed a crowdsourced gazetteer of popular toponyms of the UK coastline to help Coastguard teams quickly localize people when responding to emergency calls [30,31]. Shetland Amenity Trust has decided to collect popular names as a part of its orally transmitted history [32], and a similar technique is used in project Meitheal Logainm in Ireland [33]. Local toponyms in Tyrol are collected within the crowdsourcing project Field-name-survey in the Province in Tyrol (Austria) [13]. Gamified crowdsourcing-based collection of toponyms in Spain is presented in [14]. Crowdsourcing-based data collection is also a fundamental part of the project Place Names (in Czech: Názvy míst), initiated in 2011 by the University of Ostrava [15]. This is the only research on popular toponyms in the Czech Republic that is methodologically comparable to the project Living Names presented in this article.

3. Methods

From our perspective, there are three important aspects involved in the study of popular toponyms: a) linguistic (name, i.e., linguistically formed reflection of an object); b) geographical (to which object/space the name refers); and c) social (users of the name, their number, i.e., the degree of knowledge of the name, demographic structure, i.e., the age, domicile, social group), see so-called language level, object level, and communication level as necessary preconditions for formation and use of proper names, cf. [34], [5]. For this reason, the project was divided into two, consecutive phases:

- The collection phase: involving as many respondents as possible and collecting as many toponyms as possible, including the micro-social ones, used only by a very small group of the population (Section 4), i.e., collecting information on the linguistic and geographical aspects of the toponyms;
- The knowledge determination phase: determination of the degree of knowledge of the names collected in the first phase among the inhabitants of the studied territory (Section 5), i.e., collecting the information concerning the social aspect of the toponyms.

The common denominator of both phases is the requirement for a sufficient number of respondents and—with regard to the variability of popular toponymy—also their engagement within a short period of time. Given the availability of the Internet in the Czech Republic—Internet is used by 87% of the population in the category of 16 to 74 years, which is comparable to the average of EU countries [35], it seems to be the most suitable way to use a web application and continuously promote it through regional media and social networks. There is a potential disadvantage in this approach, and it is the exclusion

of certain groups of the population [26]. We tried to compensate for this disadvantage by persuading younger respondents to help their parents and grandparents overcome technical problems and go through both phases with them.

A separate web application was created for each phase. They each use JavaScript libraries (Leaflet.js in version 1.5.1, its plugins, jQuery in version 3.4.1) and the Bootstrap framework (v. 4.3.1), so the application can be used both on the desktop and on mobile phones. When designing them, we took into account the current trends in UI and UX to ensure their operation would be intuitive and easy. The frontend of these applications is simple and follows a minimalistic design so that their operation does not distract users from their goals, i.e., collecting the toponyms and determining their knowledge. The server part is provided by the PHP (v. 7.2.29) and PostgreSQL (v. 10.12) with the Postgis extension, where all information about respondents, added names, their knowledge and the like is stored in interconnected tables (Figure 1). There is a common database for both phases.

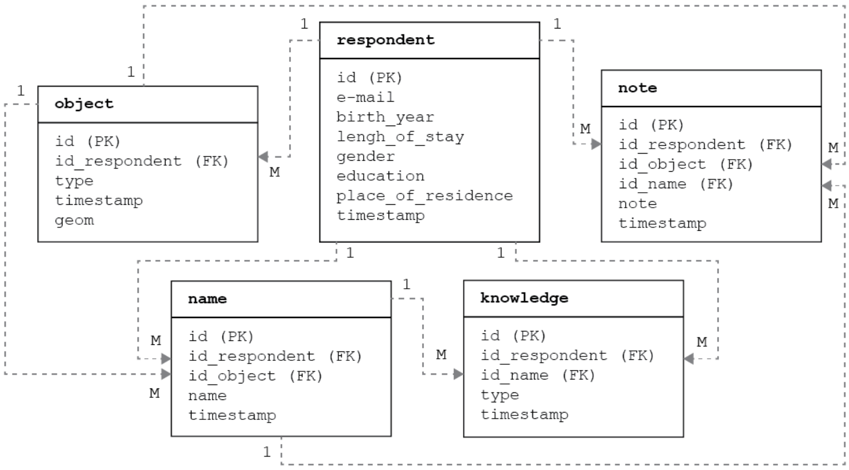

Figure 1. The general structure of the database in the Living Names project.

Instead of our own web map application, we also considered other existing tools used for creating (geo) surveys (Esri Survey123, Google Forms, Fulcrum etc.), but they did not meet our requirements (refer to next chapters) and if we combined them with other tools we would not create a user-friendly interface. The applications are available at https://mapy.fp.tul.cz/app/zj-demo and https://mapy.fp.tul.cz/app/zj-demo/znalost (both accessed on 5 May 2021).

4. Phase 1—Methodology of Collecting the Toponyms

In this phase, there are two main aspects—the linguistic (name) and the geographic (place/space to which the name is linked; in our application, due to onomastic conventions, the place or space is referred to as the "object").

The object is represented by a point, which is easier to comprehend by the respondents [36], although some of the objects (streets, squares, water areas, brownfields, etc.) are extensive or spacious. Another reason for using the point is the different spatial perception of specific objects by individual respondents, causing difficulties when connecting other variations of an object name already existing in the database. With larger objects, the respondent should place the point approximately in the middle of the object.

Respondents can add any names they know to each of the objects. In addition, we also ask whether the particular name is actively used or not (active vs. passive knowledge). Notes can be added in which the respondents can explain the etymology,

record the history of the object or provide any other information that is important from the respondent's point of view.

In order to collect the maximum number of toponyms possible, we believe that it is appropriate to make the names already recorded available to respondents because:

- They may inspire the respondents to add more names to the objects in the map, or to add the names to the other objects that are missing;
- They may inspire the addition of other variations of the name to places that already have their names, or the addition of other relevant information (notes);
- This allows you to show your knowledge to others in the community;
- This is a proactive approach that helps to eliminate the stress of being the first;
- This approach allows a limited validation of the data by other respondents.

Some of the above mentioned reasons also motivate contributors to begin contributing or continue contributing to Wikipedia [37] or other crowdsourcing projects. The application, which meets the condition of showing the previously added data, also serves as a tool for viewing it. It therefore fulfills two functions at the same time—the function of data collecting, and the function of presenting the results as well.

Due to the requirement that the application also allows you to browse the records added, its layout is map-centric, i.e., most of the website space is taken by the map while a smaller part is used for a panel with instructions, survey and information about the selected object (see Figure 2). The map controls follow from the standard layout used in other web maps. In terms of control, the map contains only the most important tools needed for working with it. Scrolling beyond the specified area is limited, even when searching for addresses.

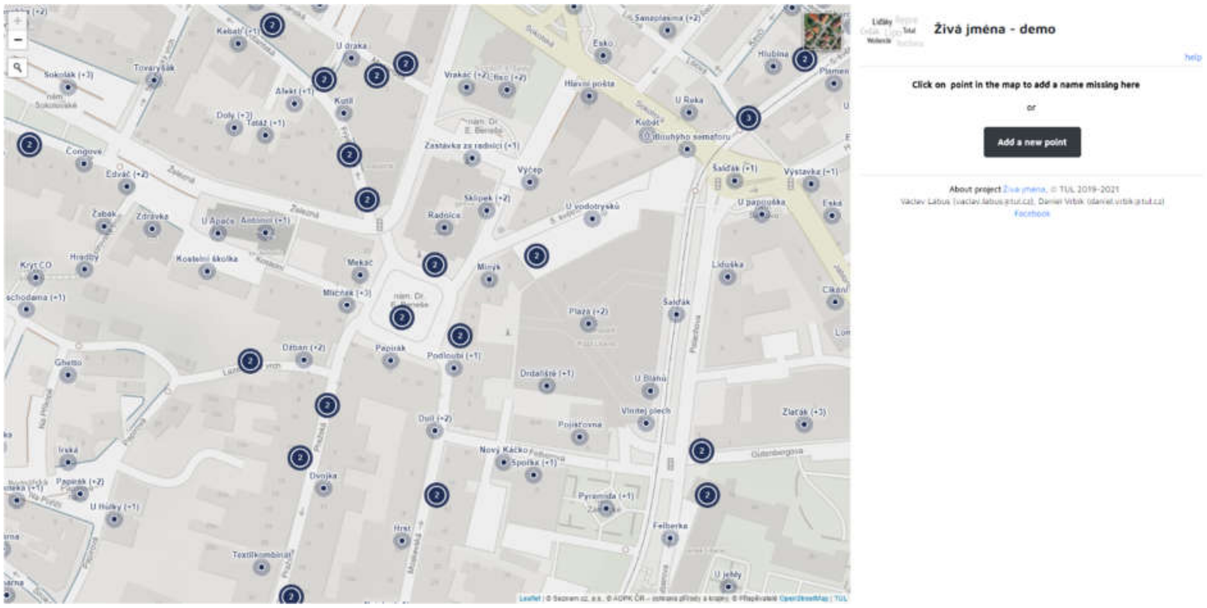

Figure 2. A view of the desktop version of the application. The application is developed in the Czech language. For the demo version, refer to https://mapy.fp.tul.cz/app/zj-demo/ (accessed on 5 May 2021).

The cartographic design of the map reflects the purpose of the application. The map portal Mapy.cz (according to SimilarWeb 2020 [38], the most used portal in the Czech Republic) is used, in which the colours are suppressed so that they do not distract the respondents from the main topic. Alternatively, ortho images may be viewed. The objects are shown by means of a dot, while the outer semi-transparent circle indicates possible uncertainty with the specified position of the object.

With regard to the topic of the map (place names), it was necessary to figure out how to display the collected names in the map. As some of the objects are associated with multiple names and displaying all of these different names would overload the map, making it disorganized and confusing, only the first name added to the object and the number of variations are shown in the map. The concrete names appear listed in the sidebar after clicking on the object. For the same reason, the objects in the map are clustered. If the objects are so close to each other that they remain in the cluster even on the largest possible scale, they will appear only after clicking on the cluster (Figure 3).

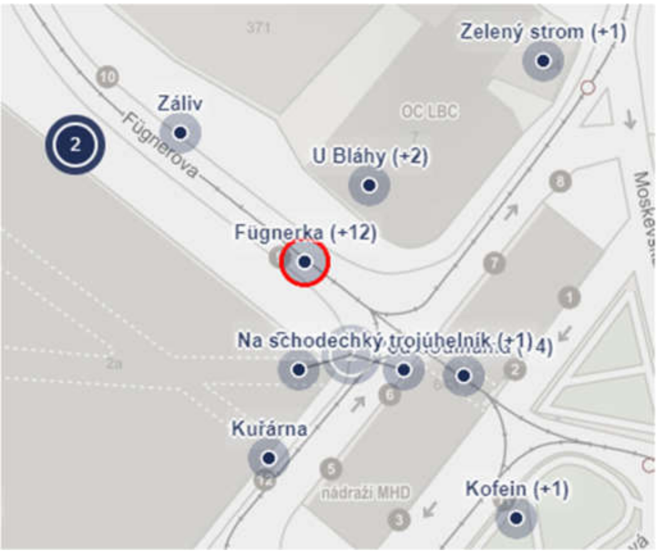

Figure 3. The suppressed map colors do not distract the respondents´ attention when reading the map. The clustering partially solves the problem of possible graphical conflicts between the objects. If objects are too close to each other, they appear with a guide line. The numbers next to the names show the numbers of other name variations. The selected object is highlighted with a red contour.

At the beginning of the collecting phase, the respondents could work with the application as registered users (by entering their e-mail address) or anonymously—in which case we would actively inform them during the work (repeated display of the modal window) what the anonymous approach means for the research results and we motivated them to register. Optionally, they could add their demographic data (gender, education, age, length of stay in the territory and residence, and anonymized using a hexagonal grid). The option of anonymous access or non-filling in the demographic data limits the amount of resulting analyzes, but it can increase the number of the people involved and the data collected, in some cases without compromising the quality of the data [39,40]. In both modes it was possible to add new names, objects and notes in the map. The only disadvantage for anonymous respondents was the inability to edit their work later on (for instance, when mistyping or adding new information). Since February 2020, when the database contained 99% of the current content, we have disabled the option of adding data anonymously (browsing without registration is possible anyway) and the added names are subject to our approval. The reason for doing this was an attempt at greater control over the quality of the entered data.

A key aspect of the success of crowdsourcing is, first and foremost, its continuous promotion and communication towards the public involved. The workflow promotion of the first phase of the project resided in the release of an introductory press release, which was then distributed to several regional media. The press release was also adopted by the Czech News Agency, which increased its impact. During the autumn of 2019, several interviews with journalists took place, which continuously promoted the project. We also

set up a Facebook page (https://www.facebook.com/zivajmena, accessed on 5 May 2021), which we periodically update with interesting facts concerning the collected names, the history of the territory and the project statistics, some through paid advertising. We also gave two public lectures.

Results of Collecting of Toponyms

The data collection phase began on 1 October 2019 and is still running (the results of the first phase presented in the article are valid as of 30 September 2020). Especially at the beginning of the first phase, the number of the names was increasing by hundreds a day (the first thousand names were added during the first three days). In February 2020, we suspended the collecting and manually corrected and cleaned the database. We decided to take this measure when incorrect input of information by some respondents was revealed (duplicate objects and names, incorrect location, intentional damage to the database, etc.). During the correction process, we also unified the written form of the names according to the rules of Czech spelling (especially capital letters). We usually kept the spelling of names containing foreign lexical units in their authentic form (e.g., *Kajlák*, from German *Keilsberg*). Due to a significant decrease in the number of the names added, corrections are now being made instantly.

As the result of the manual data correction, there are 2646 toponyms for 1614 objects. In total, 631 objects have more than one name, while the highest number of the names (13) was added to the public transport terminal in the city center. As expected, the named objects are located mainly in the inner city (ca. 80% of the names), with higher density in places of increased concentration of people, especially the city center and other local centers, such as the university dormitory (Figure 4).

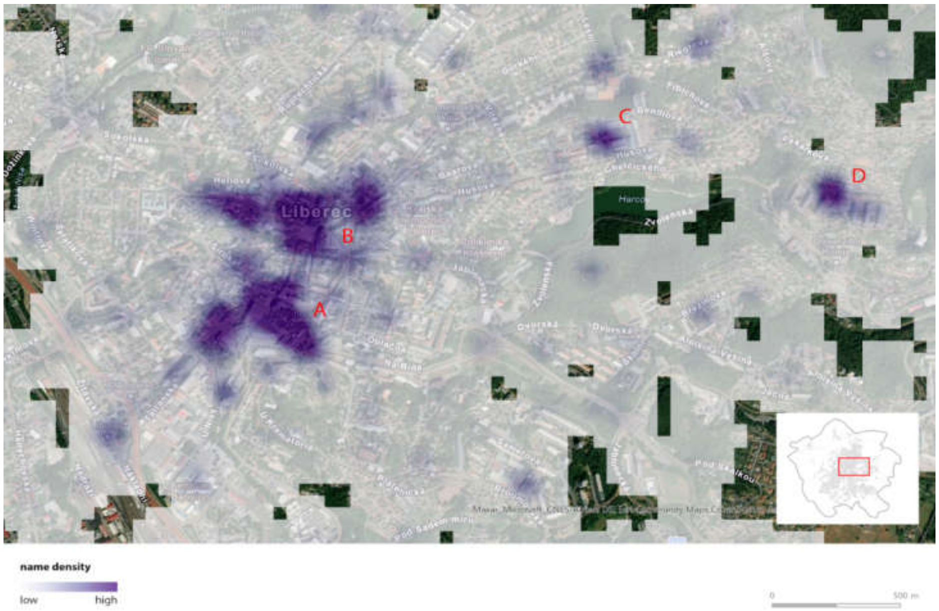

Figure 4. The highest concentration of names is in the area of the city center (clusters A—around the public transport terminal, "lower city center", and B—around the town hall, "upper city center"). Another significant cluster is the university campus (C) and the university dormitory (D). Method used: Kernel Density, 100 m.

Of the 584 registered respondents, 315 (54%) actively contributed and approximately 223 respondents were anonymous. The exact number of anonymous respondents is not known, however, it is possible to distinguish the names added in the database by an anonymous respondent during one visit to the web application so, in fact, the number of anon-

ymous users may be lower. Approximately two thirds of the registered respondents entered their age and other demographic information. The age range of those respondents is 17 to 79, the median is 39 years, and the median length of stay in the territory is 31 years (ranging between 1 and 74).

5. Phase 2—Methodology of Determining the Knowledge of the Toponyms Collected

Without information on the extent to which the names collected in the first phase are known among the inhabitants of the studied area, the informative value of the names would be rather low. In the database, beside the names widely known and used, there may also be names known only to a narrow group of people. For various reasons, there may be completely fictional names which we were unable to reveal (negative motivators were dealt with, for example, by Coleman et al. [41]). So in the second phase of the project, we ask respondents to choose one of the following options:

- I know and use the name.
- I know the name, but I don’t use it.
- I don´t know the name.

In popular toponymy, it is no exception to use the same name to denote different objects in different places (for instance, “Myší díra” (“Mouse hole”)—a narrow part of a path, a dark and confined subway, and the like). Therefore, it is important to determine the knowledge of the name in combination with the object (place, location, etc.), in other words, to find out to what extent the name is used for the concrete object. Thus the position of the object in the map is displayed in the application as well.

Objects are displayed with all associated names in which the respondent has not indicated the knowledge yet. In order to achieve approximately the same number of answers to each name, even if some respondents do not respond to each name, objects are selected from the database at random. As already mentioned, the database on which the knowledge assessment application is based contains 2646 unique names.

Again, the layout of the application is as simple as possible (Figure 5). Compared to the application used for the first phase, the map field contains an overview map for easier localization of the name in the studied territory. Based on feedback from user testing, a new functionality was added to the application used in the second phase: browsing the names by city districts (see Figure 5, right bottom corner). The knowledge of a concrete name indicated by the respondent is automatically saved in the database, which is then confirmed by a green icon next to the place name. The indication of the knowledge has also been implemented in the application used for collecting toponyms, which currently mainly serves as a presentation of the project results.

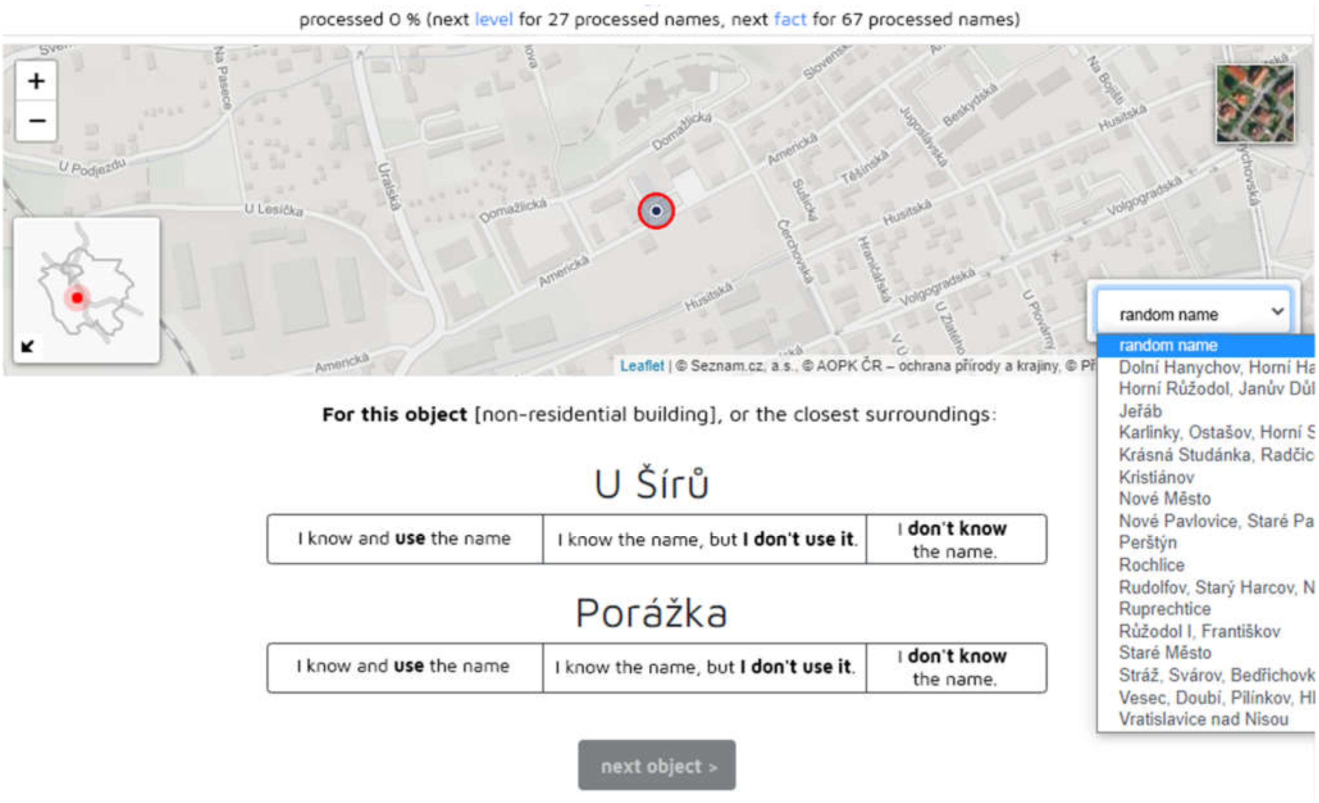

Figure 5. A part of the layout of the desktop version of the application, second phase. The heading and the bottom of the page is missing as it is the same as in the application used in the first phase. The application is developed in the Czech language. For the demo version, refer to https://mapy.fp.tul.cz/app/zj-demo/znalost/ (accessed on 5 May 2021).

Given the experience from the first phase and the positive reactions of the participating respondents, we assumed that the main motivators for participation in the second phase would be patriotism and developing respondents´ knowledge of the studied territory. In order to attract a larger number of respondents and increase the motivation to comment on as many place names as possible—ideally all of them, the application was supplemented with gamification elements. This step was necessary due to the large number of the names that emerged from the first phase and which resulted in a higher time required for their processing. There are numerous studies dealing with gamification in crowdsourcing projects [42–45] and, as reported by Morschheuser et al. [46], many of these studies show several positive effects (for instance, longer involvement, higher quality of results, and decrease in cheating). In the second phase of the project, four gamification elements were included, resulting from the recommendations in Morschheuser et al. [46]:

- An order number in the list of participants ordered by their activity, plus a hierarchic badge system—depending on the number of the names processed, the respondent can see his/her position in the list of respondents and strive for the next, higher position and a new badge. There are ten levels and the intervals between them are not linear. You can proceed faster at the beginning since you are motivated to go through a larger part of the database. The higher number of the names processed, the longer the interval between the levels. The logic behind this is that the people who find this process interesting at the beginning, will persist in doing it. In addition, the respondents who go through all the names are awarded with a certificate.
- Personal statistics—based on the answers, the respondents can see how many names and which parts of the territory they actually know.

- Interesting facts—after every 6% of the names processed, the application will show an interesting fact or facts about the toponyms or history. This is internal motivation, which stimulates curiosity [46].
- A contest—the respondents actively participating from 1 October to 31 December 2020 could participate in a raffle, where their chances of winning depended on their activity (the more processed names, the higher the chances of victory). Another condition for the participation in the raffle was processing at least 3% of the database content (ca. 100 names) and filling in the demographic data.

The initial promotion of the second phase took place by sending an information e-mail to all registered users in the first phase and using paid advertising on Facebook. In addition, we sent out a press release to the local media. Further promotion included paid advertising on Facebook and ongoing e-mail newsletters. Moreover, when respondents were inactive for more than two weeks, we automatically sent them reminders. As part of the newsletters, we asked respondents to fill in a survey, which, among other things, asked why they were involved in the project, what motivated them to go through all the names and, if they are no longer interested in the project, why they did not process all the names. Those who processed all the names were sent the survey by automatic e-mail at the end of their work.

Results of Determining the Knowledge of the Toponyms Collected

The phase of indicating the knowledge of the toponyms collected took place between 1 October 2020 and 11 January 2021. During this time, 927 respondents actively participated, of which 564 (60.8%) filled in demographic data. The age range of these respondents is 10–80 years, the median age is 39 years and the median length of stay in the territory is 30 years (range from 1 to 75 years). The age structure of the respondents corresponds to the age structure of the inhabitants of the studied territory. The majority (93.6%) of these respondents were living in Liberec at the time of the research, the other in municipalities in its immediate vicinity.

During the three months in which the second phase of the research took place, active respondents added more than 513 thousand answers. Including the answers from the first phase and user testing of the application, in total 530,738 answers are available, while the range of answers for one name ranges from 185 to 244 (median 196).

The level of knowledge within the whole set of 2646 collected popular toponyms is shown is illustrated in Figure 6. The data confirm our assumption that the names known to most respondents form a minority in absolute numbers and a large part of popular toponymy is related to various, especially geographically defined micro-social groups, cf. [47], [2]. Well-known names, in common communication indicating the main orientation dominants of the city, form the so-called toponymic centre (for the concept of the centre see [48], cf. [1]). Theoretically, the toponymic centre should be the names showing 100% knowledge. According to the second phase of the research, there are only four: *Fügnerka* (public transport terminal, derived from “Fügnerova street”), *Viadukt* / *U viaduktu* (“Viaduct”, a system of railway bridges separating two parts of the city), *Husovka* (derived from “Husova street”, one of the backbone streets of the city). If we took into account the limit of the toponymic centre at 81–100% knowledge, it would include 323 names (12.2% of the total number of 2646 collected names). At the object level, these are most often spatially or functionally significant buildings, less often streets and other public spaces [4], located mainly in the central part of the city. At the level of naming, these are most often simple deappelative names (*Viadukt* (“Viaduct”), *Radnice* (“Town hall”), and *Lázně* (“Bath”)) or deproprial names (*Globus* (shopping centre), *Baťa* (department store with a very distinctive design), *Lípa* (former cinema)). In popular urbanonymy, however, these names show a broader spatial fixation than the original name. Another important and frequent type of popular toponyms are derivatives from official urbanonymy, motivated by the effort for

brevity of expression (*Fügnerka* (derived from "Fügnerova street"), *Šalďák* (derived from "F. X. Šalda square"), *Husovka*).

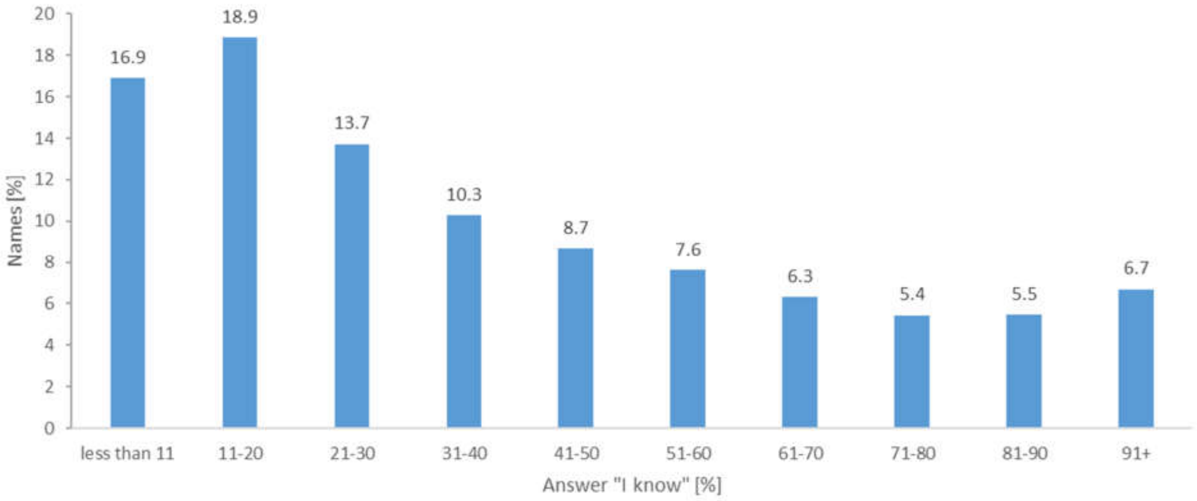

Figure 6. The level of knowledge of names (number of names = 2646).

The degree of the respondent involvement is similar to previous findings [49–51], i.e., most respondents processed only a few names; the larger the amount of the names, the smaller the number of respondents who reached it (see Figure 7). However, due to the random presentation of the names to the respondents, this different degree of participation did not significantly increase the variance in the number of responses to the name. An interesting aspect is the significant increase in the last interval, which includes 109 respondents who processed all the names.

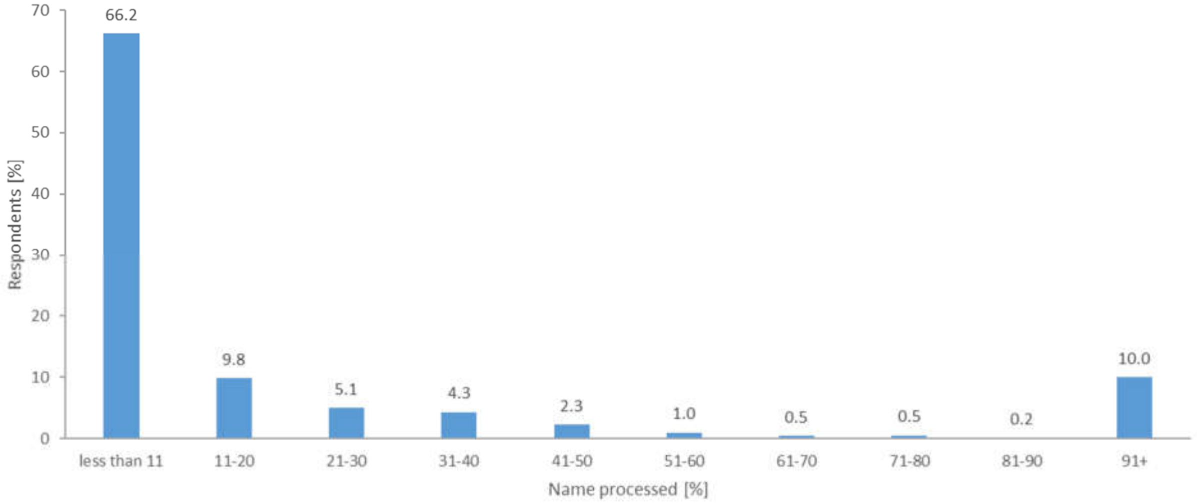

Figure 7. The degree of involvement of the respondents in the project. Only respondents who commented on at least one name are included (number of respondents = 927, number of names = 2646).

A possible explanation for the increase in the last interval can be found in the motivation of the respondents. Respondents could fill an additional survey asking, among other things, their motivation to start participating and why they continued or halted. We sent the survey to respondents in e-mail reminders, or when the respondent completed all names. We chose open answers to avoid guiding respondents into closed categories

and thus we could gain a broader range of reasons. We arranged the received answers into categories based on the modified method of [52]. At first, each of us read through all responses, than defined categories, and finally matched respondent's answers to those categories. Based on the respondent's statements of motivations, one response might fall into several categories. For example: "My motivation was to find out how many names I know and what different places are called", was considered as verification of knowledge (the first part) and curiosity (the second part). Next, we discussed our findings among ourselves and we determined nine categories (some categories were grouped, some categories were renamed).

Based on the data from the survey, mostly filled out by the respondents who processed all the names, the strongest motivators for participating in the project are patriotism, interest in history or in the region, efforts to learn something new, curiosity, altruism, and testing their knowledge (Table 1). Some of these reasons are the same as the motivation to continue in the project. In addition, there is an effort to finish the unfinished work, and the element of entertainment and game. Surprisingly, few respondents mentioned the contest or the reminders of the project received by e-mail as their motivation (only one respondent per each of these two motivators). However, the statistics of added responses over time show that the number of responses has increased after each bulk e-mail sent. In addition, it is possible to trace an increase in the number of responses from the respondent after sending an automatic notification of inactivity in the project. Therefore, we conclude that regular reminders of the project were important, although not mentioned in the responses. On the contrary, lack of time and too many names were mentioned as reasons for early termination of the participation in the project.

Table 1. Basic categories of motivation and their frequency in the second phase of the Living Names project. Some respondents participated for more than one reason, thus their answers are counted in several categories. Motivation in the meantime is calculated from respondents who processed the entire database (66% of respondents).

Motivation (Example)	at the Beginning (%) n=120	in the Meantime (%) n=79
patriotism ("This project delighted the heart of the Liberec citizen.")	23	5
general / professional interest in the topic ("I am interested in the history and present state of Liberec."/"My philological education and former interest in toponomastics.")	23	1
knowledge development ("It was interesting to browse and explore the popular place names."/"Presentation of interesting facts that gave me new angles of view of various places.")	20	22
curiosity ("What kind of project is this?"/"Curiosity related to names used for concrete places.")	16	19
altruism ("Using my knowledge to help the project.")	15	15
verification of knowledge ("I was wondering how many names I actually know.")	13	13
finishing the work	2	27

("When I get into something, I want to make it to the end.")		
fun<br>("It was adventurous and I liked it.")	0	15
game<br>("And the levels were motivating too.")	0	11

In Figure 8, we can see the degree of respondent's involvement in time, where results reflected our promotion and communication, i.e., the highest increases in the first and second phases clearly followed the press releases, paid advertising on social networks or newsletters and reminder e-mails. While the average number of collected daily responses was less than 8000 five days before publishing, the press release in the local newspaper, the average for the following five days was doubled. A similar increase was evident even after the announcement of the date of the planned end of the second phase. The peaks in the chart correspond to the dates of our communication with the respondents. The significant increase after 26 November 2020 was caused by the press release in the local newspaper, the increase on 4 January 2021 corresponds to the announcement of the end of the second phase.

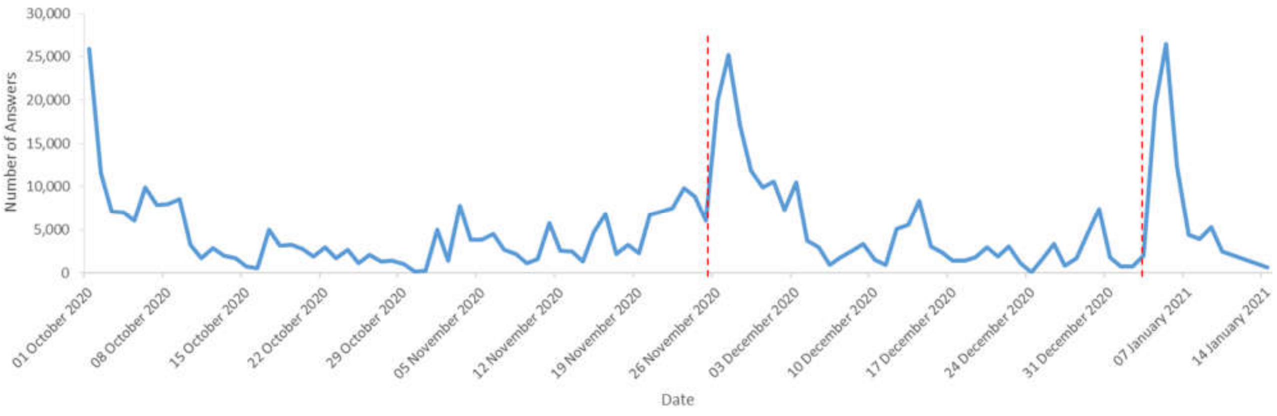

Figure 8. Numbers of answers added over time during the second phase of the project.

6. Discussion

The quantity, spatial distribution and accumulation of popular toponyms are based on the common orientation needs of the population, which cannot be fully saturated by the system of standardized street and place names. In addition, popular toponyms are an important bearer of the cultural and historical memory of the place. However, their collecting also has a practical use, for instance, for the needs of local authorities (as a reference base for determining new official terminology) or the integrated rescue system (as an aid in locating a caller who, when in a stressful situation, uses a popular place name to describe where he or she is at the moment). On the other hand, popular toponyms are variable and difficult to capture. As a solution, we propose to use crowdsourcing and thus involve a wide group of people in the particular territory. This ensures the collection of a large number of toponyms fixed primarily in oral use.

6.1. Main Findings

Involving over 900 respondents who added more than 2500 toponyms with over half a million individual responses concerning their knowledge proves that the project was

successful. The main factor is the discovery that popular toponymy as a topic is—similar to local history and interesting facts—attractive to the general public. The evidence is the speed with which the respondents were adding the names in the first phase or processing the names in the second phase. More important than the rate of filling the database, however, is the amount of information that was obtained by involving the public (historical facts, memories, etc.).

It is the theme that is the strongest motivator for involvement in this case. As confirmed by the results of the additional survey among respondents, patriotism, general interest in local knowledge and development of self-knowledge, i.e., internal or intrinsic motivators [53], were the most significant for the respondents who became more actively involved. It turned out that the right step was to include gamification elements into the application (badges, and personal statistics). On the contrary, for the respondents with higher involvement, the positive effect of including the competition was not directly proven and intrinsic motivators prevailed. These conclusions are the same as, for instance, the conclusions made by Zheng, Li, Hou [54]. We are aware that the resulting categorization of respondent's motivation may, in some cases, be subjective and thus debatable. Nevertheless, we believe that the result provides good insight into the respondents' motivation why they participated in the presented project.

The next factor is the usability of web applications. As the applications for both phases were designed to be as intuitive and as easy to use as possible, their use did not pose a problem even for people with limited digital skills. We think that the decision to create the responsive application to be usable also on mobile phones was correct. Although we did not track the amount of data added from different types of devices, according to Google Analytics data, the traffic to both applications was roughly the same from mobile devices as from desktop devices, which corresponds with current trends [55].

6.2. Implications for Future Practice

A well-chosen promotion and communication strategy, was proven to be a crucial precondition for the success of crowdsourcing based research. In our findings the degree of the respondent's involvement over the time reflected our promotion and communication strategy. This led the public to get involved in the project and kept them there as long as possible. Our suggestion is similar with [56], that communication is prerequisite before, during and after the project. This factor is highly responsible for the permanent interest of the respondents. However, to attract the widest and most demographically diverse group of respondents, promotion needs to utilize various information and news media (print × web × social).

The research in local toponymy is close to this and, as confirmed by the results of the second phase survey, patriotism, general interest in local knowledge and self-knowledge development, i.e., internal motivators, were most significant for the respondents who became more actively involved.

Together with [53,56] and resulting from communication with respondents, our suggestion is, that crowdsourcing projects should present learning opportunities (interesting facts or meetings with experts in the case of the Living Names project) and opportunities for respondents to interact with others (e.g., through social networks or directly in the application). Even if the positive effect of including the competition was not directly proven in the Living Names project, we believe that the competition makes sense, if not as motivator, then at least as the form of reward along with ladders, certificates, and badges [53]. Related to the Living Names project the question is whether the participants ever admitted that the competition was the motivator, and whether the competition was the motivator for the respondents who participated to a lesser degree.

6.3. Research Limitations and Possible Modification

The limitations of this research coincide with the limitations of other crowdsourcing projects [57–59]. The first weak point is seen in the number of respondents, their age structure and spatial distribution, which may not be sufficiently representative in the studied territory. One of the factors exacerbating this deficiency is the collecting process using digital technologies, the use of which may not be comfortable for all potential volunteers. The number and the structure of the respondents in the first phase can be considered as a boundary. However, this cannot be assessed objectively due to the collecting methodology since we cannot determine whether a particular respondent did not participate in the first phase because they no longer knew another name to add, or for another reason, or whether they participated as an anonymous person.

The second problem is the difficulty of data validation. In the case of collecting unstandardized names, it is not possible to determine to what extent the specific name is authentic or not. A clue could be the form of the name; that is, whether it has been formed in accordance with the general naming principles that are common in time and place. Therefore, it is necessary to approach not only the names with strong expressiveness or metaphoricalness, but also, for instance, those with unusual literacy or length, those with reserve. Even so, the assessment of the names seen from this angle is highly subjective. It is therefore necessary to validate the data in another way, by means of a subsequent analysis of the knowledge among the inhabitants of the territory, in this case made during the second phase of the project. However, even in this second phase, the results show a certain degree of uncertainty. It must be admitted that even here we cannot recognize whether the respondents indicated the knowledge randomly or whether they understood the task.

For data validation, we offer three possible methods for future research:

- The first option is to repeatedly display to the respondents the names they have already assessed and evaluate whether they are consistent in the answers. Unfortunately, this method may not be accurate since a name that respondents did not know the first time may seem familiar to them the second time and they may identify it as known but not used by them.
- In the second method, it would be possible to display to the respondents several totally fictitious but meaningful names. Respondents should not know them.
- The third method is based on the statistical analysis of respondents' answers. It is possible to compare whether a particular respondent often appears among those who know little known names (for instance, applying the Kendall´s tau method). Thus, it would be a matter of determining the degree of agreement between the respondents. In this way it would be possible to identify a respondent whose answers fall outside the group. However, determining the borderline at which the respondent's agreement with others is insufficient is very subjective. In addition, in the case of the low involvement of a particular respondent, the results of the statistical evaluation will not be accurate enough.

Revealing discrepancies in the data using the above methods would ultimately lead to the exclusion of all responses from a particular respondent. The question, however, is to what extent is it ever possible to validate the knowledge of popular toponyms? Such knowledge is very subjective. Although it is not possible to verify the correctness of the answers in the second phase, we believe that the number of responses reduces the mentioned degree of uncertainty and the results represent sufficiently informative material for further study. The resulting database should therefore not only contain a list of the names collected, but also an indication of their knowledge, including the number of the respondents, so that anyone and everyone can decide whether the result is valid for them or not.

The third limitation of this research is the collection of only point data, although toponyms are generally associated with not only with objects that can be represented by a point, but also with line or area objects. This can lead to the uncertainty of the respondents in determining their knowledge of the names (for instance, the respondent perceives the

name as a line object, while in the application it is represented as a point). The reason for the use of points is discussed in the introduction to Section 4. The solution seems to be to determine the object fixation of toponyms [60], i.e., to find out in which space is the particular name perceived. The intersection of these spaces forms the so-called core of the object fixation, that is, the common space described by the name or names, and it is then possible to spatially connect the names to the core of the object fixation.

One possible proposal for the project modification is to combine the first and second phases into one. This could be a good option, for instance, for local authorities that would like to determine new official names within a certain territory. In this way, the time required for collecting would be halved. However, this approach makes it difficult to determine the knowledge of the names collected, and the result may be more uncertain, for instance, because there will be more responses to the names added earlier than to those added later.

7. Conclusions

This article describes the Living Names project, its methodology, and its results. The aim of the project is not only to collect unstandardized toponyms in the studied territory by means of crowdsourcing, but also—and this is how this project differs from research projects referenced in Section 2—to determine the level of their knowledge among respondents. Based on the amount of the data collected, the number of the volunteers involved, and the feedback we received from them, we consider this project a success. The results of the project show that crowdsourcing is a legitimate method of collecting information about non-standardized toponyms. The success of the presented project lies with its interesting theme which combines historical information, memories of local inhabitants and fun in case of some creative toponyms. Next, it is the promotional strategy (press releases, social network advertisement, and reminder e-mails) to which the public responded with increased involvement. Lastly, it is the architecture of web applications, which follows the current UI and UX trends and implements gamification elements such as badges or showing interesting facts based on the respondent's involvement.

Despite the overall success of the project we are aware of some limitations. With regard to the replicability of this research, we also describe its limitations. These include the use of only a digital method of data collecting, difficult validation, and the correlation between the names displayed in the form of a point representation to the space they designate.

The results of collecting popular toponyms can be used in tourism (for instance, in unconventional guidebooks) or in the city administration. The names collected by respondents can serve as a basis for naming new residential units, streets in the suburbs and the like. The fact that people who associated with a place can participate in the process of naming that place can actually make it easier for people to accept such a name. Thus, the article can also be used as a guide for data collecting of a similar type. The resulting database is also used in the operation centre of integrated rescue systems as an aid in locating callers who, in a stressful situation, may use an unofficial place name, which they use more often than the official name, or don't even know the official name at all.

Future research should be focused on confirming our results in different places (in more or less populated cities). The scope and structure of the collected material in both phases provide a solid basis for onomastic analyses, focused primarily on naming strategies applied in popular nomenclature, and for geographical studies, focused on, for instance, the distribution of the toponyms in a given space. Due to the results of the second phase, we can also analyze the relationship of the respondents (their age, length of stay, and residence) to the toponyms. The future research question concerns the object fixation of selected toponyms: what space does the name represent? Is this representation the same for all inhabitants?

We suppose that it is not possible to collect all living names. As explained by Lynch [6], every individual creates their own image (even the toponymic one) of the space

around them, so we would have to ask everyone. In addition, popular toponymy includes a more or less stable core, but a large part of it varies over time and new names can be created every day. This is also confirmed by our experience, as during the second phase of the project the respondents added another approximately 350 valid new names or Lábus variations. The collected names are available at https://mapy.fp.tul.cz/app/zj-demo/ (accessed on 5 May 2021).

Author Contributions: Conceptualization, Daniel Vrbík and Václav Lábus; methodology, Daniel Vrbík and Václav Lábus; software, Daniel Vrbík; formal analysis, Daniel Vrbík and Václav Lábus; writing—original draft preparation, Daniel Vrbík and Václav Lábus; writing—review and editing, Daniel Vrbík and Václav Lábus; visualization, Daniel Vrbík. All authors have read and agreed to the published version of the manuscript.

Funding: This research received no external funding.

Institutional Review Board Statement: Not applicable.

Informed Consent Statement: Not applicable.

Data Availability Statement: The data presented in this study are available at http://mapy.fp.tul.cz/app/zivajmena/opendata/ziva_jmena_vysledky_opendata.csv (accessed on 5 May 2021).

Acknowledgments: We would like to thank to all involved respondents.

Conflicts of Interest: The authors declare no conflict of interest.

References

1. Pires, M. Investigating Non-Universal Popular Urban Toponyms. *Onoma* **2007**, 131–154, doi:10.2143/ONO.42.0.2047078.
2. Balode, L. Unofficial Urbanonyms of Latvia: Tendencies of Derivation. In *Names and Their Environment, Proceedings of the 25th International Congress of Onomastic Sciences, Glasgow, 25–29 August 2014*; Hough, C., Izdebska, D., Eds.; University of Glasgow: Glasgow, UK, 2016; Volume 1, pp. 69–79.
3. Český Statistický úřad: Veřejná Databáze. Available online: https://vdb.czso.cz/vdbvo2/faces/cs/index.jsf?page=profil-uzemi&uzemiprofil=31588&u=__VUZEMI__43__563889# (accessed on 1 March 2021).
4. Tom, A.; Denis, M. Language and Spatial Cognition: Comparing the Roles of Landmarks and Street Names in Route Instructions. *Appl. Cognit. Psychol.* **2004**, *18*, 1213–1230, doi:10.1002/acp.1045.
5. David, J.; Mácha, P. *Názvy Míst. Paměť, Identita, Kulturní Dědictví*; Host: Brno, Czech Republic, University of Ostrava: Ostrava, Czech Republic, 2014; pp. 71–72.
6. Lynch, K. *The Image of the City*; The MIT Press: Cambridge, MA, USA, 1960.
7. Ainiala, T. Names in Society. In *The Oxford Handbook of Names and Naming*; Hough, C., Ed.; Oxford University Press: Oxford, UK, 2016, p. 373.
8. Dunn, C.E. Participatory GIS—a People's GIS? *Prog. Hum. Geogr.* **2007**, *31*, 616–637, doi:10.1177/0309132507081493.
9. Goodchild, M.F. Citizens as Sensors: The World of Volunteered Geography. *GeoJournal* **2007**, *69*, 211–221, doi:10.1007/s10708-007-9111-y.
10. Sieber, R.; Robinson, P.; Johnson, P.; Corbett, J. Doing Public Participation on the Geospatial Web. *Ann. Am. Assoc. Geogr.* **2016**, *106*, 1–17, doi:10.1080/24694452.2016.1191325.
11. Crooks, A.; Hudson-Smith, A.; Croitoru, A.; Stefanidis, A. The evolving GeoWeb. In *GeoComputation*; CRC Press: Boca Raton, FL, USA, 2014; pp. 69–96. ISBN 978-1-4665-0328-1.
12. Hogerwerf, J. Toponymic data and map production in the Netherlands: from field work to crowd sourcing. In *Eleventh United Nations Conference on the Standardization of Geographical Names*; UNGEGN: New York, USA, 2018; E/CONF.105/87/CRP.87.
13. Rampl, G. Crowdsourcing and GIS-Based Methods in a Field Name Survey in Tyrol. 2014, pp. 1–7. Available online: https://unstats.un.org/unsd/geoinfo/UNGEGN/docs/28th-gegn-docs/WP/WP16_Field%20name%20survey%20in%20Tyrol.pdf (accessed on 1 March 2021).
14. Castellote, J.; Huerta Guijarro, J.; Pescador, J.; Brown, M. Towns Conquer: A Gamified Application to Collect Geographical Names (vernacular names/toponyms). In Proceedings of the AGILE International Conference, Leuven, Belgium, 14–17 May 2013. Available online: https://nottingham-repository.worktribe.com/output/1003559/towns-conquer-a-gamified-application-to-collect-geographical-names-vernacular-namestoponyms (accessed on 1 March 2021).
15. Názvy Míst. Available online: https://www.nazvymist.cz/ (accessed on 1 March 2021).
16. Southall, H.; Aucott, P.; Fleet, C.; Pert, T.; Stoner, M. GB1900: Engaging the Public in Very Large Scale Gazetteer Construction from the Ordnance Survey "County Series" 1:10,560 Mapping of Great Britain. *J. Map Geogr. Libr.* **2017**, *13*, 7–28, doi:10.1080/15420353.2017.1307305.

17. Perdana, A.; Ostermann, F. A Citizen Science Approach for Collecting Toponyms. *ISPRS Int. J. of Geo Inf.* **2018**, *7*, 222, doi:10.3390/ijgi7060222.
18. Pánek, J. From Mental Maps to GeoParticipation. *Cartogr. J.* **2016**, *53*, 300–307, doi:10.1080/00087041.2016.1243862.
19. Waze Mobile. Available online: https://www.waze.com/cs/ (accessed on 1 March 2021).
20. SETI@home. Available online: https://setiathome.berkeley.edu (accessed on 1 March 2021).
21. Pánek, J.; Ivan, I.; Macková, L. Comparing Residents' Fear of Crime with Recorded Crime Data—Case Study of Ostrava, Czech Republic. *ISPRS Int. J. of Geo Inf.* **2019**, *8*, 401, doi:10.3390/ijgi8090401.
22. Olteanu-Raimond, A.-M.; Hart, G.; Foody, G.M.; Touya, G.; Kellenberger, T.; Demetriou, D. The Scale of VGI in Map Production: A Perspective on European National Mapping Agencies. *Trans. GIS* **2017**, *21*, 74–90, doi.org/10.1111/tgis.12189.
23. Howe, J. The Rise of Crowdsourcing. *WIRED*, 6 June 2006, pp. 1–4.
24. Haklay, M. Geographic citizen science: An overview. In *Geographic Citizen Science Design: No One Left Behind*; Skarlatidou, A., Haklay, M., Eds.; UCL Press: London, UK, 2021; doi:10.14324/111.9781787356122, pp. 15–37.
25. Goodchild, M.F.; Glennon, J.A. Crowdsourcing geographic information for disaster response: A research frontier. *Int. J. Digit. Earth* **2010**, *3*, 231–241.
26. Cartwright, W. Neocartography: Opportunities, Issues and Prospects. *S. Afr. J. Geomat.* **2012**, *1*, 14–31.
27. Surowiecki, J. *The Wisdom of Crowds*; Anchor: New York, NY, USA, 2004, ISBN 978-0385721707.
28. Haklay, M. Citizen Science and volunteered geographic information: Overview and typology of participation. In *Crowdsourcing Geographic Knowledge: Volunteered Geographic Information (VGI) in Theory and Practice*; Sui, D.Z., Elwood, S., Goodchild, M.F., Eds.; Springer: New York, NY, USA, 2013; ISBN 978-94-007-4586-5.
29. UNGEGN. *The Benefits of Geographical Names Standardization*; UNGGN: New York, NY, USA, 2015; pp. 1–39.
30. Ordnance Survey Press Office. Do You Know Where to Find Your Nuncle Dicks or Your Deadman's Head? Available online: https://www.ordnancesurvey.co.uk/newsroom/blog/know-find-nuncle-dicks-deadmans-head (accessed on 13 April 2021).
31. Geospatial World. OS Embarks on a Vernacular Geography Project. Available online: https://www.geospatial-world.net/news/os-embarks-on-a-vernacular-geography-project/ (accessed on 13 April 2021).
32. Shetland Amenity Trust. Shetland Place Names Project. Available online: https://www.shetlandamenity.org/shetland-place-names-project (accessed on 13 April 2021).
33. Meitheal Logainm.Ie. Available online: https://meitheal.logainm.ie/en/ (accessed on 13 April 2021).
34. Šrámek, R. *Úvod do Obecné Onomastiky*; Masaryk University: Brno, Czech Republic, 1999.
35. Český Statistický Úřad: Informační Společnost v Číslech. 2020. Available online: https://www.czso.cz/documents/10180/122362632/06100420.pdf/1273f88b-7e14-4555-b58b-3087658409e0?version=1.4 (accessed on 1 March 2021).
36. Brown, G.; Pullar, D.V. An evaluation of the use of points versus polygons in public participation geographic information systems using quasi-experimental design and Monte Carlo simulation. *Int. J. Geogr. Inf. Sci.* **2012**, *26*, 231–246.
37. Wikimedia Foundation. Editor Survey Report—April 2011. Available online: https://wikimediafoundation.org/w/index.php?title=File%3AEditor_Survey_Report_-_April_2011.pdf&page=1 (accessed on 1 March 2021).
38. SimilarWeb 2020. Available online: https://www.similarweb.com/top-websites/czech-republic/category/reference-materials/maps/ (accessed on 1 March 2021).
39. Jay, C.; Dunne, R.; Gelsthorpe, D.; Vigo, M. To Sign Up, or Not to Sign Up? Maximizing Citizen Science Contribution Rates through Optional Registration. In *Proceedings of the 2016 CHI Conference on Human Factors in Computing Systems 7–12 May 2016*; ACM: San Jose, CA, USA, 2016; pp. 1827–1832.
40. Jackson, C.B.; Crowston, K.; Østerlund, C. Did They Login?: Patterns of Anonymous Contributions in Online Communities. *Proc. ACM Hum. Comput. Interact.* **2018**, *2*, 1–16, doi:10.1145/3274346.
41. Coleman, D.; Georgiadou, Y. Volunteered Geographic Information: The Nature and Motivation of Produsers. *Int. J. Spat. Data Infrastruct. Res.* **2009**, 332–358, doi:10.2902/1725-0463.2009.04.art16.
42. Eveleigh, A.; Jennett, C.; Lynn, S.; Cox, A.L. "I Want to Be a Captain! I Want to Be a Captain!": Gamification in the Old Weather Citizen Science Project. *In Proceedings of the First International Conference on Gameful Design, Research, and Applications 2–4 October 2013*; ACM: Toronto, ON, Canada, 2013; pp. 79–82, doi:10.1145/2583008.2583019.
43. Zeng, Z.; Tang, J.; Wang, T. Motivation Mechanism of Gamification in Crowdsourcing Projects. *J. Conserv. Sci.* **2017**, *1*, 71–82, doi:10.1108/IJCS-12-2016-0001.
44. Bowser, A.; Hansen, D.; He, Y.; Boston, C.; Reid, M.; Gunnell, L.; Preece, J. Using Gamification to Inspire New Citizen Science Volunteers. In *Proceedings of the First International Conference on Gameful Design, Research, and Applications 2–4 October 2013*; ACM: Toronto, ON Canada, 2013; pp. 18–25.
45. Hamari, J.; Koivisto, J.; Sarsa, H. Does Gamification Work?—A Literature Review of Empirical Studies on Gamification. In *Proceedings of the 2014 47th Hawaii International Conference on System Sciences 6–9 January 2014*; IEEE: Waikoloa, HI, USA, 2014; pp. 3025–3034.
46. Morschheuser, B.; Hamari, J.; Koivisto, J. Gamification in Crowdsourcing: A Review. In *Proceedings of the 2016 49th Hawaii International Conference on System Sciences 5–8 January 2016*; IEEE: Koloa, HI, USA, 2016; pp. 4375–4384.
47. Krško, J. *Všeobecnolingvistické Aspekty Onymie*. Matej Bel University: Banská Bystrica, Slovakia, 2016.
48. Lábus, V.; Vrbík, D. *Toponyma v Krajině a Možnosti Jejich Výzkumu*. Technical University of Liberec: Liberec, Czech Republic, 2018.

49. Nielsen, J. The 90-9-1 Rule for Participation Inequality in Social Media and Online Communities. *Nielsen Norman Group Evidence-Based User Experience Research, Training, and Consulting*. 9 September 2006. Available online: https://www.nngroup.com/articles/participation-inequality/ (accessed on 1 March 2021).
50. Vrbík, D. Crowdsourcing of Local Spatial and Historical Knowledge. *GI_Forum* **2016**, *2*, 109–122, doi:10.1553/giscience2016_02_s109.
51. Zachte, E. Distribution of Article Edits over Registered Editors. *Wikipedia Statistics—Tables—English*. 12 October 2017. Available online: https://stats.wikimedia.org/EN/TablesWikipediaEN.htm (accessed on 25 October 2017).
52. Raddick, M.J.; Bracey, G.; Gay, P.L.; Lintott, Ch. J.; Murray, P.; Schawinski, K.; Szalay, A.S.; Vandenberg, J. Galaxy Zoo: Exploring the Motivations of Citizen Science Volunteers. *Astron. Educ. Rev.* **2010**, *9*, 1, doi:10.3847/AER2009036.
53. Lotfian, M.; Ingensand, J.; Brovelli, M.A. A Framework for Classifying Participant Motivation That Considers the Typology of Citizen Science Projects. *ISPRS Int. J. Geo Inf.* **2020**, *9*, 704, doi:10.3390/ijgi9120704.
54. Zheng, H.; Li, D.; Hou, W. Task Design, Motivation, and Participation in Crowdsourcing Contests. *Int. J. Electron. Commer.* **2011**, *15*, 57–88, doi:10.2753/JEC1086-4415150402.
55. Enge, E. Mobile vs. Desktop Usage in 2019. 11 April 2019. Available online: https://www.perficient.com/insights/research-hub/mobile-vs-desktop-usage-study (accessed on 1 March 2021).
56. Moor, T.D.; Rijpma, A.; López, M.P. Dynamics of Engagement in Citizen Science: Results from the "Yes, I Do!"-Project. *Citiz. Sci. Theory Pract.* **2019**, *4*, 38, doi:10.5334/cstp.212.
57. Goodchild, M.F. Putting research into practise. In *Quality Aspects of Spatial Data Mining*; Stein, A., Shi, W., Bijker, W., Eds.; CRC Press: Boca Raton, FL, USA, 2009, pp. 345–356.
58. Haklay, M. How Good Is Volunteered Geographical Information? A Comparative Study of OpenStreetMap and Ordnance Survey Datasets. *Environ. Plan. B Plan. Des.* **2010**, *37*, 682–703, doi:10.1068/b35097.
59. Koukoletsos, T. A Framework for Quality Evaluation of VGI Linear Datasets. Ph.D. Thesis, University College London, London, UK, 2012. Available online: http://discovery.ucl.ac.uk/1359907/11/1359907.pdf (accessed on 6 August 2017).
60. Lábus, V.; Vrbík, D. Poznámky k tzv. objektové fixaci toponym. *Linguist. Brun.* **2019**, 15–26, doi:10.5817/LB2019-1-2.
