# Supplementary material for: Estimation of Undetected Asymptomatic COVID-19 Cases in South Korea Using a Probabilistic Model
Source: Int J Environ Res Public Health. 2021 May 6;18(9):4946. doi: 10.3390/ijerph18094946 (PMC8124955; doi:10.3390/ijerph18094946)
Supplement: Supplementary file 1 [file ijerph-18-04946-s001.zip › 5/ijgi-1178642.pdf]

International Journal of *Geo-Information*

MDPI

Article

Temporal and Spatial Evolution and Influencing Factors of Public Sentiment in Natural Disasters—A Case Study of Typhoon Haiyan

Ting Zhang [1,2,3] and Changxiu Cheng [1,2,3,4,*]

[1] Key Laboratory of Environmental Change and Natural Disaster, Beijing Normal University, Beijing 100875, China; zhangt@mail.bnu.edu.cn
[2] State Key Laboratory of Earth Surface Processes and Resource Ecology, Beijing Normal University, Beijing 100875, China
[3] Faculty of Geographical Science, Center for Geodata and Analysis, Beijing Normal University, Beijing 100875, China
[4] National Tibetan Plateau Data Center, Beijing 100101, China
* Correspondence: chengcx@bnu.edu.cn

Abstract: The public's attitudes, emotions, and opinions reflect the state of society to a certain extent. Understanding the state and trends of public sentiment and effectively guiding the direction of sentiment are essential for maintaining social stability during disasters. Social media data have become the most effective resource for studying public sentiment. The TextBlob tool is used to calculate the sentiment value of tweets, and this research analyzed the public's sentiment state during Typhoon Haiyan, used the biterm topic model (BTM) to classify topics, explored the changing process of public discussion topics at different stages during the disaster, and analyzed the differences in people's discussion content under different sentiments. We also analyzed the spatial pattern of sentiment and quantitatively explored the influencing factors of the sentiment spatial differences. The results showed that the overall public sentiment during Typhoon Haiyan tended to be positive, that compared with positive tweets, negative tweets contained more serious disaster information and more urgent demand information, and that the number of tweets, population, and the proportion of the young and middle-aged populations were the dominant factors in the sentiment spatial differences.

Keywords: natural disasters; sentiment analysis; topic classification; temporal and spatial evolution; factor detection; interactive detection

Citation: Zhang, T.; Cheng, C. Temporal and Spatial Evolution and Influencing Factors of Public Sentiment in Natural Disasters—A Case Study of Typhoon Haiyan. *ISPRS Int. J. Geo-Inf.* **2021**, *10*, 299. https://doi.org/10.3390/ijgi10050299

Academic Editors: Wolfgang Kainz and Yeran Sun

Received: 26 March 2021
Accepted: 30 April 2021
Published: 5 May 2021

Publisher's Note: MDPI stays neutral with regard to jurisdictional claims in published maps and institutional affiliations.

Copyright: © 2021 by the authors. Licensee MDPI, Basel, Switzerland. This article is an open access article distributed under the terms and conditions of the Creative Commons Attribution (CC BY) license (http://creativecommons.org/licenses/by/4.0/).

1. Introduction

When a natural disaster suddenly occurs, panic emotions will quickly spread among the crowd, which will affect not only the stability of society, but also the emergency management of disasters. The public's attitudes, emotions, and opinions reflect the state of society to a certain extent [1,2]. Understanding the state, trend, and abnormal changes in public sentiment will help rescue agencies make corresponding rescue decisions and coordinate disaster emergency management [3].

The widespread use of social media provides a new way to obtain disaster information, and the comments released by affected people become the first-hand information of the disaster [4–7]. People in the affected areas use social media to keep in touch with the outside world, discuss the disaster, request assistance, and receive notifications from relevant organizations during the disaster [8,9]. Scholars have conducted extensive research on disasters based on social media[10], such as disaster situation awareness and early warnings [11–13], disaster loss assessment [14–16], and information mining [17–19].

Social media information contains public attitudes, emotions, and opinions, which makes it feasible to track public responses [17].

It is a challenge to mine disaster-related information from massive, unstructured social media data. Topic models are widely used in social media information mining. Imran et al. [20] developed an artificial intelligence disaster response platform (AIDR), which combined manual supervision and machine learning, and trained an automatic classifier for nine topics related to disasters. This was successfully tested during the Pakistan earthquake. Purohit et al. [21] convert tweets into semi-structured text, and then use supervised machine learning methods to classify the text, identify relevant texts for disaster relief needs, and match them with demand providers. In supply and demand matching, their method is 72% higher than matching only using text similarity. Wan et al. [22] used latent dirichlet allocation (LDA) and support vector machine (SVM) to classify related Sina Weibo during the "July 21" heavy rain in Beijing, and obtained 6 topics. He identified and located the emergency information in the real-time text stream, and put forward the principles for the distribution of emergency supplies. Zhang et al. [23] used BTM to classify tweets during Typhoon Haiyan, identify demand-related tweets, and analysis the demand of relief supplies, which is conducive to making timely decisions in the event of a disaster. Ligutom et al. [24] used BTM to qualitatively analyze typhoon-related tweets, used open coding especially to evaluate the results, and found differences in the behavior of Filipinos during the typhoon.

Sentiment analysis is used to identify positive and negative opinions, emotions, and evaluations expressed in natural language [25]. It is very important to analyze the sentiment of social media information to understand the views expressed by the public [26]. In recent years, many scholars have used sentiment analysis for disaster research. Neppalli et al. [27] performed a sentiment analysis of tweets posted on Twitter during the disastrous Hurricane Sandy and visualized online users' sentiment on a geographical map to indicate that people's sentiment changes according to the user's location and the distance from the hurricane center. Nagy and Stamberger [28] combined sentiment dictionaries and emojis to perform sentiment detection on tweets during the 2010 natural gas explosion and fire in San Bruno, California. The results show that the accuracy of this method is 27% higher than that of Bayesian network alone. Schulz et al. [29] proposed a fine-grained sentiment analysis method to analyze Hurricane Sandy-related microblogs by dividing people's emotions into the six categories of "anger, disgust, fear, happiness, sadness, and surprise". Most disaster-related sentiment analysis studies are more concerned with the description of sentiment states and lack an exploration of the causes and the influencing factors of sentiment. Our research focuses on four questions as follows.

(1) How do the temporal and spatial patterns of public sentiment evolve after the disaster?

(2) How does the content of the public discussion change at different stages of the disaster?

(3) What are the differences in public discussion topics under different sentiments?

(4) What factors influence the spatial differences in public sentiment?

To address these four questions, we first use sentiment analysis methods to characterize public sentiment from the perspective of time and space. Second, we explore the topics of public discussion under different sentiments through topic classification. Finally, we use geographic detectors to quantitatively analyze the influencing factors of public sentiment according to spatial differences.

2. Typhoon Haiyan

This research chose Typhoon Haiyan (or Yolanda locally) as case study. On the one hand, it is the strongest typhoon ever recorded in the northwest Pacific Ocean. At 4:40 a.m. on November 8, 2013, Typhoon Haiyan landed in Samar Province and hit the provinces and cities in the central Philippines. It caused considerable economic losses and casualties and resulted in 6,300 deaths, 28,688 injuries, more than 3 million families being

affected, and economic losses exceeding 1.9 billion US dollars [30]. On the other hand, The Philippines has one of the largest shares of Twitter users in the world. During Typhoon Haiyan, people published many comments about it on Twitter, providing sufficient data for research. Figure 1 shows the path and the public storm warning signal of Haiyan.

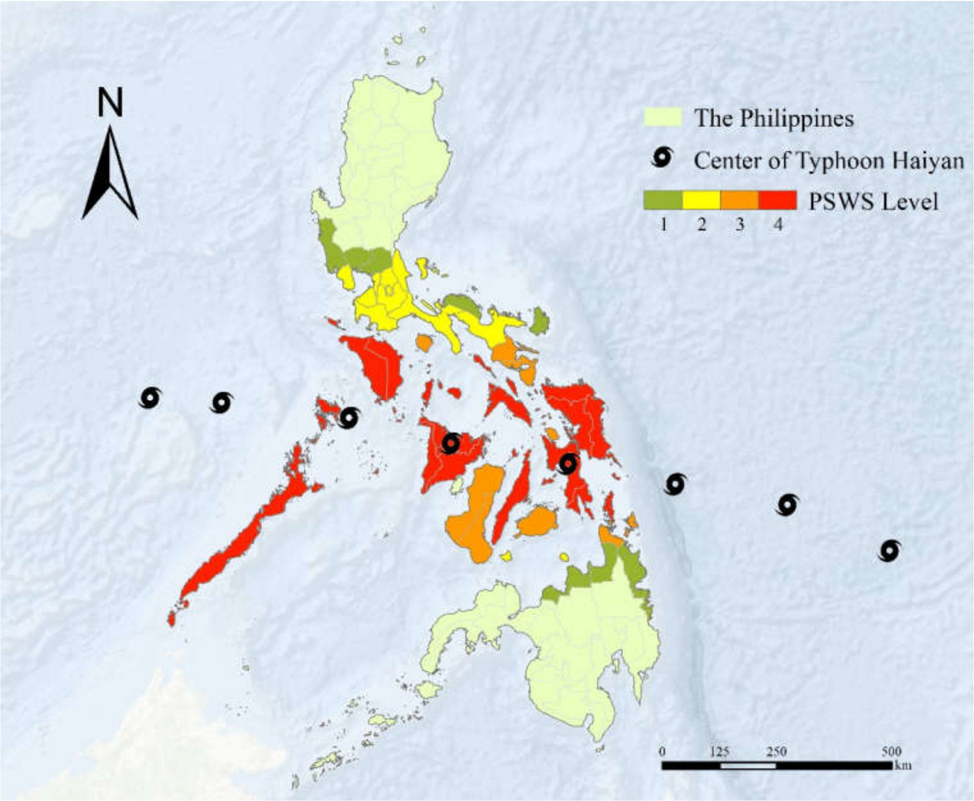

Figure 1. The path and Public Storm Warning Signal (PSWS) of Typhoon Haiyan in the Philippines. The larger the PSWS value, the higher the warning level.

3. Method

The research process of the temporal and spatial analysis of public sentiment and its influencing factors is provided in Figure 2. First, we collect tweets related to disasters and preprocess them. Then, we calculate the sentiment value of the tweets and divide the sentiment polarity of the tweets based on the TextbBlob. Finally, a temporal and spatial analysis of public sentiment was conducted. Topic classification is used to explore the evolution of public topics and analyze the reasons for the temporal changes in public sentiment based on the evolution of topics. Moreover, we use a geographic detector to explore the factors that affect the spatial distribution of public sentiment.

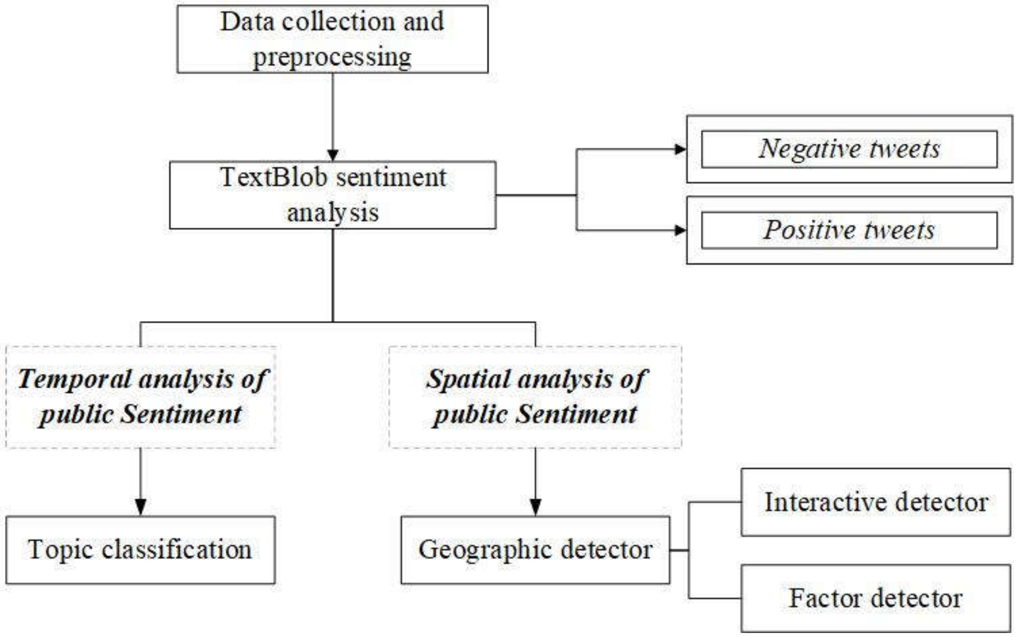

Figure 2. Research framework and workflow describing tweet selection, sentiment analysis, and temporal and spatial analyses.

3.1. Data Collection and Twitter Data Preprocessing

Historical tweets from October to December 2013 were downloaded from the 1% tweets in the Internet archive database (https://archive.org/ ,accessed on 20 May 2018). Using the hashtag filtering method proposed by Nikita and Cheng [31], through five layers of filters (time screening, spatial screening, frequency screening, expert screening, and an Support Vector Machines), 21 hashtags related to Typhoon Haiyan were obtained (Table A1). Based on the resultant hashtags, a web crawler program retrieved relevant social media datasets by screening hashtags within the disaster. Finally, 67,355 original tweets (excluding retweets) from November 2, 2013 to November 30, 2013 were obtained.

To clean and normalize the data, the preprocessing steps for the texts include:

(1) Remove repeated texts.

(2) Segment texts.

(3) Remove nonalphabetic characters, stop words, low-frequency words (which appear only once), high-frequency nonrelated words (which appear more frequently than 1% of the total number of words), and hashtags.

(4) Remove low-quality texts (texts with only 0 or 1 word left after step 3).

In addition to the Twitter data, the socio-economic data of the Philippines and the disaster data of Typhoon Haiyan were also collected to analyze the factors that affect public sentiment during disasters. The detailed data and sources are shown in Table 1.

Table 1. Data sources and download links.

Data	Sources	Download Link
Affected population data	NDRRMC（National Disaster Risk Reduction and Management Council)	http://ndrrmc.gov.ph/attachments/article/1329/FINAL_REPORT_re_Effects_of_Typhoon_YOLANDA_HAIYAN_06-09NOV2013.pdf (accessed on 21 May 2018)
Population density data of kilometers grid in 2010	Socioeconomic Data and Applications Center (SEDAC)	http://sedac.ciesin.columbia.edu (accessed on 21 March 2020)

Functional literacy rate of the population 10-64 years old	Philippine Statistics Authority	https://psa.gov.ph/ (accessed on 21 March 2020)
Gross regional domestic product	Philippine Statistics Authority	https://psa.gov.ph/ (accessed on 21 March 2020)
Young and middle-aged population	Philippine Statistics Authority	https://psa.gov.ph/ (accessed on 21 March 2020)
Typhoon track and its impact area	IBTrACS（NCDC International Best Track Archive for the Climate Stewardship Project）	https://www.ncdc.noaa.gov/ibtracs/ (accessed on 20 May 2018)

3.2. Tweet Sentiment Analysis

Sentiment analysis is the process of identifying the user's subjective emotions, opinions and attitudes from the text [32]. The most important tasks of sentiment analysis are sentiment extraction, sentiment classification, sentiment retrieval, and reporting to decision makers. Sentiment analysis includes calculating the sentiment value of the tweets and the polarity division. Currently, many methods [33–37] can perform sentiment analysis. We use TextBlob [38] to calculate the sentiment value of a tweet. TextBlob is used in many sentiment analysis studies [39–41]. It is a Python library for processing textual data and provides an API for performing common natural language processing tasks such as part-of-speech tagging, sentiment analysis, classification, and more.

The sentiment value calculation of TextBlob is actually a dictionary-based sentiment analysis. Part of speech, negative words, adverbs of degree, punctuation marks and emoticons are comprehensively considered when calculating the sentiment value. For each tweet, the 'sentiment' property returns a named tuple of the form 'Sentiment (polarity, subjectivity)'. The polarity score represents the sentiment value of the tweet, reflecting the positive or negative degree of the tweet.

$$\text{polarity} = \frac{\sum_{i=0}^{K}(-0.5)^{n}\cdot S_{i\_ad}\cdot S_{punc} * S_{em}}{K} \tag{1}$$

$$S_{i\_ad} = MAX(-1, MIN(S_i * S_{ad}, 1)) \tag{2}$$

where K represents the number of sentiment words in a tweet, S_i represents the sentiment value of current sentiment word, S_{i_ad} represents the sentiment value of sentiment word with adverbs of degree, n is the number of negative words related to the sentiment word, S_{punc} is the sentiment value of punctuation mark, and em is the sentiment value of emoticons. MAX means to keep the maximum value, MIN means to keep the minimum value

It varies within the range [–1.0, 1.0], where -1.0 is very negative, and 1.0 is very positive. The subjectivity varies within the range [0.0, 1.0], where 0.0 is very objective, and 1.0 is very subjective. Sentiment analysis is a type of subjectivity analysis [42]. Therefore, in our research, we only analyzed the tweets with a subjectivity not equal to 0.

3.3. Tweet Topic Classification

The biterm topic model (BTM) is used to extract the topic of the tweet. It was proposed by Yan et al. [43] to overcome the shortcomings caused by short texts. The BTM directly models the word co-occurrence patterns from an unordered word-pair co-occurrence in a short context. The BTM solves document sparseness by learning topics from the aggregate pattern of the entire corpus.

Since the BTM is an unsupervised method, the number of topics is determined by the perplexity curve before modeling. We use the perplexity curve to infer the number of topics. When the perplexity is smaller, the model is better. A previous study [44] introduced the process of BTM model construction and the calculation of perplexity in detail.

Since there are tweets in different languages, language inconsistency will increase the difficulty of classification and understanding. Therefore, the non-English texts were

translated into English text before classification. After running the BTM, the topic-word probability distribution matrix is obtained. This matrix represents the probability of different words in a topic. In each topic, select the top 20 most frequent words, and then describe the specific meanings of each topic. Some topics may have similar meanings, so they can be manually grouped together.

3.4. Geographical Detector

The geographical detector, i.e., geodetector, is a spatial analysis model that detects the spatial differentiation of a phenomenon and its driving mechanism [45]. In disasters, the overall sentiment of a region is affected by many factors. We explored the driving factors of sentiment differences in different regions from the four aspects of society, economy, culture and current disaster situations, and selected 7 specific indicators (which are shown in Table 2) to represent these four aspects. The indicators in each factor are independent of each other and are representative. The factor detector and interactive detector in Geodetector are used for analysis.

Table 2. Seven specific indicators of four factors.

Factor	Specific indicators
Disaster factor	Number of tweets (TN)
	Distance to typhoon center (DIS)
	Number of people affected (AF)
Economy factor	Gross domestic product (GDP)
Culture factor	Literacy rate (LR)
Society factor	Young and middle-aged population ratio (YMR)
	Population (POP)

3.4.1. Factor Detector

Geodetector is a statistical tool to measure Stratified Heterogeneity (SH) and to explore the determinants of SH. The factor detector is used to measure and find SH among sentiment data, and to test the coupling between two variables Y and X, according to their SHs. Here, the q-statistic measures the degree of SH of a variable Y, and the determinant power of an explanatory variable X of Y, and is calculated as follows:

$$q = 1 - \frac{\sum_{h=1}^{L} N_h \sigma_h^2}{N\sigma^2} \quad (3)$$

where $h = 1, \ldots, L$ is the strata of attribute Y, N_h and N are the number of units in layer h and the entire area, respectively, and σ_h^2 and σ^2 are the variance of Y for layer h and the entire area, respectively. The range of q is [0,1], and when the value of q is larger, the ability of X to explain Y is stronger.

3.4.2. Interactive Detector

The interaction detector reveals whether the risk factors $X_1$and X_2 have an interactive influence on a response variable Y, that is, to evaluate whether factors X_1 and X_2 will increase or decrease the determinant power on Y when acting together or whether these factors are independent. Specific methods are as follows:

(1)Calculate the q values of two factors X_1 and X_2 to obtain $q(X_1)$ and $q(X_2)$.
(2) Superimpose the two layers X_1 and X_2 to get a new layer $X_1 \cap X_2$.
(3)Calculate the q value of the new layer $X_1 \cap X_2$ to obtain $q(X_1 \cap X_2)$.
(4)Compare $q(X_1)$ and $q(X_2)$ and $q(X_1 \cap X_2)$.

4. Results

Calculate the sentiment value of tweets in the Philippines during Typhoon Haiyan, and conduct a temporal and spatial analysis of public sentiment, which can be used to

reflect the trend of sentiment changes and the spatial distribution of sentiment among the Filipino public. For the trend of public sentiment changes, BTM is used to classify tweets to analyze the reasons for sentiment changes, and to analyze the topic differences between positive and negative tweets. For the difference in the distribution of public sentiment, the geographic detector is used to measure its influence factors.

4.1. Temporal and Spatial Analysis of Public Sentiment

4.1.1. The Temporal Evolution of Public Sentiment

We use TextBlob to calculate the sentiment value of each tweet from November 7, 2013 to November 15, 2013 and draw a time series graph, as shown in Figure 3. During Typhoon Haiyan, people's daily average value of sentiment on Twitter is greater than 0, that is, it is positive. November 7 is the highest point of sentiment (0.30). Before November 7 is the pre-disaster preparation stage. Various regions in the Philippines issued typhoon warning signals, and people had extensive discussions on Twitter regarding the defense and preparations for Typhoon Haiyan. On November 8, Typhoon Haiyan made landfall. After that, people were affected by Typhoon Haiyan, and their sentiment values began to decline until they reached their lowest point (0.11) on November 10. At 10:00 am on this day, the National Development and Reform Commission held a technical management team meeting and a press conference. Disaster relief was in full swing and people's sentiments began to elevate.

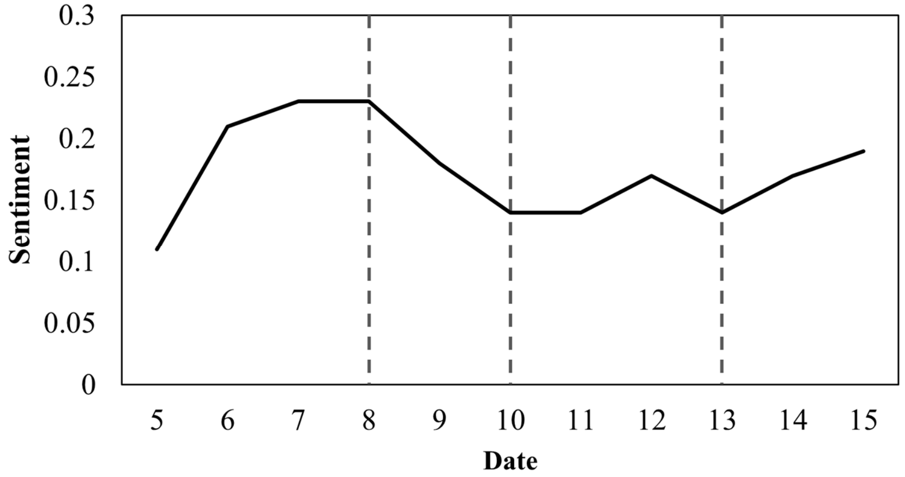

Figure 3. Time series of the daily mean value of public sentiment during Typhoon Haiyan from November 5, 2013 to November 15, 2013.

4.1.2. The Spatial Distribution of Public Sentiment

Tweets have the user's latitude and longitude information. According to the user's location information, the sentiment value is located to count the average value of the sentiment of each province, and the spatial visualization is performed, as shown in Figure 4. During Typhoon Haiyan, the overall public sentiment in the Philippines was low in the south, high in the north, low in the east, and high in the west. Typhoon Haiyan made landfall on Leyte, a coastal island in the central part of the Philippines. The maximum wind force was 75 m/s when it landed, and it then moved northwest while the wind gradually weakened. The sentiment was low near the typhoon landing point Leyte, which is 0.102. Similarly, the public sentiment in areas close to the typhoon path was relatively low.

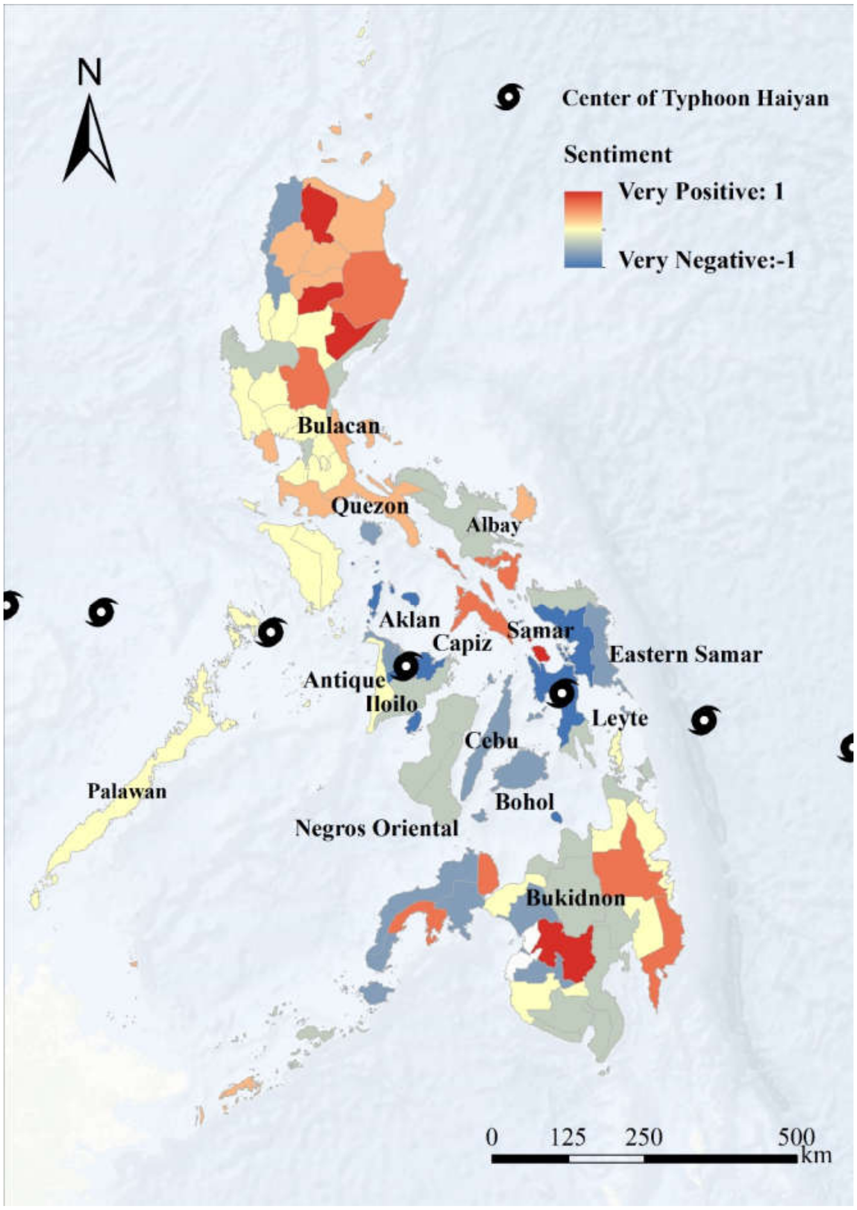

Figure 4. The spatial distribution of public sentiment during Typhoon Haiyan.

4.2. Topic Analysis of Public Sentiment

4.2.1. The Evolution of Public Topics

Through BTM, we obtained 12 topics and the top 20 words with the highest probability of occurrence in each topic. These words can describe the general content of this topic. Since some themes describe similar content, we manually merge these themes and finally obtain the five categories of disaster situation, reconstruction, praying, demand and other, as shown in Table A2.

By counting the number of tweets on different topics in the units of days, the daily changes in public topics from November 5 to November 15 are obtained, as shown in Figure 5. According to Figure 5, the impact of Typhoon Haiyan can be roughly divided into four stages. November 5 to November 7 is the "early warning stage", where typhoon warning signals were issued in various regions, and information about the disaster was disseminated. At the same time, people began to pray for safety and prepare corresponding disaster response measures. In Figure 3, public sentiment gradually rose at this stage.

The typhoon landed on November 8, and the number of tweets reached its peak. Afterwards, the typhoon began to affect the Philippines, which represents the "impact stage." At this stage, the number of tweets for "Disaster situation" and "Praying" declined but still accounted for the largest proportion, and information about "Rescue" gradually increased. Similarly, at this stage, people were affected by the typhoon, and public sentiment dropped significantly. November 11 to November 13 is the "emergency stage", where disaster relief was in full swing, and the "Rescue" tweets rose rapidly and remained at a high level. Public sentiment also began to increase. After November 13, that is, approximately a week after the disaster, the number of tweets related to "Reconstruction" remained at a certain number, the "disaster recovery stage" started, tweets on other topics gradually declined, and attention decreased.

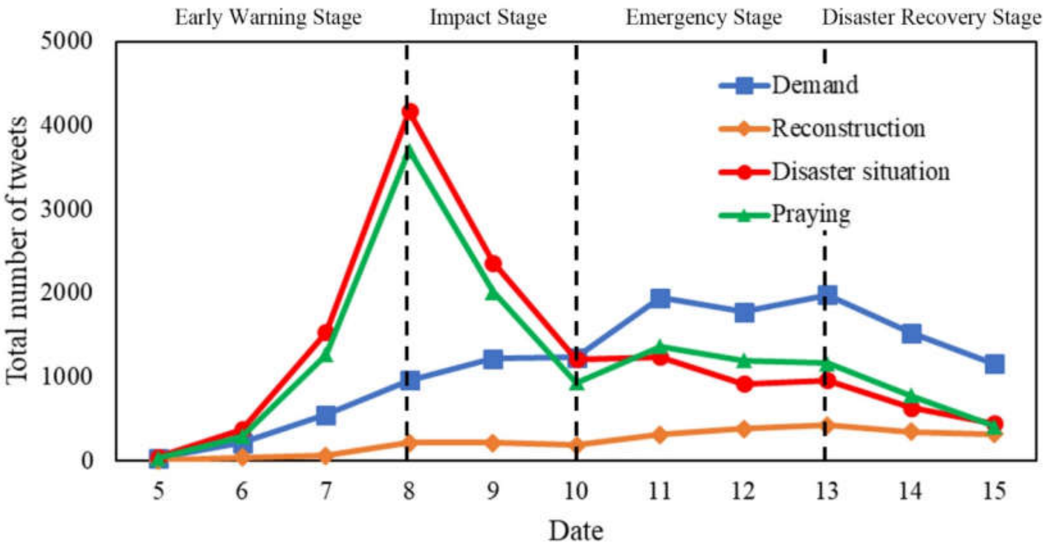

Figure 5. Daily changes in the topic of public discussion from November 5, 2013 to November 15, 2013.

4.2.2. Public Topics under Different Sentiments

With sentiment value = 0 as the boundary, tweets are divided into two categories: positive and negative. We separately classify positive tweets and negative tweets to observe the differences in the public discussion content under different sentiments.

Use BTM to classify positive sentiment tweets and negative sentiment tweets separately. The topics of positive tweets are divided into 5 categories, specifically, "Information Interaction", "Rescue", "Supplies Preparation", "Disaster Situation" and "Praying". Negative tweets are also classified into five categories, namely, "Disaster Situation", "Request Rescue", "Outside Rescue", "Traffic Impact" and "Praying".

As shown in Figure 6, in the positive tweets, people's attention to topics is ranked as follows: Disaster Situation (30%)> Supplies Preparation (28%)> Rescue (19%)> Praying (17%)> Information Interaction (6%). In the negative tweets, people's attention to topics is ranked as follows: Disaster Situation (30%)> Praying (28%)> Request Rescue (27%)> Outside Rescue (10%)> Traffic Impact (5%).

Among the positive tweets, the sentiment value of Supplies Preparation is the highest (0.42), which indicates that people were actively responding to disasters and preparing materials; the second highest sentiment value was Praying (0.38), where people prayed for the safety of themselves or others. The sentiment value of Information Interaction is 0.35. Before and during Typhoon Haiyan, the government, institutions, and the public used Twitter to exchange information and release news such as rescue hotlines,

fundraising websites, and rescue addresses, which were very active. Among the negative tweets, the lowest sentiment value is Praying (–0.37), followed by Disaster Situation (–0.24), which shows that people were very worried about the damage caused by Typhoon Haiyan.

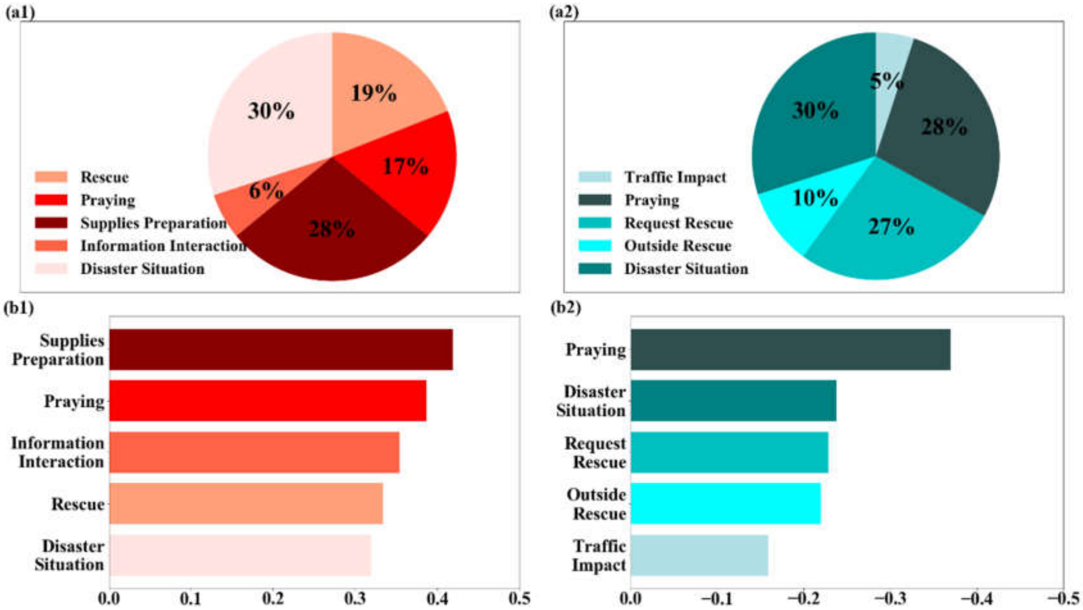

Figure 6. The proportion of topics and sentiment ranking in positive and negative tweets.(a1) Topics of positive tweets(a2) Topics of negative tweets(b1) The sentiment ranking of positive tweet topics (b2) The sentiment ranking of negative tweet topics.

4.3. The influencing Factors of Sentiment Value

During a disaster, there are various factors that affect public emotions. We discuss the influencing factors from the four aspects of disaster, economy, society and culture. As shown in Table 3, we selected seven specific indicators to represent these four aspects. The spatial pattern of these indicators is shown in Figure 7.

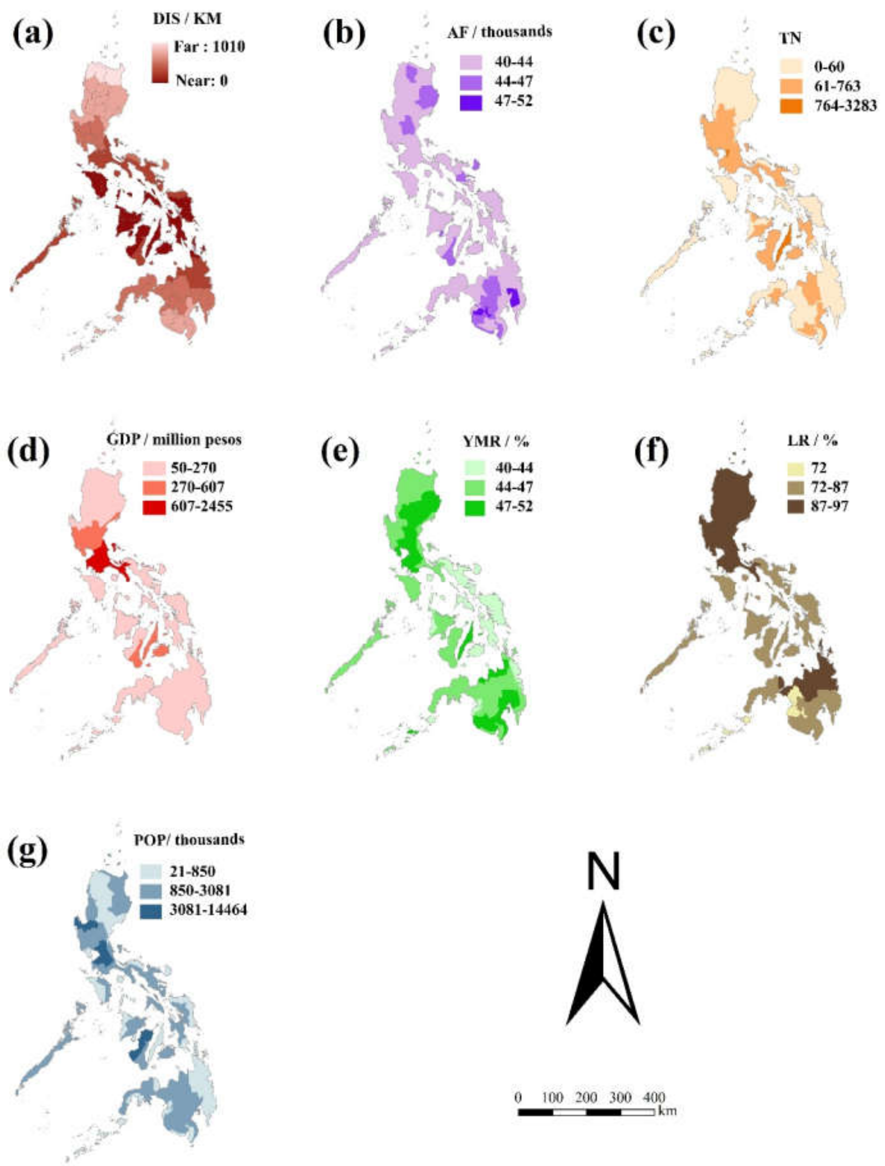

Figure 7. The spatial pattern of 7 specific indicators. (**a**)DIS (**b**) AF (**c**) TN (**d**) GDP (**e**) YMR (**f**) LR (**g**)POP

4.3.1. Results of Factor Detection

The factor detection results show the interpretive ability of the candidate factors for the overall sentiment value. As show in Figure 8, TN (Number of tweets, q=0.32) has the highest q value and the strongest determinant power, which indicates that TN is the dominant factor. The number of tweets represents people's desire to express. When there are more tweets, the desire to express is stronger, and the emotions are richer. POP (Population, q=0.18) and YMR (Young and middle-aged population ratio, q=0.17) follow TN. The demographic structure of society has two influences on sentiment. First, the young and middle-aged population is the main user of social media, and most of the tweets on Twitter are posted by them. Second, the young and middle-aged population are the backbone of disaster emergency management, and they are the most active in responding to government calls and responding to disasters. AF (Number of people affected, q=0.16) reflects

the impact of disasters. The disaster situation of the population affects peoples emotions. In addition, GDP (Gross domestic product, q=0.08), DIS (Distance to typhoon center, q=0.07), and LR (Literacy rate, q=0.07) also affect public sentiment, but the impact is relatively small.

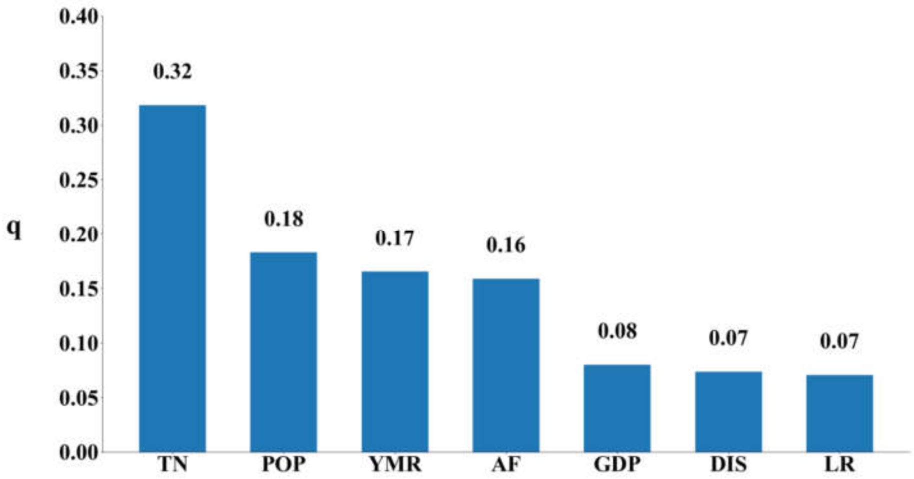

Figure 8. Results of factor detection.

4.3.2. Results of Interactive Detection

Interaction detection evaluates whether the combined effect of two factors affects the interpretation of overall sentiment. Figure 9 shows the results of the interaction detection. The results show that, except for the interactions among DIS-LR, GDP-YMR, GDP-AF, YMR-AF, and LR-AF, which are independent, the interactions among the other factors increase the determinant power on sentiment. The interaction of TN-POP has the strongest ability to explain sentiment, followed by TN-YMR, which are 0.51 and 0.47, respectively. Under the joint action of disaster factors and social factors, the spatial difference in sentiment will be more obvious. TN-AF, TN-GDP, TN-LR, AF-POP, and DIS-TN also have relatively high interpretive capabilities for sentiment. This shows that disaster factors significantly enhance their interpretive capabilities when interacting with other factors, while the interaction among society, economy, and culture is not as obvious.

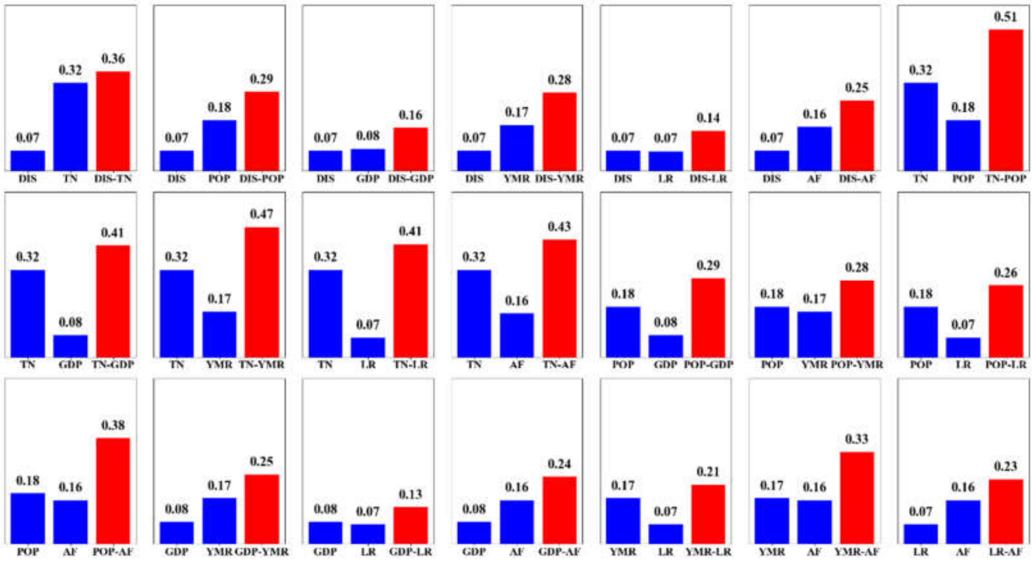

Figure 9. Results of interactive detection. Blue represents the q value of a single factor, and red represents the q value of the interaction of two factors.

5. Discussion

In this study, we analyzed the temporal and spatial patterns of public sentiment during Typhoon Haiyan and explored the differences in content under different sentiments and sentiment driving factors.

As stated by Anwar Hridoy et al. [46], social media data are one of the most effective and accurate indicators for studying public sentiment. After the disaster, Twitter was full of people's opinions and comments, and we analyzed public sentiment based on these data.

TextBlob is used to calculate specific sentiment values, and the quantified data can better reflect the trend of sentiment changes. Through topic classification and drawing the daily variation curve, we found that the topics discussed by the public after the disaster are mainly divided into the four categories of "demand", "disaster situation", "praying", and "reconstruction". The changing characteristics of these topics reflect the different stages of the disaster, which is basically in line with the five main stages of the disaster cycle as defined by Debarati and Michel [47], namely the early warning stage, impact stage, emergency stage, recovery stage, and reconstruction stage. The evolution of public topics corresponds to changes in sentiment and can be explained by one another.

Similarly, we classified the positive and negative tweets separately to explore the differences in the content that people pay attention to in different sentiment states. Among the positive and negative emotions, people are most concerned about the "disaster situation". After a disaster occurs, people have a wide range of discussions about the disaster situation. These discussions can be used for disaster monitoring and disaster assessment [19,39].

Many scholars use Twitter to study Typhoon Haiyan. Takahashi et al. [48] obtained Twitter information through three hashtags, analyzed the topics of tweets before, during and after Typhoon Haiyan, and found that reporting second-hand information, commemorating and coordinating disaster relief work were the main topics. David et al. [49] revealed the time evolution of the discussion about Typhoon Haiyan on Twitter, and found that tweets about typhoon and disaster relief were dominant. However, these two studies use manual coding to identify the subject of tweets, which is not efficient. Our research uses BTM and TextBlob to mine topics and sentiment. After manual inspection (Table A3,Table A4), it proves that BTM can effectively identify the topic of tweets, and Textblob can quickly identify the sentiment of tweets.

Unexpectedly, under positive and negative sentiments, some topics of the tweets are similar, including "disaster situation", "praying" and "rescue". Table 3 lists the positive and negative tweets of these three types of topics.

(1) Disaster situation: In negative tweets, people describe the information and impact of Typhoon Haiyan, usually with negative emotions. In positive tweets, people usually use non-emotional sentences to state the disaster situation and describe their current situation as optimistic, for example, "As of 8 am, rainfall moderate but the winds are pretty fierce We still have electricity Hope every 1 Else is safe". This type of tweet usually has a positive sentiment.

(2) Praying: In positive tweets, people mostly express blessings directly, for example, "Hope we'll be all safe" and "I believe that our house is strong". In negative tweets, people first describe the disaster as bad and terrible and then express blessings, such as "Here comes the devastating Typhoon Pray for the Visayas", "The things that are happening in the Philippines is just so devastating Hold on & let's keep on praying". This type of tweet not only is related to praying but also contains disaster information, which leads to negative emotions in the tweet.

(3) Rescue: In positive tweets, most of the rescue is the distribution of relief supplies, evacuation, etc., while in negative tweets, people describe the disaster and the current situation and then request rescue, such as "Alert MACUPA LEYTE, is in URGENT NEED of food and water No rescue & people are getting sick Need response ASAP".

In disaster emergency management, understanding the topics that people are concerned about, guiding negative topics, solving related problems, and understanding peoples sentiment states and trends are very important for maintaining social order in disasters.

Table 3. Examples of tweets with similar topics in positive and negative tweets.

Topic	Positive	Negative
**Disaster situation**	As of 8 am, rainfall moderate but the winds are pretty fierce We still have electricity Hope every1 else is safe	PAGASA said Metro Manila will experience the worst of by 5 p m or 6 p m tonight when typhoon reaches Mindoro via PIA-NCR
**Rescue**	About 349 residents, mostly members of the Ati tribe, have been evacuated to safer ground in City of Naga, via	Devastating To all travelers who have the Philippines, pls help spread word We need help
	DSWD Central Visayas in Cebu City monitoring DSWD has 7000 relief packs ready for dispatch	Alert MACUPA LEYTE, is in URGENT NEED of food and water No rescue & people are getting sick Need response ASAP
	One bright spot this stormy night is Dinagat's PDRRMC It is well coordinated and quick to respond to requests for assistance	Brgy Hipona,Pontevedra, Capiz badly need help No electricity Flood and bad communication
**Praying**	Hope we'll be all safe 'til the typhoon passed the Philippine area	Here comes the devastating Typhoon Pray for the Visayas
	I believe that our house is strong and that it can withstand him at any pressure of Have faith	How insane is it to see all these photos of destruction and look out my window to see sunlight I pray for everyone affected
	Prayer is the best weapon	The things that are happening in the philippines is just so devastating Hold on & let's keep on praying

After further analysis of the content of the tweets, we found that compared to positive tweets, tweets with negative emotions can better reflect people's situation and should be given more attention in disaster emergency management. For example, in negative tweets, the demand for relief supplies mentioned by people is usually unresolved and urgent, and the disaster impacts described by people are often more severe than those in positive tweets. Therefore, in disaster rescue, relief supplies distribution, and post-disaster recovery, it is necessary to pay more attention to negative tweets, to dig out the relevant information in the tweets, and to pay more attention to the areas where negative tweets are concentrated. Bai and Yu [3] developed a framework based on crowd negative emotions prediction, which can discover incident events in a post-disaster situation.

Previous studies have shown that the spatial characteristics of public emotions are correlated with factors such as geographic location and disaster intensity, and there is an aggregation pattern of positive and negative emotions [27,50,51]. Wang and Taylor [52] conducted a linear regression analysis on sentiment and earthquake intensity and revealed a negative correlation between the collective emotional level and earthquake intensity, but they did not explore the relationship between the sentiment level and other factors. Lin [53] identified communities with relatively stable sentiment characteristics and analyzed the correlation between their sentiment and community demographic characteristics. Different from previous studies, the use of Geodetecor to analyse the sentiment has three advantages: (1) Our research uses the four aspects of society, economy, culture, and disasters and seven specific indicators to explore the influencing factors of sentiment. (2) Geodetecor can detect both numerical data and qualitative data, which helps us take social data into account. (3) The public's sentiment is usually the result of a variety of factors. Therefore, using Geodetector can help us analyze the interaction between two factors.

The results of the sentiment analysis were related to the Twitter data. Although the Twitter API does not obtain all the Twitter data, the Twitter API is indeed suitable for research [54]. Twitter allows users to quickly share information and interact with other users using specific hashtags. These hashtags can be used to search for specific topics. Keyword-based acquisition of data is not entirely appropriate because it does not assess the relevance of the content of the tweet to a specific situation [55]. Whether keywords can represent events, whether they are specific, and comprehensive will directly affect the effectiveness of data acquisition. Generally, researchers choose keywords based on experience or expert opinions, and manual keyword selection is the main cause of content bias [56]. In this study, hashtags related to Typhoon Haiyan were selected based on the hashtag screening method. On the one hand, tweets can be obtained as comprehensively as possible, reducing content deviation caused by manual selection. On the other hand, during disasters, social media is not all about disasters. Through hashtag screening, only data related to target cases can be obtained. The analysis of such tweets can more truly reflect people's sentiment about disasters.

This study has certain limitations in sentiment analysis and influencing factor detection. At present, we only divide the polarity of tweets, without a fine-grained division of sentiments, which may result in insufficient sentiment description. In the selection of influencing factors, seven specific factors were selected to represent the four aspects of society, economy, culture, and disasters. In future research, more factors can be considered. In addition, since the official languages of the Philippines are Filipino and English, there are multiple languages in tweets. To facilitate processing and understanding, Filipino tweets are first translated into English during data preprocessing. However, perhaps due to writing errors or some unique usages, the meaning of some tweets (1.4% of the total tweets) cannot be accurately understood, so they have not been translated, which may affect the effects of sentiment analysis and topic classification. In future research, the analysis of different kinds of languages can be considered.

6. Conclusions

This study analyzes the sentiment state of the public during Typhoon Haiyan, explores the changing process of public discussion topics at different stages after the disaster, and analyzes the differences in people's discussion content under different sentiments. During Typhoon Haiyan, the public's overall sentiment was positive, and the evolution of sentiments and topics reflected the different stages of the disaster. Compared with positive tweets, negative tweets contain more serious disaster information and more urgent demand information and are more worthy of attention in disaster emergency management. In addition, we visualized public sentiment in space, analyzed its spatial pattern, explored the influencing factors of the spatial differences, and quantitatively described the explanatory ability of these factors. The results show that the number of tweets, population, and the proportion of the young and middle-aged population are the dominant factors in the sentiment spatial differences. At the same time, the interaction between disaster factors and society, economy, and culture is the most obvious, and the ability to explain sentiment during the interaction is significantly increased.

Our research provides quantifiable sentiment data that will help to better evaluate the public's sentiment state. The analysis of topics can quickly explain the public content and development status, which will help the government to effectively respond to public sentiment, guide topics, and control the spread of negative emotions. The analysis of influencing factors can be used to reveal the driving mechanism of sentiment differences. In the future, we hope to analyze more cases in disasters, public safety, and epidemics, and to consider more influencing factors to achieve more general conclusions.

Author Contributions: Conceptualization, Changxiu Cheng; Funding acquisition, Changxiu Cheng; Investigation, Ting Zhang; Methodology, Ting Zhang; Project administration, Changxiu Cheng; Validation, Ting Zhang; Writing – original draft, Ting Zhang; Writing – review & editing, Changxiu Cheng, Ting Zhang. All authors have read and agreed to the published version of the manuscript.

Funding: This research was funded by the National Key Research and Development Plan of China, Grant No.2017YFB0504102 and National Natural Science Foundation of China, Grant No. 41771537.

Data Availability Statement: The data presented in this study are openly available in [FigShare] at [10.6084/m9.figshare.13491087].

Acknowedgments: We are very grateful to the high-performance computing support from the Center for Geodata and Analysis, Faculty of Geographical Science, Beijing Normal University (https://gda.bnu.edu.cn/).

Conflicts of Interest: The authors declare no conflicts of interest

Appendix A

Table A1. Hashtags related to typhoon Haiyan.

Hashtags
#bangonvisayas #haiyan #ormoc #prayfortacloban #prayforphilippines #prayfortheph #prayforthephilippines #reliefph #rescuph #strongrph #supertyphoon #surigaodelnorte #tacloban #typhoonhaiyan #typhoonhaiyan #uge #yolanda #yolandaupdates #safenow #tracingph #yolandaph

Table A2. Results of the manual topic description and classification.

Topic ID	TOP20 words	Topic description	Category
1	Relief, aid, effort, affect, govern, aquino, disast, survivor, respons, recoveri,	Government disaster emergency response, disaster relief	Demand

	presid, rehab, govt, assist, gov, nation, fund, support, post, rehabilit		
2	Sa, ang, ng, po, lang, mga, pa, ni, ko, grabe, sana, yung, guy, tayo, hope, ka, naman, news, si, haha	Filipino text	Other
3	Survivor, victim, rais, benefit, support, fund, relief, proceed, photo, light, christma, sale, typhoon, concert, love, tonight, pm, parti, flag, join	Post-disaster fundraising, condolences, commemoration	Reconstruction
4	Samar, leyt, cebu, island, eastern, signal, northern, town, visaya, famili, iloilo, citi, updat, affect, damag, provinc, bantayan, power, hit, guiuan	Disaster area	Disaster situation
5	Water, relief, food, cebu, citi, team, survivor, suppli, medic, leyt, send, arriv, aid, oper, affect, power, airport, ship, emerg, hospit	Various relief supplies, medical assistance	Demand
6	Relief, volunt, pack, dswd, repack, effort, oper, drop, center, survivor, accept, call, op, villamor, contact, citi, cebu, check, pm	Rescue, package, distribute supplies	Demand
7	News, watch, report, heart, death, dead, heartbreak, devast, live, toll, break, cnn, leyt, video, aftermath, sad, cri, happen, updat, ndrrmc	News reports, disaster casualties, mass sentiment	Disaster situation
8	Red, text, cross, updat, affect, globe, free, smart, call, person, send, tweet, list, relief, amount, servic, info, hashtag, link	Fundraising, Red Cross, Rescue	Demand
9	Citi, leyt, famili, cebu, hous, san, tree, brgi, mayor, school, class, ormoc, evacu, photo, palo, roof, damag, resid, center, jose	Trees, bridges, roofs, etc. are damaged by typhoons, school holidays	Disaster situation
10	God, filipino, hope, prayer, bless, lord, affect, countri, stay, storm, strong, love, super, faith, spirit, visaya, heart, famili, stronger, guy	Bless, pray	Praying
11	Wind, pm, rain, manila, strong, pagasa, metro, kph,	Describe the hazard factors such as wind, rain, etc.	Disaster situation

	heavi, expect, weather, updat, eye, signal, stay, citi, novemb, warn, km		
12	Strongest, storm, super, hit, filipino, haiyan, countri, surg, histori, nation, record, cnn, landfal, philippin, god, stronger, stay, cyclon, planet	Describe hazards such as storms, waves, mudslides, etc.	Disaster situation

Table A3. Results of the manual test of the BTM.

Actual category / Forecasted category	Demand	Praying	Reconstruction	Disaster situation	Others	Number of samples	Precision
Demand	64	2	8	8	23	100	0.64
Praying	6	72	5	2	15	100	0.72
Reconstruction	1	4	64	3	28	100	0.64
Disaster situation	10	13	4	59	14	100	0.59
Others	3	13	0	2	82	100	0.82
Recall	0.76	0.69	0.79	0.86	0.51		
Total Accuracy							0.68

Table A4. Results of the manual test of the TextBlob

Actual category / Forecasted category	Positive	Negative	Number of samples	Precision
Positive	67	33	100	0.67
Negative	38	62	100	0.72
Disaster situation	10	13	100	0.59
Others	3	13	100	0.82
Recall	0.76	0.69		
Total Accuracy				0.68

Reference

1. Cao, X.; Zhang, X.; Liu, L.; Fang, K.; Duan, F.; Li, S. Analysis of public opinion heats of emergencies based on response level. *China Manag. Sci.* **2014**, *22*, 82–89.
2. Wang, R.; ZHang, E.; Li, T. Research on public opinion countermeasures of incidents based on Weibo. *Intell. Sci.* **2016**, *34*, 94–98.
3. Bai, H.; Yu, G. A Weibo-based approach to disaster informatics: Incidents monitor in post-disaster situation via Weibo text negative sentiment analysis. *Nat. Hazards* **2016**, *83*, 1177–1196, doi:10.1007/s11069-016-2370-5.
4. Abel, F.; Hauff, C.; Houben, G.-J.; Stronkman, R.; Tao, K. Twitcident: Fighting fire with information from Social Web streams. In Proceedings of the WWW'12-Proceedings of the 21st Annual Conference on World Wide Web Companion, Lyon, France, 16-20 April 2012; pp. 305–308.
5. Alexander, D.E. Social Media in Disaster Risk Reduction and Crisis Management. *Sci. Eng. Ethics* **2014**, *20*, 717–733, doi:10.1007/s11948-013-9502-z.
6. Cameron, M.; Power, R.; Robinson, B.; Yin, J. Emergency situation awareness from twitter for crisis management. In Proceedings of the 21st World Wide Web Conference 2012, Lyon, France, 16–20 April 2012.

7. Chowdhury, S.; Imran, M.; Asghar, M.R.; Amer-Yahia, S.; Castillo, C. Tweet4act: Using Incident-Specific Profiles for Classifying Crisis-Related Messages. In Proceedings of the 10th international conference on information systems for crisis response and management, Baden-Baden, Germany, 19–22 May 2013
8. Lachlan, K.A.; Spence, P.R.; Lin, X. Expressions of risk awareness and concern through Twitter: On the utility of using the medium as an indication of audience needs. *Comput. Hum. Behav.* **2014**, *35*, 554–559, doi:10.1016/j.chb.2014.02.029.
9. Liu, B.F.; Fraustino, J.D.; Jin, Y. Social Media Use During Disasters: How Information Form and Source Influence Intended Behavioral Responses. *Commun. Res.* **2015**, *43*, 626–646, doi:10.1177/0093650214565917.
10. Martínez-Rojas, M.; Maria, P.F.; Rubio-Romero, J.C. Twitter as a tool for the management and analysis of emergency situations: A systematic literature review. *Int. J. Inf. Manag.* **2018**, *43*, 196–208, doi:10.1016/j.ijinfomgt.2018.07.008.
11. Kent, J.D.; Capello, H.T. Spatial patterns and demographic indicators of effective social media content during the Horsethief Canyon fire of 2012. *Cartogr. Geogr. Inf. Sci.* **2013**, *40*, 78–89, doi:10.1080/15230406.2013.776727.
12. Robinson, B.; Power, R.; Cameron, M. A sensitive Twitter earthquake detector. In Proceedings of the 22nd International Conference on World Wide Web, Rio de Janeiro, Brazil, 13–17 May 2013; pp. 999–1002.
13. Wang, Z.; Ye, X. Social media analytics for natural disaster management. *Int. J. Geogr. Inf. Sci.* **2018**, *32*, 49–72, doi:10.1080/13658816.2017.1367003.
14. Cervone, G.; Sava, E.; Huang, Q.; Schnebele, E.; Harrison, J.; Waters, N. Using Twitter for tasking remote-sensing data collection and damage assessment: 2013 Boulder flood case study. *Int. J. Remote Sens.* **2016**, *37*, 100–124, doi:10.1080/01431161.2015.1117684.
15. Kryvasheyeu, Y.; Chen, H.; Obradovich, N.; Moro, E.; Van Hentenryck, P.; Fowler, J.; Cebrian, M. Rapid assessment of disaster damage using social media activity. *Sci. Adv.* **2016**, *2*, e1500779, doi:10.1126/sciadv.1500779.
16. Yates, D.; Paquette, S. Emergency knowledge management and social media technologies: A case study of the 2010 Haitian earthquake. *Int. J. Inf. Manag.* **2011**, *31*, 6–13, doi:10.1016/j.ijinfomgt.2010.10.001.
17. Olteanu, A.; Castillo, C.; Diaz, F.; Vieweg, S. CrisisLex: A lexicon for collecting and filtering Microblogged communications in crises. In Proceedings of the International AAAI Conference on Web and Social Media, Ann Arbor, MI, USA, **2014**; pp. 376–385.
18. Nazer, T.H.; Xue, G.; Ji, Y.; Liu, H. Intelligent Disaster Response via Social Media Analysis A Survey. *ACM SIGKDD Explor. Newsl.* **2017**, *19*, 46–59, doi:10.1145/3137597.3137602.
19. Fohringer, J.; Dransch, D.; Kreibich, H.; Schröter, K. Social media as an information source for rapid flood inundation mapping. *Nat. Hazards Earth Syst. Sci.* **2015**, *15*, 2725–2738, doi:10.5194/nhess-15-2725-2015.
20. Imran, M.; Castillo, C.; Lucas, J.; Meier, P.; Rogstadius, J. Coordinating Human and Machine Intelligence to Classify Microblog Communications in Crises. In Proceedings of the 11th International ISCRAM Conference, University Park, PA, USA, 2014.
21. Purohit, H.; Castillo, C.; Diaz, F.; Sheth, A.; Meier, P. Emergency-Relief Coordination on Social Media: Automatically Matching Resource Requests and Offers. *First Monday* **2014**, *19,* 1–6, doi:10.5210/fm.v19i1.4848.
22. Wang, Y.; Li, H.; Wang, T.; Zhu, J. The Mining and Analysis of Emergency Information in Sudden Events Based on Social Media. *Geomat. Inf. Sci. Wuhan Univ.* **2016**, *41*, 290–297, doi:10.13203/j.whugis20140804.
23. Zhang, T.; Shen, S.; Cheng, C.; Su, K.; Zhang, X. A topic model based framework for identifying the distribution of demand for relief supplies using social media data. *Int. J. Geogr. Inf. Sci.* **2021**, *10*, 1–22, doi:10.1080/13658816.2020.1869746.
24. Ligutom, C.; Orio, J.V.; Ramacho, D.A.M.; Montenegro, C.; Oco, N. Using Topic Modelling to make sense of typhoon-related tweets. In Proceedings of the 2016 International Conference on Asian Language Processing (IALP), Tainan, Taiwan, 23–26 November 2016; pp. 362-365.
25. Wilson, T.; Wiebe, J.; Hoffmann, P. Recognizing contextual polarity: An exploration of features for phrase-level sentiment analysis. *Comput. Linguistics* **2009**, *35*, 399–433, doi:10.1162/coli.08-012-R1-06-90.
26. Thelwall, M.; Buckley, K.; Paltoglou, G.; Cai, D.; Kappas, A. Sentiment strength detection in short informal text. *J. Am. Soc. Inf. Sci. Technol.* **2010**, *61*, 2544–2558, doi:10.1002/asi.21416.
27. Neppalli, V.; Caragea, C.; Squicciarini, A.; Tapia, A.; Stehle, S. Sentiment analysis during Hurricane Sandy in emergency response. *Int. J. Disaster Risk Reduct.* **2017**, *21*, 213–222, doi:10.1016/j.ijdrr.2016.12.011.
28. Nagy, A.; Stamberger, J. Crowd Sentiment Detection during Disasters and Crises. In Proceedings of the 9th International ISCRAM Conference, Vancouver, BC, Canada, 2012.
29. Schulz, A.; Paulheim, H.; Schweizer, I.J.K. A Fine-Grained Sentiment Analysis Approach for Detecting Crisis Related Microposts. In Proceedings of the 10th International ISCRAM Conference, Baden-Baden, Germany,19–22 May 2013.
30. NDRRMC. *Final Report Effects of Typhoon Yolanda (Haiyan)*; NDRRMC, Ed.; NDRRMC: Quezon City, Philippines, 2014.
31. Nikita, M.; Cheng, C. Disaster Hashtags in Social Media. *ISPRS Int. J. Geo-Inf.* **2017**, *6*, 204.
32. Zhang, J.; Ye, Q.; Law, R.; Li, Y. The impact of e-word-of-mouth on the online popularity of restaurants: A comparison of consumer reviews and editor reviews. *Int. J. Hosp. Manag.* **2010**, *29*, 694–700, doi:10.1016/j.ijhm.2010.02.002.
33. Bo, P. Opinion Mining And Sentiment Analysis (Foundations And Trends(R) In Information Retrieval). *Found. Trends Inf. Retr.* **2008**, *2*, 1–135.
34. Feldman, R. Techniques and Applications for Sentiment Analysis. *Commun. ACM* **2013**, *56*, 82–89, doi:10.1145/2436256.2436274.
35. Neviarouskaya, A.; Prendinger, H.; Ishizuka, M. Affect Analysis Model: Novel rule-based approach to affect sensing from text. *Int. J. Nat. Lang. Eng.* **2011**, *17*, 95–135.
36. Volkova, S.; Wilson, T.; Yarowsky, D. Exploring Sentiment in Social Media: Bootstrapping Subjectivity Clues from Multilingual Twitter Streams. In Proceedings of the 51st Annual Meeting of the Association for Computational Linguistics, Sofia, Bulgaria, 4–9 August 2013.

37. Xu, L. Constructing the Affective Lexicon Ontology. *J. China Soc. Sci. Tech. Inf.* **2008**, *27*, 180–185.
38. Steven, L. TextBlob: Simplified Text Processing. Available online: http://textblob.readthedocs.org/en/dev(accessed at 12 March 2020).
39. Khan, R.; Urolagin, S. Airline Sentiment Visualization, Consumer Loyalty Measurement and Prediction using Twitter Data. *Int. J. Adv. Comput. Sci. Appl.* **2018**, *9*, 9, doi:10.14569/IJACSA.2018.090652.
40. Micu, A.; Micu, A.E.; Geru, M.; Lixandroiu, R.C. Analyzing user sentiment in social media: Implications for online marketing strategy. *Psychol. Mark.* **2017**, *34*, 1094–1100, doi:10.1002/mar.21049.
41. Rezgui, A.; Fahey, D.; Smith, I. *AffinityFinder: A System for Deriving Hidden Affinity Relationships on Twitter Utilizing Sentiment Analysis*; IEEE: Piscataway Township, NJ, USA, 2016; pp. 212–215.
42. Wiebe, J.M. Tracking point of view in narrative. *Comput. Linguist.* **1994**, *20*, 233–287.
43. Yan, X.; Guo, J.; Lan, Y. A biterm topic model for short texts. In Proceedings of the 22nd international conference on World Wide Web-WWW, Rio de Janeiro, Brazil, 13 May 2013; pp. 45–56.
44. Su, k.; Cheng, C.; Nikita, M.; Zhang, T. Application and Comparison of Topic Model in Identifying Latent Topics from Disaster-Related Tweets. *J. Geo-Inf. Sci.* **2019**, *21*, 1152–1160, doi:10.12082/dqxxkx.2019.190046.
45. Wang, J.; Xu, C. Geodetector: Principle and prospective. *Acta Geogr. Sin.* **2017**, *72*, 116–134.
46. Hridoy, S.A.A.; Ekram, M.T.; Islam, M.S.; Ahmed, F.; Rahman, R.M. Localized twitter opinion mining using sentiment analysis. *Decis. Anal.* **2015**, *2*, 8, doi:10.1186/s40165-015-0016-4.
47. Debarati, G.-S.; Michel, F.L. Information systems and needs assessment in natural disasters: An approach for better disaster relief management. *Disasters* **1986**, *10*, 232–237.
48. Takahashi, B.; Tandoc, E.C.; Carmichael, C. Communicating on Twitter during a disaster: An analysis of tweets during Typhoon Haiyan in the Philippines. *Comput. Hum. Behav.* **2015**, *50*, 392–398, doi:10.1016/j.chb.2015.04.020.
49. David, C.C.; Ong, J.C.; Legara, E.F.T. Tweeting Supertyphoon Haiyan: Evolving Functions of Twitter during and after a Disaster Event. *PLoS ONE* **2016**, *11*, e0150190, doi:10.1371/journal.pone.0150190.
50. Bertrand, K.; Bialik, M.; Virdee, K.; Gros, A.; Bar-Yam, Y. Sentiment in New York City: A High Resolution Spatial and Temporal View. *arXiv* 20 August **2013,** arXiv: 1308.5010.
51. Chen, S.; Mao, J.; Li, G.; Ma, C.; Cao, Y. Uncovering sentiment and retweet patterns of disaster-related tweets from a spatiotemporal perspective–A case study of Hurricane Harvey. *Telemat. Inform.* **2020**, *47*, 101326, doi:10.1016/j.tele.2019.101326.
52. Wang, Y.; Taylor, J. Coupling sentiment and human mobility in natural disasters: A Twitter-based study of the 2014 South Napa Earthquake. *Nat. Hazards* **2018**, *92*, 907–925, doi:10.1007/s11069-018-3231-1.
53. Lin, Y.-R. Assessing Sentiment Segregation in Urban Communities. *Acm Int. Conf. Proceeding Ser.* **2014**, *2014*, 1–8, doi:10.1145/2639968.2640084.
54. Laylavi, F.; Rajabifard, A.; Kalantari, M. A Multi-Element Approach to Location Inference of Twitter: A Case for Emergency Response. *Isprs Int. J. Geo-Inf.* **2016**, *5*, 56, doi:10.3390/ijgi5050056.
55. Laylavi, F.; Rajabifard, A.; Kalantari, M. Event relatedness assessment of Twitter messages for emergency response. *Inf. Process. Manag.* **2017**, *53*, 266–280, doi:10.1016/j.ipm.2016.09.002.
56. Olteanu, A.; Vieweg, S.; Castillo, C. What to Expect When the Unexpected Happens: Social Media Communications Across Crises. In Proceedings of the 18th ACM Conference on Computer Supported Cooperative Work & Social Computing, Vancouver, BC, Canada, 14–18 March 2015; pp. 994–1009.
