# Supplementary material for: Estimation of Undetected Asymptomatic COVID-19 Cases in South Korea Using a Probabilistic Model
Source: Int J Environ Res Public Health. 2021 May 6;18(9):4946. doi: 10.3390/ijerph18094946 (PMC8124955; doi:10.3390/ijerph18094946)
Supplement: Supplementary file 1 [file ijerph-18-04946-s001.zip › 6/ijerph-1193106-fc done.pdf]

International Journal of
Environmental Research and Public Health

Article

Body Mapping in a Drug and Alcohol Treatment Program: Eliciting New Identity and Experience

Sophia Macken [1], Sally Nathan [1,*], Michelle Jersky [2], Katherine M. Boydell [3] and Alexandra Gibson [4]

[1] School of Population Health, UNSW Sydney, Sydney 2052, Australia; fe.macken@gmail.com
[2] Sydney Children's Hospital Network, Sydney 2031, Australia; michelle.jersky@health.nsw.gov.au
[3] Black Dog Institute, Sydney 2031, Australia; k.boydell@blackdog.org.au
[4] School of Health, Victoria University of Wellington-Te Herenga Waka, Wellington 6012, New Zealand; ally.gibson@vuw.ac.nz
* Correspondence: s.nathan@unsw.edu.au

Abstract: Arts-based approaches have not been widely used in the drug and alcohol treatment sector. In this study, we examined the utility of the arts-based method of Body Mapping in an Australian residential treatment centre. Two workshops were held to explore young people's strengths and support networks in order to improve understandings of young people's lives, identities and experiences of treatment. Semi-structured follow-up interviews were conducted and triangulated with observational notes and staff interviews. We identified four major themes: engaging through art; removing the mask; revealing strengths; and a sense of achievement. Overall, this study highlighted the value of Body Mapping as an approach to engage with young people, providing rich in-depth data about their lived experiences, including in the alcohol and other drug (AOD) treatment context.

Keywords: Body Mapping; drug and alcohol residential treatment; arts-based methods

Citation: Macken, S.; Nathan, S.; Jersky, M.; Boydell, K.M.; Gibson, A. Body Mapping in a Drug and Alcohol Treatment Program: Eliciting New Identity and Experience. *Int. J. Environ. Res. Public Health* **2021**, *18*, 4942. https://doi.org/10.3390/ijerph18094942

Academic Editor: Paul B. Tchounwou

Received: 6 April 2021
Accepted: 30 April 2021
Published: 6 May 2021

Publisher's Note: MDPI stays neutral with regard to jurisdictional claims in published maps and institutional affiliations.

Copyright: © 2021 by the authors. Licensee MDPI, Basel, Switzerland. This article is an open access article distributed under the terms and conditions of the Creative Commons Attribution (CC BY) license (http://creativecommons.org/licenses/by/4.0/).

1. Introduction

Problematic alcohol and other drug (AOD) use is a significant public health issue, warranting attention in research, policy and practice [1,2]. Problematic AOD use is often determined by the diagnostic criteria for 'substance abuse or dependence' or 'substance use disorders' in the Diagnostic and Standard Manual of Mental Disorders fourth edition (DSM IV) [3] and DSM V [4], respectively. Adolescents, aged 13–18 years, are more likely to engage in risky health behaviours, including experimenting with drugs and alcohol, than any other age group [1,5]. Drug and alcohol use can also quickly escalate to problematic levels in this age group, which can have detrimental consequences on neurological development [6,7], as well as complex and wide-reaching emotional, educational and socio-familial implications [8,9].

Treatment options for adolescents with problematic AOD use can include residential treatment, such as a therapeutic community (TC) model, which aim to address the complex health and social support needs of these young people [10–12]. In this treatment field, research underscores the importance of treatment completion to improve long-term outcomes for these young people [13,14]. Benefits of treatment completion or longer stays are shown to include lower levels of drug use, fewer arrests, increased employment rates and improved social engagement and psychological well-being following treatment compared with those who left the program early [12,15–19]. 'Engagement' in programs, not just time in treatment, has also been identified as important in ensuring positive outcomes from residential programs [18,20–23]. Maximising engagement and preventing drop out are therefore key priorities for the residential AOD treatment sector.

TCs implement a wide array of treatment modalities and activities to support and engage participants in programs [11,21]. However, according to Foster and colleagues

(2010), adolescents in a TC in Sydney Australia wanted methods beyond the typical journaling and talk-based group sessions to reflect on their experiences, including their experiences in the program [24]. This finding, together with the increasing evidence of the need for 'engagement' to reduce treatment drop out and maximise positive outcomes from treatment [18,20–23], led the authors of this paper to explore the role of arts-based methods as a research tool in this setting, but also as a means of 'engagement' for clients in residential treatment programs.

Arts-based methods in research often involve the integration of artmaking in data collection alongside more traditional data collection methods such as interviews or focus groups [25–29]. Arts-based methods are understood as those in which participants engage in a creative practice of self-expression at any point in the research process. Practices of creative self-expression might include the use of art forms such as poetry, drawing, photographs, digital story telling or theatre for example [30,31]. Arts-based methods have been shown to both value and include participants' voices and give them greater agency in the research process including more control over the expression of their experiences and perspectives [32,33]. This has been particularly found for those who are often marginalised or silenced within society [26,27,34,35], as is commonly the case for young people accessing AOD services.

To date, there has been a lack of research examining methods to better engage and understand young people's experiences as they progress through AOD treatment. As such, this paper describes a study of the innovative visual method of Body Mapping, which is growing in its use in diverse contexts [36,37] to gain a deeper understanding of young people's identities, beliefs and experiences while in AOD treatment. Body Mapping involves creating life-sized artworks of the human body to visually depict an individual's perception of their body, identity and experiences [36,38]. Most commonly the method involves drawing an outline in pairs of a person's body in a position they wish to be represented on a large piece of drawing paper and then using a range of art materials to fill in the Body Map in response to questions posed in a workshop [36]. Body Mapping offers an alternate lens to capture, examine and better understand the complex layering of lived experiences, particularly in marginalised populations [36].

Body mapping originated in South Africa in 2002 as part of the Memory Box Project, a community outreach program, and was designed to facilitate the sharing of personal stories of women living with HIV and AIDS to raise community awareness and decrease the associated stigma [39]. Variations of this method have since been applied as a research tool and/or therapeutic intervention with vulnerable populations for a variety of health conditions [36], for example the experiences of young refugees [38], undocumented workers [37] and people with complex support needs [26]. However, Body Mapping has not been previously used to explore problematic AOD use nor experiences in treatment.

In this paper, we examine the utility of Body Mapping as a research method with young people in residential treatment for AOD issues and provide insight into how this approach can support young people to share their emotions, strengths and supports and elicit new narratives on identity and experience which may be useful to foster engagement and better support young people in treatment.

2. Methods

2.1. Setting

This study was undertaken at the Ted Noffs Foundation's Program for Adolescent Life Management (PALM), located in urban NSW, Australia. PALM is a modified TC for adolescents aged 13–18 years who are addressing complex issues related to their AOD use [24]. Adolescents are referred to PALM from the community and the juvenile justice system, with criterion for admission being a 'Pre-Treatment Assessment' and evidence of problematic AOD use as defined by the DSM-IV criteria. The DSM-IV criteria has routinely been used for PALM admissions for consistency and because DSM-IV facilitates the

distinction between "substance abuse" and "substance dependence," which helps stratify the severity of problematic AOD use and guide management decisions.

Broader issues owing to problematic AOD use, such as socio-familial or health implications, unstable living arrangements and impeded daily functioning are also considered. The program is abstinence based whilst in treatment, holistic and multi-disciplinary, providing up to three months of residential rehabilitation. The program incorporates life skills development, group work, family support, vocational education and individual counselling, designed to assist participants to effectively self-manage their own health and recovery [24]. The aim is for clients to remain in residence for the full duration of the program. However early discharges can occur as a result of self-discharge or disciplinary discharge for not complying with the rules of the program, as was the case for two participants involved in this study.

The space selected for the Body Mapping workshops was emptied of the usual chairs and tables which determine where people place themselves. In addition, art materials were purposefully placed to create a sense of wonder and curiosity for participants upon entering the workshop space. This helped to support the 'new' terms of engagement for participants. The creative 'warm up' exercises that participants were invited to engage in prior to commencing their body maps, were intended to build a sense of community and trust among participants and researchers. The fact that participants and researchers all engaged in these initial activities created a sense of being in this together. From the outset, participants and staff were able to further locate themselves in the space by choosing where in the room they would create their body map. Collaboration was facilitated by working in pairs to create the outline of their body map. The art materials chosen for the workshop were purposefully chosen to offer a wide range of choices for participants and included paint, collage and drawing materials. The wide range of art materials also allowed for the various levels of artistic skills amongst the group to be recognised and valued.

2.2. Study Objectives

1. To explore the use of Body Mapping as a research method with young people in residential treatment for AOD issues.
2. To examine how Body Mapping can engage young people in exploring their strengths and sources of support during treatment.

2.3. Study Design

Between August and September 2018, two separate Body Mapping workshops were piloted by the authorship team at PALM and follow-up interviews were held with participants within the following two-week period. The workshops lasted 2.5 h and focused on using an adaptation of the Body Mapping technique to explore the personal strengths and support systems that young people might and could draw on during residential treatment. The workshops were designed in collaboration with an artist (MJ) who also led the workshops. In discussions with staff, it was decided that one workshop would be solely with male-identifying residents and the second with female-identifying residents in order to ensure that participants felt as comfortable as possible when creating their body maps. Counselling staff members were present during each workshop to check in with young people and completed their own body map focused on the strengths that they draw on as staff members. The workshops included several activities to encourage relaxation and creativity before engaging in the Body Mapping process (see Appendix A Table A1).

2.4. Recruitment

Adolescents (aged 13–18 years) who were admitted to PALM during the study period were recruited. Sampling was purposeful, with staff determining which of the young people in PALM would be willing and able to take part in the workshops. Eight residents

took part in the Body Mapping workshops, comprising approximately half of the residents in PALM at the time. Five boys participated in the first workshop and three girls in the second workshop. Seven residents formally consented to the workshop, interview and publication of their body map. However, two male residents were discharged from the program owing to disciplinary behaviour prior to their scheduled interview date and one of those did not formally consent; thus, only six participants took part in the follow-up interviews. All residents who were interviewed were between the ages of 15 and 17 years and Table 1 is included to contextualize their stay in the program and demonstrate the diversity of the participants who took part in the workshop and interviews. Three Ted Noffs' counselling staff were also recruited to participate in the Body Mapping workshops and in follow-up interviews. The assistant-manager of the program chose which PALM counsellors would be involved, with two staff members taking part in the boys' workshop and one in the girls' workshop.

Table 1. Characteristics of Interviewed Participants.

Pseudonym.	Admission Type	Length of Stay at Time of Workshop (Days)	Identified as Aboriginal	Drug of Greatest Concern
Liam	Juvenile Justice	86	N	Tranquilizer
Connor	Case Worker	65	N	Amphetamine
Adrian	Family	49	N	Tranquilizer
Kaylee	Self	54	Y	Amphetamine
Talia	Juvenile Justice	6	N	Amphetamine
Emma	Self	13	Y	Amphetamine

2.5. Ethics

This study was approved on 27 January 2015 by the Aboriginal Health and Medical Research Council of NSW, Australia (1062/14), with the modification to include Body Mapping approved 12 March 2018. Informed written consent for participation in this study, audio recording of follow-up interviews and for a copy of completed Body Maps to be used in written publications was obtained from all participants who took part. Data in the form of de-identified transcripts and audio recordings are stored on a password protected server folder, only accessible to the research team. To illustrate key findings, participant and staff quotes are included as indented text with pseudonyms to protect their anonymity.

2.6. Data Collection

The main data collection processes for this study consisted of two Body Mapping workshops and semi-structured follow-up interviews, all of which were held at PALM. The Body Mapping workshops were centred on encouraging young people to visually depict their sources of strength and support when moving through treatment on a life-sized outline of their body on paper using a range of art materials supplied at the workshop. In addition to the artist who was leading the workshops (MJ), four members of the research team were involved in the first workshop as facilitators and three in the second. A recorded group discussion with all the participants was also conducted at the end of each workshop to gain young people's feedback on how they found the process of creating their body maps. Additionally, in-depth field notes were taken during the workshops by team members and a team debriefing with all the facilitators was also recorded at the completion of each workshop.

Semi-structured interviews were then conducted with each of the participants involved in the Body Mapping workshops. These interviews were scheduled with the help of Ted Noffs' staff in the week following the workshop. An interview guide was developed for participants and consisted of a series of open-ended questions designed to gain

an understanding of what the young people had depicted in their body maps and how they found the Body Mapping process (Figure 1). The interviews each lasted approximately 20–30 min. They were recorded and led by the same two field researchers (SM & AG). An A3-sized photo print out of the young person's body map was provided for the participant interviews as a visual aid when discussing the content of their body maps. This photo was given to the young people after the completion of the interview along with a $50 gift card to thank them for their time. Interviews were subsequently held with the three staff members who participated in the workshops. A separate interview guide was developed to focus on gaining the staffs' perspective on the workshops and how the young people responded to Body Mapping (Figure 2).

Body Mapping content/process:

- How did you find the day?
- How did you find choosing the objects? Do you remember which object you chose? Why do you think you chose this?
- How did you find doing the line drawing of your face?
- What about when you came to lying down on your body map? What were you thinking? How did you decide on your pose?
- Looking at your map now, what stands out for you? What do you notice about the colours, shapes, textures or materials you used and how you placed things on your map? What does this mean to you?
- How did you find the experience creating your own body map? What was going through your mind whilst you were doing that?
  - What did you like about doing it?
  - What did you find the most challenging?
  - If you could do it again, what things would you change (if anything)
- How did you feel that afternoon and on the weekend after doing that activity?
- How useful do you think doing an exercise like body mapping would be at PALM?

Sources of Support and Strengths:

- What does being strong mean to you in terms of getting through treatment?
- Who supports you? How do they show their support? What does having their support mean to you? How has this helped you through treatment?
- Is there anything about yourself that you think has helped you get through treatment so far? If so, what?
- How do you find talking about your strengths/supports?
- How do you feel you reflected your strengths/sources of support in your map? Looking back at these, how does it make you feel?
- If we had just asked about your strengths/support in an interview and not done the body maps, do you think this would have changed what you have just shared with us? In what way?

Figure 1. Interview Guide for Body Mapping Participants.

Body Mapping process:

From your perspective as a staff member at PALM, how did you think the workshop went?

How did you think the young people responded to doing an activity like body mapping?

Do you think being arts-based specifically, that worked for most people or would there be some barriers for some?

We are focusing on exploring the young people's strengths and sources of support? How do you think they responded to that?

How do you think this process helped them express their strengths and supports?

How did you find completing your own body map at the same time? What insights did the activity give you?

How do you think the young people felt afterwards? In the house/follow-up counselling

When do you think would be a good time to run body mapping for the young people in terms of their time in treatment – beginning, middle or end?

How feasible/useful do you think doing an exercise like body mapping would be at PALM? What aspects would need to change etc?

Figure 2. Interview Guide for PALM Staff.

As such, five sources of data were ultimately triangulated to examine the utility and effectiveness of Body Mapping as a research tool: (1) the visual data of the body maps, (2) in-depth interviews with participants, (3) staff interviews, (4) group discussion with participants following the workshop and 5) researcher field notes and team debriefs of the workshops.

2.7. Data Analysis

All interviews were recorded and transcribed verbatim by a professional transcriber. Transcripts were reviewed for accuracy by SM before being de-identified and assigning each of the participants and staff members a pseudonym. Analysis began one week after all interviews were completed and an inductive approach to thematic analysis was applied to generate initial codes (i.e., recurring concepts or meanings). Through repeated engagement with the transcripts, codes were expanded upon, refined and then grouped together to form broader themes [40]. NVivo qualitative data management software (version 12, QSR International Pty Ltd., Doncaster, Australia) was used to organise and manage the data. All data were coded by the same researcher (SM) and interpreted within a contextualist perspective to explore the young people's experiences, which are understood as existing within individualistic and broader societal contexts that give shape to these experiences. Regular meetings were held with the other research team members to discuss the interpretation of the data and development of codes to encourage reflexivity and ensure a more thorough and rigorous analytical approach.

3. Results

Body Mapping participants ($n = 6$) and staff ($n = 3$) were interviewed following the workshops, focusing on exploring how the young people found the Body Mapping process and how the method helped them to express their strengths and sources of support. Four recurrent themes were identified across the interview data. They were: (1) engaging through art, (2) removing the mask that hides their emotions, (3) revealing their strengths and (4) a sense of achievement.

3.1. Engaging Through Art

Body Mapping was overarchingly perceived by young people as an enjoyable, fun and valuable experience for both themselves and their peers as 'it was different' to the typical group sessions in PALM. Staff and young people reported that the residents at PALM rarely have opportunities to engage in artistic activities in the program and often

leave the talk-based sessions early or do not fully engage in them, as they reported they do not always enjoy them. This is illustrated by two of the participants, who said,

I've been here quite a long time-two and a half months I've done. We do like four groups a day and this is definitely one of the best groups I've done in my whole time here. Cause a lot of the groups aren't interactive so it's really good it was very interactive, fun and you can do your own thing. (Liam, young person (YP))

It'd be different if there wasn't an activity like this [Body Mapping] 'cause everyone, being quite frank, would just be like, 'I don't give a fuck about this. I'm just gonna walk out.' (Emma, YP)

As Body Mapping is a creative, hands-on and uniquely individual process, it was observed by the research team to pique young people's interest and engage them more than talking face to face in traditional interviews. The study team also observed that during the workshop young people were initially reserved, but once they started creating their body maps, they were focused and regularly moved between the art materials displayed on a table and their body maps, spending time considering what materials to use in different parts of their body map. Staff also commented on the increased engagement levels of the young people during the workshops, which lasted 2.5 h,

The young people stayed in a lot longer than what they normally do in a group. It's normally hard to keep them in there for like half an hour. It's good 'cause they like more hands-on stuff. So I agree with doing something physical instead of sitting there and having a conversation. It gets them moving and gets them doing stuff. (Kate, staff)

Through field observations it appeared that the exercises included at the start of the workshops helped the young people to 'warm up' and feel more at ease and engaged in the creative process when making their body maps. The hands-on process of creating their body maps also seemed to have a calming effect for those involved and it was observed that the young people did not leave the room, seemed absorbed in the process and focused on the task without needing much assistance. One of the young people told the interviewers that he had attention-deficit/hyperactivity disorder (ADHD) and then said,

It makes me feel good that I actually put effort into it and that I sort of got lost in it as well […] (Partial text removed for ease of meaning.) it stopped me over-thinking everything. I could just pay attention to that [doing the body map]. (Connor, YP)

The staff members noted they could also see a change in the young people after Body Mapping was completed,

I think it [doing Body Mapping] centred them a little actually. We [the staff] made the comment at the start that it was a bit like herding cats [to the workshop] as one would get to the table and another one would leave. I think that was a sign of their hesitation to the whole exercise. But afterwards […] I think it really did ground them a little and calmed them […] they weren't in their heads so much. (Lexi, staff)

It was also suggested that Body Mapping encouraged an experience like being "in flow," which is a positive psychological state whereby a person is fully absorbed and focused on an activity [41],

A few in the group are very hard on themselves, very harsh critics and perfectionists. So [pause] it was really interesting to see that when they got into the Body Mapping that went away a little. At first [they] were in their heads but then I think the process took over and they fell into it and threw themselves in. (Lexi, staff)

Findings from both staff and participants highlight the value of Body Mapping in engaging the young people for a substantial amount of time, helping them to be more reflective and creative, something neither group saw happening in regular sessions at PALM where the focus was on talking. Body Mapping places the participant at the centre of the creative process as both the creator and interpreter of the meaning of their body map. This enhanced ownership of the creative and meaning making processes of Body

Mapping and can empower participants to assert control in the creation and sharing of their stories [36].

3.2. Removing the Mask That Hides Their Emotions

Body Mapping was a creative, as well as a flexible and dynamic, process which appeared to facilitate the participants with processing and becoming more attuned to their emotions. The exploration and visual representation of their feelings through Body Mapping was reported to encourage a more honest depiction of emotions, compared to when verbally discussing them with others as described by participants in the interviews following the workshop,

> *I find it hard to talk to people but doing that kind of body map was a lot different than sitting face-to-face with someone and talking about yourself. (Talia, YP)*

> *People here struggle with talking about themselves and their feelings. But when doing this [Body Mapping], it kind of subconsciously goes onto the page…We're always talking, so you can get very good at hiding stuff. But creatively… it's kind of hard to like mask it out. (Liam, YP)*

By having the opportunity to express themselves through an art form as a first step, the young people claimed they felt more comfortable in disclosing a personal account of their emotions later in the interviews. One staff member said,

> *Especially for someone like Kaylee […] if you sat her in a room and asked her questions [about how she is feeling], you would have got a one-worded answer. She has real trust barrier and she's just shy. But getting her into the art stuff, she loved it. (Kate, staff)*

This increased sense of comfort could also be owing to the fact that the research team were present during the workshops, so rapport could be built with the participants prior to undertaking the interviews. Staff and participants also shared that Body Mapping offered a means for the young people to identify, externalise and communicate their feelings in a non-verbal and uniquely personal way. Furthermore, through the hidden meanings and visual symbolism which could be incorporated into their body maps, the young people felt they were able to provide a more vivid and transparent depiction of their experiences,

> *Body Mapping was a good way to get them to kind of think about it [their emotions]. And then by putting it on a piece of paper and not having to like look you in the eye and tell you these things it made it a lot easier for them [to express their emotions]. (Kate, staff)*

> *Yeah [the mask with pointy teeth drawn on my body map] that would be a representation of [me] trying to act tougher like as a protection thing and to mask my other emotions. 'Cause that's been a big thing I've been working with here at PALM, like saying that I'm not okay or saying how I feel. […] But this [Body Mapping] is kind of a bit more secretive [than talking], so you can be a bit more honest [about how you feel] (Liam, YP).*

> *Yeah [The leaves on my body map represent] my grandmother 'cause she passed away and we were both into like nature and stuff. So […] every time I draw, I don't know. I have a fascination with just drawing leaves […] And like [drawing the leaves] it reminded me of my Nan, so I just kept on drawing them. So, like, it's a symbol for me, my own symbol. (Kaylee, YP)*

The fact that the artist is the creator of the symbolism in their body map, as well as the interpreter of their meanings [26,30,32] could also have contributed to the young people feeling more at ease in sharing their emotions through their body map. Some young people also mentioned that creating art was more familiar than talking about their feelings, reminding them of the positive experiences they had had in school or at home when they had done art,

I did heaps of artwork as a kid but I [have] sort of have lost that. So I started looking back and going, "Oh yeah. I do have a bit of a creative mind, I can do those things." [...] It was fun to get back into that and it brought back cool memories of days as a kid back at my school, like the days I really enjoyed. (Connor, YP)

As shown above, it may be that activities like Body Mapping that are similar to younger childhood experiences of doing art can evoke more positive emotions and a sense of comfort, encouraging the expression of feelings about oneself in the present in a more relaxed setting. This also could be because expressing oneself through pictures could be a more accessible and comfortable medium of expression than verbal language.

The study found that Body Mapping was a valuable vehicle for participants to tap into their emotions. By enabling them to feel more comfortable and giving them the freedom to express themselves visually, Body Mapping seemed to lessen the need for them to 'bottle' or 'mask' their emotions compared to when only required to talk about them.

3.3. Revealing Their Strengths

The participants described how being engaged in the activity and by becoming more emotionally attuned through the Body Mapping process, they were also able to recognise and reveal their strengths. The majority of the participants mentioned that negative self-talk and poor self-esteem pervades their thoughts and view of themselves. Yet, the strengths-based focus of the Body Mapping exercise appeared to challenge their common default of negative thought patterns and helped to encourage a more positive mindset. One participant said,

I've had a really difficult upbringing. So it was hard [...] 'Cause I've never had more of a positive outlook on life [...] So yeah. It was a really, really good outcome because I did think more positively after that. (Emma, YP)

The staff also indicated how the reflective and introspective process of creating a visual body map seemed to promote a more positive inner dialogue for the young people involved,

They can't really acknowledge any strengths that they hold [...] and if we were to sit there and ask them, "What's good about you and what do you like about yourself?" they literally wouldn't have been able to say anything. But, once they got their mind into the artwork, they did. (Kate, staff)

They find it hard to talk about their strengths or anything they are good at [...] This [Body Mapping] definitely brought it [their strengths] out of them in a different way. I struggle a lot with this with clients, it's a recurring theme. I try to end on, "Ok tell me something that is good about you" [in my counseling sessions] but they can't think of one thing [...] but looking around at the maps, I thought, "Wow." This is a side of you that they probably have never really thought about or they had even expressed to their counsellors or anyone ever before. (Emily, staff)

Through the interviews, it became evident that the Body Mapping process enabled the young people to more easily recognise and communicate a more positive, strengths-based narrative about themselves. Three examples of the participant's body maps are included in Figure 3 to exemplify that Body Mapping facilitated a dialogue on their personal strengths and supports in the interviews.

The staff interviews also clearly suggested that the strengths-based narratives that emerged via the body maps would not have been accessible through solely interview-based techniques and thus Body Mapping enriched the quality of the data we obtained,

I think a different side came out, that if we had asked the question [about strengths and sources of support] verbally we could not have gotten that answer. It was a way of getting them to express it and getting it out in a way that we wouldn't have gotten from a verbal question. So it was really valuable. Really rich. (Lexi, staff)

These positive responses from both staff and participants attest to the utility and effectiveness of Body Mapping as a facilitation tool for these young participants to share their character strengths and support systems.

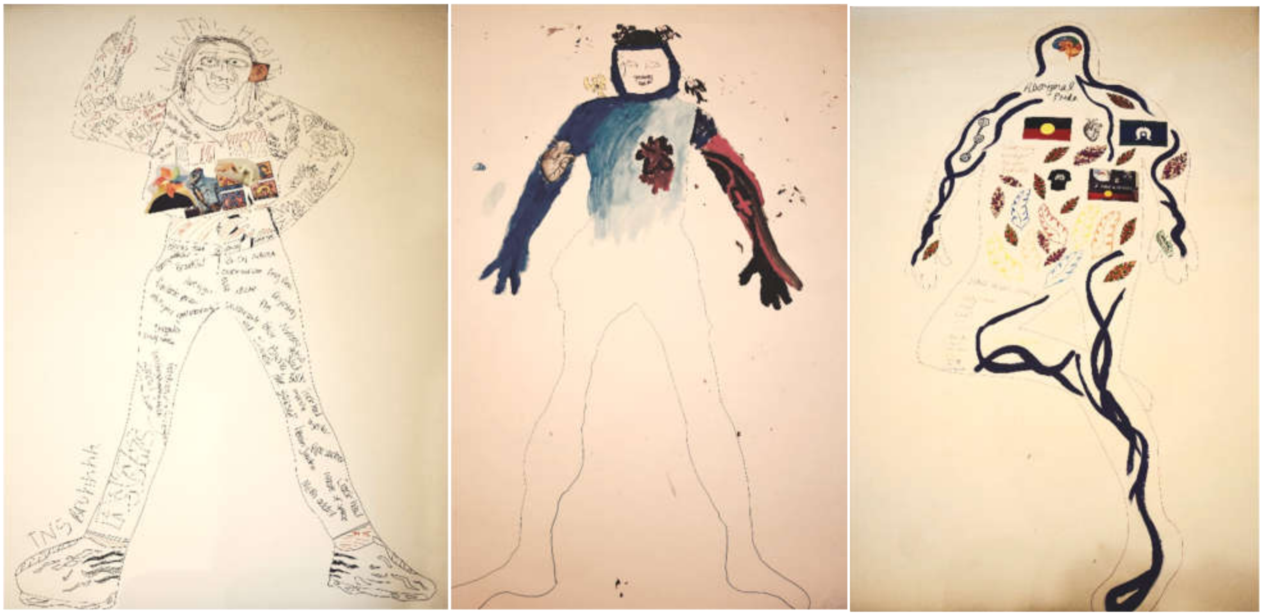

(**a**) (**b**) (**c**)

Figure 3. Examples of Body Maps: (**a**) Emma's Body Map—Emma described how when she initially started her Body Map, she was in a very negative mindset, so she wrote negative words and illustrated her previous drug use on the right side of her body. However, through the Body Mapping process, she was able to start thinking more positively and was able to include more positive words and the family members and friends who have supported her on the other side of her Body Map. (**b**) Liam's Body Map—Liam expressed how the blue colour included in his Body Map represented his love for swimming, which is an activity he used to enjoy with his family and is hoping to get back into after he graduates from PALM. It was also used to symbolize the increasing inner strength he has gained through PALM and how this is helping him to gradually overcome his vices, which are manifested by his red/black arm. Spirituality also provided Liam with a lot of support and strength and is illustrated by an angel on his 'strong' (blue) shoulder. (**c**) Kaylee's Body Map—Kaylee discussed how Body Mapping helped her open up about the support she receives from her family, who she usually does not talk about much. The leaves she included on her Body Map were used to symbolize her Nan who had been a big part of her life but passed away. Kaylee mentioned that Body Mapping also helped her feel more in touch with her culture and the process prompted her to include her tribes on her map and other cultural references like an Aboriginal dot painting.

3.4. A Sense of Achievement

Both young people and staff acknowledged that as a finished product, the body map itself was something that young people took pride in,

> *Four months ago it would have been like a very different picture [referring to his body map]. It probably [would have been] more darker-looking. I feel happy and accomplished that when I did do it, I could colour in most of it [the body map] blue [symbolising his inner strength]. (Liam, YP)*

> *"One of the girls, who isn't even my client, came up to me afterwards, and was like, "Come down and see my body map! See what I've done!" […] she really wanted that validation as she was quite proud of it [her body map]. And she is such a quiet person too. She was explaining what she did and was just so proud and so excited about it." (Emily, staff)*

For the young people, creating a body map focusing on their strengths appeared to provide them with a sense of achievement. Many participants mentioned how they

wanted to put their map up on their wall and show it to their family. For some, looking at their body map also provided further motivation to continue in treatment:

> *When I arrived, I didn't really participate in any of the groups and stuff. But I think if I'd done this [Body Mapping] early on when I came I would have like not think about going [leaving the program] 'cause like it like talks to you, the picture, like you can do it, this is what you're here for. (Kaylee, YP)*

> *It's [my body map is] kind of like a good visual reminder […] of where I was and where I am [now] […] it's nice to see I'm on the road to recovery. (Liam, YP)*

Both young people and staff reflected that Body Mapping was a valuable experience and one which promoted feelings of pride and accomplishment amongst participants. By stepping back and being able to see their strengths and support systems visually depicted on a single page, the photo of their body map seemed to enable participants to recognise their progress and possibly serve as a motivator for them to continue through the treatment program.

4. Discussion

In this study, we found that there was potential to utilize Body Mapping as a data collection method within research in residential AOD treatment for adolescents to derive richer data and access new narratives not usually captured in more traditional qualitative methods. Body Mapping was found to have engaged and focused the young participants in a task and provided a creative platform for residents to express their strengths and supports, a subject recognised by staff and participants as challenging for them to discuss verbally. The creative process of crafting a body map, allowed each participant to approach it in their own way and have ownership on how their story was conveyed. Furthermore, the reflective processing and introspection that Body Mapping facilitated, provided an opportunity for the participants to recognise their growth throughout the program, generating a sense of pride and accomplishment. Another unintended benefit of this study was that the staff involved in the workshops gained valuable insights into the process and were reportedly interested in promoting the inclusion of Body Mapping into PALM as a counselling tool. Whilst the therapeutic benefits of Body Mapping were not formally evaluated in this study, it appears to be a suitable topic for further research.

Body Mapping has previously been employed as a qualitative research method with other select populations of varying ages and on different issues [34]. Several of our key findings align with the existing body of literature on the benefits of Body Mapping as a data collection tool [36], supporting its utility in AOD treatment settings. First, Body Mapping is an innovative method which is able to elicit rich data around a central theme by engaging participants, encouraging reflection through creative practice and facilitating agency in how to represent themselves [25,26,36,42–44]. Second, Body Mapping can aid in capturing the complexity of lived experiences through facilitating the gradual process of layering and incorporating symbolic representation into the artwork, allowing the participants time to carefully craft their story, compared to more traditional and immediate responses expected in interviews [36]. Third, several studies have found Body Mapping to be an approach which boosted the morale, self-esteem and perceived self-worth of participants by enabling greater recognition of one's growth [25,26,36,37,43] as we found in this study. Finally, Body Mapping was found to illuminate and champion the personal narratives and embodied experiences of participants through visual representation 38,42,44].

However, our study differs from many previous studies in that it focused less on the specific strengths-based content young people included in their maps, and more on how Body Mapping facilitated discussions on a subject matter that is normally challenging for young people to talk about. Additionally, our study highlights the potential applicability and utility of Body Mapping as a tool to improve understanding of the lives and experiences of adolescents undergoing AOD residential treatment and as a potential additional

tool for therapeutic engagement which could potentially reduce drop out. Leaving treatment early and not engaging in treatment are major challenges in the youth residential AOD treatment field [16–18,21].

Determining ways of improving insight into young people's perspectives, emotions and experiences during treatment is of value to service providers in improving the acceptability and overall effectiveness of treatment programs. Whilst our findings do not provide an evaluation of the treatment program, nor the methods currently implemented at PALM, they suggest that using more arts-based activities like Body Mapping within the program could provide a more engaging and creative outlet for young people to express themselves, which could generate conversations, pinpoint areas to address during treatment and thereby improve their treatment engagement and overall experience. This study has demonstrated that Body Mapping has the potential to capture rich qualitative data when utilized as a research tool in this context, shedding further light on the adolescent experience in residential treatment programs.

4.1. Implications

Visual methods such as Body Mapping hold promise for working with younger participants in AOD treatment and other research settings as they appear to be engaging and less confrontational form of data collection compared with interviews alone. Body Mapping is a useful tool to help start conversations on topics which participants find hard to verbalise face to face. The potential therapeutic benefits of Body Mapping for this population also warrant investigation. Further research may reveal the utility of Body Mapping as a therapeutic or counselling tool within the PALM residential treatment program or other similar TCs.

4.2. Limitations and Challenges

We acknowledge the limitations inherent in our study. As only two Body Mapping workshops were held, this limited the number of participants recruited, and possibly reduced the range of experiences that could have been explored. As is the case with many qualitative studies, this study was exploratory to generate hypotheses for further testing. A systematic review (PROSPERO registration CRD42020161675) conducted on arts-based methods by author Nathan with young people with complex needs found sample sizes in arts-based studies (n = 26) with young people with complex needs ranged from just a few to 33 participants, with many having between 6 and 12 participants at a single site. These studies produced rich data collected to inform their research questions as with the current study. The Body Maps presented a snapshot of participant's thoughts and feelings at a single point in time, rather than across the program. Future studies might consider doing a series of body maps during the young person's stay in treatment to capture the changes in their visualisations of their strengths and supports as they progress further through the program.

5. Conclusions

Body Mapping has potential to be a valuable data-generating research tool with young people undergoing residential treatment for AOD issues. This technique engaged young people, facilitated a transparent expression of their thoughts and feelings and stimulated dialogue about their strengths and support systems, all of which staff and participants attested could not have been achieved through interview-based approaches alone. The combined visual and verbal media facilitated a more holistic understanding of the young people's perspectives and treatment experience, which, with further research, could help improve the service delivery of youth residential treatment programs to optimise treatment engagement and retention.

Author Contributions: Conceptualization, S.M., S.N., M.J., K.M.B., and A.G.; methodology, S.M., S.N., M.J., K.M.B., and A.G.; data curation/interviews, S.M. and A.G.; writing—original draft preparation, S.M. and S.N.; writing—review and editing, S.M., S.N., M.J., K.M.B., and A.G. All authors have read and agreed to the published version of the manuscript.

Funding: This research was supported by an Australian Research Council Linkage Projects funding grant (LP140100429), a seed grant from the School of Population Health at UNSW Sydney and the Ted Noffs Foundation.

Institutional Review Board Statement: This study was approved by the Aboriginal Health and Medical Research Council of NSW, Australia (1062/14) on 27 January 2015 with the modification to include Body Mapping approved 12 March 2018.

Informed Consent Statement: Informed written consent was obtained from all participants involved in this study.

Data Availability Statement: This publication has used qualitative data that in its full form could be identifiable and researchers do not have permission to disclose the full data set and transcripts to any person other than those authorised for the research project.

Acknowledgments: We acknowledge and thank the young people and staff who participated in the workshops and interviews.

Conflicts of Interest: The authors declare no conflict of interest.

Appendix A

Table A1. Workshop Run Sheet and Activities.

Time	Details
8:30 a.m.	**Facilitators arrived to set up art supplies**
10:00 a.m.	**PALM residents and staff arrived–Welcome & Introductions**<br>**Arrival:** Welcoming of artists, giving out of name tags and allowing for 'orientation in room'
10:10 a.m.	**Coming together–Explanation of process/purpose of workshops**<br>**Introductions and ground rules:** Discussed workshop aims–focusing on strengths and sources of support. Informed that they don't have to be an artist and how this is a very personal process so they can take breaks with snacks/step out whenever they need. Provide overview of what Body Mapping is.
	**Explanation of the research focus explained:**<br>Talked about research being undertaken at Noffs and interest in getting feedback on the process of Body Mapping rather than on the content of their maps.
	**Consent:** Briefly and simply talked through consent form, discuss importance of it and confirmed that everyone has filled out the forms/handed them in before starting. Discussed recording group discussion at end and photos of the process taken throughout.
10:15 a.m.	**'Choose an object'—**Asked to select one of the objects (e.g., a rubber duck, beaded elephant, mask, bible etc.). on the table and discuss why it related to them. Designed to get participants to think about the deeper, personal meanings that can be attached to certain symbols or objects and start thinking of how to represent themselves creatively.
10:25 a.m.	**Creative exercise—**Non-dominant hand continuous line drawing of face. Designed to equalise everyone's drawing abilities and reduce feelings of apprehension about expressing themselves creatively or worrying about being a perfectionist. Asked to describe why they chose the colour they did to start thinking about how colours can also represent their thoughts and feelings.
10:40 a.m.	**Body tracing** (with facilitators or each other)<br>Encouraged removing bulky clothing, choosing their pose, and getting a partner or facilitators to help draw pose. * NB a body relaxation exercise of trialing different poses as a group in a circle was incorporated into the second session as the participants seemed a little more hesitant and anxious initially about how to approach Body Mapping.
11:00 a.m.	**Working on Body Maps**

	Time provided for artist to fill their Body Map with images/symbols/words which reflect strengths/sources of support using various art materials.
12:10 p.m.	**Group discussion**<br>Script:<br>"Now that we have finished, we would just like to take some time to get your feedback on today.<br>—How did you find doing this activity?<br>—Did anything surprise/excite/challenge you about creating a Body Map?<br>—What did you think before doing this activity?<br>If anyone would be willing and comfortable to share something about their Body Map with us, we would really appreciate it. Like which part you liked the most about the workshop/creating a body map/your own body map and why? How it made you think about your strengths?<br>During lunch we would like to have the opportunity to wander around and enjoy everyone's creative efforts. If you don't feel comfortable with others seeing it you can roll it up."
12:25 p.m.	**Wrap up + photos of maps**<br>Explanation that their counsellor will follow up with them and interviews will be held sometime next week. Photographs of their body maps took place during lunch by a member of the research team.
12:30 p.m.	**Workshop completion**<br>Lunch supplied for all participants, facilitators, and staff.
12:45 p.m.	**Pack up + team discussion & de-brief**

References

1. Ritter, A.; King, T.; Hamilton, M. *Drug Use in Australian Society*; Oxford University Press: Oxford, UK, 2013.
2. United Nations Office on Drugs and Crime. *World Drug Report*; United Nations Publication: Herndon, VA, USA, 2017; No. E. 17.
3. American Psychiatric Association. *Diagnostic and Statistical Manual of Mental Disorders: DSM-4*, 4th ed.; American Psychiatric Association: Washington, DC, USA, 2000.
4. American Psychiatric Association. *Diagnostic and Statistical Manual of Mental Disorders: DSM-5*, 5th ed.; American Psychiatric Association: Washington, DC, USA, 2013.
5. AIHW. *2016 National Drug Strategy Household Survey Report*; AIHW: Canberra, Australia, 2017.
6. Mitchell, S.G.; Gryczynski, J.; O'Grady, K.E.; Schwartz, R.P. SBIRT for adolescent drug and alcohol use: Current status and future directions. *J. Subst. Abus. Treat.* **2013**, *44*, 463–472.
7. Sawyer, S.M.; Afifi, R.A.; Bearinger, L.H.; Blakemore, S.-J.; Dick, B.; Ezeh, A.C.; Patton, G.C. Adolescence: A foundation for future health. *Lancet* **2012**, *379*, 1630–1640.
8. Hingson, R.W.; Zha, W. Age of Drinking Onset, Alcohol Use Disorders, Frequent Heavy Drinking, and Unintentionally Injuring Oneself and Others After Drinking. *Pediatrics* **2009**, *123*, 1477–1484.
9. Kelly, A.B.; Evans-Whipp, T.J.; Smith, R.; Chan, G.C.; Toumbourou, J.W.; Patton, G.C.; Hemphill, S.A.; Hall, W.D.; Catalano, R.F. A longitudinal study of the association of adolescent polydrug use, alcohol use and high school non-completion. *Addiction* **2015**, *110*, 627–635.
10. Dixson, G.; Woolfenden, S.; Jayasinha, R.; Rawstorne, P.; Palmer, K.; Nathan, S. Characteristics of Australian adolescent females in residential treatment who report using methamphetamines. *Drug Alcohol Rev.* **2017**, *37*, doi:10.1111/dar.12641.
11. De Leon, G.; Wexler, H. The Therapeutic Community for Addictions: An Evolving Knowledge Base. *J. Drug Issues* **2009**, *39*, 167–177.
12. Nathan, S.; Maru, K.; Williams, M.; Palmer, K.; Rawstorne, P. Koori voices: Self-harm, suicide attempts, arrests and substance use among Aboriginal and Torres Strait Islander adolescents following residential treatment. *Health Justice* **2020**, *8*, 4.
13. Darke, S.; Campbell, G.; Popple, G. Retention, early dropout and treatment completion among therapeutic community admissions. *Drug Alcohol Rev.* **2012**, *31*, 64–71.
14. Neumann, A.; Ojong, T.N.; Yanes, P.K.; Tumiel-Berhalter, L.; Daigler, G.E.; Blondell, R.D. Differences between adolescents who complete and fail to complete residential substance abuse treatment. *J. Addict. Dis.* **2010**, *29*, 427–435.
15. Chan, K.S.; Wenzel, S.; Orlando, M.; Montagnet, C.; Mandell, W.; Becker, K.; Ebener, P. How important are client characteristics to understanding treatment process in the therapeutic community? *Am. J. Drug Alcohol Abus.* **2004**, *30*, 871–891.
16. Chen, G.; Elisha, E.; Timor, U.; Ronel, N. Why Do Adolescents Drop Out of a Therapeutic Community for People With Drug Addiction? *J. Child Adolesc. Subst. Abus.* **2016**, *25*, 65–77.
17. Edelen, M.O.; Slaughter, M.E.; McCaffrey, D.F.; Becker, K.; Morral, A.R. Long-term effect of community-based treatment: Evidence from the adolescent outcomes project. *Drug Alcohol Depend.* **2010**, *107*, 62–68.
18. Gunter, W.D.; Abdel-Salam, S. Therapeutic engagement and posttreatment substance use in adolescent TC clients: Direct and indirect effects. *J. Drug Issues* **2014**, *44*, 22–36.

19. Mills, L.; Pepler, D.; Cribbie, R.A. Effectiveness of residential treatment for substance abusing youth: Benefits of the Pine River Institute program. *Resid. Treat. Child. Youth* **2013**, *30*, 202–226.
20. de Andrade, D.; Elphinston, R.A.; Quinn, C.; Allan, J.; Hides, L. The effectiveness of residential treatment services for individuals with substance use disorders: A systematic review. *Drug Alcohol Depend.* **2019**, *201*, 227–235.
21. Winters, K.C. Treating adolescents with substance use disorders: An overview of practice issues and treatment outcome. *Subst. Abus.* **1999**, *20*, 203–225.
22. Winters, K.C.; Stinchfield, R.D.; Opland, E.; Weller, C.; Latimer, W.W. The effectiveness of the Minnesota Model approach in the treatment of adolescent drug abusers. *Addiction* **2000**, *95*, 601–612.
23. Davidson, K.M.; Young, J.T.N. Treatment engagement in a prison-based Therapeutic Community: A mixed-methods approach. *J. Subst. Abus. Treat.* **2019**, *103*, 33–42.
24. Foster, M.; Nathan, S.; Ferry, M. The experience of drug-dependent adolescents in a therapeutic community, *Drug Alcohol Rev.* **2010**, 531–539, doi:10.1111/j.1465-3362.2010.00169.x.
25. Lys, C.; Gesink, D.; Strike, C.; Larkin, J. Body Mapping as a Youth Sexual Health Intervention and Data Collection Tool. *Qual. Health Res.* **2018**, *28*, 1049732317750862.
26. Dew, A.; Smith, L.; Collings, S.; Dillon Savage, I. Complexity Embodied: Using Body Mapping to Understand Complex Support Needs. *FQS* **2018**, *19*, doi:10.17169/fqs-19.2.2929.
27. Boydell, K.; Cheng, C.; Gladstone, B.; Nadin, S.; Stasiulis, E. Co-Producing Narratives on Access to Care in Rural Communities: Using Digital Storytelling to Foster Social Inclusion of Young People Experiencing Psychosis (Dispatch). *Stud. Soc. Justice* **2018**, *11*, 298.
28. de Jager, A.; Fogarty, A.; Tewson, A.; Lenette, C.; Boydell, K.M. Digital storytelling in research: A systematic review. *Qual. Rep.* **2017**, *22*, 2548–2582.
29. Bates, E.A.; McCann, J.J.; Kaye, L.K.; Taylor, J.C. "Beyond words": A researcher's guide to using photo elicitation in psychology. *Qual. Res. Psychol.* **2017**, *14*, 459–481.
30. Hodgins, M.J.; Boydell, K.M. Interrogating ourselves: Reflections on arts-based health research. *Forum Qual. Soz. Forum Qual. Soc. Res.* **2013**, *15*, doi:10.17169/fqs-15.1.2018.
31. D'Amico, M.; Denov, M.; Khan, F.; Linds, W.; Akesson, B. Research as intervention? Exploring the health and well-being of children and youth facing global adversity through participatory visual methods. *Glob. Public Res.* **2016**, *11*, 528–545.
32. Boydell, K.M.; Hodgins, M.; Gladstone, B.M.; Stasiulis, E.; Belliveau, G.; Cheu, H.; Kontos, P.; Parsons, J. Arts-based health research and academic legitimacy: Transcending hegemonic conventions. *Qual. Res.* **2016**, *16*, 681–700.
33. Boydell, K.M.; Hodgins, M.J.; Gladstone, B.M.; Stasiulis, E. Ineffable knowledge: Tensions (and solutions) in art-based research representation and dissemination. *J. Appl. Arts Health* **2017**, *8*, 193–207.
34. Rydzik, A.; Pritchard, A.; Morgan, N.; Sedgley, D. The potential of arts-based transformative research. *Ann. Tour. Res.* **2013**, *40*, 283–305.
35. Huss, E.; Braun-Lewensohn, O.; Ganayiem, H. Using arts-based research to access sense of coherence in marginalised indigenous Bedouin youth. *Int. J. Psychol.* **2018**, *53*, 64–71.
36. Boydell, K.E. *Applying Body Mapping in Research*; Routledge: London, UK, 2021.
37. Gastaldo, D.; Magalhães, L.; Carrasco, C.; Davy, C. *Body-Map Storytelling as Research: Methodological Considerations for Telling the Stories of Undocumented Workers through Body Mapping*; Creative Commons: Toronto, ON, Canada, 2012.
38. Davy, C.; Magalhães, L.V.; Mandich, A.; Galheigo, S.M. Aspects of the resilience and settlement of refugee youth: A narrative study using body maps/Aspectos da resiliência e da integração social de jovens refugiados: Um estudo narrativo usando mapas corporais. *Cad. Bras. Ter. Ocup.* **2014**, *22*, doi:10.4322/cto.2014.045.
39. Vasquez, G. *Body Perceptions of HIV and AIDS: The Memory Box Project*; University of Cape Town: Cape Town, Sotuh Africa, 2004. Available from: http://hdl.handle.net/11427/19177 (acessed on 3 May 2019).
40. Terry, G.; Hayfield, N.; Clarke, V.; Braun, V. Thematic analysis. In *The Sage Handbook of Qualitative Research in Psychology*; SAGE Publications: NewYork, NY, USA, 2017; pp. 17–37.
41. Nakamura, J.; Csikszentmihalyi, M. The concept of flow. In *Flow and the Foundations of Positive Psychology*; Springer: Berlin, Germany, 2014; pp. 239–263.
42. Brett-MacLean, P. Body mapping: Embodying the self living with HIV/AIDS. *Can. Med Assoc. J.* **2009**, *180*, 740–741.
43. Orchard, T.; Smith, T.; Michelow, W.; Salters, K.; Hogg, B. Imagining adherence: Body mapping research with HIV-positive men and women in Canada. *AIDS Res. Hum. Retrovir.* **2014**, *30*, 337–338.
44. Ludlow, B.A. Witnessing: Creating visual research memos about patient experiences of body mapping in a dialysis unit. *Am. J. Kidney Dis.* **2014**, *64*, A13–A14.
