# Supplementary material for: Estimation of Undetected Asymptomatic COVID-19 Cases in South Korea Using a Probabilistic Model
Source: Int J Environ Res Public Health. 2021 May 6;18(9):4946. doi: 10.3390/ijerph18094946 (PMC8124955; doi:10.3390/ijerph18094946)
Supplement: Supplementary file 1 [file ijerph-18-04946-s001.zip › COVID-asymptomatic-supplementary.pdf]

## Supplementary material

Here, we provide detailed derivation of factorized probabilities from the joint distribution P (D, Sy, Sc, T) in **Supplementary Table S1.** In the derivation, we assumed PPV =1, namely, P (D = 1|T = 1) = 1 and NPV = 1 (P (D = 0|T = 0) = 1). We let each row of the table to be indexed with numbers for clear understanding.

| Index | Disease | Symptoms | Screening | Test Result | Factorized Probabilities |
|---|---|---|---|---|---|
| (1) | 1 | 1 | 1 | 1 | $P(Sy = 1\|T = 1) * P(T = 1)$ |
| (2) | 1 | 1 | 1 | 0 | 0 |
| (3) | 1 | 1 | 0 | - | $\frac{\delta_2}{\delta_1} * \frac{P(Sc=0)}{P(Sc=1)} * P(Sy = 1\|T = 1) * P(T = 1)$ |
| (4) | 1 | 0 | 1 | 1 | $P(Sy = 0\|T = 1) * P(T = 1)$ |
| (5) | 1 | 0 | 1 | 0 | 0 |
| (6) | 1 | 0 | 0 | - | $\frac{\delta_2}{\delta_1} * \frac{P(Sc=0)}{P(Sc=1)} * P(Sy = 1\|T = 0) * P(T = 0)$ |
| (7) | 0 | 1 | 1 | 1 | 0 |
| (8) | 0 | 1 | 1 | 0 | $P(Sy = 1\|T = 0) * P(T = 0)$ |
| (9) | 0 | 1 | 0 | - | $\frac{\delta_2}{\delta_1} * \frac{P(Sc=0)}{P(Sc=1)} * P(Sy = 0\|T = 0) * P(T = 1)$ |
| (10) | 0 | 0 | 1 | 1 | 0 |
| (11) | 0 | 0 | 1 | 0 | $P(Sy = 0\|T = 0) * P(T = 0)$ |
| (12) | 0 | 0 | 0 | - | $\frac{\delta_2}{\delta_1} * \frac{P(Sc=0)}{P(Sc=1)} * P(Sy = 0\|T = 0) * P(T = 0)$ |

**Supplementary Table S1.** Derivation of the joint distribution P (D, Sy, Sc, T) using chain rule of probability.

(1) $P(D=1, Sy=1, Sc = 1, T = 1) = P( Sy =1| T = 1) P(T = 1)$

$$\begin{aligned} P(D=1, Sy=1, Sc = 1, T = 1) &= P(D = 1, Sy =1, Sc = 1| T = 1) P(T = 1) \\ &= P(D = 1, Sy =1| T = 1) P(T = 1) \\ &= P(Sy =1| D = 1, T = 1) P(D = 1|T = 1) P(T = 1) \\ &= P(Sy =1| T = 1) P(T = 1) \end{aligned}$$

(2) $P(D=1, Sy=1, Sc = 1, T = 0) = 0$

$$P(D=1, Sy=1, Sc = 1, T = 0) = P(D = 1, Sy =1, Sc = 1| T = 0) P(T = 0)=0$$

$$\because P(D = 0|T = 0) = 1,\ P(D = 1|T = 0) = 0$$

(3) $P(D=1, Sy=1, Sc=0) = \frac{\delta_2}{\delta_1} * \frac{P(Sc=0)}{P(Sc=1)} * P(Sy=1|T=1) * P(T=1)$

$$P(D=1, Sy=1, Sc=0) = P(D=1, Sy=1|\ Sc=0)P(Sc=0)$$

$$= \frac{P(D=1, Sy=0|Sc=0)}{P(D=1, Sy=0|Sc=1)}\ P(D=1, Sy=1|\ Sc=1)P(Sc=0)$$

$$= \frac{P(D=1, Sy=0|Sc=0)}{P(D=1, Sy=0|Sc=1)}\ \frac{P(D=1, Sy=1, Sc=1)}{P(Sc=1)}\ P(Sc=0)$$

$$= \frac{P(D=1, Sy=0|Sc=0)}{P(D=1, Sy=0|Sc=1)}\ \frac{P(Sc=0)}{P(Sc=1)}\ P(D=1, Sy=1, Sc=1)$$

$$= \frac{P(D=1, Sy=0|Sc=0)}{P(D=1, Sy=0|Sc=1)}\ \frac{P(Sc=0)}{P(Sc=1)}\ \{P(D=1, Sy=1, Sc=1, T=0)+P(D=1, Sy=1, Sc=1, T=1)\}$$

$$= \frac{P(D=1, Sy=0|Sc=0)}{P(D=1, Sy=0|Sc=1)}\frac{P(Sc=0)}{P(Sc=1)}\{0+P(D=1, Sy=1, Sc=1, T=1)\}$$

$$= \frac{P(D=1, Sy=0|Sc=0)}{P(D=1, Sy=0|Sc=1)}\frac{P(Sc=0)}{P(Sc=1)}P(Sy=1|\ T=1)\ P(T=1)$$

$$= \frac{\delta_2}{\delta_1} * \frac{P(Sc=0)}{P(Sc=1)} * P(Sy=1|T=1) * P(T=1)$$

(4) $P(D=1, Sy=0, Sc=1, T=1) = P(Sy=0|\ T=1)(T=1)$

$$P(D=1, Sy=0, Sc=1, T=1) = P(D=1, Sy=0, Sc=1|\ T=1)\ P(T=1)$$

$$= P(D=1, Sy=0|\ T=1)P(T=1)$$

$$= P(Sy=0|\ D=1, T=1)P(D=1|T=1)P(T=1)$$

$$= P(Sy=0|\ T=1)(T=1)$$

(5) $P(D=1, Sy=0, Sc=1, T=0)= 0$

$$P(D=1, Sy=0, Sc=1, T=0)= P(D=1, Sy=0, Sc=1|\ T=0)P(T=0)=0$$

$$\because P(D=0|T=0) = 1,\ P(D=1|T=0) = 0$$

(6) $P(D=1, Sy=0, Sc=0) = \frac{\delta_2}{\delta_1} * \frac{P(Sc=0)}{P(Sc=1)} * P(Sy=0|\ T=1)P(T=1)$

$$P(D=1, Sy=0, Sc=0)= P(D=1, Sy=0|\ Sc=0)P(Sc=0)$$

$$= \frac{P(D=1, Sy=0|Sc=0)}{P(D=1, Sy=0|Sc=1)}P(D=1, Sy=0|\ Sc=1)P(Sc=0)$$

$$= \frac{P(D=1, Sy=0|Sc=0)}{P(D=1, Sy=0|Sc=1)}\frac{P(D=1, Sy=0, Sc=1)}{P(Sc=1)}\ P(Sc=0)$$

$$= \frac{P(D=1, Sy=0|Sc=0)}{P(D=1, Sy=0|Sc=1)}\frac{P(Sc=0)}{P(Sc=1)}\ P(D=1, Sy=0, Sc=1)$$

$$= \frac{P(D=1, Sy=0|Sc=0)}{P(D=1, Sy=0|Sc=1)}\frac{P(Sc=0)}{P(Sc=1)}\{P(D=1, Sy=0, Sc=1, T=0)+P(D=1, Sy=0, Sc=1, T=1)\}$$

$$= \frac{P(D = 1, Sy=0|Sc =0)}{P(D = 1, Sy=0|Sc =1)} \frac{P(Sc=0)}{P(Sc=1)} \{0+P(D = 1, Sy = 0, Sc=1, T = 1)\}$$

$$= \frac{P(D = 1, Sy=0|Sc =0)}{P(D = 1, Sy=0|Sc =1)} \frac{P(Sc=0)}{P(Sc=1)} P(Sy =0| T = 1) P(T = 1)$$

(7) $P(D=0, Sy=1, Sc = 1, T = 1)= 0$

$$P(D=0, Sy=1, Sc = 1, T = 1)= P(D = 0, Sy =0, Sc = 1| T = 1)P(T = 1)=0$$

$$\because P(D = 0|T = 1) = 0,\ P(D = 1|T = 1) = 1$$

(8) $P(D = 0, Sy =1, Sc = 1, T = 0) = P(Sy =1| T = 0)P(T = 0)$

$$P(D=0, Sy=1, Sc = 1, T = 0) = P(D = 0, Sy =1, Sc = 1| T = 0) P(T = 0)$$

$$= P(D = 0, Sy =1| T = 0)P(T = 0)$$

$$= P(Sy =1| D = 0, T = 0)P(D = 0|T = 0)P(T = 0)$$

$$= P(Sy =1| T = 0)P(T = 0)$$

(9) $P(D = 0, Sy =1, Sc = 0) =\frac{P(D = 1, Sy=0|Sc =0)}{P(D = 1, Sy=0|Sc =1)} \frac{P(Sc=0)}{P(Sc=1)} P(Sy =1| T = 0)P(T = 0)$

$$P(D = 0, Sy =1, Sc = 0) = P(D = 0, Sy =1| Sc = 0)P(Sc = 0)$$

$$= \frac{P(D = 1, Sy=0|Sc =0)}{P(D = 1, Sy=0|Sc =1)} P(D = 0, Sy =1| Sc = 1)P(Sc = 0)$$

$$= \frac{P(D = 1, Sy=0|Sc =0)}{P(D = 1, Sy=0|Sc =1)} \frac{P(D=0, Sy=1,Sc=1)}{P(Sc=1)} P(Sc = 0)$$

$$= \frac{P(D = 1, Sy=0|Sc =0)}{P(D = 1, Sy=0|Sc =1)} \frac{P(Sc=0)}{P(Sc=1)} P(D = 0, Sy = 1, Sc=1)$$

$$= \frac{P(D = 1, Sy=0|Sc =0)}{P(D = 1, Sy=0|Sc =1)} \frac{P(Sc=0)}{P(Sc=1)} \{P(D = 0, Sy = 1, Sc=1, T = 0)+P(D = 0, Sy = 1, Sc=1, T = 1)\}$$

$$= \frac{P(D = 1, Sy=0|Sc =0)}{P(D = 1, Sy=0|Sc =1)} \frac{P(Sc=0)}{P(Sc=1)} \{P(D = 0, Sy = 1, Sc=1, T = 0)+0\}$$

$$= \frac{P(D = 1, Sy=0|Sc =0)}{P(D = 1, Sy=0|Sc =1)} \frac{P(Sc=0)}{P(Sc=1)} P(Sy =1| T = 0) P(T = 0)$$

(10) $P(D=0, Sy=0, Sc = 1,T = 1)= 0$

$$P(D=0, Sy=0, Sc = 1, T = 1)= P(D = 0, Sy =0, Sc = 1| T = 1) P(T = 0)=0$$

$$\because P(D = 0|T = 1) = 0,\ P(D = 1|T = 1) = 1$$

(11) $P(D = 0, Sy =0, Sc = 1, T = 0) = P(Sy =0| T = 0)P(T = 0)$

$$P(D=0, Sy=0, Sc = 1, T = 0)= P(D = 0, Sy =0, Sc = 1| T = 0) P(T = 0)$$

$$= P(D = 0, Sy = 0 | T = 0)P(T = 0)$$

$$= P(Sy = 0 | D = 0, T = 0)P(D = 0 | T = 0)P(T = 0)$$

$$= P(Sy = 0 | T = 0)P(T = 0)$$

(12) $P(D = 0, Sy = 0, Sc = 0) = \frac{P(D = 1, Sy=0|Sc = 0)}{P(D = 1, Sy=0|Sc = 1)} \frac{P(Sc=0)}{P(Sc=1)} P(Sy = 0 | T = 0)P(T = 0)$

$$P(D = 0, Sy = 0, Sc = 0) = P(D = 0, Sy = 0 | Sc = 0)P(Sc = 0)$$

$$= \frac{P(D = 1, Sy=0|Sc = 0)}{P(D = 1, Sy=0|Sc = 1)} P(D = 0, Sy = 0 | Sc = 1)P(Sc = 0)$$

$$= \frac{P(D = 1, Sy=0|Sc = 0)}{P(D = 1, Sy=0|Sc = 1)} \frac{P(D=0, Sy=0, Sc=1)}{P(Sc=1)} P(Sc = 0)$$

$$= \frac{P(D = 1, Sy=0|Sc = 0)}{P(D = 1, Sy=0|Sc = 1)} \frac{P(Sc=0)}{P(Sc=1)} P(D = 0, Sy = 0, Sc=1)$$

$$= \frac{P(D = 1, Sy=0|Sc = 0)}{P(D = 1, Sy=0|Sc = 1)} \frac{P(Sc=0)}{P(Sc=1)} \{P(D = 0, Sy = 0, Sc=1, T = 0)+P(D = 0, Sy = 0, Sc=1, T = 1)\}$$

$$= \frac{P(D = 1, Sy=0|Sc = 0)}{P(D = 1, Sy=0|Sc = 1)} \frac{P(Sc=0)}{P(Sc=1)} \{P(D = 0, Sy = 0, Sc=1, T = 0)+0\}$$

$$= \frac{P(D = 1, Sy=0|Sc = 0)}{P(D = 1, Sy=0|Sc = 1)} \frac{P(Sc=0)}{P(Sc=1)} P(Sy = 0 | T = 0) P(T = 0)$$
